# Catalyst shuttling enabled by a thermoresponsive polymeric ligand: facilitating efficient cross-couplings with continuously recyclable ppm levels of palladium†
†Electronic supplementary information (ESI) available. See DOI: 10.1039/c9sc02171j


**DOI:** 10.1039/c9sc02171j

**Published:** 2019-07-23

**Authors:** Erfei Wang, Mao Chen

**Affiliations:** a State Key Laboratory of Molecular Engineering of Polymers , Department of Macromolecular Science , Fudan University , Shanghai 200433 , China . Email: chenmao@fudan.edu.cn ; http://chenmaofudan.wixsite.com/polymao

## Abstract

A polymeric monophosphine ligand WePhos has been synthesized and complexed with palladium(ii) acetate [Pd(OAc)_2_] to generate a thermoresponsive pre-catalyst that can shuttle between water and organic phases, with the change being regulated by temperature. The structure of the polymeric ligand was confirmed with matrix-assisted laser desorption/ionization-time-of-flight (MALDI-TOF) mass spectrometry and size-exclusion chromatography (SEC) analysis, as well as nuclear magnetic resonance (NMR) measurements. This polymeric metal complex enables highly efficient Pd-catalyzed cross-couplings and tandem reactions using 50 to 500 ppm palladium, and this can facilitate reactions that are tolerant to a broad spectrum of (hetero)aryl substrates and functional groups, as demonstrated with 73 examples with up to 99% isolated yields. Notably, 97% Pd remained in the aqueous phase after 10 runs of catalyst recycling experiments, as determined *via* inductively coupled plasma-atomic emission spectrometry (ICP-AES) measurements, indicating highly efficient catalyst transfer. Furthermore, a continuous catalyst recycling approach has been successfully developed based on flow chemistry in combination with the catalyst shuttling behavior, allowing Suzuki–Miyaura couplings to be conducted at gram-scales with as little as 10 ppm Pd loading. Given the significance of transition-metal catalyzed cross-coupling and increasing interest in sustainable chemistry, this work is an important step towards the development of a responsive catalyst, in addition to having high activity, by tuning the structures of the ligands using polymer science.

## Abstract 2

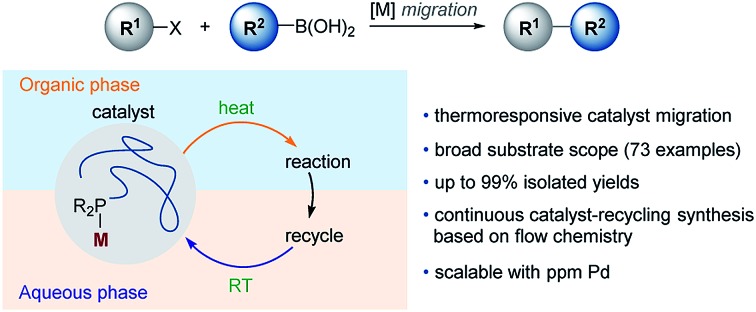
A thermoresponsive polymeric Pd-complex was synthesized, enabling highly efficient cross-couplings and continuous catalyst-recycling flow reactions with ultralow Pd usages.

## Introduction

Transition-metal catalyzed cross-coupling reactions have become indispensable tools in organic synthesis. The palladium (Pd)-catalyzed Suzuki–Miyaura (SM) coupling is one of the most heavily used reactions for C–C bond formation,^1^–^3^ and has revolutionized synthetic approaches for pharmaceuticals,^4^ agrochemicals,^5^ polymer materials^6^ and so on.^7^ Notwithstanding its extraordinary advances, the cost of Pd and its separation, as well as the sustainability of this chemistry are almost always concerns when using SM coupling, especially in process research and development; these are also concerns with other precious metal catalyzed reactions.^8^–^10^ Consequently, methods featuring low Pd loading,^11^–^16^ recyclable catalysts^17^,^18^ and aqueous conditions^19^–^21^ have received increasing attention. For example, Lipshutz and co-workers disclosed that the ppm levels of Pd contained in the iron(iii) chloride (FeCl_3_) salt catalyzed aqueous SM coupling with a dialkylphosphine ligand.^11^ They also reported that SM couplings could be facilitated using amphiphilic molecules in water (H_2_O).^12^,^22^,^23^ Based on previous achievements of using electron-rich phosphine ligands to promote SM couplings,^24^–^31^ Carrow and co-workers revealed that a tri(adamantyl)phosphine–Pd complex catalyzed a process with low Pd loadings in an organic solvent.^13^ Whereas polymeric materials loaded with Pd are easily recyclable for homo/heterogeneous SM couplings,^32^–^42^ in comparison to small molecule catalysts, polymeric catalysts generally exhibit: (a) limited functional group tolerance, (b) decreased reactivity toward deactivated aryl chlorides, and (c) unsatisfactory compatibility with heterocycles.^43^ Furthermore, tandem/iterative catalysis based on chemoselective SM coupling^44^–^46^ has rarely been realized with polymeric catalysts.

**Scheme 1:**
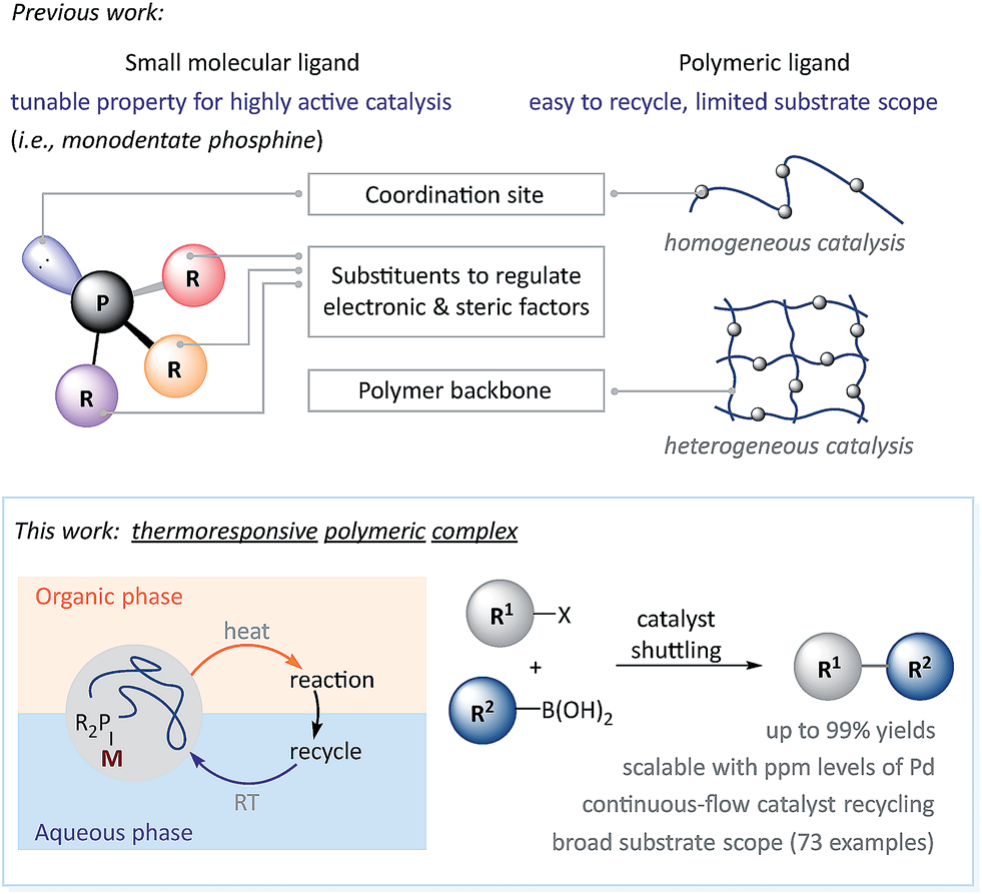
Ligand design in transition-metal catalyzed cross-coupling.

In this research, a novel polymeric pre-catalyst was synthesized, which was composed of methoxy poly(ethylene glycol) (PEG) linked dicyclohexylphosphine ligand (WePhos) and Pd, which enables a general, efficient and low Pd loading SM coupling *via* a rapid catalyst transfer between aqueous and toluene phases (Scheme 1). This polymeric complex catalyzed reaction is tolerant of various functional groups as well as (hetero)aromatic rings, and is realized in tandem coupling with high chemoselectivity. Furthermore, taking advantage of the thermoresponsive catalyst shuttling, a continuous catalyst-recycling approach based on flow chemistry was developed to streamline scalable SM couplings using down to 10 ppm of Pd.

The lower critical solution temperature (LCST) behaviour of PEG is known in polymer science,^47^–^50^ with PEGylated materials exhibiting dramatically decreased solubility in water upon heating above the LCST. It was thought that by introducing a PEGylated phosphine ligand, the corresponding Pd complex would display a thermoresponsive solubility in a biphasic system, thereby promoting a reaction in the organic phase and facilitating catalyst recycling simply by phase separation.

## Results and discussion

**Fig. 1:**
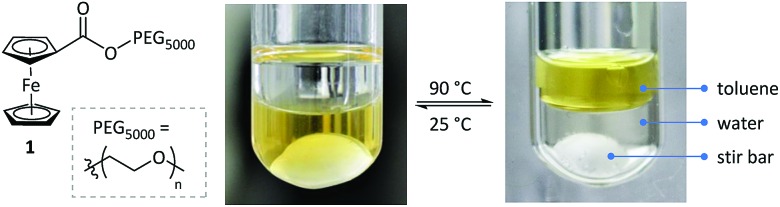
Optical images of the migration behavior of compound **1** between water and toluene phases in response to temperature.

At the beginning of the investigation, to confirm and visualize the shuttling effect, a yellow polymeric compound **1** was synthesized with PEG_5000_ (number average molecular weight, *M*_n_ ∼ 5000 g mol^–1^) and carboxyferrocene (Fig. 1). When **1** is dissolved in a mixture of water and toluene at 25 °C, only the water phase shows yellow coloration, however, upon heating to 90 °C, the toluene phase turns yellow within 30 s. The whole process is rapid and completely reversible, clearly exhibiting the thermoresponsive shuttling of a PEG-supported compound between water and organic phases.

**Scheme 2:**
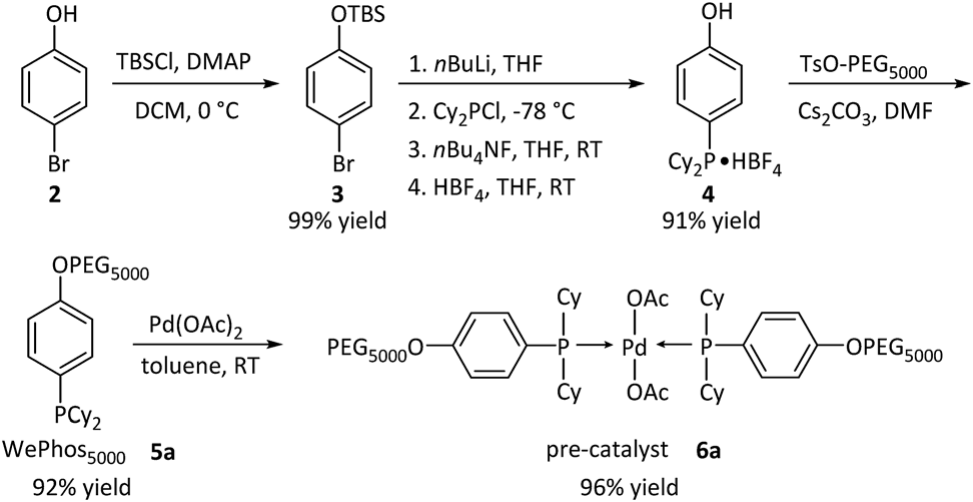
Synthetic pathway of WePhos_5000_**5a** and pre-catalyst **6a**; Cy = cyclohexyl group, AcO = acetate group.

**Fig. 2:**
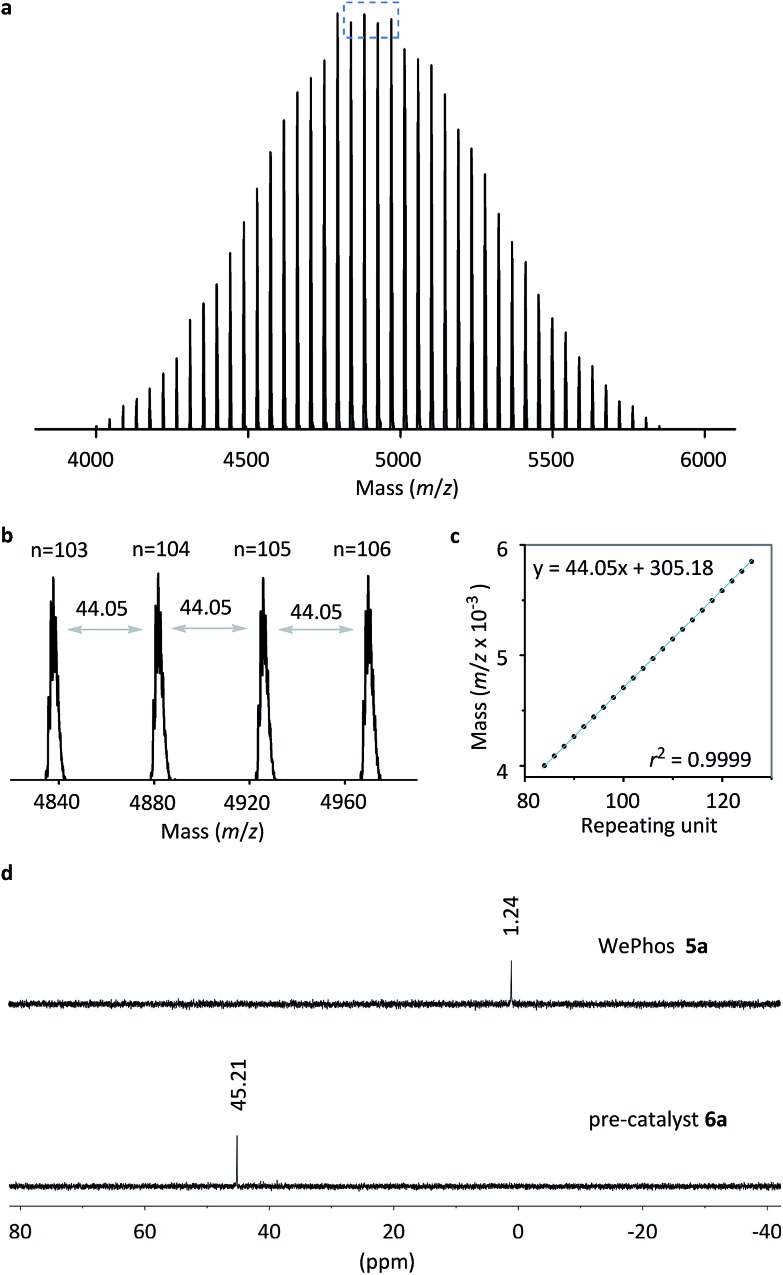
(a) MALDI-TOF mass spectrum of WePhos_5000_**5a**. (b) Magnified MALDI-TOF mass spectrum of the blue box in (a). (c) *m*/*z versus* the number of repeating units for the MALDI-TOF mass spectrum of **5a**. (d) ^31^P-NMR spectra of **5a** and **6a**.

Subsequently, the new ligand WePhos_5000_**5a** was synthesized using a synthetic route illustrated in Scheme 2. Starting from 4-bromophenol **2**, a high overall yield of 83% was obtained for ligand **5a**. This compound was air stable, and was characterized using matrix-assisted laser desorption/ionization-time-of-flight (MALDI-TOF) mass spectrometry. As shown in Fig. 2a, a single set of peaks is observed in the MALDI-TOF mass spectrum, and each peak is separated by the molar mass of a single repeating unit in PEG (*m*/*z* = 44.05, Fig. 2b). The absolute *m*/*z* value was consistent with the calculated molecular weight of **5a**. The *y*-intercept of the best-fit trend line of *m*/*z versus* the number of repeating units indicated the molecular weight of the chain-end group of **5a** (Fig. 2c), which was consistent with the expected value. The proton-nuclear magnetic resonance (^1^H-NMR) analysis^51^ also confirmed the chemical structure of WePhos_5000_. Mixing **5a** with palladium(ii) acetate [Pd(OAc)_2_] in a 2 : 1 ratio, quantitatively generated pre-catalyst **6a**. A peak shift from 1.24 to 45.21 ppm in the ^31^P-NMR spectra revealed the successful coordination of phosphine to the Pd^II^ center (Fig. 2d).^52^,^53^

**Fig. 3:**
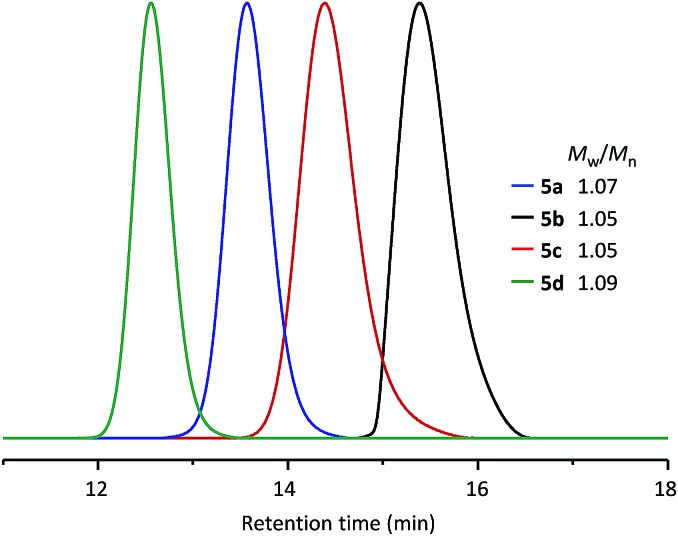
SEC results for WePhos ligands of different molecular weights.

Using the same synthetic method, a series of WePhos ligands were prepared using PEGs of different molecular weights (*M*_n_ ∼ 750 g mol^–1^ for **5b**, *M*_n_ ∼ 2000 g mol^–1^ for **5c**, *M*_n_ ∼ 10 000 g mol^–1^ for **5d**). When these ligands were characterized using size-exclusion chromatography (SEC) (Fig. 3), the SEC profiles showed clear shifts from high to low retention times with no low molar mass tailing (molecular weight distribution, weight average molecular weight/number average molecular weight (*M*_w_/*M*_n_) = 1.05–1.09), which indicated the successful preparation of the polymeric ligands with a precise structure.

**Fig. 4:**
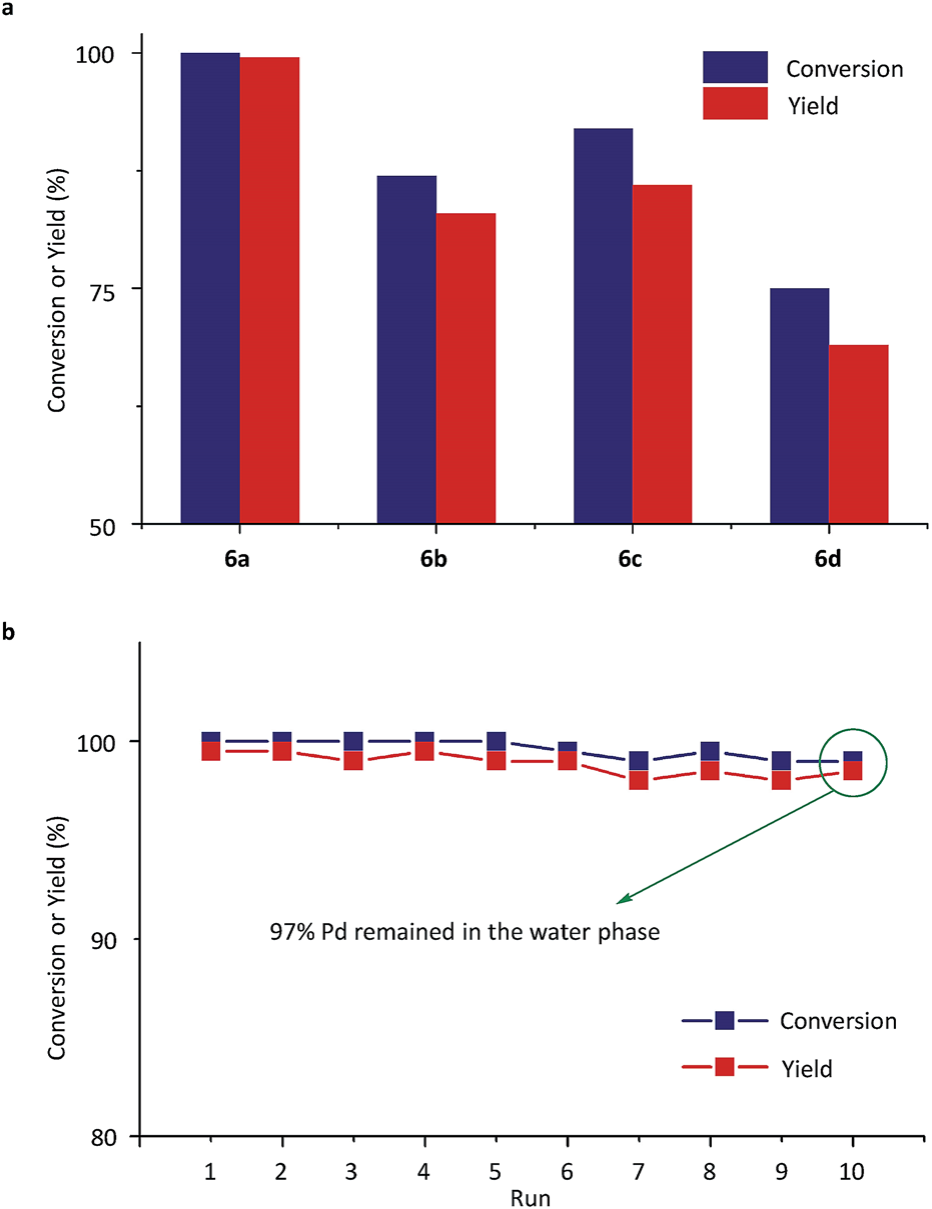
(a) Conversions and yields of SM cross-couplings between bromobenzene and 4-methoxybenzeneboronic acid with pre-catalysts **6a**, **6b**, **6c** and **6d**. (b) Conversions and yields of target product (4-methoxybiphenyl) for the catalyst recycling experiments with **6a**. Conversions and yields were determined using GC analysis.

Next, Pd-complexes of ligands from **5a** to **5d** were used in the SM cross-coupling of bromobenzene and 4-methoxybenzeneboronic acid with potassium carbonate (K_2_CO_3_) as the base in water/toluene (v/v = 4/1) at 90 °C. As summarized in Fig. 4a, when the pre-catalyst **6a** was used, the reaction gave the highest yield, in a reaction time of 20 min, determined using gas chromatography (GC, yield > 99%).^54^ After cooling down the reaction mixture with **6a** to room temperature (RT), the metal-complex catalyst was transferred to the aqueous layer and the 4-methoxybiphenyl product was left in the toluene. Using a simple phase separation, the aqueous layer was collected and reused in the next SM cross-coupling reaction. The water phase containing the catalyst was recycled 10 times in this way and there was no clear decrease in GC yields (Fig. 4b). The final aqueous phase was analysed using inductively coupled plasma-atomic emission spectrometry (ICP-AES), which indicated that 97% of the Pd remained in the aqueous phase, which highlighted the robust catalyst shuttling facilitated by the thermoresponsive polymeric ligand.

**Scheme 3:**
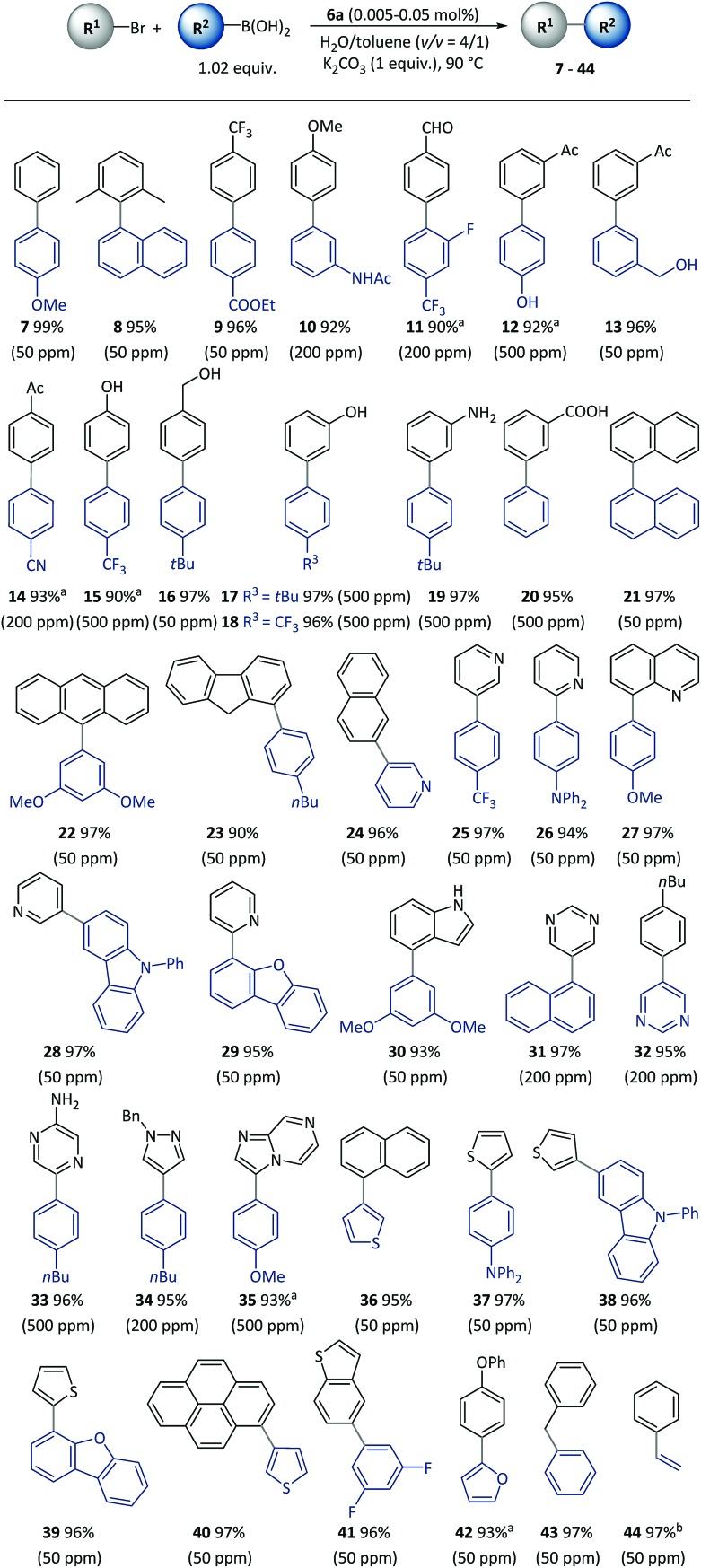
Cross-coupling of (hetero)aryl bromides using pre-catalyst **6a**. Conditions: R^1^-Br (0.5 mmol), R^2^-B(OH)_2_ (0.51 mmol), **6a** (50–500 ppm), K_2_CO_3_ (0.5 mmol), H_2_O (0.8 mL), toluene (0.2 mL), 90 °C, (30 min). Catalyst loadings are listed in parentheses. Isolated yields are based on aryl bromides. ^a^2 h. ^b^R^2^-BPin (boronic acid pinacol ester) was used instead of R^2^-B(OH)_2_.

With the optimized reaction conditions resolved, the substrate scope of the (hetero)aryl bromides was investigated (Scheme 3). All the substrates underwent complete conversion in 30 min to 2 h, giving products with good to excellent isolated yields with 50 to 500 ppm of catalyst. The SM reactions catalyzed with polymeric complexes could be successfully carried out with *para*-, *meta*-, and *ortho*-substituted aryl compounds and fused aryl compounds. Electron-deficient, electron-neutral and electron-rich aryl halides were all suitable substrates. The reaction conditions were compatible with a broad scope of functional groups including ester (**9**), amide (**10**), aldehyde (**11**), acetyl (**12–14**), and nitrile (**14**), as well as unprotected hydroxyl (**13–18**), amino (**19**) and carboxylic acid (**20**) groups. As is already known, heterocycles are core units of many important pharmaceuticals, organic materials and natural products.^55^ However, heterocycles are likely to coordinate with transition metals, thus representing a challenging class of starting materials for metal-catalyzed reactions with low catalyst dosages.^56^ With this method, a variety of heterocyclic compounds including pyridine (**24–28**), quinoline (**29**), carbazole (**27**), dibenzofuran (**28**), indole (**30**), pyrimidine (**31–32**), pyrazine (**33**), pyrazole (**34**), imidazo[1,2-*a*]pyrazine (**35**), thiophene (**36–40**), benzothiophene (**41**), and furan (**42**), could be used to generate corresponding products, representing the broadest substrate scope obtained with a polymeric catalyst as far as is known.

**Scheme 4:**
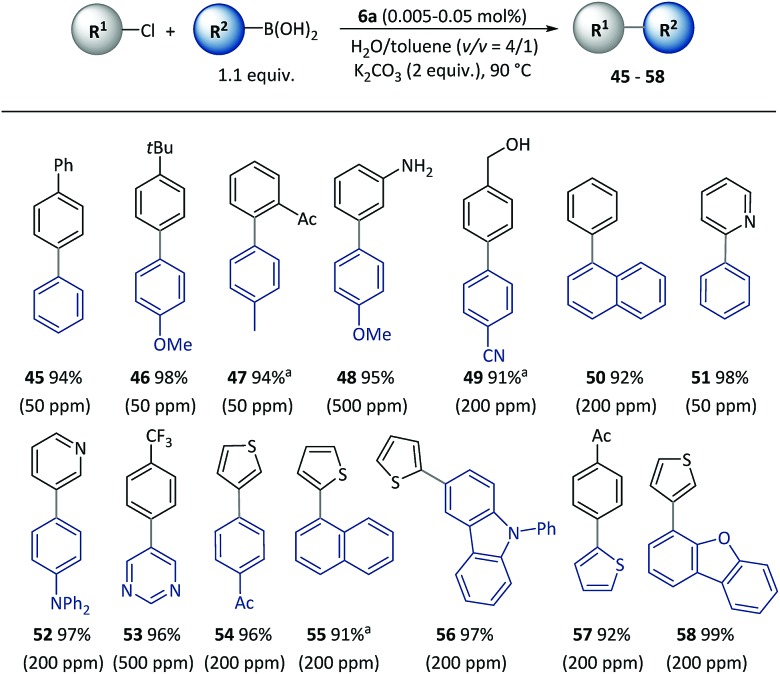
Cross-coupling of (hetero)aryl chlorides with pre-catalyst **6a**. Conditions: R^1^-X (0.5 mmol), R^2^-B(OH)_2_ (0.55 mmol), **6a** (50–500 ppm), K_2_CO_3_ (1.0 mmol), H_2_O (0.8 mL), toluene (0.2 mL), 90 °C, 2 h. Catalyst loadings are listed in parentheses. Isolated yields are based on aryl chlorides. ^a^5 h.

Furthermore, the SM cross-coupling using pre-catalyst **6a** was found to be applicable to (hetero)aryl chlorides with a wide substrate scope as shown in Scheme 4. Aryl chlorides with a substituent group at different positions could effectively react with aryl boronic acids (**45–48**) to give biaryl compounds with yields of 94–98%. Functional groups [for example, acetyl (**47**), unprotected amino (**48**) and hydroxyl (**49**)] and heterocycles including pyridine (**51–52**), pyrimidine (**53**), thiophene (**54–58**) and dibenzofuran (**58**) underwent a smooth reaction with a low (50–500 ppm) catalyst loading, and there were complete conversions in 2–5 h.

**Scheme 5:**
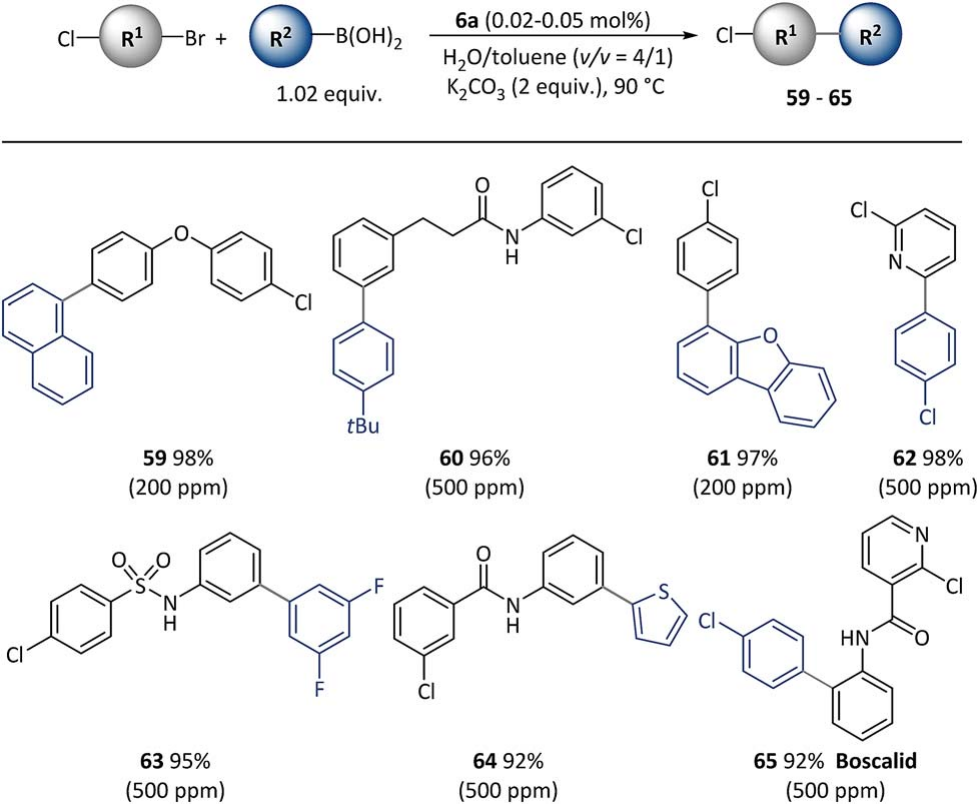
The chemoselective cross-coupling of conjunctive dihalide compounds using pre-catalyst **6a**. Conditions: Cl-R^1^-Br (0.5 mmol), R^2^-B(OH)_2_ (0.51 mmol), **6a** (50–500 ppm), K_2_CO_3_ (1.0 mmol), H_2_O (0.8 mL), toluene (0.2 mL), 90 °C, 2 h. Catalyst loadings are listed in parentheses. Isolated yields are based on conjunctive dihalide compounds.

Given the different reactivities of C–Br and C–Cl bonds demonstrated in Pd catalyzed cross-coupling with many small molecule catalysts, the chemical selectivity of polymeric catalyst **6a** was next evaluated, as shown in Scheme 5. Excellent selectivity was observed in all the cases examined. A variety of functionalized bi(hetero)aryl were produced with intact aryl chlorides groups (**59–65**) at above 90% isolated yields, which could be useful in further metal-catalyzed transformations. It is important to note that the chemoselectivity was not affected by steric or electronic factors with use of the polymeric catalyst.

**Scheme 6:**
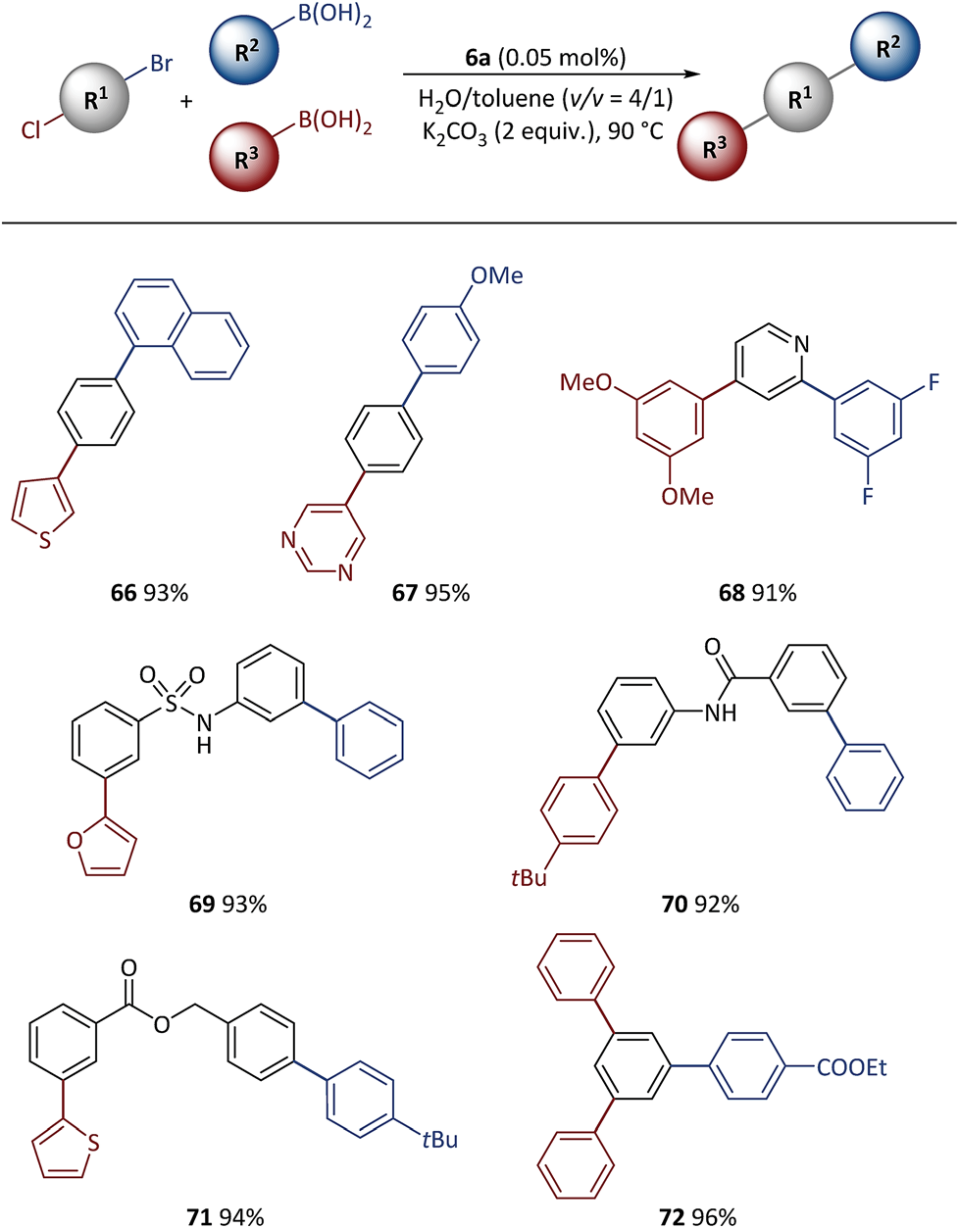
Tandem SM cross-coupling with pre-catalyst **6a**. Conditions: R^1^-X (0.5 mmol), R^2^-B(OH)_2_ (0.51 mmol), R^3^-B(OH)_2_ (0.55 mmol), **6a** (500 ppm), K_2_CO_3_ (1.0 mmol), H_2_O (0.8 mL), toluene (0.2 mL), 90 °C. Isolated yields based on conjunctive dihalide compounds.

Tandem SM coupling based on chemoselective reaction facilitated modular and rapid synthesis of diversified molecules for applications such as material screening and drug discovery.^44^–^46^,^57^–^59^ Next, the polymeric catalyst was used in tandem SM coupling based on the different reactivities of electrophiles (Scheme 6). In these reactions, after the first step arylation *via* C–Br bond cleavage, boronic acids were subsequently added into the reaction mixtures without additional catalyst and base. Excellent yields (91–96%) were obtained for all examples after two-step syntheses in the presence of heterocycles [for example, thiophene (**66**), pyrimidine (**67**), pyridine (**68**) and furan (**69**)] and functional groups [for example, sulfonamide (**69**), amide (**70**) and ester (**71**, **72**)], demonstrating the high efficiency achieved with this method.

**Fig. 5:**
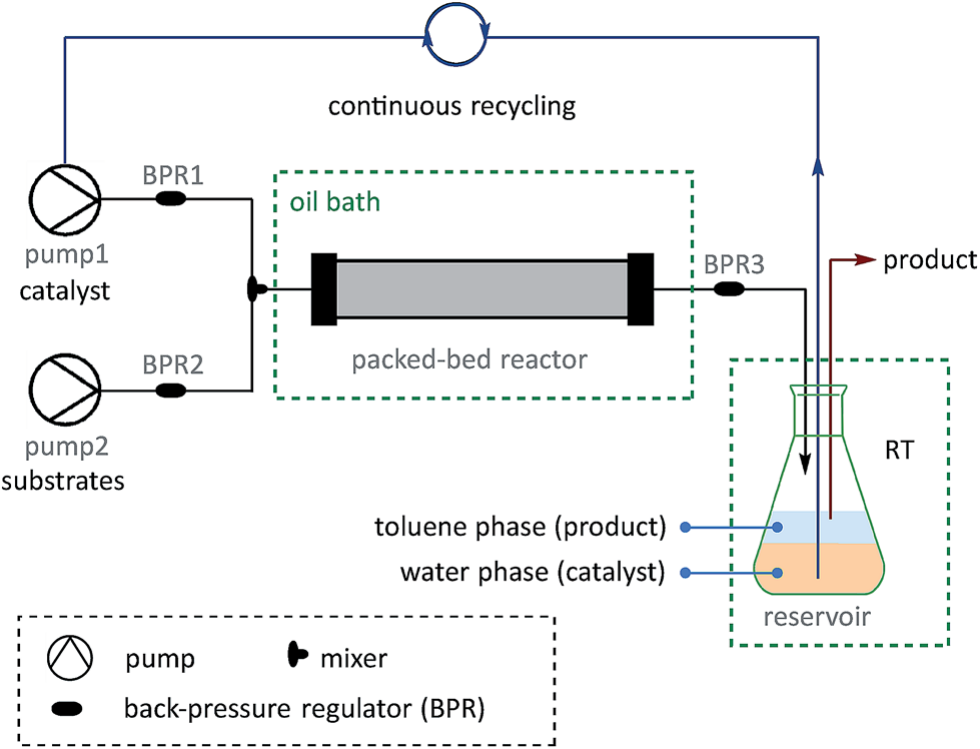
Set-up for continuous catalyst-recycling synthesis based on flow chemistry.

**Scheme 7:**
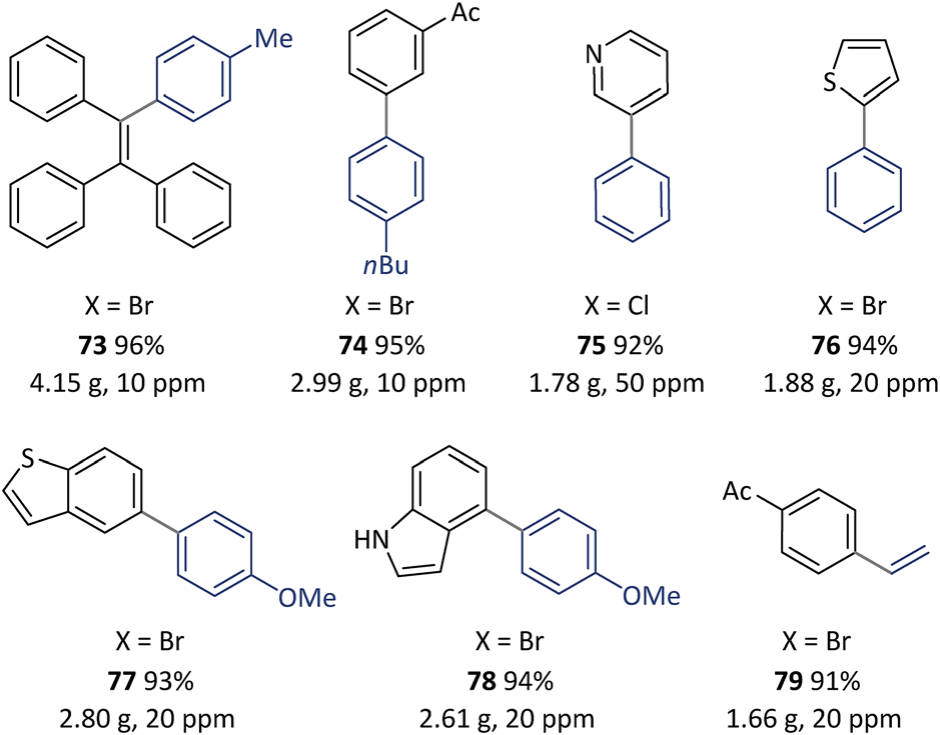
Examples of SM couplings with the continuous catalyst-recycling approach based on flow chemistry. Collections were based on 12.5 mmol aryl halides for all examples. The Pd loadings were calculated according to the molar ratio of [Pd]/[aryl halide]. See the ESI† for details of the flow set-up and operations.

On the basis of the thermoresponsive catalyst migration, it was anticipated that a continuous-flow synthetic strategy^60^–^67^ would be practical for scaling-up the SM couplings at ultralow Pd loadings by continuous recycling of catalyst. To realize this idea, a continuous-flow setup was assembled (Fig. 5 and Scheme 7) to synthesize **73**, a typical unit with aggregation-induced emission (AIE) behavior, which has been used in high-tech applications such as optoelectronic materials and biomedical sensors.^68^

A solution of pre-catalyst and base in water was mixed with a solution of bromotriphenylethylene and 4-methylphenylboronic acid pinacol ester in toluene, and delivered into a packed bed reactor (packed with stainless steel beads)^51^ submerged in a preheated bath at 110 °C with a 30 min residence time. After the reaction, the resulting mixture was collected in a flask under a nitrogen atmosphere at RT. The water layer containing the catalyst was continuously extracted using pump 1 and then reinjected into the flow line. In this way, after 8.3 h of catalyst recycling, 4.15 g of **73** was isolated from the collected organic layer using only 1.3 mg of pre-catalyst **6a** (13.8 μg Pd).

Once this flow technique was available, other bi(hetero)aryl compounds (**74–78**) and substituted styrene (**79**) were also successfully synthesized at the gram scale with 10–50 ppm of Pd. Based on the excellent behavior of the continuous catalyst-recycling synthesis using flow chemistry, it is believed that a larger scale preparation could be realized by extending the collection time without adding extra catalyst.

## Conclusions

In conclusion, a novel thermoresponsive polymeric catalyst was developed, which can rapidly shuttle between water and organic phases, facilitating a highly efficient SM cross-coupling and tandem reaction with good to excellent isolated yields at ppm levels of catalyst usage. This method allows the preparation of a broad scope of bi(hetero)aryls, and can tolerate various functional groups. Furthermore, in combination with flow chemistry, the catalyst shuttling enables continuous catalyst-recycling, further promoting the scalability and efficiency of cross-coupling using ultralow loadings of palladium. Given the significant influence of transition-metal-catalyzed cross-coupling and increasing interest in sustainable chemistry, it is believed that, based on the strategy presented here, new response modes can be developed by tuning the structures of the ligands using different polymeric species. Also, the compatibility of this method with other metal-catalyzed reactions is under investigation, which would show promise for facilitating other diverse applications.

## Conflicts of interest

There are no conflicts to declare.

## Supplementary Material

Supplementary information
