# Supplementary material for: Catalyst shuttling enabled by a thermoresponsive polymeric ligand: facilitating efficient cross-couplings with continuously recyclable ppm levels of palladium
Source: Chem Sci. 2019 Jul 23;10(36):8331–7. doi: 10.1039/c9sc02171j (PMC6844270; doi:10.1039/c9sc02171j)
Supplement: Supplementary file 1 [file SC-010-C9SC02171J-s001.pdf]

Electronic Supplementary Material (ESI) for Chemical Science.
This journal is © The Royal Society of Chemistry 2019

Supplementary Information

Table of Contents

1. General information

2. Synthesis and characterization of compound 1 in Fig. 1

3. Synthesis and characterization of pre-catalyst 6 in Scheme 2

4. Experiments of condition optimization, catalyst recycling and scale-up

5. Synthesis and characterization of substrates

6. General procedure and characterization results for Scheme 3, 4, 5

7. General procedure and characterization results for Scheme 6

8. General procedure and characterization results for Scheme 7

9. Reference

10. NMR Spectra

1. General information

1.1 General reagent information

Tetrahydrofuran (THF) was distilled from sodium before use. Dichloromethane (DCM) was distilled from CaH_2 before use. Dimethylformamide (DMF) was distilled from NaH before use. Other reagents and solvents were purchased from Sigma-Aldrich, Adamas or Aladdin Chemical, and used without further purification.

1.2 General analytical information

Nuclear magnetic resonance (NMR) was recorded on an Advance III 400 MHz Bruker spectrometer at 298 K. ^{1}H NMR signals were measured relative to the signal for residual chloroform (7.26 ppm) in deuterochloroform ($CDCl_3$) or methanol (3.31 ppm) in deuteromethanol (CD_3OD), and are reported in δ units, parts per million (ppm). ^{13}C NMR signals are reported in ppm units relative to $CDCl_3$ (77.16 ppm) or CD_3OD (49.00 ppm), and were obtained with 1H decoupling. Copies of ^{1}H NMR and ^{13}C NMR spectra of unknown compounds can be found at the end of the supporting information. ^{31}P NMR signals are reported in ppm units. Size exclusion chromatography (SEC) measurements were performed in THF at 35°C with an elution rate of 0.35 mL/min on a TOSOH instrument equipped with a Bryce refractive index detector. Three columns were employed, including one 6 μm superMultipore HZ-H gel column and two 4 μm superMultipore HZ-M columns. The calibration was performed with polystyrene standards. Matrix-assisted laser desorption ionization time of flight (MALDI-TOF) mass spectrometry was conducted on an AB SCIEX 5800 MALDI-TOF/TOF mass instrument. Gas chromatography (GC) measurements were carried out on SHIMADZU GC-2014 instrument using achiral capillary columns. Inductively coupled plasma–atomic emission spectrometer (ICP-AES) was characterized by a LEEMAN Prodigy instrument. Melting points (m.p.) were taken on a SG X-4 capillary melting point apparatus. High resolution mass spectra (HRMS) were measured with a Waters Micromass GCT instrument. Products were purified by flash column chromatography using silica gel (230-400 mesh).

2. Synthesis and characterization of compound 1 in Fig. 1

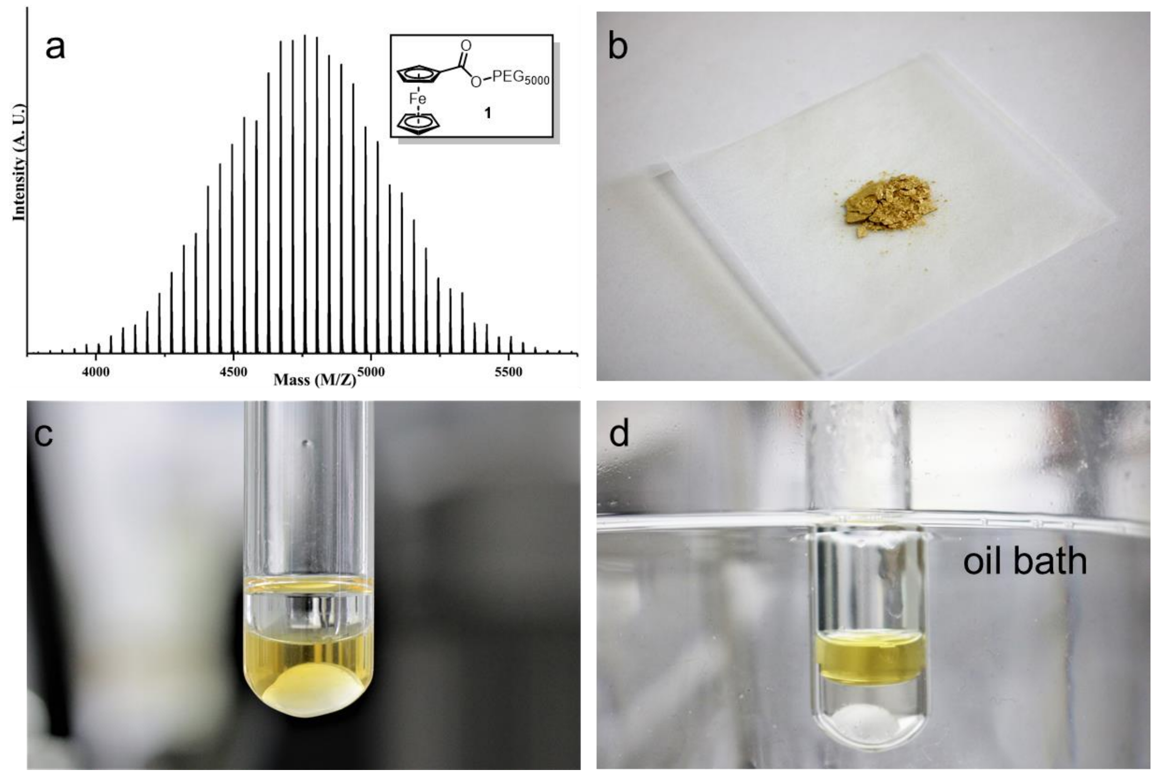

Fig. S1 (a) MALDI-TOF mass spectrum of compound **1**. (b) Optical image of compound **1**. (c) and (d) Optical images of the thermoresponsive migration behavior of compound **1** in water and toluene. When the temperature is at 25 °C, compound **1** is in the water phase; when the temperature is increased to 90 °C, compound **1** migrates to the toluene layer. The thermoresponsive migration is completely reversible as controlled by temperature.

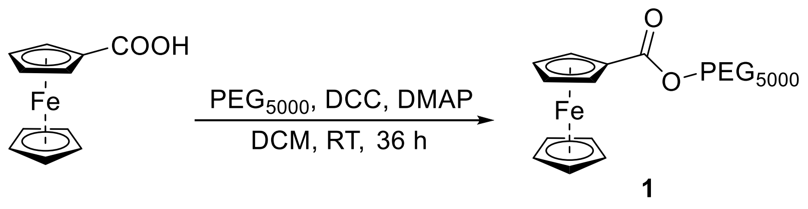

Methoxy poly(ethylene glycol) (PEG_{5000}, 232.5 mg, 0.05 mmol), carboxyferrocene (46.0 mg, 0.2 mmol), dimethylaminopyridine (DMAP, 6.1 mg, 0.05 mol) and dicyclohexylcarbodiimide (DCC, 41.2 mg, 0.2 mmol) were dissolved

in 2 mL DCM in a 10 mL round bottom flask equipped with a stir bar. The reaction mixture was stirred at room temperature (RT = 25 °C) for 24 h. After reaction, the reaction mixture was diluted with dichloromethane, and washed with saturated aqueous NH_4Cl solution for three times. Water phase was extracted with DCM for two times. Combined organic layers were dried over anhydrous Na_2SO_4, concentrated under reduced pressure. Obtained residue was added into 100 mL cold ethyl ether to give product as a precipitate. Collected product was dried under vacuum to afford a yellow solid (232.1 mg, 96%). The compound was characterized by MALDI-TOF mass spectrometry (Fig. S1a), ^1H and ^{13}C NMR measurements. ^1H NMR (400 MHz, $CDCl_3$) δ: 4.83 (s, 2 H), 4.55-2.93 (m, 405 H), 2.52-1.76 (m, 9 H) ppm. ^{13}C NMR (100 MHz, $CDCl_3$) δ: 71.9, 71.3, 70.6, 70.2, 69.8, 69.4, 63.3, 59.0 ppm.

3. Synthesis and characterization of pre-catalysts 6 in Scheme 2

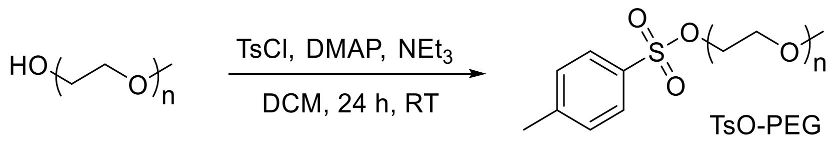

A 100 mL Schlenk flask equipped with a stir bar was charged with PEG_{5000} (4.65 g, M_n ~ 5000 g/mol), triethylamine (NEt_3, 280 μL, 2 mmol), *p*-tosylchloride (TsCl, 0.57 g, 3 mmol), DMAP (12.2 mg, 0.1 mmol) and 20 mL anhydrous DCM under N_2. The reaction mixture was stirred at room temperature for 24 h. After reaction, the solution was diluted with DCM and washed with saturated aqueous NH_4Cl solution for three times. The water phase was extracted with DCM. Combined organic layers were dried over anhydrous Na_2SO_4, concentrated under reduced pressure. The obtained residue was added into 400 mL cold diethyl ether to give product as a precipitate. The collected solids were dried under vacuum to afford target product[1] (4.71 g, 98%) as a white powder. The SEC results of PEG_{5000} and TsO-PEG_{5000} were showed in Fig. S2a and S2b, respectively. TsO-PEG_{10000}, TsO-PEG_{2000} and TsO-PEG_{750} were synthesized with an analogous procedure

using PEG_{10000} (M_n ~ 10000 g/mol), PEG_{2000} (M_n ~ 2000 g/mol), PEG_{750} (M_n ~ 750 g/mol) instead of PEG_{5000}.

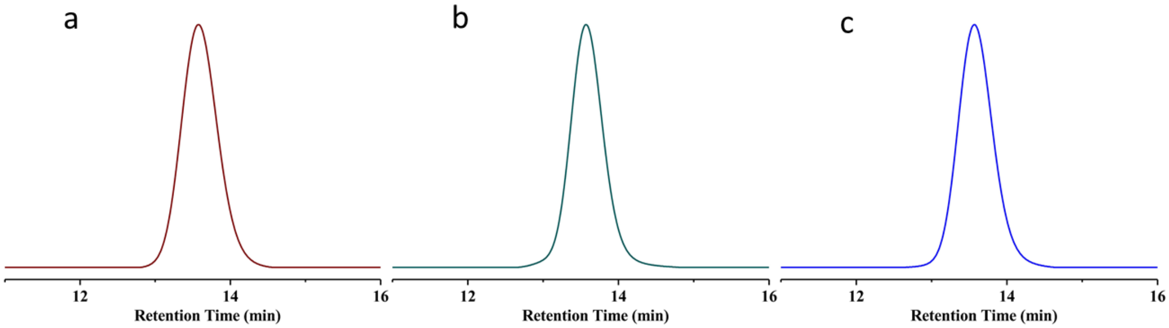

Fig. S2 SEC results of PEG_{5000} (a), TsO-PEG_{5000} (b) and $WePhos_{5000}$ (c).

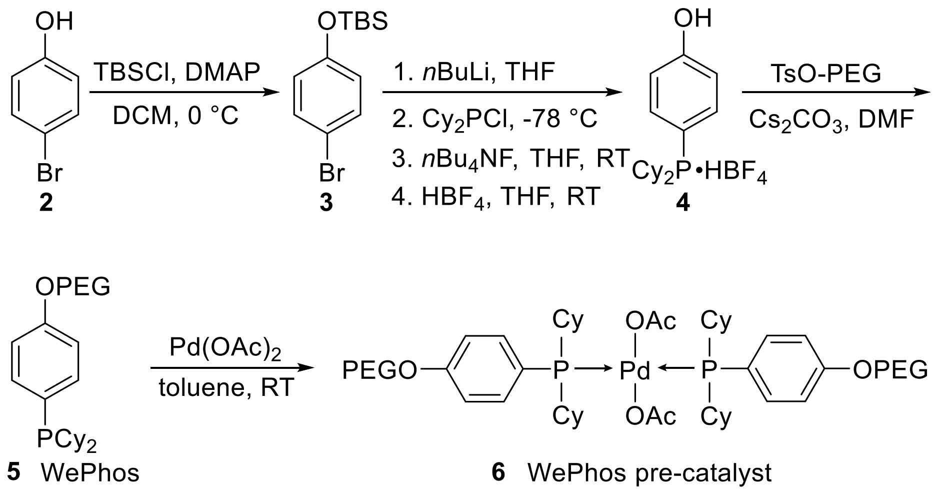

4-Bromophenol **2** (8.00 g, 46.2 mmol) and DMAP (0.56 g, 4.6 mmol) were dissolved in 120 mL DCM in a 250 mL round bottom flask equipped with a stir bar at 0 °C. After stirring for 15 min, imidazole (4.72 g, 69.4 mmol) and *t*-butyldimethylsilyl chloride (TBSCl, 10.45 g, 69.4 mmol) were added into the flask, and the mixture was stirred at 0 °C for 1 h. After reaction, DCM was removed using a rotary evaporator, and the resulting mixture was dissolved in 100 mL Et_2O. The obtained solution was washed with ammonium hydroxide, water and brine. Then, the solution was concentrated under vacuum to give compound **3**[2] (13.20 g, 99%)

as a colorless oil, which was directly used in the next step of synthesis.

A 100 mL Schlenk flask equipped with a stir bar was charged with compound **3** (1.44 g, 5.0 mmol) and 10 mL anhydrous THF under N_2. A solution of *n*-butyllithium (*n*BuLi) in *n*-hexanes (2.5 M, 2.4 mL) was added dropwise into this flask at -78 °C during 10 min. The mixture was stirred at -78 °C for 1 h. Then, chlorodicyclohexylphosphine (Cy_2PCl, 1.16 g, 5.0 mmol) was added dropwise via a syringe into the flask under N_2. The reaction mixture was stirred at -78 °C for 12 h. After moving the flask to room temperature, tetrabutylammonium fluoride (nBu_4NF, 1 M in THF, 10 mL) was added dropwise into the mixture, and the mixture was stirred at room temperature for 2 h. After reaction, 2 mL HBF_4 (7.6 M) and 10 mL H_2O were added into the flask. The mixture was stirred at room temperature for 1 h. Then, the mixture was concentrated under vacuum to remove organic solvents. The resulted mixture was extracted with DCM for three times. The combined organic layers were dried over Na_2SO_4 and concentrated under vacuum. The obtained residue was purified by column chromatography (eluting with 0-5% MeOH in DCM) to give compound **4** (1.34 g, 91%) as a white solid. ^1H NMR (400 MHz, CD_3OD) δ: 7.98-7.34 (m, 2 H), 7.05 (m, 2 H), 3.31 (s, 1 H), 2.78 (br, 1 H), 2.04 (m, 2 H), 1.91-1.69 (m, 7 H), 1.57-1. 07 (m, 11 H) ppm. ^{13}C NMR (100 MHz, CD_3OD) δ: 136.1, 136.0, 117.2, 117.1, 28.3, 27.9, 26.9, 25.8, 25.3, 24.9 ppm. ^{31}P NMR (162 MHz, CD_3OD) δ: 25.65 ppm. HRMS (ESI): m/z calculated for $C_{18}H_{28}OP^+$ [M+H$^+$]: 291.1872, found: 291.1873.

A 50 mL Schlenk flask equipped with a stir bar was charged with TsO-PEG$_{5000}$ (2.40 g, 0.5 mmol), compound **4** (0.57 g, 1.5 mmol), cesium carbonate (1.30 g, 4.0 mmol) and 10 mL DMF under N_2. The mixture was stirred at 100 °C for 72 h. After reaction, the mixture was cooled to room temperature and treated with 10 mL H_2O and stirred at room temperature for 1 h. The resulting mixture was extracted with DCM for three times. The combined organic layers were dried over Na_2SO_4 and concentrated under vacuum. Obtained residue was added into cold diethyl ether to give a precipitate, which was dried under vacuum to give WePhos$_{5000}$ **5a** (2.26 g, 92%) as a white powder, and the compound was characterized by MALDI-TOF mass spectrometry (Fig. 2a), SEC (Fig. S2c), ^1H, ^{13}C NMR and ^{31}P NMR

measurements. ^{1}H NMR (400 MHz, $CDCl_3$) δ: 8.03 (s, 2 H), 7.37 (s, 2 H), 5.01-2.66 (m, 385 H), 2.32-0.82 (m, 39 H) ppm. ^{13}C NMR (100 MHz, $CDCl_3$) δ: 71.9, 70.5, 58.7, 23.9, 19.6, 13.6 ppm. ^{31}P NMR (162 MHz, $CDCl_3$) δ: 1.24 ppm. $WePhos_{10000}$, $WePhos_{2000}$ and $WePhos_{750}$ were synthesized with an analogous procedure using TsO-PEG_{10000}, TsO-PEG_{2000} and TsO-PEG_{750} instead of TsO-PEG_{5000}.

An oven-dried 5 mL vial containing a magnetic stir bar was charged with $Pd(OAc)_2$ (22.4 mg, 0.1 mmol) and 0.5 mL toluene under N_2 atmosphere. The mixture was stirred at room temperature until $Pd(OAc)_2$ was completely dissolved. Another oven-dried 5 mL vial was charged a solution of $WePhos_{5000}$ **5a** (981.0 mg, 0.2 mmol) and 1 mL anhydrous toluene. This solution was added dropwise into previous vial containing $Pd(OAc)_2$ via a syringe over 5 min. Then, the mixture was stirred at room temperature for 12 h under N_2. After reaction, the mixture was added into cold diethyl ether to give the precipitate of pre-catalyst **6a**. This complex was further dried under vacuum to give **6a** as a brown powder in 96% yield (963.6 mg). ^{1}H NMR (400 MHz, $CDCl_3$) δ: 7.65-7.53 (m, 4H), 7.44-7.33 (m, 4H), 4.28-3.42 (m, 733H), 2.08-0.98 (m, 111H) ppm. ^{13}C NMR (100 MHz, $CDCl_3$) δ: 70.6, 58.6, 25.9, 23.8, 19.6, 13.6 ppm. ^{31}P NMR (162 MHz, $CDCl_3$) δ: 45.21 ppm.

4. Experiments of condition optimization, catalyst recycling and scale-up

4.1 General procedure to prepare the pre-catalyst solution

An oven-dried 2.0 mL volumetric vial was charged with pre-catalyst **6a** (25.0 mg, Table S1, entry 1). Then, deionized water was added into the vial to make the solution volume 2.0 mL. The volumetric flask was sonicated to accelerate the dissolution of solids. For the preparation of other pre-catalyst solutions, similar operations were used. Details of the amount of pre-catalysts can be found in Table S1. To carry out a coupling reaction at 0.5 mmol scale (based on aryl halide), 20 μL pre-catalyst solution was used for 0.005 mol% catalyst loading (50 ppm).

Table S1 Detailed information for the preparation of pre-catalyst solution

Entry	Ligand	Pre catalyst	Amount (10^{-3} mmol)	Weight (mg)	Total volume (mL)	[Cat.] (mmol/L)
1	$WePhos_{5000}$ **5a**	**6a**	2.5	25.0	2.0	1.25
2	$WePhos_{750}$ **5b**	**6b**	2.5	3.8	2.0	1.25
3	$WePhos_{2000}$ **5c**	**6c**	2.5	10.0	2.0	1.25
4	$WePhos_{10000}$ **5d**	**6d**	2.5	50.0	2.0	1.25

4.2 Screening pre-catalysts of WePhos ligands of different molecular weights

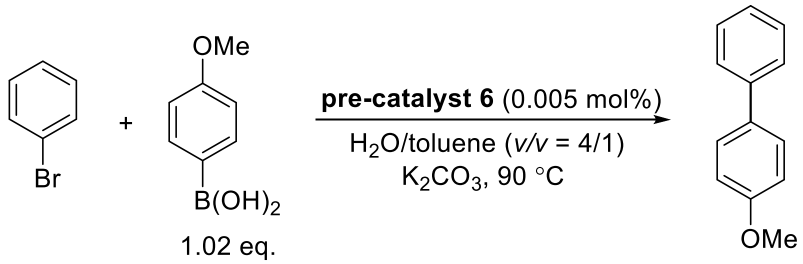

General procedure (taking pre-catalyst ***6a*** *as an example):*

A 10 mL Schlenk tube equipped with a stir bar was charged with 4-methoxyphenylboronic acid (77.5 mg, 0.51 mmol), K_2CO_3 (69.0 mg, 0.5 mmol), pre-catalyst **6a** (20 μL pre-catalyst solution in Table S1, entry 1), 0.2 mL toluene and 0.8 mL deionized H_2O under N_2 atmosphere. Bromobenzene (78.5 mg, 0.5 mmol) was added into this tube via a microsyringe under N_2. Then, the mixture was stirred at 90 °C. After 20 min, the reaction was cooled to room temperature. Biphenyl was added into the mixture as an internal standard. The resulting mixture was extracted with ethyl acetate for three times. The combined organic layers were dried over Na_2SO_4. A small aliquot of the organic layer was filtered through a short

silica gel cartridge, and characterized with GC analysis to give GC yields of target product 4-methoxybiphenyl. Results are summarized in Fig. 4a.

4.3 Experiments of catalyst recycling

A 10 mL Schlenk tube equipped with a stir bar was charged with 4-methoxyphenylboronic acid (77.5 mg, 0.51 mmol), bromobenzene (78.5 mg, 0.5 mmol), K_2CO_3 (27.6 mg, 0.2 mmol), pre-catalyst **6a** (20 μL pre-catalyst solution in Table S1, entry 1), 0.2 mL toluene and 0.8 mL deionized H_2O under N_2 atmosphere. The mixture was stirred at 90 °C for 30 min. After reaction, the mixture was cooled to room temperature. Biphenyl was added into the mixture as an internal standard. The resulting mixture was extracted with toluene for three times. The combined organic layers were dried over Na_2SO_4. A small aliquot of the organic layer was filtered through a short silica gel cartridge, and characterized with GC analysis to give the GC yield of the first run as shown in Fig. 4a. Water layer was left in the Schlenk tube. In the second run, bromobenzene (78.5 mg, 0.5 mmol), 4-methoxyphenylboronic acid (77.5 mg, 0.51 mmol), K_2CO_3 (27.6 mg, 0.2 mmol) and 0.2 mL toluene was added into the Schlenk tube. The mixture was bubbled with N_2 for 5 min. Then, the mixture was stirred at 90 °C for 30 min. After reaction, internal standard of biphenyl was added into the mixture, followed by extracting the mixture with toluene for three times. The combined organic layers were employed in the GC analysis using the same procedure as shown for the first run. In the following experiments of catalyst recycling from the third to the tenth runs, the water phase containing Pd-catalyst was reused after phase separation using the same procedure as indicated with the second run. After catalyst recycling experiments of 10 runs in total, the final aqueous phase was diluted with 4 mL deionized H_2O and analyzed with ICP-AES measurement by the analysis and testing center of Shanghai Institute of Organic Chemistry (SIOC), indicating 97% Pd was remaining in the aqueous phase.

4.4 Experiments of scale-up

A 100 mL Schlenk flask equipped with a stir bar was charged with 4-

methoxyphenylboronic acid (1.55 g, 10.2 mmol), bromobenzene (1.57 g, 10 mmol), K_2CO_3 (1.38 g, 10 mmol), pre-catalyst **6a** (10.0 mg, 50 ppm), 4.0 mL toluene and 16.0 mL deionized H_2O under N_2 atmosphere. The mixture was stirred at 90 °C for 2 h. After reaction, the flask was cooled to room temperature. The resulting mixture was extracted three times with ethyl acetate. The combined organic layers were dried over Na_2SO_4 and concentrated under vacuum. The target compound **7** 4-Methoxybiphenyl[3] was isolated (1.77 g, 96%) as a white solid by column chromatography (eluting with petroleum ether). ^1H NMR (400 MHz, $CDCl_3$) δ: 7.63-7.55 (m, 4 H), 7.50-7.42 (m, 2 H), 7.41-7.31 (m, 1 H), 7.06-6.98 (m, 2 H), 3.89 (s, 3 H) ppm. ^{13}C NMR (100 MHz, $CDCl_3$) δ: 159.2, 140.8, 133.8, 128.7, 128.2, 126.7, 126.7, 114.2, 55.4 ppm.

5. Synthesis and characterization of substrates

4-Bromo-4'-chlorodiphenyl ether[4]

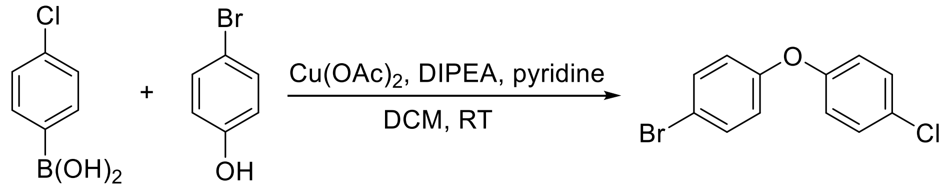

A 100 mL round bottom flask equipped with a stir bar was charged with 4-chlorophenylboronic acid (2.34 g, 15.0 mmol), 4-bromophenol (1.73 g, 10.0 mmol), copper (II) acetate (1.82 g, 10.0 mmol), diisopropylethylamine (DIPEA, 8.3 mL, 50.0 mmol), pyridine (4.0 mL, 50.0 mmol) and 20 mL DCM. The mixture was stirred at room temperature under air atmosphere overnight. After reaction, the mixture was concentrated under vacuum, and treated with ethyl acetate. The obtained mixture was washed with 1 M HCl and brine, dried over Na_2SO_4 and concentrated under vacuum. The obtained residue was purified by flash column chromatography over silica gel (eluting with petroleum ether) to give target compound (1.87 g, 66%) as a colorless oil. ^1H NMR (400 MHz, $CDCl_3$) δ: 7.50-7.42 (d, J = 8.0 Hz, 2 H), 7.35-7.29 (d, J = 8.0 Hz, 2 H), 6.99-6.93 (d, J = 8.0 Hz, 2 H), 6.93-6.86 (d, J = 8.0

Hz, 2 H) ppm. ^{13}C NMR (100 MHz, $CDCl_3$) δ: 156.1, 155.4, 132.8, 129.9, 120.5, 120.2, 120.1, 116.1 ppm.

N-(2-bromophenyl)-2-chloronicotinamide[5]

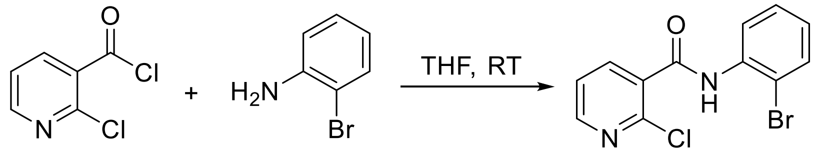

A 50 mL round bottom flask equipped with a stir bar was charged with 2-bromoaniline (0.86 g, 5 mmol) and 10 mL THF. 2-Chloronicotinyl chloride (0.97 g, 5.5 mmol) was added into the flask, and the mixture was stirred at room temperature for 24 h. After reaction, the mixture was concentrated under vacuum, and treated with ethyl acetate. The obtained mixture was washed with 5% $NaHCO_3$ and brine, dried over Na_2SO_4 and concentrated under vacuum. The obtained residue was purified by flash column chromatography over silica gel (eluting with 0-20% ethyl acetate in petroleum ether) to give target compound (1.53 g, 98%) as a white solid. ^1H NMR (400 MHz, $CDCl_3$) δ: 8.74 (br, 1H), 8.57 (dd, J = 4.8, 2.0 Hz, 1H), 8.55-8.48 (m, 1H), 8.27 (dd, J = 7.6, 2.0 Hz, 1H), 7.63 (dd, J = 8.0, 1.6 Hz, 1H), 7.50-7.37 (m, 2H), 7.13-7.05 (m, 1H) ppm. ^{13}C NMR (100 MHz, $CDCl_3$) δ: 162.6, 151.6, 147.1, 140.3, 135.4, 132.6, 131.1, 128.5, 126.1, 123.0, 122.3, 114.0 ppm.

General procedure for the preparation of amide and ester substrates

A 100 mL round bottom flask equipped with a stir bar was charged with carboxyl acid compound (6.0 mmol), amine or alcohol (5.0 mmol), DMAP (10.0 mmol), 1-(3-dimethylaminopropyl)-3-ethylcarbodiimide hydrochloride (EDC•HCl, 20.0 mmol) and 20 mL anhydrous DMF. The mixture was stirred at room temperature for 12 h. After reaction, the mixture was treated with water and DCM. The organic layer was washed with 1 M HCl and brine, dried over Na_2SO_4 and concentrated under vacuum. The collected residue was purified by column chromatography (eluting with 0-20% ethyl acetate in petroleum ether) to give target compound.

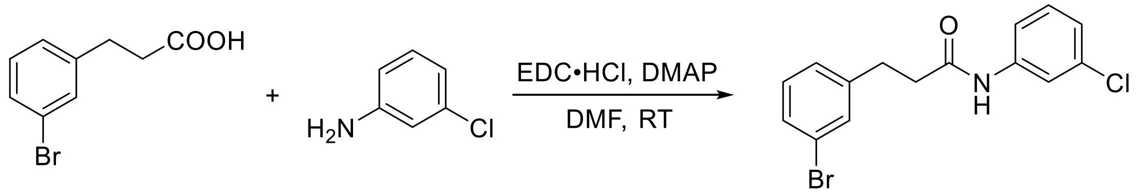

3-Bromo-N-(3-chlorophenyl)-benzenepropanamide The target compound was isolated (1.56 g, 92%) as a white solid, m.p. = 101-102 °C. ^{1}H NMR (400 MHz, $CDCl_3$) δ: 7.64-7.57 (m, 1 H), 7.45-7.33 (m, 2 H), 7.30 (d, *J* = 8.0 Hz, 1 H), 7.27-7.14 (m, 4 H), 7.13-7.04 (m, 1 H), 3.03 (t, *J* = 7.6 Hz, 2 H), 2.66 (t, *J* = 7.6 Hz, 2 H) ppm. ^{13}C NMR (100 MHz, $CDCl_3$) δ: 170.0, 142.8, 138.7, 134.7, 131.4, 130.2, 130.0, 129.6, 127.1, 124.5, 122.6, 120.1, 117.9, 39.0, 30.9 ppm. HRMS (ESI): m/z calculated for $C_{15}H_{14}BrClNO^+$ [M+H$^+$]: 337.9942, found: 337.9932, 339.9904, 341.9872 (isotope abundance ratio = 3:4:1).

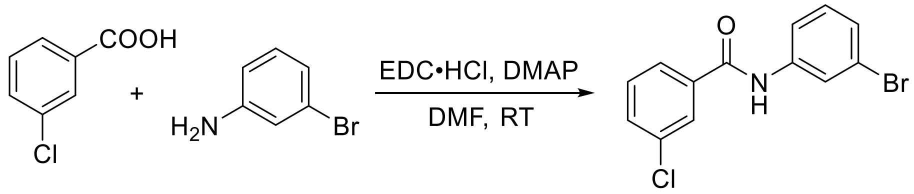

***N*-(3-Bromophenyl)-3-chloro-benzamide** The target compound was isolated (1.40 g, 90%) as a white solid, m.p. = 119-120 °C. ^{1}H NMR (400 MHz, $CDCl_3$) δ: 8.04-7.97 (m, 1 H), 7.88 (br, 1 H), 7.82-7.76 (m, 2 H), 7.71 (dd, *J* = 8.0, 2.0 Hz, 1 H), 7.50 (dd, *J* = 8.0, 2.0 Hz, 1 H), 7.38 (t, *J* = 8.0 Hz, 1 H), 7.31 (t, *J* = 8.0 Hz, 1 H), 7.17 (dd, *J* = 8.0, 2.0 Hz, 1 H) ppm. ^{13}C NMR (100 MHz, $CDCl_3$) δ: 164.3, 138.7, 136.5, 135.1, 134.8, 130.4, 130.2, 130.1, 125.6, 125.0, 123.0, 120.4, 118.2 ppm. HRMS (ESI): m/z calculated for $C_{13}H_{10}BrClNO^+$ [M+H$^+$]: 309.9629, found: 309.9617, 311.9591, 313.9560 (isotope abundance ratio = 3:4:1).

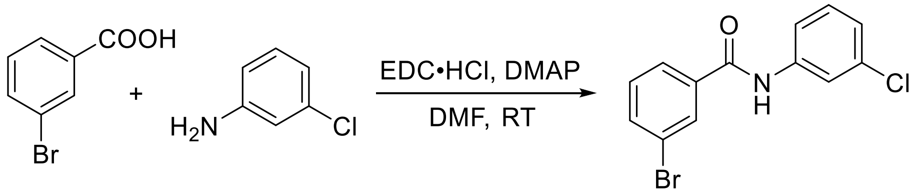

3-Bromo-*N*-(3-chlorophenyl)-benzamide The target compound was isolated (1.37 g, 88%) as a white solid, m.p. = 138-140 °C. ^{1}H NMR (400 MHz, $CDCl_3$) δ: 8.04 (br, 1 H), 8.00-7.96 (m, 1 H), 7.80-7.74 (m, 2 H), 7.68 (dd, *J* = 8.0, 2.0 Hz, 1 H), 7.49 (dd, *J* = 8.0, 2.0 Hz, 1 H), 7.39-7.29 (m, 2 H), 7.15 (dd, *J* = 8.0, 2.0 Hz, 1 H) ppm. ^{13}C NMR (100 MHz, $CDCl_3$) δ: 164.5, 138.7, 136.4, 135.0, 134.8, 130.4, 130.3, 130.1, 125.7, 125.0, 123.0, 120.5, 118.3 ppm. HRMS (ESI): m/z calculated for $C_{13}H_{10}BrClNO^+$ [M+H$^+$]: 309.9629, found: 309.9619, 311.9592, 313.9561 (isotope abundance ratio = 3:4:1).

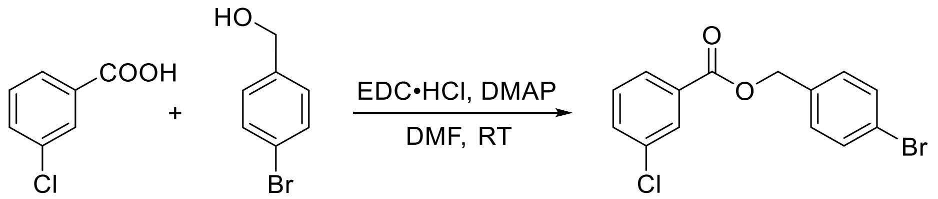

(4-Bromophenyl)methyl-3-chloro-benzoic ester The target compound was isolated (1.56 g, 96%) as a white solid, m.p. = 50-51 °C. ^{1}H NMR (400 MHz, $CDCl_3$) δ: 8.08-8.03 (m, 1 H), 7.96 (d, *J* = 8.0 Hz, 1 H), 7.60-7.51 (m, 3 H), 7.41 (t, *J* = 8.0 Hz, 1 H), 7.37-7.32 (m, 2 H), 5.33 (s, 2 H) ppm. ^{13}C NMR (100 MHz, $CDCl_3$) δ: 165.1, 134.7, 134.6, 133.2, 131.8, 131.6, 130.0, 129.8, 129.7, 127.8, 122.5, 66.3 ppm. HRMS (ESI): m/z calculated for $C_{14}H_{11}BrClO_2^+$ [M+H$^+$]: 324.9626, found: 324.9623, 326.9596, 328.9565 (isotope abundance ratio = 3:4:1).

General procedure for the preparation of sulfonamide substrates

A 50 mL round bottom flask equipped with a stir bar was charged with aryl sulfonyl chloride (7.5 mmol), aniline (5.0 mmol), pyridine (0.6 mL, 7.5 mmol) and 10 mL DCM. The mixture was stirred at room temperature overnight. After reaction, the mixture was treated with water and DCM. The separated organic phase was washed with 1 M HCl and brine, dried over Na_2SO_4 and concentrated under vacuum. The residue was purified by column chromatography to give target compound (eluting with 0-20% ethyl acetate in petroleum ether).

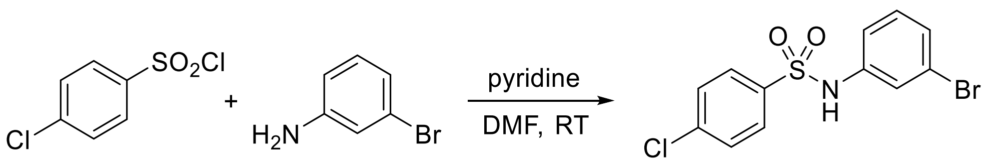

***N*-(3-Bromophenyl)-4-chlorobenzenesulfonamide** The target compound was isolated (1.71 g, 99%) as a white solid, m.p. = 101-102 °C. ^{1}H NMR (400 MHz, $CDCl_3$) δ: 7.80-7.74 (m, 2 H), 7.51-7.44 (m, 2 H), 7.31-7.26 (m, 2 H), 7.17-7.10 (m, 2 H), 7.05 (dd, *J* = 8.0, 2.0 Hz, 1 H) ppm. ^{13}C NMR (100 MHz, $CDCl_3$) δ: 140.0, 137.4, 137.1, 130.8, 129.6, 128.7, 128.7, 124.2, 123.0, 119.8 ppm. HRMS (ESI): m/z calculated for $C_{12}H_9BrClNNaO_2S^+$ [M+Na$^+$]: 367.9118, found: 367.9105, 369.9078, 371.9047 (isotope abundance ratio = 3:4:1).

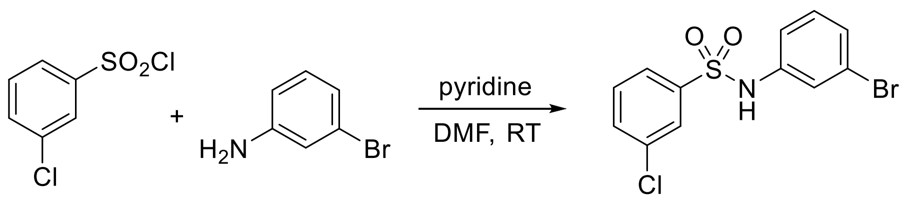

***N*-(3-Bromophenyl)-3-chlorobenzenesulfonamide** The target compound was isolated (1.70 g, 98%) as a white solid, m.p. = 100-102 °C. ^{1}H NMR (400 MHz, $CDCl_3$) δ: 7.79-7.74 (m, 1 H), 7.69 (dd, *J* = 7.8, 2.0 Hz, 1 H), 7.61 (dd, *J* = 8.0, 2.0 Hz, 1 H), 7.50 (t, *J* = 8.0 Hz, 1 H), 7.32-7.28 (m, 1 H), 7.24 (dd, *J* = 7.8, 2.0 Hz, 1 H), 7.17 (t, *J* = 8.0 Hz, 1 H), 7.09 (dd, *J* = 8.0, 2.0 Hz, 1 H) ppm. ^{13}C NMR (100 MHz, $CDCl_3$) δ: 141.2, 138.8, 134.8, 132.7, 130.4, 127.4, 126.6, 125.7, 125.1, 123.3, 122.2, 119.2 ppm. HRMS (ESI): m/z calculated for $C_{12}H_{10}BrClNO_2S^+$ [M+H$^+$]: 345.9299, found: 345.9289, 347.9262, 349.9231 (isotope abundance ratio = 3:4:1).

6. General procedure and characterization results for Scheme 3, 4, 5

6.1. General procedure for Scheme 3, 4, 5

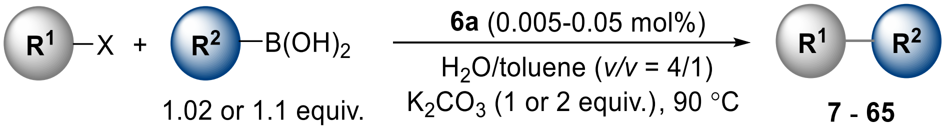

A 10 mL Schlenk tube equipped with a stir bar was charged with boronic acid (0.51 or 0.55 mmol, 1.02 or 1.1 eq.), aryl halides (0.5 mmol), K_2CO_3 (69.0 mg, 0.5 mmol), pre-catalyst **6a** (20-200 μL solution of **6a** in Table S1, entry 1), 0.2 mL toluene and 0.8 mL deionized H_2O under N_2 atmosphere. The mixture was stirred at 90 °C for corresponding reaction time. After reaction, the tube was cooled to room temperature. The resulting mixture was extracted three times with ethyl acetate. The combined organic layers were dried over Na_2SO_4 and concentrated under vacuum. The collected residue was purified by column chromatography using silica gel to give target compound. The product was characterized by ^{1}H, ^{13}C and ^{19}F NMR measurements. Unknown compounds were further confirmed with HRMS.

6.2. Characterization results for compounds in Scheme 3, 4, 5

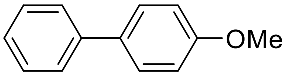

4-Methoxybiphenyl (7)[3] Pre-catalyst **6a** (50 ppm) was used. The target compound was isolated (91.2 mg, 99%) as a white solid by column chromatography (eluting with petroleum ether). ^{1}H NMR (400 MHz, $CDCl_3$) δ: 7.63-7.55 (m, 4 H), 7.50-7.42 (m, 2 H), 7.41-7.31 (m, 1 H), 7.06-6.98 (m, 2 H), 3.89 (s, 3 H) ppm. ^{13}C NMR (100 MHz, $CDCl_3$) δ: 159.2, 140.8, 133.8, 128.7, 128.2, 126.7, 126.7, 114.2, 55.4 ppm.

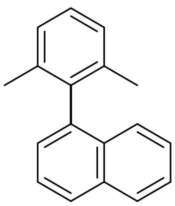

1-(2,6-Dimethylphenyl)naphthalene (8)[6] Pre-catalyst **6a** (50 ppm) was used. The target compound was isolated (110.3 mg, 95%) as a white solid by column chromatography (eluting with petroleum ether). ^{1}H NMR (400 MHz, $CDCl_3$) δ: 7.97-7.86 (m, 2 H), 7.62-7.55 (m, 1 H), 7.53-7.49 (m, 1 H), 7.41-7.36 (m, 2 H), 7.33-7.26 (m, 2 H), 7.21 (d, *J* = 7.6 Hz, 2 H), 1.94 (s, 6 H) ppm. ^{13}C NMR (100 MHz, $CDCl_3$) δ: 139.6, 138.7, 137.0, 133.7, 131.7, 128.3, 127.3, 127.3, 127.2, 126.4, 126.1, 125.8, 125.7, 125.4, 20.4 ppm.

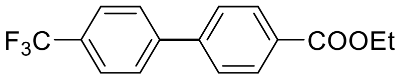

Ethyl 4-(4-trifluoromethylphenyl)benzoate (9)[7] Pre-catalyst **6a** (50 ppm) was used. The target compound was isolated (141.3 mg, 96%) as a white solid by column chromatography (eluting with 0-5% ethyl acetate in petroleum ether). ^{1}H NMR (400 MHz, $CDCl_3$) δ: 8.21-8.14 (m, 2 H), 7.72-7.62 (m, 4 H), 7.72-7.66 (m, 2 H), 4.44 (q, *J* = 7.2 Hz, 2 H), 1.45 (t, *J* = 7.2 Hz, 3 H) ppm. ^{13}C NMR (100 MHz, $CDCl_3$) δ: 166.3, 144.0, 143.6, 134.9, 130.2, 130.0 (q, 32.0 Hz), 127.6, 127.2, 125.8 (q, *J* = 4.0 Hz), 124.3 (q, *J* = 272.4 Hz), 61.2, 14.4 ppm. ^{19}F NMR (376 MHz, $CDCl_3$) δ: -62.56 ppm.

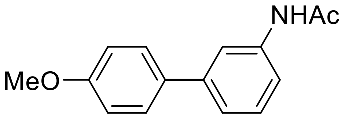

4'-(1,1-Dimethylethyl)[1,1'-Biphenyl]-4-methanol (10)[8] Pre-catalyst **6a** (200 ppm) was used. The target compound was isolated (110.9 mg, 92%) as an off white solid by column chromatography (eluting with 0-50% ethyl acetate in petroleum ether). ^{1}H NMR (400 MHz, $CDCl_3$) δ: 7.77-7.67 (m, 1 H), 7.57-7.51 (m, 2 H), 7.48 (d, *J* = 8.0 Hz, 1 H), 7.42-7.30 (m, 3 H), 6.98 (d, *J* = 8.8 Hz, 2 H), 3.87 (s, 3 H), 2.22 (s, 3 H) ppm. ^{13}C NMR (100 MHz, $CDCl_3$) δ: 168.4, 159.3, 141.7, 138.2, 134.3, 129.4, 128.2, 127.2, 122.7, 118.2, 114.2, 55.4, 24.7 ppm.

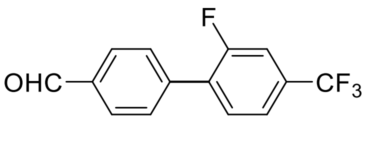

2'-Fluoro-4'-(trifluoromethyl)[1,1'-biphenyl]-4-carboxaldehyde (11) Pre-catalyst **6a** (200 ppm) was used. The target compound was isolated (120.7 mg,

90%) as a colorless oil by column chromatography (eluting with 0-10% ethyl acetate in petroleum ether). ^{1}H NMR (400 MHz, $CDCl_3$) δ: 10.11 (s, 1 H), 8.06-7.98 (m, 2 H), 7.78-7.66 (m, 4 H), 7.43-7.35 (m, 1 H) ppm. ^{13}C NMR (100 MHz, $CDCl_3$) δ: 191.7, 155.5 (d, *J* = 257.0 Hz), 143.3, 140.2, 135.9, 134.5 (d, *J* = 3.0 Hz), 129.9, 129.6 (d, *J* = 28.0 Hz), 129.7 (d, *J* = 3.0 Hz), 128.6 (q, *J* = 34.0 Hz), 127.2 (q, *J* = 4.0 Hz), 124.3 (q, *J* = 271.0 Hz) ppm. ^{19}F NMR (376 MHz, $CDCl_3$) δ: -61.36, -119.21 ppm. HRMS (ESI): m/z calculated for $C_{14}H_9F_4O^+$ [M+H$^+$]: 269.0584, found: 269.0574.

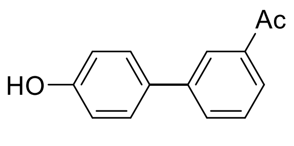

1-(4'-Hydroxy-1,1'-biphenyl-3-yl)ethenone (12)[9] Pre-catalyst **6a** (500 ppm) was used. The target compound was isolated (97.6 mg, 92%) as an off white solid by column chromatography (eluting with 0-20% ethyl acetate in petroleum ether). ^{1}H NMR (400 MHz, $CDCl_3$) δ: 8.22-8.13 (m, 1 H), 7.92 (d, *J* = 8.0 Hz, 1 H), 7.77 (d, *J* = 8.0 Hz, 1 H), 7.58-7.48 (m, 3 H), 6.98 (d, *J* = 8.0 Hz, 2 H), 5.55 (br, 1 H), 2.69 (s, 3 H) ppm. ^{13}C NMR (100 MHz, $CDCl_3$) δ: 198.8, 155.8, 141.3, 137.5, 132.7, 131.4, 129.0, 128.5, 126.7, 126.5, 115.9, 26.9 ppm.

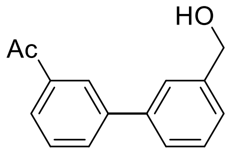

1-[3'-(Hydroxymethyl)[1,1'-biphenyl]-3-yl]ethanone (13) Pre-catalyst **6a** (50 ppm) was used. The target compound was isolated (108.6 mg, 96%) as a colorless oil by column chromatography (eluting with 0-50% ethyl acetate in petroleum ether). ^{1}H NMR (400 MHz, $CDCl_3$) δ: 8.24-8.15 (m, 1 H), 7.95 (d, *J* = 8.0 Hz, 1 H), 7.81 (dd, *J* = 8.0 Hz, 1 H), 7.68-7.62 (m, 1 H), 7.55 (t, *J* = 8.0 Hz, 2 H), 7.48 (t, *J* = 7.6 Hz, 1 H), 7.44-7.38 (m, 1 H), 4.80 (s, 2 H), 2.68 (s, 3 H), 2.03 (br, 1 H) ppm. ^{13}C NMR (100 MHz, $CDCl_3$) δ: 198.3, 141.6, 141.5, 140.4, 137.6, 131.8, 129.2, 129.1, 127.4, 126.9, 126.5, 126.4, 125.8, 65.2, 26.8 ppm. HRMS (ESI): m/z calculated for $C_{15}H_{15}O_2^+$ [M+H$^+$]: 227.1067, found: 227.1060.

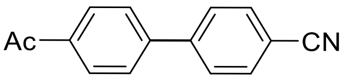

4-Acetyl-4'-cyanobiphenyl (14)[10] Pre-catalyst **6a** (200 ppm) was used. The target compound was isolated

(102.8 mg, 93%) as a white solid by column chromatography (eluting with 0-10% ethyl acetate in petroleum ether). ^{1}H NMR (400 MHz, $CDCl_3$) δ: 8.13-8.06 (m, 2 H), 7.83-7.68 (m, 6 H), 2.68 (s, 3 H) ppm. ^{13}C NMR (100 MHz, $CDCl_3$) δ: 197.5, 144.3, 143.5, 136.9, 132.8, 129.1, 127.9, 127.5, 118.7, 111.9, 26.8 ppm.

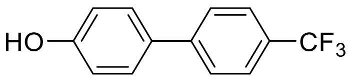

4-(4'-Trifluoromethylphenyl)phenol (15)[11] Pre-catalyst **6a** (500 ppm) was used. The target compound was isolated (107.2 mg, 90%) as a white solid by column chromatography (eluting with 0-20% ethyl acetate in petroleum ether). ^{1}H NMR (400 MHz, $CDCl_3$) δ: 7.75-7.61 (m, 4 H), 7.58-7.47 (m, 2 H), 7.01-6.92 (m, 2 H), 5.00 (br, 1 H) ppm. ^{13}C NMR (100 MHz, $CDCl_3$) δ: 155.8, 144.2, 132.5, 129.1, 128.6 (q, J = 32.0 Hz), 126.9, 125.7 (q, J = 4.0 Hz), 124.3 (q, J = 272.0 Hz), 115.9 ppm. ^{19}F NMR (376 MHz, $CDCl_3$) δ: -62.35 ppm.

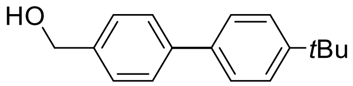

4'-(1,1-Dimethylethyl)[1,1'-Biphenyl]-4-methanol (16) Pre-catalyst **6a** (50 ppm) was used. The target compound was isolated (116.5 mg, 97%) as a colorless oil by column chromatography (eluting with 0-20% ethyl acetate in petroleum ether). ^{1}H NMR (400 MHz, $CDCl_3$) δ: 7.66-7.55 (m, 4 H), 7.54-7.42 (m, 4 H), 4.77 (s, 2 H), 1.75 (br, 1 H), 1.40 (s, 9 H) ppm. ^{13}C NMR (100 MHz, $CDCl_3$) δ: 150.4, 140.5, 139.6, 137.9, 127.5, 127.2, 126.7, 125.8, 65.2, 34.6, 31.4 ppm. HRMS (ESI): m/z calculated for $C_{17}H_{21}O^+$ [M+H$^+$]: 241.1587, found: 241.1583.

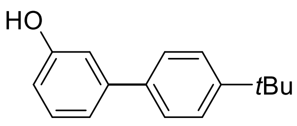

4'-(1,1-Dimethylethyl)[1,1'-biphenyl]-3-ol (17) Pre-catalyst **6a** (500 ppm) was used. The target compound was isolated (109.3 mg, 97%) as a white solid by column chromatography (eluting with 0-20% ethyl acetate in petroleum ether), m.p. = 85-86 °C. ^{1}H NMR (400 MHz, $CDCl_3$) δ: 7.59-7.43 (m, 4 H), 7.32 (t, J = 8.0 Hz, 1 H), 7.19 (d, J = 8.0 Hz, 1 H), 7.12-7.05 (m, 1 H), 6.82 (d, J = 8.0 Hz, 1 H), 4.91 (br, 1 H), 1.39 (s, 9 H) ppm. ^{13}C NMR (100 MHz, $CDCl_3$) δ: 155.8, 150.5, 142.9, 137.8,

129.9, 126.7, 126.5, 125.7, 119.7, 113.9, 34.6, 31.4 ppm. HRMS (ESI): m/z calculated for $C_{16}H_{19}O^+$ [M+H$^+$]: 227.1431, found: 227.1425.

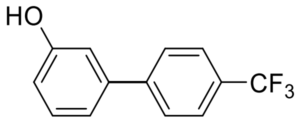

4'-(Trifluoromethyl)[1,1'-biphenyl]-3-ol (18)[12] Pre-catalyst **6a** (500 ppm) was used. The target compound was isolated (114.3 mg, 96%) as a white solid by column chromatography (eluting with 0-20% ethyl acetate in petroleum ether). ^{1}H NMR (400 MHz, $CDCl_3$) δ: 7.78-7.63 (m, 4 H), 7.37 (t, *J* = 8.0 Hz, 1 H), 7.20 (d, *J* = 8.0 Hz, 1 H), 7.11-7.07 (m, 1 H), 6.90 (dd, *J* = 8.0, 2.4 Hz, 1 H), 5.09 (br, 1 H) ppm. ^{13}C NMR (100 MHz, $CDCl_3$) δ: 156.0, 144.2, 141.5, 130.3, 128.6 (q, *J* = 32.0 Hz), 127.4, 125.7 (q, *J* = 4.0 Hz), 124.3 (q, *J* = 272.0 Hz), 119.9, 115.1, 114.2 ppm. ^{19}F NMR (376 MHz, $CDCl_3$) δ: -62.45 ppm.

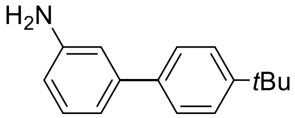

4'-(1,1-Dimethylethyl)[1,1'-Biphenyl]-3-amine (19)[13] Pre-catalyst **6a** (500 ppm) was used. The target compound was isolated (109.0 mg, 97%) as a light yellow solid by column chromatography (eluting with 0-20% ethyl acetate in petroleum ether), m.p. = 91-92 °C. ^{1}H NMR (400 MHz, $CDCl_3$) δ: 7.57-7.52 (m, 2 H), 7.50-7.45 (m, 2 H), 7.27-7.21 (m, 1 H), 7.03 (d, *J* = 7.6 Hz, 1 H), 6.96-6.92 (m, 1 H), 6.69 (dd, *J* = 8.0, 2.4 Hz, 1 H), 3.76 (br, 2H), 1.39 (s, 9 H) ppm. ^{13}C NMR (100 MHz, $CDCl_3$) δ: 150.2, 146.6, 142.3, 138.5, 129.6, 126.7, 125.6, 117.6, 113.9, 113.8, 34.5, 31.4 ppm. HRMS (ESI): m/z calculated for $C_{16}H_{20}N^+$ [M+H$^+$]: 226.1590, found: 226.1585.

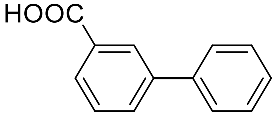

3-Biphenylcarboxylic acid (20)[14] Pre-catalyst **6a** (500 ppm) was used. The target compound was isolated (94.1 mg, 95%) as a white solid by column chromatography (eluting with 0-10% methanol in DCM). ^{1}H NMR (400 MHz, $CDCl_3$) δ: 12.09 (s, 1 H), 8.44-8.38 (m, 1 H), 8.18-8.12 (m, 1 H), 7.88 (m, 1 H), 7.71-7.64 (m, 2 H), 7.59 (m, 1 H), 7.55-7.48 (m, 2 H), 7.46-7.38 (m, 1 H) ppm. ^{13}C NMR (100 MHz, $CDCl_3$) δ: 172.1, 141.7, 139.9, 132.5, 129.8, 129.1, 129.0, 129.0, 128.9, 127.9, 127.2 ppm.

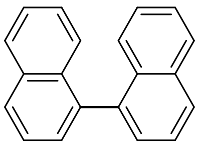

Naphthalene (21)[15] Pre-catalyst **6a** (50 ppm) was used. The target compound was isolated (123.2 mg, 97%) as a white solid by column chromatography (eluting with petroleum ether). ^{1}H NMR (400 MHz, $CDCl_3$) δ: 8.07-7.94 (m, 4 H), 7.63 (t, *J* = 8.0 Hz, 2 H), 7.58-7.47 (m, 4 H), 7.43 (d, *J* = 8.8 Hz, 2 H), 7.32 (m, 2 H) ppm. ^{13}C NMR (100 MHz, $CDCl_3$) δ: 138.5, 133.5, 132.9, 128.1, 127.9, 127.8, 126.6, 126.0, 125.8, 125.4 ppm.

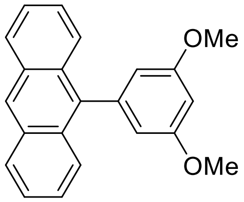

9-(3,5-Dimethoxyphenyl)anthracene (22) Pre-catalyst **6a** (50 ppm) was used. The target compound was isolated (152.3 mg, 97%) as a white solid by column chromatography (eluting with petroleum ether), m.p. = 144-146 °C. ^{1}H NMR (400 MHz, $CDCl_3$) δ: 8.52 (s, 1 H), 8.12-8.04 (m, 2 H), 7.79 (d, *J* = 8.4 Hz, 2 H), 7.54-7.45 (t, *J* = 8.8 Hz,, 2 H), 7.41 (t, *J* = 8.8 Hz, 2 H), 6.72-6.60 (m, 3 H), 3.86 (s, 6 H) ppm. ^{13}C NMR (100 MHz, $CDCl_3$) δ: 160.7, 140.9, 136.9, 131.3, 129.9, 128.3, 126.9, 126.6, 125.4, 125.2, 109.2, 99.8, 55.5 ppm. HRMS (ESI): m/z calculated for $C_{22}H_{19}O_2^+$ [M^+]: 315.1380, found: 315.1379.

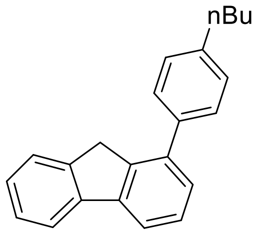

1-(4-Butylphenyl)fluorene (23) Pre-catalyst **6a** (50 ppm) was used. The target compound was isolated (134.3 mg, 90%) as a white solid by column chromatography (eluting with petroleum ether), m.p. = 185-187 °C. ^{1}H NMR (400 MHz, $CDCl_3$) δ: 7.91-7.77 (m, 3 H), 7.68-7.56 (m, 4 H), 7.42 (t, *J* = 6.8 Hz, 1 H), 7.37-7.29 (m, 3 H), 3.99 (s, 2 H), 2.76-2.63 (m, 2 H), 1.74-1.62 (m, 2 H), 1.48-1.39 (m, 2 H), 0.99 (t, *J* = 7.2 Hz, 3 H) ppm. ^{13}C NMR (100 MHz, $CDCl_3$) δ: 143.9, 143.4, 142.0, 141.5, 140.6, 139.8, 138.8, 128.9, 127.0, 126.8, 126.6, 125.8, 125.0, 123.6, 120.1, 119.9, 37.0, 35.3, 33.7, 22.5, 14.0 ppm. HRMS (ESI): m/z calculated for $C_{23}H_{22}^+$ [M^+]: 298.1722, found: 298.1713.

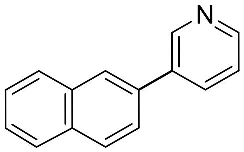

3-(2-Naphthyl)pyridine (24)[16] Pre-catalyst **6a** (50 ppm) was used. The target compound was isolated (98.5 mg, 96%) as a white solid by column chromatography (eluting with 0-20%

ethyl acetate in petroleum ether). ^{1}H NMR (400 MHz, $CDCl_3$) δ: 9.03-8.99 (m, 1 H), 8.69-8.61 (m, 1 H), 8.11-8.06 (m, 1 H), 8.05-7.89 (m, 4 H), 7.74 (dd, *J* = 8.4, 2.0 Hz, 1 H), 7.60-7.51 (m, 2 H), 7.44 (dd, *J* = 8.0, 4.8 Hz, 1 H) ppm. ^{13}C NMR (100 MHz, $CDCl_3$) δ: 148.6, 148.5, 136.6, 135.2, 134.6, 133.6, 132.9, 128.9, 128.2, 127.7, 126.6, 126.5, 126.2, 125.0, 123.6 ppm.

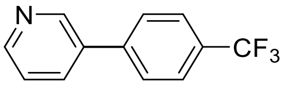

3-(4-Trifluoromethylphenyl)pyridine (25)[17] Pre-catalyst **6a** (50 ppm) was used. The target compound was isolated (108.5 mg, 97%) as a white solid by column chromatography (eluting with 0-20% ethyl acetate in petroleum ether). ^{1}H NMR (400 MHz, $CDCl_3$) δ: 8.95-8.84 (m, 1 H), 8.73-8.62 (m, 1 H), 7.92 (d, *J* = 8.0 Hz, 1 H), 7.81-7.74 (m, 2 H), 7.74-7.69 (m, 2 H), 7.44 (dd, *J* = 8.0, 4.8 Hz, 1 H) ppm. ^{13}C NMR (100 MHz, $CDCl_3$) δ: 149.3, 148.2, 141.3, 135.4, 134.6, 128.8 (q, *J* = 32.0 Hz), 127.5, 126.0 (q, *J* = 5.0 Hz), 124.5 (q, *J* = 272.0 Hz), 123.7 ppm. ^{19}F NMR (376 MHz, $CDCl_3$) δ: -62.88 ppm.

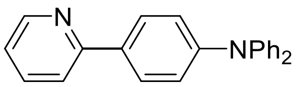

***N,N*-Diphenyl-4-(2-pyridinyl)benzenamine (26)**[18] Pre-catalyst **6a** (50 ppm) was used. The target compound was isolated (151.3 mg, 94%) as a light yellow solid by column chromatography (eluting with 0-5% ethyl acetate in petroleum ether). ^{1}H NMR (400 MHz, $CDCl_3$) δ: 8.72-8.64 (m, 1 H), 7.94-7.85 (m, 2 H), 7.78-7.66 (m, 2 H), 7.34-7.29 (m, 3 H), 7.28 (s, 1 H), 7.22-7.15 (m, 7 H), 7.12-7.03 (m, 2 H) ppm. ^{13}C NMR (100 MHz, $CDCl_3$) δ: 157.0, 149.5, 148.7, 147.5, 136.7, 133.0, 129.3, 127.7, 124.7, 123.2, 123.2, 121.5, 119.9 ppm.

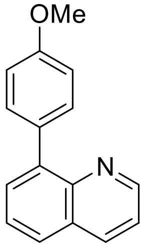

8-(4-Methoxyphenyl)quinoline (27)[19] Pre-catalyst **6a** (50 ppm) was used. The target compound was isolated (113.9 mg, 97%) as a light yellow solid by column chromatography (eluting with 0-10% ethyl acetate in petroleum ether). ^{1}H NMR (400 MHz, $CDCl_3$) δ: 9.02-8.95 (m, 1 H), 8.22 (dd, *J* = 8.4, 2.0 Hz, 1 H), 7.82 (d, *J* = 8.0 Hz, 1 H), 7.75 (dd, *J* = 7.2, 1.6 Hz, 1 H), 7.72-7.71-7.67 (m, 2 H), 7.64-7.60 (m Hz, 1 H), 7.47-7.39 (m, 1 H), 7.12-7.04 (m,

2 H), 3.91 (s, 3 H) ppm. ^{13}C NMR (100 MHz, $CDCl_3$) δ: 159.1, 150.2, 146.1, 140.5, 136.3, 131.9, 131.7, 130.0, 128.8, 127.1, 126.3, 120.9, 113.6, 55.3 ppm.

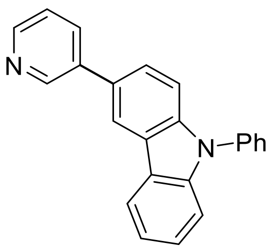

9-Phenyl-3-(3-pyridinyl)-9*H*-carbazole (28) Pre-catalyst **6a** (50 ppm) was used. The target compound was isolated (155.3 mg, 97%) as a yellow oil by column chromatography (eluting with 0-20% ethyl acetate in petroleum ether). ^{1}H NMR (400 MHz, $CDCl_3$) δ: 9.06-8.98 (m, 1 H), 8.68-8.58 (m, 1 H), 8.40-8.36 (m, 1 H), 8.25-8.22 (m, 1 H), 8.04-8.01 (m, 1 H), 7.70-7.58 (m, 5 H), 7.56-7.34 (m, 6 H) ppm. ^{13}C NMR (100 MHz, $CDCl_3$) δ: 148.5, 147.7, 141.4, 140.7, 137.4, 134.5, 130.0, 129.8, 127.7, 127.1, 126.4, 125.2, 124.2, 124.1, 123.6, 123.2, 120.4, 120.3, 119.0, 110.4, 110.1 ppm. HRMS (ESI): m/z calculated for $C_{23}H_{16}N_2^+$ [M$^+$]: 320.1313, found: 320.1312.

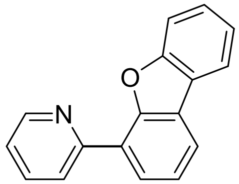

4-(2-Pyridyl)dibenzofuran (29)[20] Pre-catalyst **6a** (50 ppm) was used. The target compound was isolated (116.5 mg, 95%) as a white solid by column chromatography (eluting with 0-10% ethyl acetate in petroleum ether). ^{1}H NMR (400 MHz, $CDCl_3$) δ: 8.82 (d, *J* = 4.8, 1 H), 8.48-8.41 (m, 1 H), 8.32-8.30 (m, 1 H), 8.09-8.01 (m, 2 H), 7.91 (m, 1 H), 7.67 (d, *J* = 8.0 Hz, 1 H), 7.52 (t, *J* = 8.4 Hz, 2 H), 7.41 (t, *J* = 7.6 Hz, 1 H), 7.33 (dd, *J* = 7.6, 4.8 Hz, 1 H) ppm. ^{13}C NMR (100 MHz, $CDCl_3$) δ: 156.1, 153.8, 153.7, 149.8, 136.7, 127.3, 127.2, 125.2, 124.3, 124.2, 124.0, 123.3, 123.0, 122.5, 121.3, 120.7, 111.8 ppm.

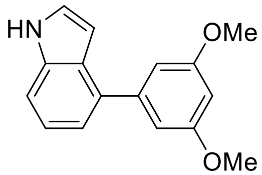

4-(3,5-Dimethoxyphenyl)indole (30) Pre-catalyst **6a** (50 ppm) was used. The target compound was isolated (117.8 mg, 93%) as a light yellow oil by column chromatography (eluting with 0-10% ethyl acetate in petroleum ether). ^{1}H NMR (400 MHz, $CDCl_3$) δ: 8.41-8.18 (br, 1 H), 7.43 (d, *J* = 8.0 Hz, 1 H), 7.33-7.29 (m, 1 H), 7.26 -7.20 (m, 2 H), 6.98-6.87 (m, 2 H), 6.84-6.73 (m, 1 H), 6.59-6.50 (m, 1 H), 3.89 (s, 6 H) ppm. ^{13}C NMR (100 MHz, $CDCl_3$) δ: 160.8, 143.4, 136.2, 134.4, 126.1,

124.5, 122.2, 119.6, 110.5, 106.9, 102.3, 99.3, 55.5 ppm. HRMS (ESI): m/z calculated for $C_{16}H_{16}NO_2^+$ [M+H$^+$]: 254.1176, found: 254.1171.

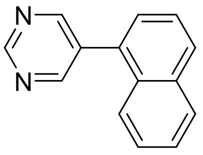

5-(Naphthalen-1-yl)pyrimidine (31)[21] Pre-catalyst **6a** (200 ppm) was used. The target compound was isolated (99.9 mg, 97%) as a white solid by column chromatography (eluting with 0-40% ethyl acetate in petroleum ether). ^{1}H NMR (400 MHz, $CDCl_3$) δ: 9.35 (s, 1 H), 8.93 (s, 2 H), 8.02-7.95 (m, 2 H), 7.76 (d, *J* = 8.0 Hz, 1 H), 7.67-7.48 (m, 3 H), 7.44 (d, *J* = 8.0 Hz, 1 H) ppm. ^{13}C NMR (100 MHz, $CDCl_3$) δ: 157.3, 134.9, 134.4, 133.8, 132.3, 131.1, 129.5, 128.7, 127.8, 127.1, 126.5, 125.5, 124.5 ppm.

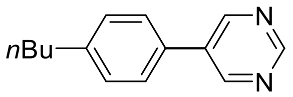

5-(4-Butylphenyl)pyrimidine (32) Pre-catalyst **6a** (200 ppm) was used. The target compound was isolated (100.8 mg, 95%) as a colorless oil by column chromatography (eluting with 0-20% ethyl acetate in petroleum ether). ^{1}H NMR (400 MHz, $CDCl_3$) δ: 9.20 (s, 1 H), 8.96 (s, 2 H), 7.53 (d, *J* = 8.0 Hz, 2 H), 7.36 (d, *J* = 8.0 Hz, 2 H), 2.70 (t, *J* = 8.0 Hz, 2 H), 1.73-1.59 (m, 2 H), 1.43-1.35 (m, 2 H), 0.99-0.94 (t, *J* = 8.0 Hz, 3 H) ppm. ^{13}C NMR (100 MHz, $CDCl_3$) δ: 157.2, 154.8, 144.1, 134.3, 131.5, 129.5, 126.8, 35.4, 33.6, 22.4, 14.0 ppm. HRMS (ESI): m/z calculated for $C_{14}H_{17}N_2^+$ [M+H$^+$]: 213.1386, found: 213.1386.

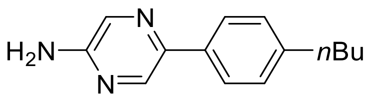

5-(4-Butylphenyl)pyrazin-2-amine (33) Pre-catalyst **6a** (500 ppm) was used. The target compound was isolated (108.9 mg, 96%) as a white solid by column chromatography (eluting with 0-50% ethyl acetate in petroleum ether), m.p. = 119-121 °C. ^{1}H NMR (400 MHz, $CDCl_3$) δ: 8.46 (s, 1 H), 8.08 (s 1 H), 7.89-7.75 (m, 2 H), 7.31-7.27 (m, 2 H), 4.63 (br, 2 H), 2.75-2.61 (m, 2 H), 1.71-1.58 (m, 2 H), 1.45 - 1.35 (m, 2 H), 0.96 (t, *J* = 7.2 Hz, 3 H) ppm. ^{13}C NMR (100 MHz, $CDCl_3$) δ: 166.2, 152.9, 143.2, 138.9, 134.4, 131.5, 129.0, 125.6, 35.4, 33.6, 22.4, 14.0 ppm. HRMS (ESI): m/z calculated for $C_{14}H_{18}N_3^+$ [M+H$^+$]: 228.1495, found: 228.1496.

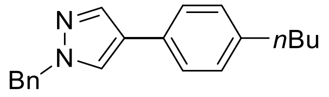

1-Benzyl-4-(4-butylphenyl)-1H-pyrazole (34) Pre-catalyst **6a** (200 ppm) was used. The target compound was isolated (137.8 mg, 95%) as a white solid by column chromatography (eluting with 0-10% ethyl acetate in petroleum ether), m.p. = 88-90 °C. ^{1}H NMR (400 MHz, $CDCl_3$) δ: 7.83 (s, 1 H), 7.61 (s, 1 H), 7.43-7.24 (m, 7 H), 7.22-7.16 (m, 2 H), 5.36 (s, 2 H), 2.68-2.59 (m, 2 H), 1.68-1.59 (m, 2 H), 1.45 - 1.34 (m, 2 H), 0.95 (t, *J* = 7.2 Hz, 3 H) ppm. ^{13}C NMR (100 MHz, $CDCl_3$) δ: 169.2, 163.1, 141.2, 136.9, 129.8, 129.4, 128.9, 128.1, 127.7, 126.0, 125.4, 56.2, 35.3, 33.7, 22.3, 14.0 ppm. HRMS (ESI): m/z calculated for $C_{20}H_{23}N_2^+$ [M+H$^+$): 291.1856, found: 291.1855.

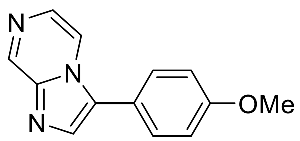

3-(4-Methoxyphenyl)imidazo[1,2-a]pyrazine (35) Pre-catalyst **6a** (500 ppm) was used. The target compound was isolated (104.6 mg, 93%) as a white solid by column chromatography (eluting with 0-5% methanol in DCM), m.p. = 132-134 °C. ^{1}H NMR (400 MHz, $CDCl_3$) δ: 9.15 (d, *J* = 8.0 Hz, 1 H), 8.28-8.17 (m, 1 H), 7.95-7.79 (m, 2 H), 7.58-7.45 (m, 2 H), 7.17-7.05 (m, 2 H), 3.91 (s, 3 H) ppm. ^{13}C NMR (100 MHz, $CDCl_3$) δ: 186.4, 160.2, 144.5, 134.1, 129.8, 129.5, 127.6, 120.0, 116.2, 114.9, 55.5 ppm. HRMS (ESI): m/z calculated for $C_{13}H_{12}N_3O^+$ [M+H$^+$]: 226.0975, found: 226.0875.

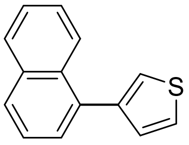

3-(1-Naphthyl)thiophene (36)[22] Pre-catalyst **6a** (50 ppm) was used. The target compound was isolated (99.8 mg, 95%) as a white solid by column chromatography (eluting with petroleum ether). ^{1}H NMR (400 MHz, $CDCl_3$) δ: 8.10 (d, *J* =8.0 Hz, 1 H), 7.97-7.93 (m, 1 H), 7.93-7.87 (m, 1 H), 7.58-7.48 (m, 5 H), 7.47-7.42 (m, 1 H), 7.38-7.34 (m, 1 H) ppm. ^{13}C NMR (100 MHz, $CDCl_3$) δ: 142.0, 141.2, 135.0, 133.8, 131.9, 129.7, 128.4, 127.8, 127.0, 126.2, 125.9, 125.4, 125.3, 123.5 ppm.

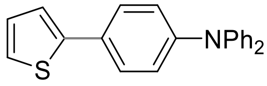

***N,N*-Diphenyl-4-(2-thienyl)benzenamine (37)[23]** Pre-catalyst **6a** (50 ppm) was used. The target compound was

isolated (158.6 mg, 97%) as a white solid by column chromatography (eluting with 0-5% ethyl acetate in petroleum ether). ^{1}H NMR (400 MHz, $CDCl_3$) δ: 7.54-7.47 (m, 2 H), 7.32-7.29 (m, 3 H), 7.28-7.24 (m, 3 H), 7.18-7.01 (m, 9 H) ppm. ^{13}C NMR (100 MHz, $CDCl_3$) δ: 147.5, 147.2, 144.3, 129.3, 128.6, 128.0, 126.7, 124.5, 124.0, 123.8, 123.0, 122.2 ppm.

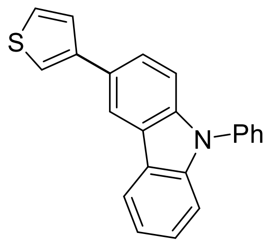

9-Phenyl-3-(3-thienyl)-9*H*-carbazole (38) Pre-catalyst **6a** (50 ppm) was used. The target compound was isolated (156.2 mg, 96%) as a white solid by column chromatography (eluting with petroleum ether), m.p. = 147-148 °C. ^{1}H NMR (400 MHz, $CDCl_3$) δ: 8.44-8.37 (m, 1 H), 8.23 (d, *J* = 8.0 Hz, 1 H), 7.73-7.60 (m, 5 H), 7.60-7.41 (m, 7 H), 7.40-7.32 (m, 1 H) ppm. ^{13}C NMR (100 MHz, $CDCl_3$) δ: 143.1, 141.3, 140.2, 137.6, 129.9, 128.3, 127.5, 127.1, 126.8, 126.2, 126.1, 125.0, 123.8, 123.4, 120.4, 120.1, 119.1, 118.1, 110.0, 110.0 ppm. HRMS (ESI): m/z calculated for $C_{22}H_{16}NS^+$ [M+H$^+$]: 326.0998, found: 326.0985.

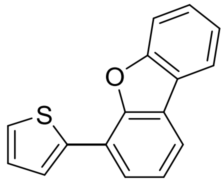

4-(2-Thienyl)dibenzofuran (39)[24] Pre-catalyst **6a** (50 ppm) was used. The target compound was isolated (120.0 mg, 96%) as an off white solid by column chromatography (eluting with 0-5% ethyl acetate in petroleum ether), m.p. = 59-60 °C. ^{1}H NMR (400 MHz, $CDCl_3$) δ: 8.03-7.96 (m, 2 H), 7.90 (dd, *J* = 8.0, 1.2 Hz, 1 H), 7.77 (dd, *J* = 8.0, 1.2 Hz, 1 H), 7.69 (d, *J* = 8.0 Hz, 1 H), 7.56-7.45 (m, 1 H), 7.46-7.36 (m, 3 H), 7.27-7.23 (m, 1 H) ppm. ^{13}C NMR (100 MHz, $CDCl_3$) δ: 156.2, 152.1, 138.6, 128.6, 128.0, 127.4, 127.2, 126.5, 125.3, 125.0, 124.1, 123.2, 123.0, 120.8, 119.4, 111.9 ppm.

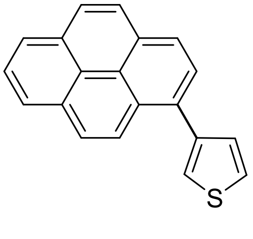

1-(3-Thienyl)pyrene (40) Pre-catalyst **6a** (50 ppm) was used. The target compound was isolated (137.9 mg, 97%) as a white solid by column chromatography (eluting with petroleum ether), m.p. = 85-86 °C. ^{1}H NMR (400 MHz, $CDCl_3$) δ: 8.36 (d, *J* = 8.0 Hz, 1 H), 8.26-8.18 (m, 3 H), 8.12 (s, 2 H), 8.11-8.02 (m, 3 H),

7.60-7.53 (m, 2 H), 7.52-7.48 (m, 1 H) ppm. ^{13}C NMR (100 MHz, $CDCl_3$) δ: 141.6, 132.4, 131.5, 131.0, 130.6, 130.0, 128.8, 127.6, 127.6, 127.4, 127.4, 127.3, 127.1, 126.1, 125.5, 125.2, 125.0, 124.9, 124.7, 123.9 ppm. HRMS (ESI): m/z calculated for $C_{20}H_{15}S^+$ [M+H$^+$]: 287.0889, found: 287.0895.

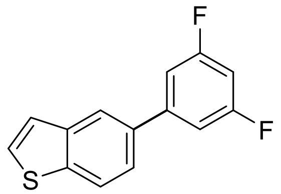

5-(3,5-Difluorophenyl)benzo[*b*]thiophene (41) Pre-catalyst **6a** (50 ppm) was used. The target compound was isolated (108.1 mg, 96%) as a white solid by column chromatography (eluting with petroleum ether), m.p. = 74-75 °C. ^{1}H NMR (400 MHz, $CDCl_3$) δ: 8.04-7.95 (m, 2 H), 7.59-7.51 (m, 2 H), 7.42 (d, J = 5.6 Hz, 1 H), 7.25-7.16 (m, 2 H), 6.87-6.78 (m, 1 H) ppm. ^{13}C NMR (100 MHz, $CDCl_3$) δ: 164.5 (dd, J = 246.0, 14.0 Hz), 144.7, 140.2, 135.3, 127.6, 124.0, 123.3, 123.0, 122.0, 110.2 (d, J = 26.0 Hz), 110.0 (d, J = 12.0 Hz), 102.4 (t, J = 25.0 Hz) ppm. ^{19}F NMR (376 MHz, $CDCl_3$) δ: -109.74 ppm. HRMS (ESI): m/z calculated for $C_{14}H_9F_2S^+$ [M+H$^+$]: 247.0488, found: 247.0485.

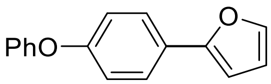

2-(4-Phenoxyphenyl)furan (42) Pre-catalyst **6a** (50 ppm) was used. The target compound was isolated (109.7 mg, 93%) as a white solid by column chromatography (eluting with petroleum ether), m.p. = 58-59 °C. ^{1}H NMR (400 MHz, $CDCl_3$) δ: 7.72 - 7.67 (m, 2 H), 7.49-7.45 (m, 1 H), 7.43-7.34 (m, 2 H), 7.20 - 7.13 (m, 1 H), 7.12-7.02 (m, 4 H), 6.65-6.59 (m, 1 H), 6.49-6.46 (m, 1 H) ppm. ^{13}C NMR (100 MHz, $CDCl_3$) δ: 157.0, 156.6, 153.6, 141.8, 129.8, 126.3, 125.3, 123.4, 119.1, 118.9, 111.6, 104.2 ppm. HRMS (ESI): m/z calculated for $C_{16}H_{13}O_2^+$ [M+H$^+$]: 237.0910, found: 237.0903.

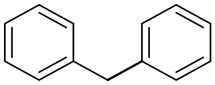

Diphenylmethane (43)[25] Pre-catalyst **6a** (50 ppm) was used. The target compound was isolated (81.5 mg, 97%) as a colorless oil by column chromatography (eluting with petroleum ether). ^{1}H NMR (400 MHz, $CDCl_3$) δ: 7.40-7.32 (m, 4 H), 7.31-7.22 (m, 6 H), 4.07 (s, 2 H) ppm. ^{13}C NMR (100 MHz, $CDCl_3$) δ: 141.2, 129.0, 128.5, 126.1, 42.0 ppm.

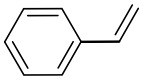

Styrene (44) Pre-catalyst **6a** (50 ppm) was used. The target compound was isolated (50.6 mg 97%) as a colorless oil by column chromatography (eluting with petroleum ether). ^{1}H NMR (400 MHz, $CDCl_3$) δ: 7.63-7.29 (m, 5 H), 6.98-6.68 (m, 1 H), 5.81 (d, *J* = 16.0 Hz, 1 H), 5.30 (d, *J* = 12.0 Hz, 1 H) ppm. ^{13}C NMR (100 MHz, $CDCl_3$) δ: 137.6, 136.9, 128.5, 127.8, 126.2, 113.8 ppm.

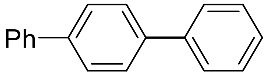

Diphenyl benzene (45)[26] Pre-catalyst **6a** (50 ppm) was used. The target compound was isolated (113.9 mg, 94%) as a white solid by column chromatography (eluting with petroleum ether). ^{1}H NMR (400 MHz, $CDCl_3$) δ: 7.74-7.65 (m, 8 H), 7.54-7.45 (m, 4 H), 7.43-7.37 (m, 2 H) ppm. ^{13}C NMR (100 MHz, $CDCl_3$) δ: 140.7, 140.1, 128.8, 127.5, 127.4, 127.1 ppm.

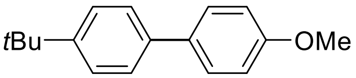

4-(1,1-Dimethylethyl)-4'-methoxy-1,1'-biphenyl (46)[27] Pre-catalyst **6a** (50 ppm) was used. The target compound was isolated (117.6 mg, 98%) as a white solid by column chromatography (eluting with petroleum ether). ^{1}H NMR (400 MHz, $CDCl_3$) δ: 7.58-7.50 (m, 4 H), 7.49-7.44 (m, 2 H), 7.03-6.96 (m, 2 H), 3.88 (s, 3 H), 1.39 (s, 9 H) ppm. ^{13}C NMR (100 MHz, $CDCl_3$) δ: 158.9, 149.6, 137.9, 133.6, 128.0, 126.4, 125.7, 114.1, 55.3, 34.5, 31.4 ppm.

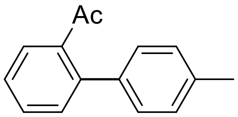

1-(4'-Methyl-[1,1'-biphenyl]-2-yl)ethan-1-one (47)[28] Pre-catalyst **6a** (50 ppm) was used. The target compound was isolated (98.7 mg, 94%) as a colorless oil by column chromatography (eluting with 0-10% ethyl acetate in petroleum ether). ^{1}H NMR (400 MHz, $CDCl_3$) δ: 7.59-7.49 (m, 2 H), 7.46-7.38 (m, 2 H), 7.27-7.24 (m, 4 H), 2.43 (s, 3 H), 2.04 (s, 3 H) ppm. ^{13}C NMR (100 MHz, $CDCl_3$) δ: 205.2, 140.9, 140.5, 137.8, 130.7, 130.2, 129.4, 128.7, 127.8, 127.2, 126.8, 30.5, 21.2 ppm.

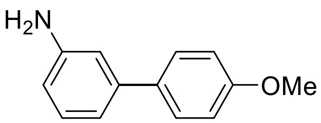

4'-Methoxy[1,1'-biphenyl]-3-amine (48)[29] Pre-catalyst **6a** (500 ppm) was used. The target compound was isolated (94.6 mg, 95%) as a white solid by column chromatography (eluting with 0-20% ethyl acetate in petroleum ether). ^{1}H NMR (400 MHz, $CDCl_3$) δ: 7.53 (d, *J* = 8.0 Hz, 2 H), 7.27-7.20 (m, 1 H), 7.03-6.95 (m, 3 H), 6.93-6.89 (m, 1 H), 6.71 - 6.63 (m, 1 H), 3.87 (s, 3 H), 3.76 (br, 2 H) ppm. ^{13}C NMR (100 MHz, $CDCl_3$) δ: 159.1, 146.7, 142.0, 133.9, 129.7, 128.1, 117.3, 114.1, 113.6, 113.5, 55.4 ppm.

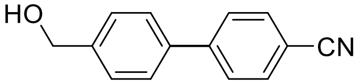

4-Cyano-4'-(hydroxymethyl)-1,1'-biphenyl (49)[30] Pre-catalyst **6a** (200 ppm) was used. The target compound was isolated (95.1 mg, 91%) as a white solid by column chromatography (eluting with 0-50% ethyl acetate in petroleum ether). ^{1}H NMR (400 MHz, $CDCl_3$) δ: 7.80-7.67 (m, 4 H), 7.65-7.57 (m, 2 H), 7.55-7.49 (m, 2 H), 4.80 (s, 2 H), 1.81 (br, 1 H) ppm. ^{13}C NMR (100 MHz, $CDCl_3$) δ: 145.3, 141.4, 138.5, 132.6, 127.7, 127.6, 127.4, 119.0, 110.9, 64.8 ppm.

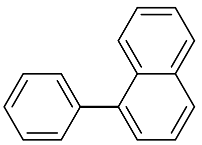

1-Phenylnaphthalene (50)[31] Pre-catalyst **6a** (200 ppm) was used. The target compound was isolated (93.9 mg, 92%) as a white solid by column chromatography (eluting with petroleum ether). ^{1}H NMR (400 MHz, $CDCl_3$) δ: 7.96 (d, *J* = 8.0 Hz, 2 H), 7.94-7.89 (m, 1 H), 7.61-7.52 (m, 6 H), 7.52-7.43 (m, 3 H) ppm. ^{13}C NMR (100 MHz, $CDCl_3$) δ: 140.8, 140.3, 133.8, 131.7, 130.1, 128.3, 127.6, 127.2, 126.9, 126.1, 126.0, 125.8, 125.6, 125.4 ppm.

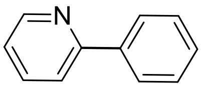

2-Phenylpyridine (51)[32] Pre-catalyst **6a** (50 ppm) was used. The target compound was isolated (76.0 mg, 98%) as a colorless oil by column chromatography (eluting with 0-2% ethyl acetate in petroleum ether). ^{1}H NMR (400 MHz, $CDCl_3$) δ: 8.77-8.68 (m, 1 H), 8.06-7.97 (m, 2 H), 7.80-7.70 (m, 2 H), 7.53-7.47 (m, 2 H), 7.47-7.41 (m, 1 H), 7.27-7.23 (m, 1 H) ppm. ^{13}C NMR (100 MHz, $CDCl_3$) δ: 149.7, 142.3, 139.4, 136.8, 129.0, 128.7,

126.9, 122.1, 120.6 ppm.

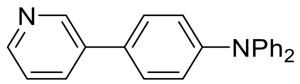

***N,N*-Diphenyl-4-(3-pyridinyl)benzenamine (52)**[33] Pre-catalyst **6a** (200 ppm) was used. The target compound was isolated (156.2 mg, 97%) as a yellow solid by column chromatography (eluting with 0-20% ethyl acetate in petroleum ether). ^{1}H NMR (400 MHz, $CDCl_3$) δ: 8.90-8.82 (m, 1 H), 8.63-8.50 (m, 1 H), 7.89-7.84 (m, 1 H), 7.52-7.45 (m, 2 H), 7.39-7.29 (m, 5 H), 7.22-7.13 (m, 6 H), 7.13-7.05 (m, 2 H) ppm. ^{13}C NMR (100 MHz, $CDCl_3$) δ: 148.0, 147.9, 147.4, 136.1, 133.8, 131.2, 129.4, 127.8, 124.9, 124.7, 123.6, 123.5, 123.3 ppm.

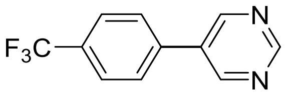

5-(4-(Trifluoromethyl)phenyl)pyrimidine (53)[34] Pre-catalyst **6a** (500 ppm) was used. The target compound was isolated (107.6 mg, 96%) as a white solid by column chromatography (eluting with 0-50% ethyl acetate in petroleum ether), m.p. = 99-101 °C. ^{1}H NMR (400 MHz, $CDCl_3$) δ: 9.29 (s, 1 H), 9.00 (s, 2 H), 7.84-7.79 (m, 2 H), 7.76-7.69 (m, 2 H) ppm. ^{13}C NMR (100 MHz, $CDCl_3$) δ: 158.2, 155.1, 137.8, 133.1, 128.8 (q, J = 32.0 Hz), 127.4, 126.4 (q, J = 4.0 Hz), 124.3 (q, J = 272.0 Hz) ppm. ^{19}F NMR (376 MHz, $CDCl_3$) δ: -62.66 ppm.

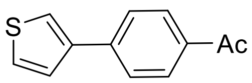

3-(4-Acetylphenyl)thiophene (54)[35] Pre-catalyst **6a** (200 ppm) was used. The target compound was isolated (97.0 mg, 96%) as a white solid by column chromatography(eluting with petroleum ether). ^{1}H NMR (400 MHz, $CDCl_3$) δ: 8.04-8.00 (m, 2 H), 7.74-7.69 (m, 2 H), 7.62-7.58 (m, 1 H), 7.48-7.43 (m, 2 H), 2.64 (s, 3 H) ppm. ^{13}C NMR (100 MHz, $CDCl_3$) δ: 197.5, 141.1, 140.2, 135.6, 129.0, 126.8, 126.4, 126.2, 122.0, 26.6 ppm.

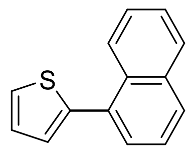

2-(1-Naphthyl)thiophene (55)[36] Pre-catalyst **6a** (200 ppm) was used. The target compound was isolated (95.6 mg, 91%) as a colorless oil by column chromatography (eluting with petroleum ether). ^{1}H NMR (400 MHz, $CDCl_3$) δ: 8.35-8.21 (m, 1 H), 7.97-7.87 (m, 2 H), 7.62

(d, J = 7.2 Hz, 1 H), 7.59-7.51 (m, 3 H), 7.51 - 7.43 (m, 1 H), 7.32-7.29 (m, 1 H), 7.26-7.19 (m, 1 H) ppm. ^{13}C NMR (100 MHz, $CDCl_3$) δ: 141.8, 133.8, 132.4, 131.9, 128.4, 128.4, 128.2, 127.4, 127.3, 126.5, 126.0, 125.8, 125.7, 125.3 ppm.

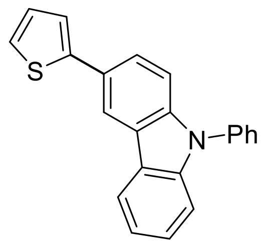

9-Phenyl-3-(2-thienyl)-9*H*-carbazole (56)[37] Pre-catalyst **6a** (200 ppm) was used. The target compound was isolated (157.6 mg, 97%) as a light yellow oil by column chromatography (eluting with petroleum ether. ^{1}H NMR (400 MHz, $CDCl_3$) δ: 8.42-8.37 (m, 1 H), 8.21 (d, J = 8.0 Hz, 1 H), 7.74-7.57 (m, 5 H), 7.54-7.29 (m, 7 H), 7.17-7.13 (m, 1 H) ppm. ^{13}C NMR (100 MHz, $CDCl_3$) δ: 145.6, 141.4, 140.4, 137.5, 129.9, 128.0, 127.6, 127.1, 126.9, 126.8, 126.3, 124.6, 123.9, 123.3, 122.3, 120.5, 120.2, 117.8, 110.1, 110.0 ppm.

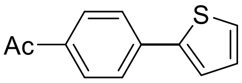

2-(4-Acetylphenyl)thiophene (57)[38] Pre-catalyst **6a** (200 ppm) was used. The target compound was isolated (92.9 mg, 92%) as a white solid by column chromatography (eluting with petroleum ether). ^{1}H NMR (400 MHz, $CDCl_3$) δ: 8.03-7.95 (m, 2 H), 7.76-7.68 (m, 2 H), 7.48-7.44 (m, 1 H), 7.43-7.37 (m, 1 H), 7.18-7.12 (m, 1 H), 2.64 (s, 3 H) ppm. ^{13}C NMR (100 MHz, $CDCl_3$) δ: 197.3, 143.0, 138.8, 135.8, 129.1, 128.4, 126.5, 125.7, 124.6, 26.6 ppm.

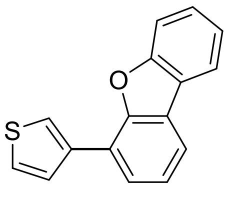

4-(3-Thienyl)dibenzofuran (58) Pre-catalyst **6a** (200 ppm) was used. The target compound was isolated (120.1 mg, 99%) as an off white solid by column chromatography (eluting with 0-5% ethyl acetate in petroleum ether), m.p. = 86-87 °C. ^{1}H NMR (400 MHz, $CDCl_3$) δ: 8.16-8.12 (m, 1 H), 8.01 (d, J = 7.6 Hz, 1 H), 7.92 (dd, J = 8.0, 1.2 Hz, 1 H), 7.79 (dd, J = 5.2, 1.2 Hz, 1 H), 7.75 (dd, J = 8.0, 1.2 Hz, 1 H), 7.67 (d, J = 8.0 Hz, 1 H), 7.55-7.49 (m, 2 H), 7.45-7.37 (m, 2 H) ppm. ^{13}C NMR (100 MHz, $CDCl_3$) δ: 156.1, 153.0, 136.6, 127.3, 127.1, 125.6, 125.5, 125.0, 124.2, 123.6, 123.1, 122.9, 120.7, 120.6, 119.3, 111.8 ppm. HRMS (ESI): m/z calculated for $C_{16}H_{11}OS^+$ [M+H$^+$]: 251.0525, found: 251.0515.

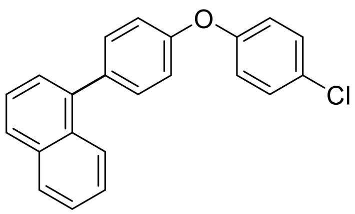

1-[4-(4-Chlorophenoxy)phenyl]naphthalene (59) Pre-catalyst **6a** (200 ppm) was used. The target compound was isolated (161.8 mg, 98%) as a colorless oil by column chromatography (eluting with petroleum ether). ^{1}H NMR (400 MHz, $CDCl_3$) δ: 7.99-7.94 (m, 2 H), 7.91 (d, *J* = 8.0 Hz, 1 H), 7.59-7.38 (m, 8 H), 7.19-7.10 (m, 4 H) ppm. ^{13}C NMR (100 MHz, $CDCl_3$) δ: 156.4, 155.8, 139.4, 138.6, 136.1, 133.8, 131.5, 129.9, 128.4, 128.2, 127.7, 127.0, 126.6, 126.1, 125.9, 125.4, 120.4, 118.5 ppm. HRMS (ESI): m/z calculated for $C_{22}H_{16}ClO^+$ [M+H$^+$]: 331.0884, found: 331.0887, 333.0855 (isotope abundance ratio = 3:1).

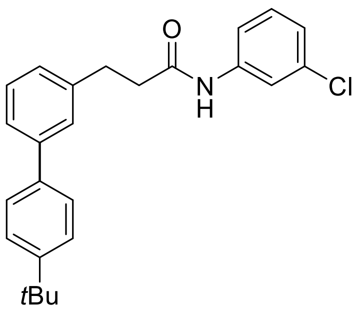

3-[4-(1,1-Dimethylethyl)phenyl]-*N*-(3-chlorophenyl)-benzene-propan-amide (60) Pre-catalyst **6a** (500 ppm) was used. The target compound was isolated (187.9 mg, 96%) as a white solid by column chromatography (eluting with 0-20% ethyl acetate in petroleum ether), m.p. = 151-153 °C. ^{1}H NMR (400 MHz, $CDCl_3$) δ: 7.63-7.57 (m, 1 H), 7.55-7.50 (m, 2 H), 7.50-7.45 (m, 4 H), 7.39 (t, *J* = 7.6 Hz, 1 H), 7.28-7.18 (m, 3 H), 7.15-7.05 (m, 2 H), 3.13 (t, *J* = 7.6 Hz, 2 H), 2.72 (t, *J* = 7.6 Hz, 2 H), 1.39 (s, 9 H) ppm. ^{13}C NMR (100 MHz, $CDCl_3$) δ: 150.4, 141.5, 140.8, 138.8, 138.0, 135.4, 134.6, 130.0, 129.1, 127.1, 127.0, 126.8, 125.8, 125.2, 124.4, 120.0, 117.8, 39.5, 34.6, 31.6, 31.4 ppm. HRMS (ESI): m/z calculated for $C_{25}H_{27}ClNO^+$ [M+H$^+$]: 392.1776, found: 392.1782, 394.1750 (isotope abundance ratio = 3:1).

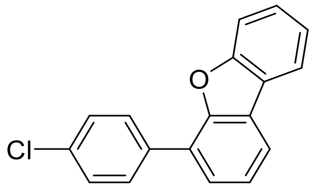

4-(4-Chlorophenyl)dibenzofuran (61) Pre-catalyst **6a** (200 ppm) was used. The target compound was isolated (135.2 mg, 97%) as a white solid by column chromatography (eluting with petroleum ether), m.p. = 76-77 °C. ^{1}H NMR (400 MHz, $CDCl_3$) δ: 8.02 (d, *J* = 8.0 Hz, 1 H), 7.98 (dd, *J* = 7.6, 1.6 Hz, 1 H), 7.94-7.83 (m, 2 H), 7.67-7.58 (m, 2 H), 7.57-7.37 (m, 5 H) ppm. ^{13}C

NMR (100 MHz, $CDCl_3$) δ: 156.1, 153.2, 134.8, 133.8, 130.1, 128.9, 127.4, 126.6, 125.0, 124.6, 124.1, 123.3, 122.9, 120.8, 120.0, 111.9 ppm. HRMS (ESI): m/z calculated for $C_{18}H_{12}ClO^+$ [$M+H^+$]: 279.0571, found: 279.0570, 281.0539 (isotope abundance ratio = 3:1).

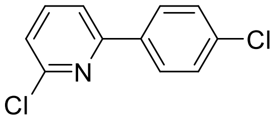

2-Chloro-6-(4-chlorophenyl)pyridine (62) Pre-catalyst **6a** (500 ppm) was used. The target compound was isolated (109.8 mg, 98%) as a colorless oil by column chromatography (eluting with 0-5% ethyl acetate in petroleum ether). ^{1}H NMR (400 MHz, $CDCl_3$) δ: 8.00-7.93 (m, 2 H), 7.73 (t, *J* = 8.0 Hz, 1 H), 7.68-7.62 (m, 1 H), 7.49-7.43 (m, 2 H), 7.30 (d, *J* = 8.0 Hz, 1 H) ppm. ^{13}C NMR (100 MHz, $CDCl_3$) δ: 156.8, 151.5, 139.4, 136.1, 135.8, 129.0, 128.3, 122.8, 118.4 ppm. HRMS (ESI): m/z calculated for $C_{11}H_8Cl_2N^+$ [$M+H^+$]: 224.0028, found: 224.0029, 225.9998, 227.9967 (isotope abundance ratio = 9:6:1).

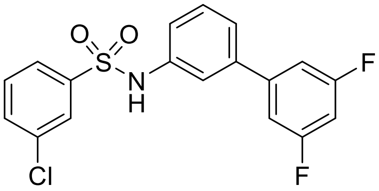

***N*-[3-(3,5-Difluorophenyl)phenyl]-3-chlorobenzene-sulfonamide (63)** Pre-catalyst **6a** (500 ppm) was used. The target compound was isolated (180.4 mg, 95%) as a white solid by column chromatography (eluting with 0-20% ethyl acetate in petroleum ether), m.p. = 102-103 °C. H NMR (400 MHz, $CDCl_3$) δ: 7.82-7.74 (m, 2 H), 7.49-7.42 (m, 2 H), 7.40-7.31 (m, 2 H), 7.31-7.29 (m, 1 H), 7.18 - 6.97 (m, 4 H), 6.87-6.77 (m, 1 H) ppm. ^{13}C NMR (100 MHz, $CDCl_3$) δ: 164.6 (dd, *J* = 247.0, 13.0 Hz), 140.3, 139.9, 137.2, 136.7, 132.3, 130.4, 130.2, 129.5, 128.7, 124.4, 121.4, 120.2, 110.1 (d, *J* = 26 Hz), 110.0 (d, *J* = 12.0 Hz), 103.1 (t, *J* = 26.0 Hz) ppm. ^{19}F NMR (376 MHz, CDCl3) δ: -109.22 ppm. HRMS (ESI): m/z calculated for $C_{18}H_{13}ClF_2NO_2S^+$ [$M+H^+$]: 380.0318, found: 380.0317, 382.0285 (isotope abundance ratio = 3:1).

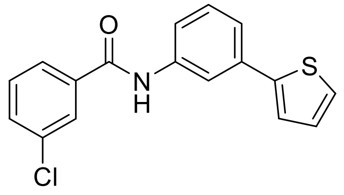

N-[3-(2-Thienyl)phenyl]-3-chloro-benzamide (64) Pre-catalyst **6a** (500 ppm) was used. The target compound was isolated (144.3 mg, 92%) as a white solid by column chromatography (eluting with 0-20% ethyl acetate in petroleum ether), m.p. = 116-118 °C. ^{1}H NMR (400 MHz, $CDCl_3$) δ: 7.98-7.84 (m, 3 H), 7.81-7.71 (m, 1 H), 7.63-7.29 (m, 7 H), 7.16-7.08 (m, 1 H) ppm. ^{13}C NMR (100 MHz, $CDCl_3$) δ: 164.4, 143.7, 138.1, 136.6, 135.4, 135.0, 132.0, 130.1, 129.7, 128.1, 127.8, 127.4, 125.2, 123.6, 122.4, 119.3, 117.7 ppm. HRMS (ESI): m/z calculated for $C_{17}H_{13}ClNOS^+$ [M+H$^+$]: 314.0401, found: 314.0404, 316.0372 (isotope abundance ratio = 3:1).

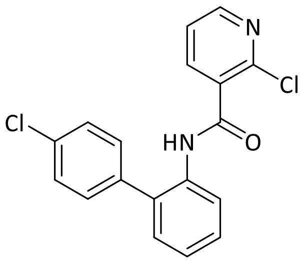

Boscalid (65)[39] Pre-catalyst **6a** (500 ppm) was used. The target compound was isolated (157.9 mg, 92%) as a white solid by column chromatography (eluting with 0-5% ethyl acetate in petroleum ether). ^{1}H NMR (400 MHz, $CDCl_3$) δ: 8.45 (dd, J = 4.8, 1.6 Hz, 1H), 8.41 (d, J = 8.0 Hz, 1H), 8.15 (dd, J = 8.0, 2.0 Hz, 2H), 7.43-7.48 (m, 3H), 7.33-7.37 (m, 4H), 7.27 (br, 1H) ppm. ^{13}C NMR (100 MHz, $CDCl_3$) δ: 162.7, 151.2, 146.7, 140.0, 136.4, 134.4, 134.3, 132.6, 131.2, 130.9, 130.4, 129.3, 128.9, 125.5, 123.0, 122.5 ppm.

7. General procedure and characterization results for Scheme 6

7.1. General procedure for Scheme 6

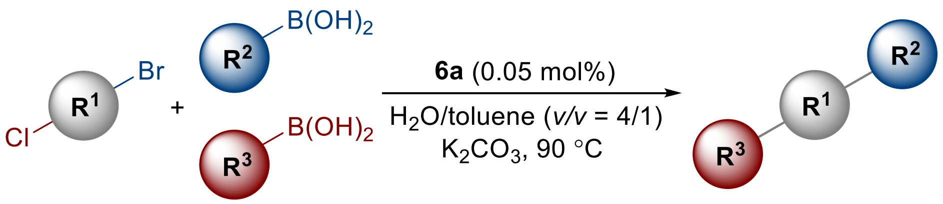

A 10 mL Schlenk tube equipped with a stir bar was charged with the first type of boronic acid (0.51 mmol), dihalogen compound (0.5 mmol), K_2CO_3 (138.0 mg, 1.0 mmol), pre-catalyst **6a** (200 μL solution of **6a** in Table S1, entry 1), 0.2 mL

toluene and 0.8 mL deionized H_2O under N_2 atmosphere. The mixture was stirred at 90 °C. After 1 h, the second boronic acid (0.55 mmol) was added under N_2, and the mixture was stirred at 90 °C for 5 h. After reaction, the tube was cooled to room temperature. The resulting mixture was extracted with ethyl acetate for three times. The combined organic layers were dried over Na_2SO_4 and concentrated under vacuum. The collected residue was purified by column chromatography using silica gel to give target compound. The product was characterized by ^{1}H, ^{13}C and ^{19}F NMR measurements. Unknown compounds were also confirmed with HRMS.

7.2. Characterization results for compounds in Scheme 6

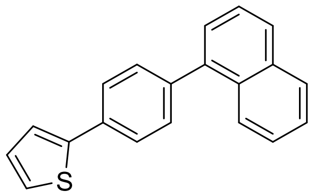

3-(4-(Naphthalen-1-yl)phenyl)thiophene (66) The target compound was isolated (132.9 mg, 93%) as a white solid by column chromatography (eluting with petroleum ether), m.p. = 142-144 °C. ^{1}H NMR (400 MHz, $CDCl_3$) δ: 8.00 (d, *J* = 8.0 Hz, 1 H), 7.95 (d, *J* = 8.0 Hz, 1 H), 7.91 (d, *J* = 8.0 Hz, 1 H), 7.79-7.74 (m, 2 H), 7.62-7.43 (m, 9 H) ppm. ^{13}C NMR (100 MHz, $CDCl_3$) δ: 142.0, 139.8, 139.6, 134.8, 133.8, 131.6, 130.5, 128.3, 127.7, 127.0, 126.9, 126.4, 126.3, 126.1, 126.0, 125.8, 125.4, 120.4 ppm. HRMS (ESI): m/z calculated for $C_{20}H_{15}S^+$ [M+H$^+$]: 287.0889, found: 287.0889.

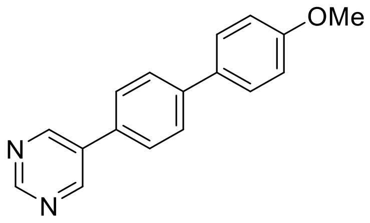

5-(4'-methoxy-[1,1'-biphenyl]-4-yl)pyrimidine (67)[40] The target compound was isolated (124.6 mg, 95 %) as a white solid by column chromatography (eluting with petroleum ether). ^{1}H NMR (400 MHz, $CDCl_3$) δ: 9.20 (s, 1 H), 8.97 (s, 2 H), 7.75-7.63 (m, 4 H), 7.57 (d, *J* = 8.0 Hz, 2 H), 7.04 (d, *J* = 8.0 Hz, 2 H), 3.96 (s, 3 H) ppm. ^{13}C NMR (100 MHz, $CDCl_3$) δ: 159.4, 157.1, 156.8, 154.4, 141.5, 133.9, 132.3, 128.1, 127.6, 127.2, 114.3, 55.4 ppm.

2-(3,5-Difluorophenyl)-4-(3,5-dimethoxyphenyl)-pyridine (68) The target compound was isolated (148.8 mg, 91%) as a white solid by column chromatography (eluting with 0-5% ethyl acetate in petroleum ether), m.p. = 122-124 °C. ^{1}H NMR (400 MHz, $CDCl_3$) δ: 8.75 (d, *J* = 5.2 Hz, 1 H), 7.92-7.84 (m, 1 H), 7.72-7.57 (m, 2 H), 7.50 (dd, *J* = 5.4, 2.0 Hz, 1 H), 6.96 - 6.86 (m, 1 H), 6.85-6.77 (m, 2 H), 6.65-6.52 (m, 1 H), 3.91 (s, 6 H) ppm. ^{13}C NMR (100 MHz, $CDCl_3$) δ: 164.6 (dd, *J* = 246.0, 12.0 Hz), 161.4, 159.6, 155.4, 150.2, 149.7, 121.4, 118.8, 110.0 (d, *J* = 27.0 Hz), 109.8 (d, *J* = 11.0 Hz),105.4, 104.3 (t, *J* = 26.0 Hz), 100.8, 55.6 ppm. ^{19}F NMR (376 MHz, $CDCl_3$) δ: -109.47 ppm. HRMS (ESI): m/z calculated for $C_{19}H_{16}F_2NO_2^+$ [M+H$^+$]: 328.1144, found: 328.1144.

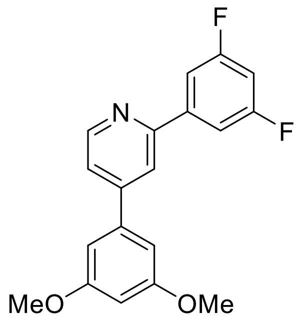

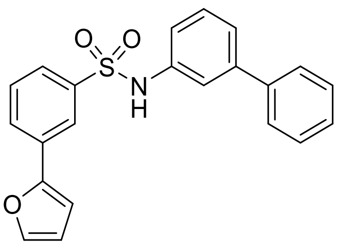

***N*-([1,1'-biphenyl]-3-yl)-3-(furan-2-yl)benzenesulfonamide (69)** The target compound was isolated (174.6 mg, 93%) as a white solid by column chromatography (eluting with 0-20% ethyl acetate in petroleum ether), m.p. = 131-133 °C. ^{1}H NMR (400 MHz, $CDCl_3$) δ: 8.19-8.13 (m, 1 H), 7.84 (d, *J* = 8.0 Hz, 1 H), 7.67 (d, *J* = 8.0 Hz, 1 H), 7.51-7.33 (m, 10 H), 7.09 (d, *J* = 7.6 Hz, 1 H), 6.86 (br, 1 H), 6.73-6.68 (m, 1 H), 6.53-6.48 (m, 1 H) ppm. ^{13}C NMR (100 MHz, $CDCl_3$) δ: 143.1, 136.7, 129.8, 129.5, 129.0, 128.8, 128.1, 127.9, 127.7, 127.4, 127.1, 127.0, 125.5, 124.4, 122.4, 122.3, 120.5, 120.5, 112.0, 106.9 ppm. HRMS (ESI): m/z calculated for $C_{22}H_{17}NO_3S^+$ [M+H$^+$]: 226.1358, found: 226.1366.

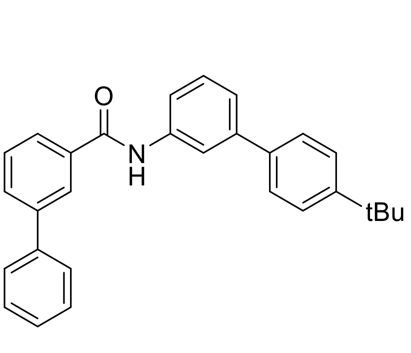

***N*-(4'-(tert-butyl)-[1,1'-biphenyl]-3-yl)-[1,1'-biphenyl]-3-carboxamide (70)** The target compound was isolated (186.3 mg, 92%) as a light yellow solid by column chromatography (eluting with 0-20% ethyl acetate in petroleum ether), m.p. = 58-60 °C. ^{1}H NMR (400 MHz, $CDCl_3$) δ: 8.16-7.98

(m, 2 H), 7.94-7.76 (m, 3 H), 7.71-7.62 (m, 3 H), 7.62-7.54 (m, 3 H), 7.53-7.39 (m, 7 H), 1.39 (s, 9 H) ppm. ^{13}C NMR (100 MHz, $CDCl_3$) δ: 166.2, 150.5, 142.0, 141.8, 140.1, 138.4, 137.7, 135.5, 130.5, 129.4, 129.0, 127.9, 127.2, 126.8, 126.0, 125.8, 123.3, 120.5, 119.1, 119.0, 118.4, 34.6, 31.4.ppm. HRMS (ESI): m/z calculated for $C_{29}H_{28}NO^+$ [M+H$^+$]: 406.2166, found: 406.2166.

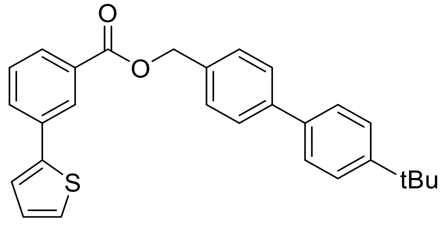

(4'-(tert-butyl)-[1,1'-biphenyl]-4-yl)methyl 3-(thiophen-2-yl)benzoate (71) The target compound was isolated (200.5 mg, 94%) as a white solid by column chromatography (eluting with 0-5% ethyl acetate in petroleum ether), m.p. = 51-53 °C. ^1H NMR (400 MHz, $CDCl_3$) δ: 8.40-8.34 (m, 1 H), 8.03 (d, J = 7.6 Hz, 1 H), 7.83 (d, J = 7.6 Hz, 1 H), 7.65 (d, J = 8.0 Hz, 2 H), 7.60-7.54 (m, 4 H), 7.53-7.47 (m, 3 H), 7.43-7.38 (m, 1 H), 7.37-7.33 (m, 1 H), 7.14-7.11 (m, 1 H), 5.46 (s, 2 H), 1.40 (s, 9 H) ppm. ^{13}C NMR (100 MHz, $CDCl_3$) δ: 150.5, 141.1, 137.8, 134.8, 134.8, 134.6, 130.8, 130.4, 129.0, 128.7, 128.5, 128.2, 127.6, 127.2, 127.0, 126.8, 125.8, 125.5, 123.9, 66.7, 34.6, 31.4 ppm. HRMS (ESI): m/z calculated for $C_{28}H_{27}O_2S^+$ [M+H$^+$]: 427.1727, found: 427.1726.

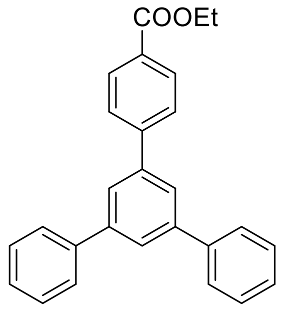

3',5'-diphenyl-biphenyl-4-carboxylic acid ethyl ester (72) The target compound was isolated (181.6 mg, 96%) as a colorless solid by column chromatography (eluting with 0-5% ethyl acetate in petroleum ether), m.p. = 81-83 °C. ^1H NMR (400 MHz, $CDCl_3$) δ: 8.25-8.16 (m, 2 H), 7.88-7.82 (m, 3 H), 7.83-7.78 (m, 2 H), 7.77-7.71 (m, 4 H), 7.57-7.50 (m, 4 H), 7.47-7.41 (m, 2 H), 4.46 (q, J = 7.2 Hz, 2 H), 1.47 (t, J = 7.2 Hz, 3 H) ppm. ^{13}C NMR (100 MHz, $CDCl_3$) δ: 166.5, 145.4, 142.6, 141.2, 140.9, 130.2, 129.5, 128.9, 127.7, 127.4, 127.2, 126.0, 125.2, 61.1, 14.4 ppm. HRMS (ESI): m/z calculated for $C_{27}H_{23}O_2^+$ [M+H$^+$]: 379.1693, found: 379.1693.

8. General procedure and characterization results for Scheme 7

8.1. General material information for continuous-flow setup

The equipment configuration used for continuous-flow syntheses is depicted in Fig. 5 and Scheme 7. All PFA connecting tubings, connectors, nuts, frits, fittings and back-pressure regulators were purchased from IDEX Health and Science. Stainless steel tubing and related tube fittings were purchased from Swagelok. Pumps was purchased from Tansoole Co. The stainless steel (SS) packed-bed reactor (30 cm length, OD × ID = 0.25″ × 0.17″) was packed with SS beads (120-150 mesh). Connections of reactor were made using stainless steel tube fittings (OD × ID = 0.25″ × 0.0625″) and SS tubings (OD × ID = 0.0625″ × 0.04″). Other connections were made using super flangeless ferrules with 1/4-28 flangeless nuts and PFA connecting tubings (OD × ID = 0.0625″ × 0.04″). The mixers and back-pressure regulators used in this work were made of PEEK.

8.2. General procedure for Scheme 7

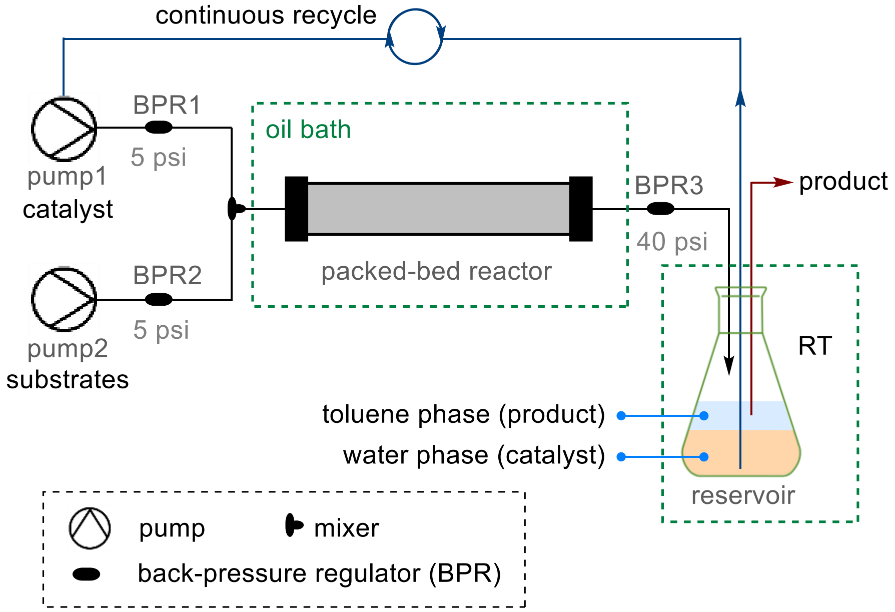

Fig. S3 Continuous-catalyst-recycling flow synthesis based on flow chemistry. (Setup in Scheme 7 and Fig. 5).

General procedure for the flow synthesis:

The packed-bed reactor was assembled according to the literature procedure.[41, 42] A 10 mL round-bottom flask was charged with K_2CO_3 (1.38 g, 10 mmol), Pre-catalyst **6a** (100-500 μL solution of **6a** in Table S1, entry 1. For example, for the synthesis of compound **73**, 100 μL pre-catalyst solution was used) and 4 mL deionized H_2O under N_2 atmosphere. Another 10 mL round-bottom flask was charged with (hetero)aryl halide (20 mmol, 2.5 M) and boronic acid pinacol ester (21 mmol). Deoxygenized toluene was added into this flask to make the solution volume of 8 mL under N_2 atmosphere. The flasks were sonicated to accelerate the dissolution of solids. Following the setup in Fig. S3, the first solution of pre-catalyst was loaded into the container of pump 1. The second solution of substrates was loaded into the container of pump 2. Before reaction, all connecting tubings from pumps to the collection container (reservoir) and the packed-bed reactor were filled with deoxygenized toluene. The collection container was kept under inert atmosphere with a balloon filled with N_2 at room temperature. The flow rate for solution of pump 1 was 40 μL/min. The flow rate for solution of pump 2 was 10 μL/min. The packed-bed reactor was immersed in a preheated oil bath at 110 °C. After reaching the steady state in 60 min (monitored by GC analysis of the toluene layer in the reservoir), the organic phase was removed, and the collection was started. Before and after reaching the steady state, the water solution containing catalyst was also collected in the same reservoir, and the water solution was continuously re-injected into the packed-bed reactor as pumped by pump 1. After a collection of 500 min, the organic layer was separated and the water phase was extracted with ethyl acetate for 3 times. The combined organic layers were rinsed with brine, dried over Na_2SO_4, and concentrated under vacuum. The collected residue was purified by column chromatography using silica gel to give target compound. The products were characterized by ^1H and ^{13}C NMR measurements.

8.3. Characterization results for compounds in Scheme 7

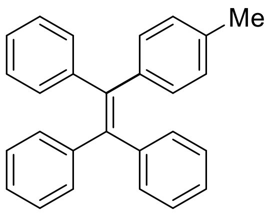

1-Methyl-4-(1,2,2-triphenylethenyl)benzene (73)[43] Pre-catalyst **6a** (10 ppm) was used. The target compound was isolated (4.15 g, 96%) as a white solid by column chromatography (eluting with petroleum ether). ^{1}H NMR (400 MHz, $CDCl_3$) δ: 7.18-7.02 (m, 15 H), 6.97-6.91 (m, 4 H), 2.29 (s, 3 H) ppm. ^{13}C NMR (100 MHz, $CDCl_3$) δ: 143.9, 140.7, 140.4, 136.1, 131.4, 131.3, 131.2, 130.3, 129.5, 128.4, 127.7, 127.6, 126.3, 126.3, 21.2 ppm.

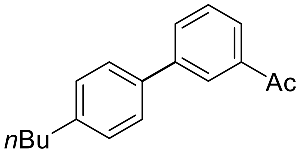

1-(4'-Butyl-[1,1'-biphenyl]-3-yl)ethan-1-one (74)[44] Pre-catalyst **6a** (10 ppm) was used. The target compound was isolated (2.99 g, 95%) as a colorless oil by column chromatography (eluting with 0-10% ethyl acetate in petroleum ether). ^{1}H NMR (400 MHz, $CDCl_3$) δ: 8.23-8.18 (m, 1 H), 7.94 (d, *J* = 8.0 Hz, 1 H), 7.81 (d, *J* = 8.0 Hz, 1 H), 7.59-7.54 (m, 3 H), 7.34-7.29 (m, 2 H), 2.71 (d, *J* = 8.0 Hz, 2 H), 2.68 (s, 3 H),1.72-1.65 (m, 2 H), 1.48-1.39 (m, 2 H), 0.98 (t, *J* = 7.2 Hz, 3 H) ppm. ^{13}C NMR (100 MHz, $CDCl_3$) δ: 198.2, 142.7, 141.7, 137.6, 137.5, 131.6, 129.0, 129.0, 127.0, 126.9, 126.8, 35.3, 33.6, 26.8, 22.4, 14.0 ppm.

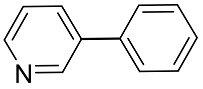

3-Phenylpridine (75)[45] Pre-catalyst **6a** (50 ppm) was used. The target compound was isolated (1.78 g, 92%) as a colorless oil by column chromatography (eluting with 0-10% ethyl acetate in petroleum ether). ^{1}H NMR (400 MHz, $CDCl_3$) δ: 8.92-8.84 (m, 1 H), 8.65-8. 58 (m, 1 H), 7.93 - 7.85 (m, 1 H), 7.64-7.58 (m, 2 H), 7.53-7.46 (m, 2 H), 7.46-7.34 (m, 2 H) ppm. ^{13}C NMR (100 MHz, $CDCl_3$) δ: 148.4, 148.3, 137.8, 136.7, 134.4, 129.1, 128.1, 127.1, 123.5 ppm.

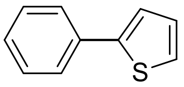

2-Phenylthiophene (76)[46] Pre-catalyst **6a** (20 ppm) was used. The target compound was isolated (1.88 g, 94%) as a white solid by column chromatography (eluting with petroleum ether). ^{1}H NMR

(400 MHz, $CDCl_3$) δ: 7.70-7.62 (m, 2 H), 7.45-7.37 (m, 2 H), 7.35-7.29 (m, 3 H), 7.17-7.08 (m, 1 H) ppm. ^{13}C NMR (100 MHz, $CDCl_3$) δ: 134.4, 128.9, 128.8, 128.0, 127.5, 126.0, 124.8, 123.1 ppm.

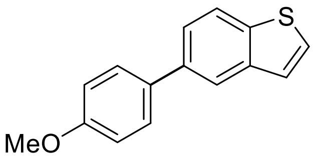

5-(4-Methoxyphenyl)benzo[*b*]thiophene (77)[47] Pre-catalyst **6a** (20 ppm) was used. The target compound was isolated (2.80 g, 93%) as a white solid by column chromatography (eluting with petroleum ether). ^{1}H NMR (400 MHz, $CDCl_3$) δ: 8.04-7.98 (m, 1 H), 7.94 (d, *J* = 8.4 Hz, 1 H), 7.66-7.60 (m, 2 H), 7.58 (dd, *J* = 8.4, 2.0 Hz, 1 H), 7.52-7.46 (m, 1 H), 7.40 (d, *J* = 5.6 Hz, 1 H), 7.07-7.00 (m, 2 H), 3.90 (s, 3 H) ppm. ^{13}C NMR (100 MHz, $CDCl_3$) δ: 159.1, 140.2, 138.2, 137.3, 133.9, 128.4, 127.0, 124.0, 123.7, 122.6, 121.5, 114.3, 55.4 ppm.

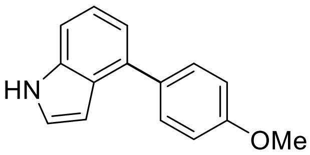

4-(4-Methoxyphenyl)indole (78)[48] Pre-catalyst **6a** (20 ppm) was used. The target compound was isolated (2.61 g, 94%) as a light yellow solid by column chromatography (eluting with 0-10% ethyl acetate in petroleum ether). ^{1}H NMR (400 MHz, $CDCl_3$) δ: 8.28 (br, 1 H), 7.73-7.64 (m, 2 H), 7.45-7.29 (m, 2 H), 7.27-7.25 (m, 1 H), 7.19 (d, *J* = 7.2 Hz, 1 H), 7.09-7.02 (m, 2 H), 6.80-6.72 (m, 1 H), 3.91 (s, 3 H) ppm. ^{13}C NMR (100 MHz, $CDCl_3$) δ: 158.8, 136.2, 134.1, 133.8, 129.8, 124.3, 122.3, 119.4, 113.9, 110.2, 109.8, 102.2, 55.4 ppm.

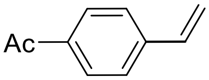

4-Acetylstyrene (79)[49] Pre-catalyst **6a** (20 ppm) was used. The target compound was isolated (1.66 g, 91%) as a colorless oil by column chromatography (eluting with 0-10% ethyl acetate in petroleum ether). ^{1}H NMR (400 MHz, $CDCl_3$) δ: 8.01-7.91 (m, 2 H), 7.50 (d, *J* = 8.0 Hz, 2 H), 6.84 -6.73 (m, 1 H), 5.90 (d, *J* = 17.6 Hz, 1 H), 5.42 (d, *J* = 10.8 Hz, 1 H), 2.62 (s, 3 H) ppm. ^{13}C NMR (100 MHz, $CDCl_3$) δ: 197.6, 157.0, 142.1, 135.9, 128.7, 126.3, 116.8, 26.7 ppm.

9. Reference

1. P. M. Reichstein, S. Goedrich, G. Papastavrou and M. Thelakkat, *Macromolecules*, 2016, **49**, 5484-5493.
2. T. B. Shrestha, D. L. Troyer and S. H. Bossmann, *Synthesis*, 2014, **46**, 646-652, 647 pp.
3. Y. Liu, B. Lou, L. Shangguan, J. Cai, H. Zhu and B. Shi, *Macromolecules*, 2018, **51**, 1351-1356.
4. M. E. Hart, K. L. Suchland, M. Miyakawa, J. R. Bunzow, D. K. Grandy and T. S. Scanlan, *J. Med. Chem.*, 2006, **49**, 1101-1112.
5. G. Evindar and R. A. Batey, *J. Org. Chem.*, 2006, **71**, 1802-1808.
6. P. R. Melvin, A. Nova, D. Balcells, W. Dai, N. Hazari, D. P. Hruszkewycz, H. P. Shah and M. T. Tudge, *ACS Catal.*, 2015, **5**, 3680-3688.
7. F. M. Piller, P. Appukkuttan, A. Gavryushin, M. Helm and P. Knochel, *Angew. Chem., Int. Ed.*, 2008, **47**, 6802-6806.
8. F.-Y. Mo, D. Qiu, Y.-B. Jiang, Y. Zhang and J.-B. Wang, *Tetrahedron Lett.*, 2011, **52**, 518-522.
9. M. Cowart, R. Faghih, M. P. Curtis, G. A. Gfesser, Y. L. Bennani, L. A. Black, L. Pan, K. C. Marsh, J. P. Sullivan, T. A. Esbenshade, G. B. Fox and A. A. Hancock, *J. Med. Chem.*, 2005, **48**, 38-55.
10. W. K. Chow, O. Y. Yuen, C. M. So, W. T. Wong and F. Y. Kwong, *J. Org. Chem.*, 2012, **77**, 3543-3548.
11. A. N. Marziale, D. Jantke, S. H. Faul, T. Reiner, E. Herdtweck and J. Eppinger, *Green Chem.*, 2011, **13**, 169-177.
12. Y. Izawa, D. Pun and S. S. Stahl, *Science*, 2011, **333**, 209-213.
13. H. Ji, L.-Y. Wu, J.-H. Cai, G.-R. Li, N.-N. Gan and Z.-H. Wang, *RSC Advances*, 2018, **8**, 13643-13648.
14. D. B. Pacardo, M. Sethi, S. E. Jones, R. R. Naik and M. R. Knecht, *ACS Nano*, 2009, **3**, 1288-1296.
15. P. R. Melvin, A. Nova, D. Balcells, W. Dai, N. Hazari, D. P. Hruszkewycz, H. P. Shah and M. T. Tudge, *ACS Catal.*, 2015, **5**, 3680-3688.
16. S. Shi and Y. Zhang, *Green Chem.*, 2008, **10**, 868-872.

17. S. Santra, P. K. Hota, R. Bhattacharyya, P. Bera, P. Ghosh and S. K. Mandal, *ACS Catal.*, 2013, **3**, 2776-2789.
18. J. Zhao, F. Dang, Z. Feng, B. Liu, X. Yang, Y. Wu, G. Zhou, Z. Wu and W.-Y. Wong, *Chem. Commun.*, 2017, **53**, 7581-7584.
19. P. Leowanawat, N. Zhang, A.-M. Resmerita, B. M. Rosen and V. Percec, *J. Org. Chem.*, 2011, **76**, 9946-9955.
20. Y. Kitamura, S. Sako, A. Tsutsui, Y. Monguchi, T. Maegawa, Y. Kitade and H. Sajiki, *Adv. Synth. Catal.*, 2010, **352**, 718-730.
21. S. Handa, Y. Wang, F. Gallou and B. H. Lipshutz, *Science*, 2015, **349**, 1087-1091.
22. G. A. Molander and F. Beaumard, *Org. Lett.*, 2010, **12**, 4022-4025.
23. Y. J. Chang and T. J. Chow, *Tetrahedron*, 2009, **65**, 9626-9632.
24. A. S. K. Hashmi, W. Yang and F. Rominger, *Chem. Eur. J*, 2012, **18**, 6576-6580.
25. L. Offner-Marko, A. Bordet, G. Moos, S. Tricard, S. Rengshausen, B. Chaudret, K. L. Luska and W. Leitner, *Angew. Chem., Int. Ed.*, 2018, **57**, 12721-12726.
26. B. Singh, R. Paira, G. Biswas, B. K. Shaw and S. K. Mandal, *Chem. Commun.*, 2018, **54**, 13220-13223.
27. D. Paul, S. Rudra, P. Rahman, S. Khatua, M. Pradhan and P. N. Chatterjee, *J. Organomet. Chem.*, 2018, **871**, 96-102.
28. L. J. Goossen, B. Zimmermann and T. Knauber, *Angew. Chem., Int. Ed.*, 2008, **47**, 7103-7106.
29. J. Wei, K.-M. Liu and X.-F. Duan, *J. Org. Chem.*, 2017, **82**, 1291-1300.
30. Y. H. Park, H. R. Ahn, B. Canturk, S. I. Jeon, S. Lee, H. Kang, G. A. Molander and J. Ham, *Org. Lett.*, 2008, **10**, 1215-1218.
31. J. Kim and S. H. Hong, *ACS Catal.*, 2017, **7**, 3336-3343.
32. F. Schroeter, J. Soellner and T. Strassner, *Organometallics*, 2018, **37**, 4267-4275.
33. C. Liu, X. Rao, X. Song, J. Qiu and Z. Jin, *RSC Adv.*, 2013, **3**, 526-531.
34. G. Ranjani and R. Nagarajan, *Org. Lett.*, 2017, **19**, 3974-3977.

35. A. Derible, C. Diebold, J. Dentzer, R. Gadiou, J.-M. Becht and C. Le Drian, *Eur. J. Org. Chem.*, 2014, **2014**, 7699-7706.
36. X.-B. Lan, F.-M. Chen, B.-B. Ma, D.-S. Shen and F.-S. Liu, *Organometallics*, 2016, **35**, 3852-3860.
37. J. Song, F. Wei, W. Sun, X. Cao, C. Liu, L. Xie and W. Huang, *Org. Chem. Front.*, 2014, **1**, 817-820.
38. Y. Yamamoto, M. Takizawa, X.-Q. Yu and N. Miyaura, *Angew. Chem., Int. Ed.*, 2008, **47**, 928-931.
39. Y. Wang, Y. Wu, Y. Li and Y. Tang, *Chemical Science*, 2017, **8**, 3852-3857.
40. X. Li, C. Liu, L. Wang, Q. Ye, X. Jin and Z. Jin, *Org. Biomol. Chem.*, 2018, **16**, 8719-8723.
41. M. Chen, S. Ichikawa and S. L. Buchwald, *Angew. Chem., Int. Ed.*, 2015, **54**, 263-266.
42. J. R. Naber and S. L. Buchwald, *Angew. Chem., Int. Ed.*, 2010, **49**, 9469-9474.
43. M. Banerjee, S. J. Emond, S. V. Lindeman and R. Rathore, *J. Org. Chem.*, 2007, **72**, 8054-8061.
44. S. B. Blakey and D. W. C. MacMillan, *J. Am. Chem. Soc.*, 2003, **125**, 6046-6047.
45. A.-C. Bedard, A. Adamo, K. C. Aroh, M. G. Russell, A. A. Bedermann, J. Torosian, B. Yue, K. F. Jensen and T. F. Jamison, *Science*, 2018, **361**, 1220-1225.
46. A. Banik, R. Paira, B. K. Shaw, G. Vijaykumar and S. K. Mandal, *J. Org. Chem.*, 2018, **83**, 3236-3244.
47. Y. Qiu, D. Ma, C. Fu and S. Ma, *Tetrahedron*, 2013, **69**, 6305-6312.
48. F. Petronijevic, C. Timmons, A. Cuzzupe and P. Wipf, *Chem. Commun.*, 2009, 104-106.
49. G. Wienhoefer, F. A. Westerhaus, R. V. Jagadeesh, K. Junge, H. Junge and M. Beller, *Chem. Commun.*, 2012, **48**, 4827-4829.

10. NMR Spectra

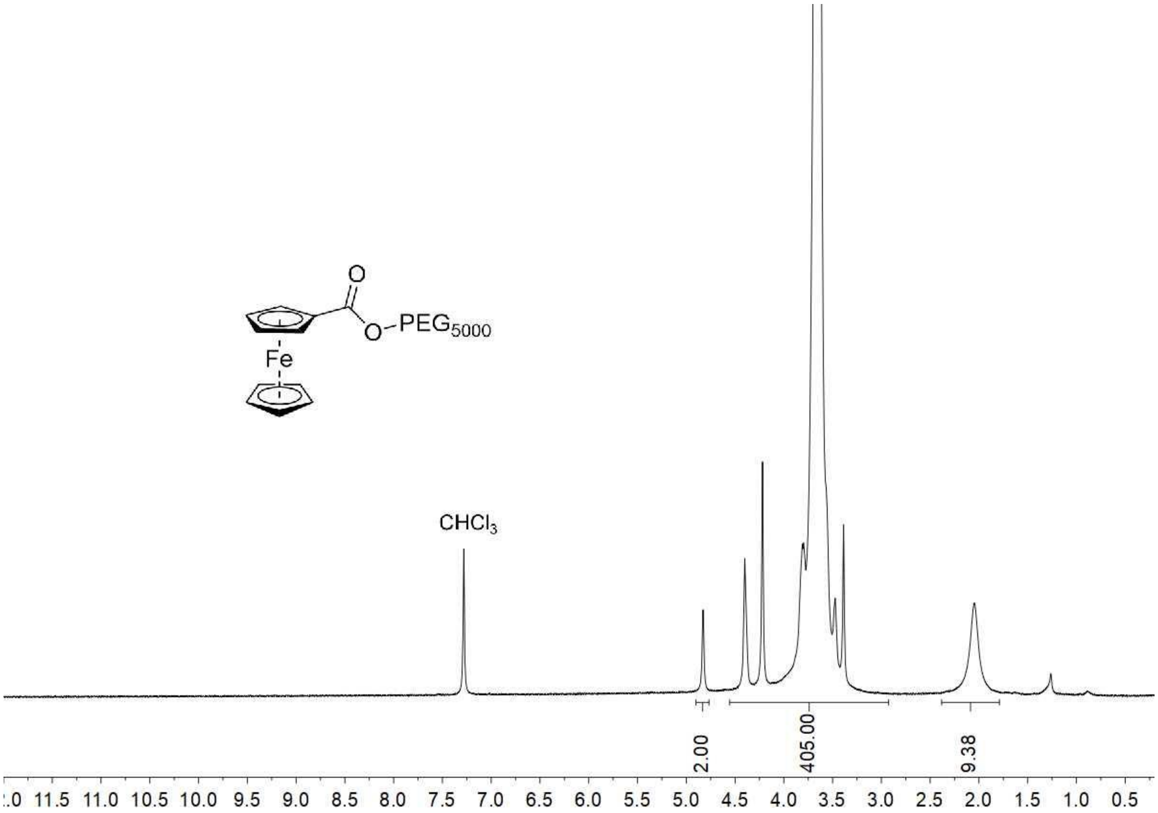

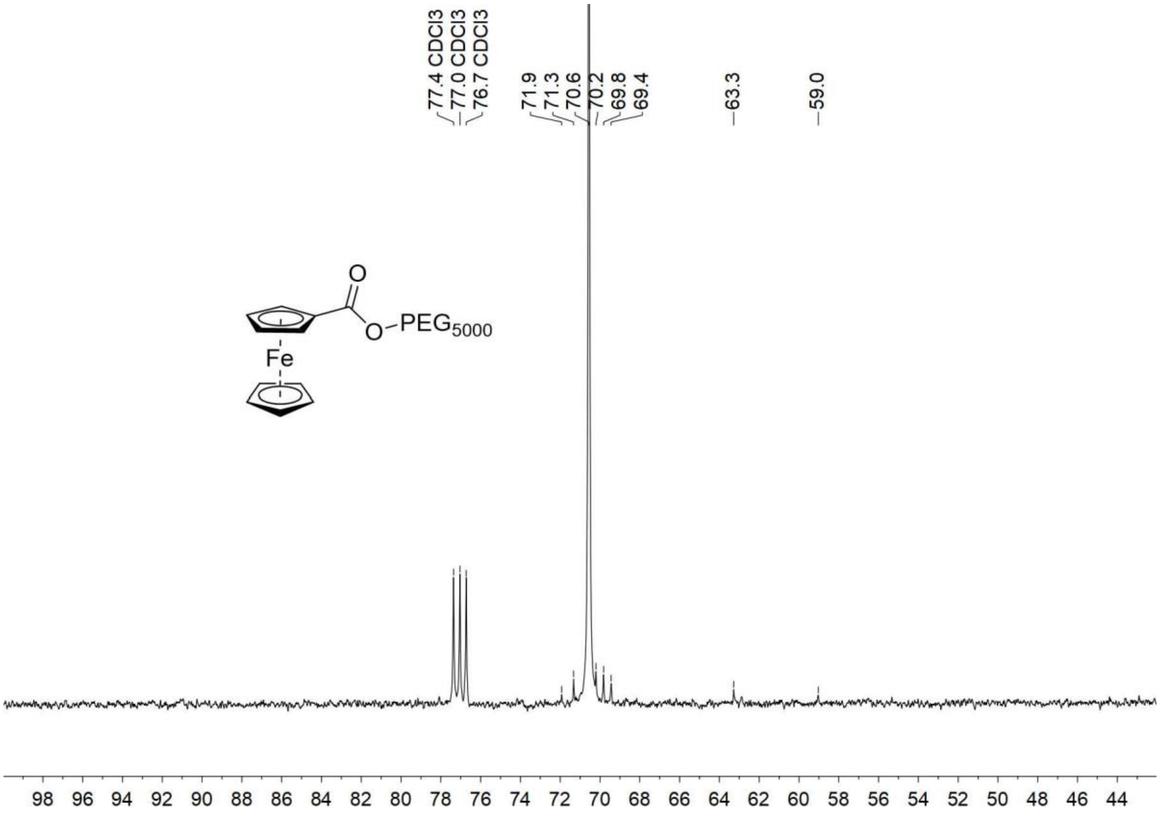

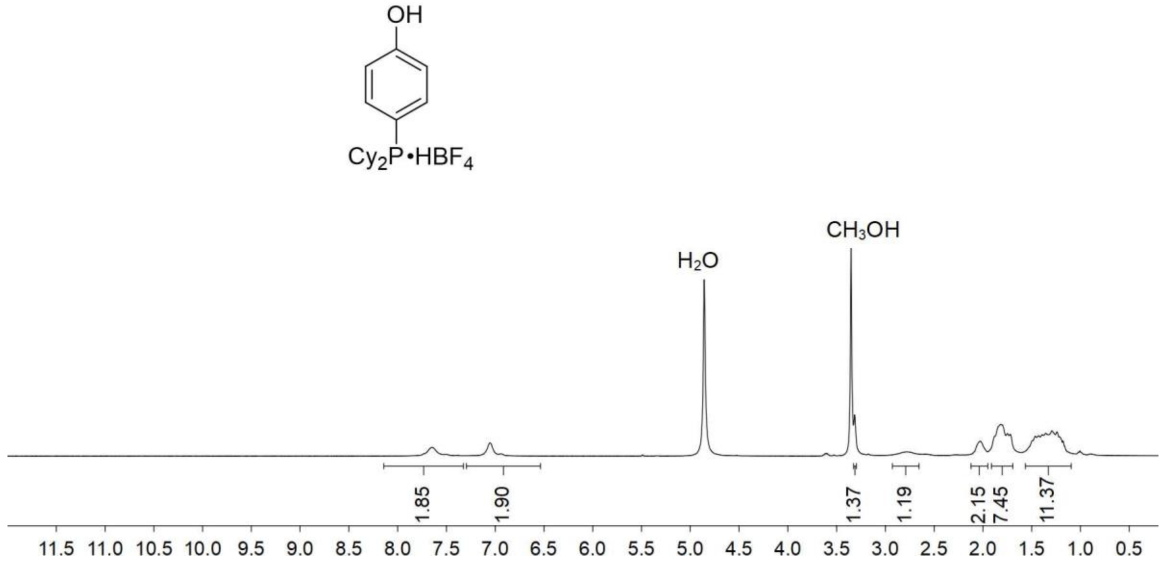
OH
Cy2P•HBF4
H2O
CH3OH
1.85
1.90
1.37
1.19
2.15
7.45
11.37
11.5 11.0 10.5 10.0 9.5 9.0 8.5 8.0 7.5 7.0 6.5 6.0 5.5 5.0 4.5 4.0 3.5 3.0 2.5 2.0 1.5 1.0 0.5

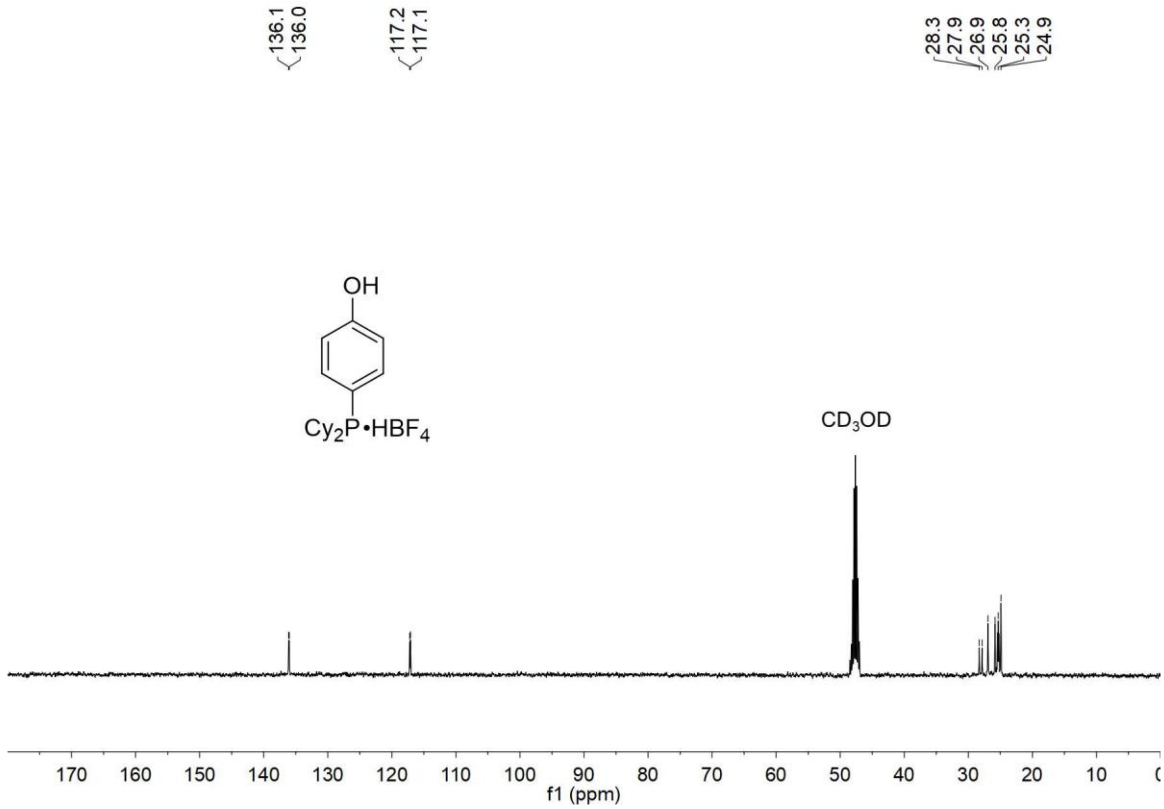
136.1
136.0
117.2
117.1
28.3
27.9
26.9
25.8
25.3
24.9
OH
Cy2P•HBF4
CD3OD
170 160 150 140 130 120 110 100 90 80 70 60 50 40 30 20 10
f1 (ppm)

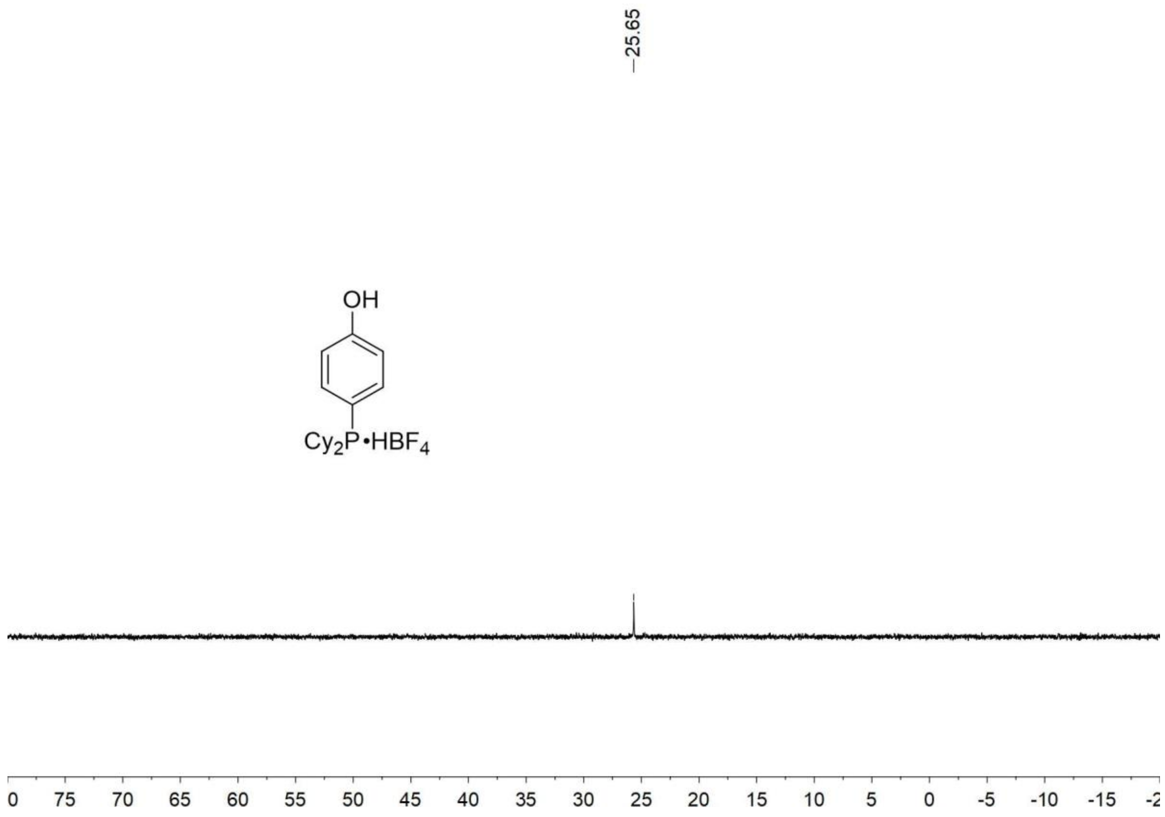

—25.65
OH
Cy2P•HBF4
0 75 70 65 60 55 50 45 40 35 30 25 20 15 10 5 0 -5 -10 -15

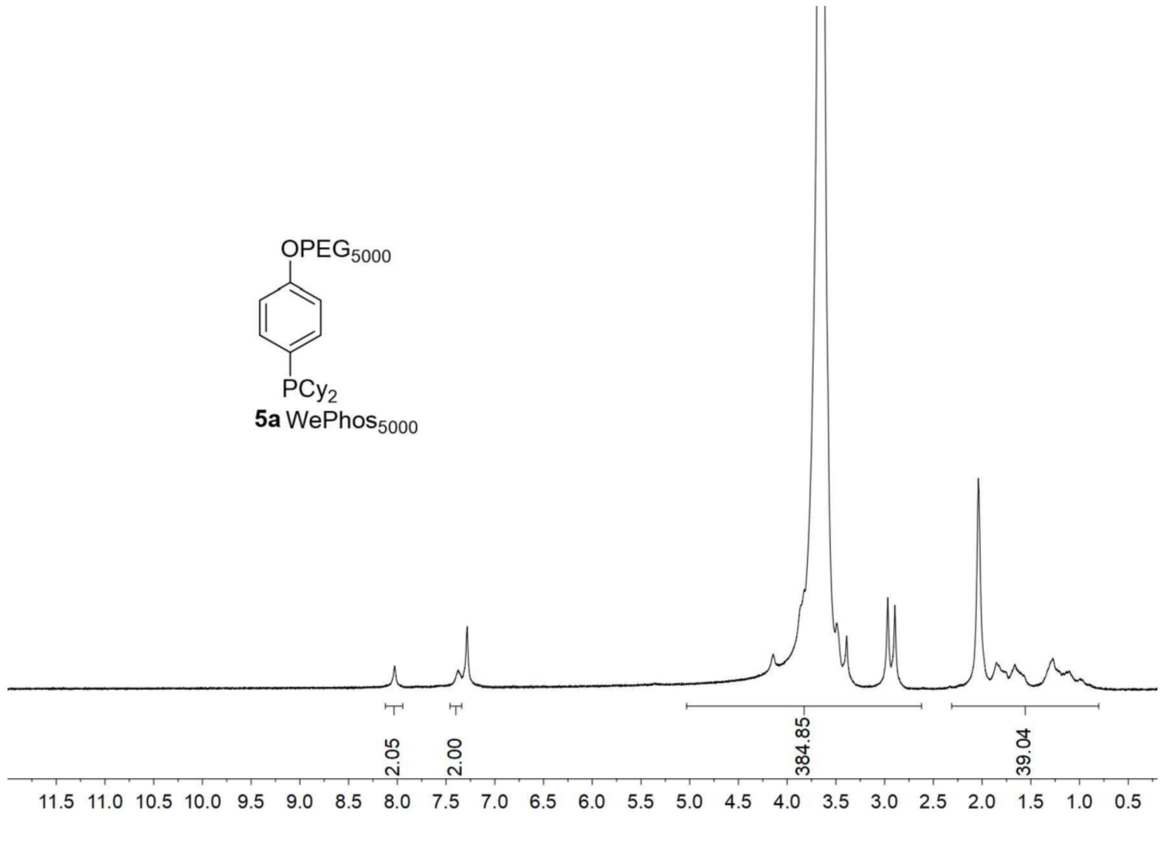

OPEG5000
PCy2
5a WePhos5000
2.05
2.00
384.85
39.04
11.5 11.0 10.5 10.0 9.5 9.0 8.5 8.0 7.5 7.0 6.5 6.0 5.5 5.0 4.5 4.0 3.5 3.0 2.5 2.0 1.5 1.0 0.5

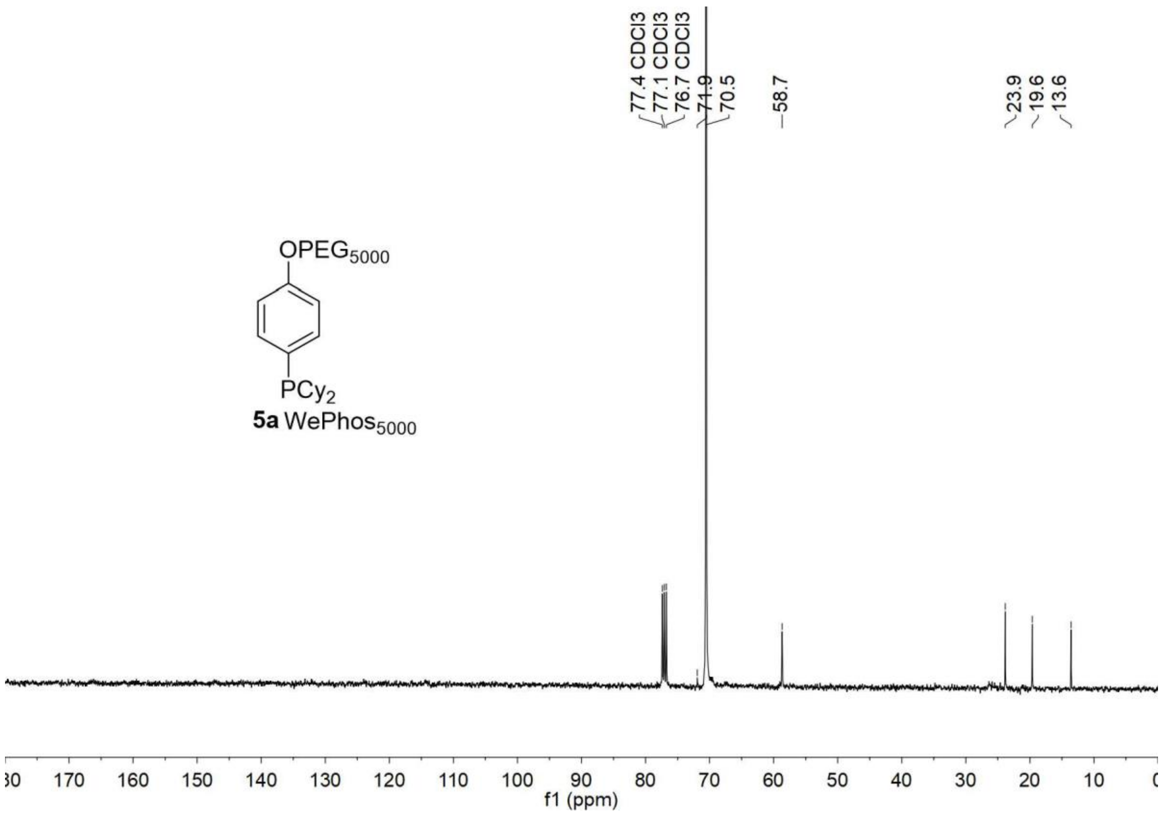
77.4 CDCl3
77.1 CDCl3
76.7 CDCl3
71.9
70.5
58.7
23.9
19.6
13.6
OPEG5000
PCy2
5a WePhos5000
170 160 150 140 130 120 110 100 90 80 70 60 50 40 30 20 10
f1 (ppm)

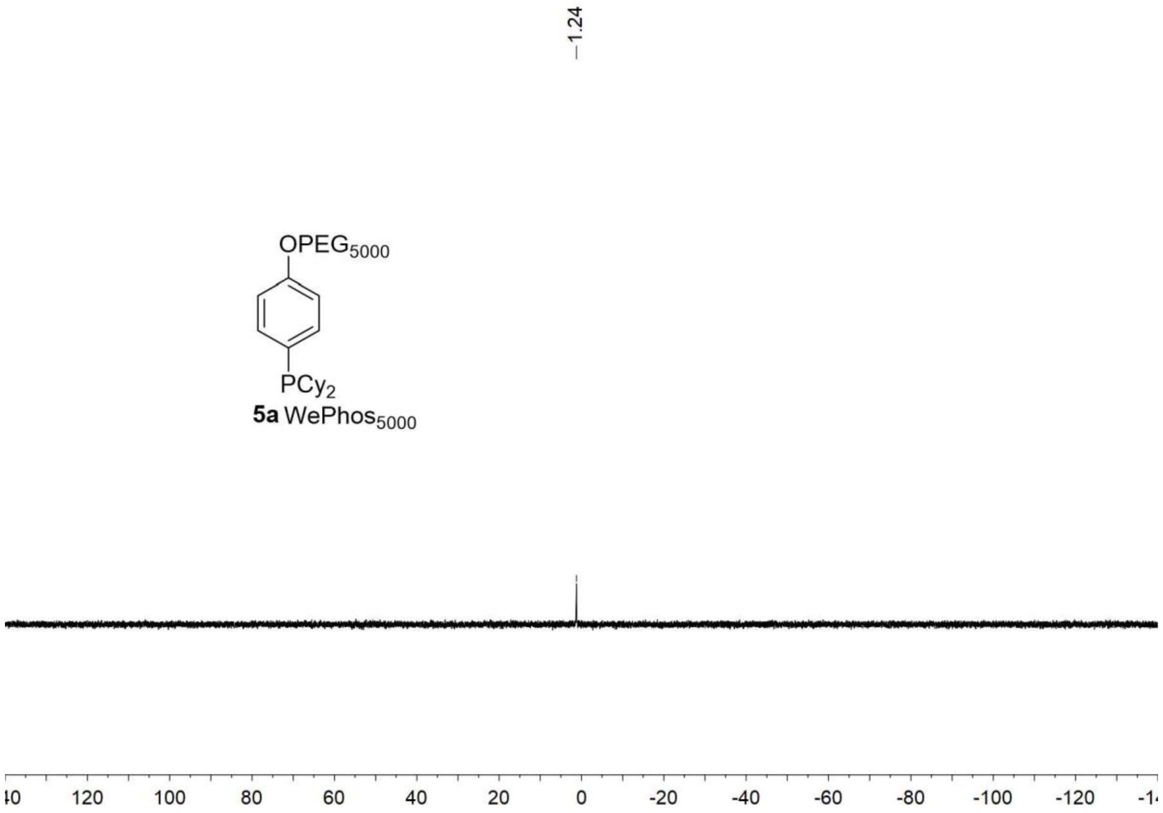
1.24
OPEG5000
PCy2
5a WePhos5000
120 100 80 60 40 20 0 -20 -40 -60 -80 -100 -120

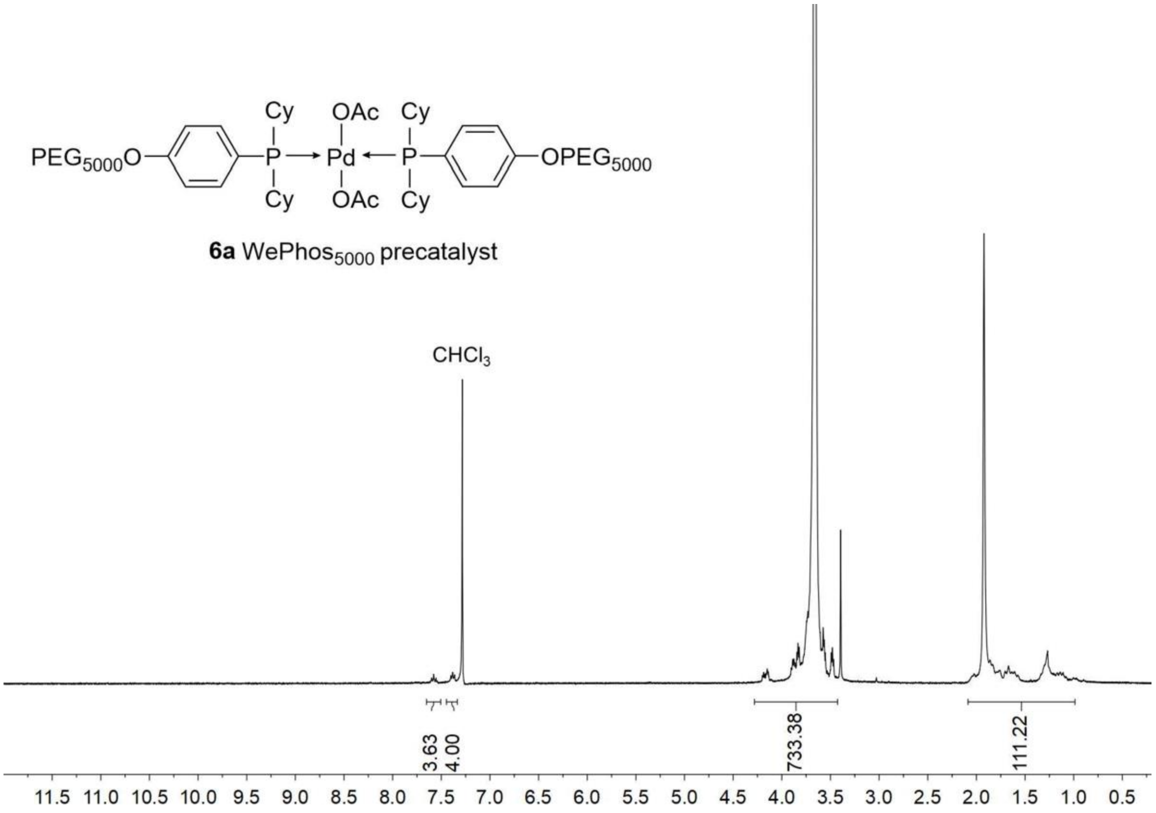

Cy
OAc
Cy
PEG5000O
P
Pd
P
OPEG5000
Cy
OAc
Cy
6a WePhos5000 precatalyst
CHCl3
3.63
4.00
733.38
111.22
11.5 11.0 10.5 10.0 9.5 9.0 8.5 8.0 7.5 7.0 6.5 6.0 5.5 5.0 4.5 4.0 3.5 3.0 2.5 2.0 1.5 1.0 0.5

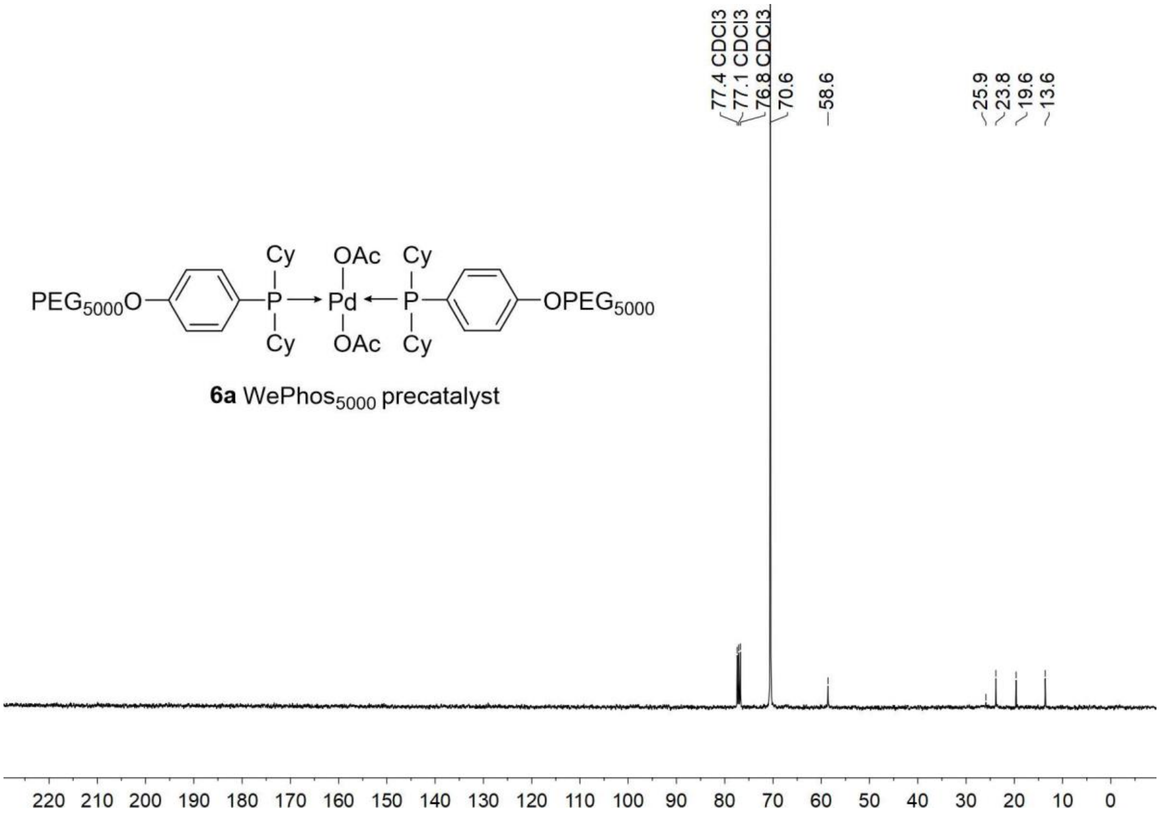

77.4 CDCl3
77.1 CDCl3
76.8 CDCl3
70.6
58.6
25.9
23.8
19.6
13.6
Cy
OAc
Cy
PEG5000O
P
Pd
P
OPEG5000
Cy
OAc
Cy
6a WePhos5000 precatalyst
220 210 200 190 180 170 160 150 140 130 120 110 100 90 80 70 60 50 40 30 20 10 0

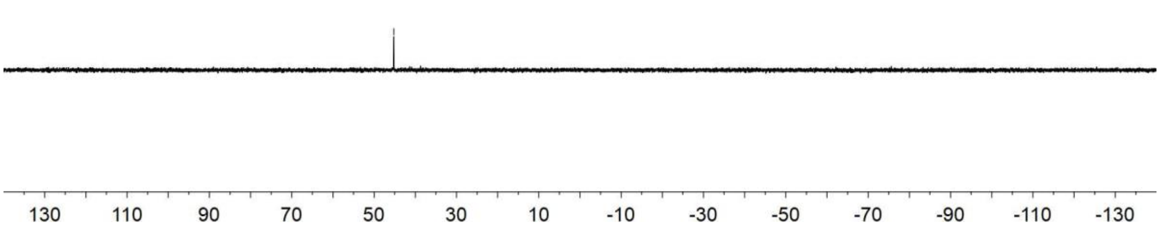

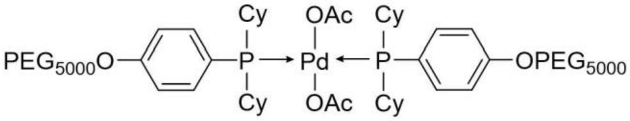

6a WePhos$_{5000}$ precatalyst

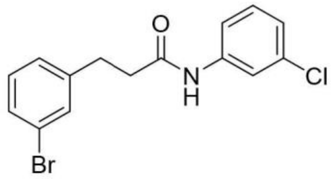

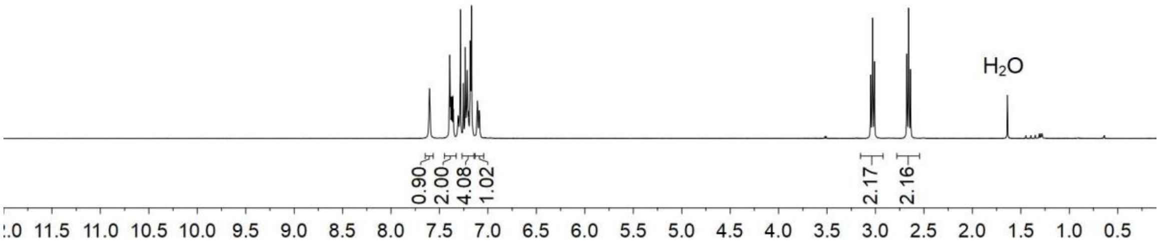

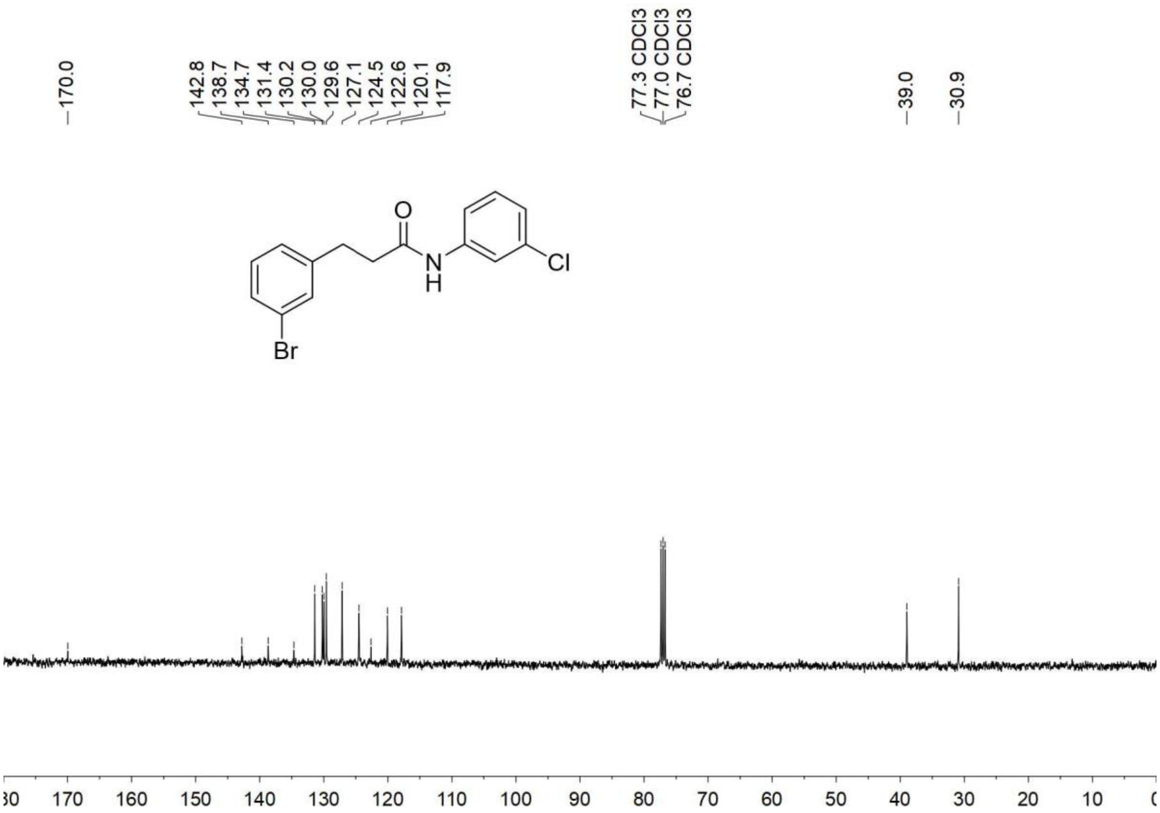

170.0
142.8
138.7
134.7
131.4
130.2
130.0
129.6
127.1
124.5
122.6
120.1
117.9
77.3 CDCl3
77.0 CDCl3
76.7 CDCl3
39.0
30.9
O
N
H
Cl
Br

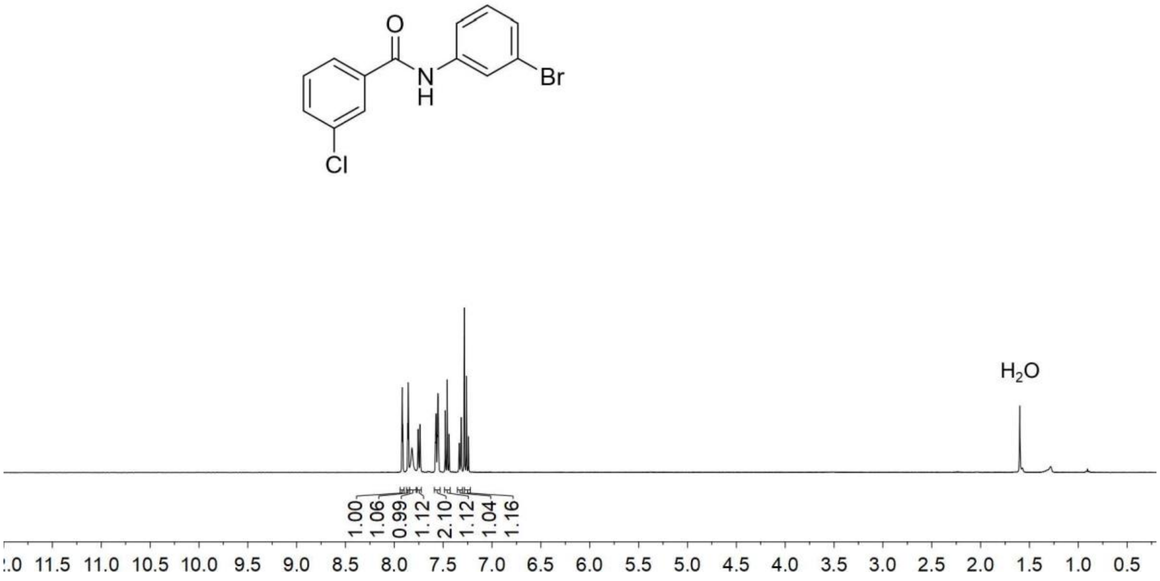

O
N
H
Br
Cl
H2O
1.00
1.06
0.99
1.12
2.10
1.12
1.04
1.16

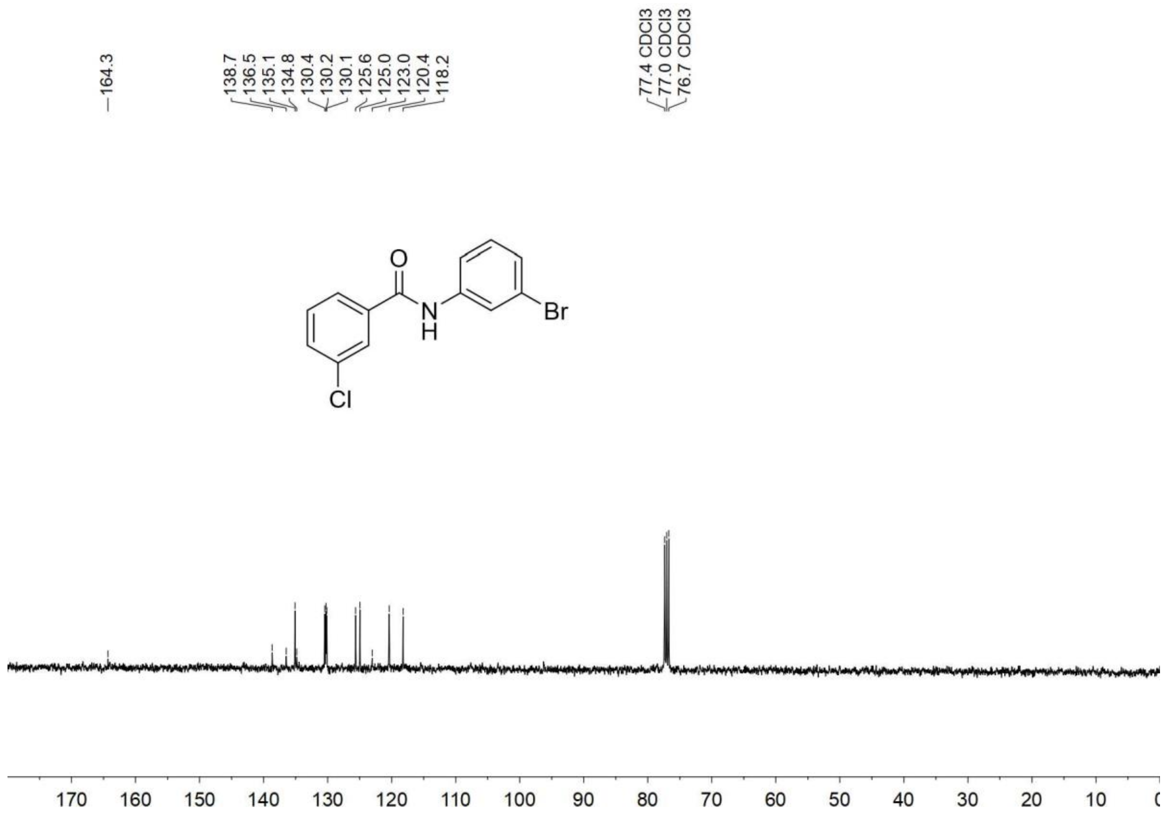
164.3
138.7
136.5
135.1
134.8
130.4
130.2
130.1
125.6
125.0
123.0
120.4
118.2
77.4 CDCl3
77.0 CDCl3
76.7 CDCl3
O
N
H
Br
Cl
170 160 150 140 130 120 110 100 90 80 70 60 50 40 30 20 10

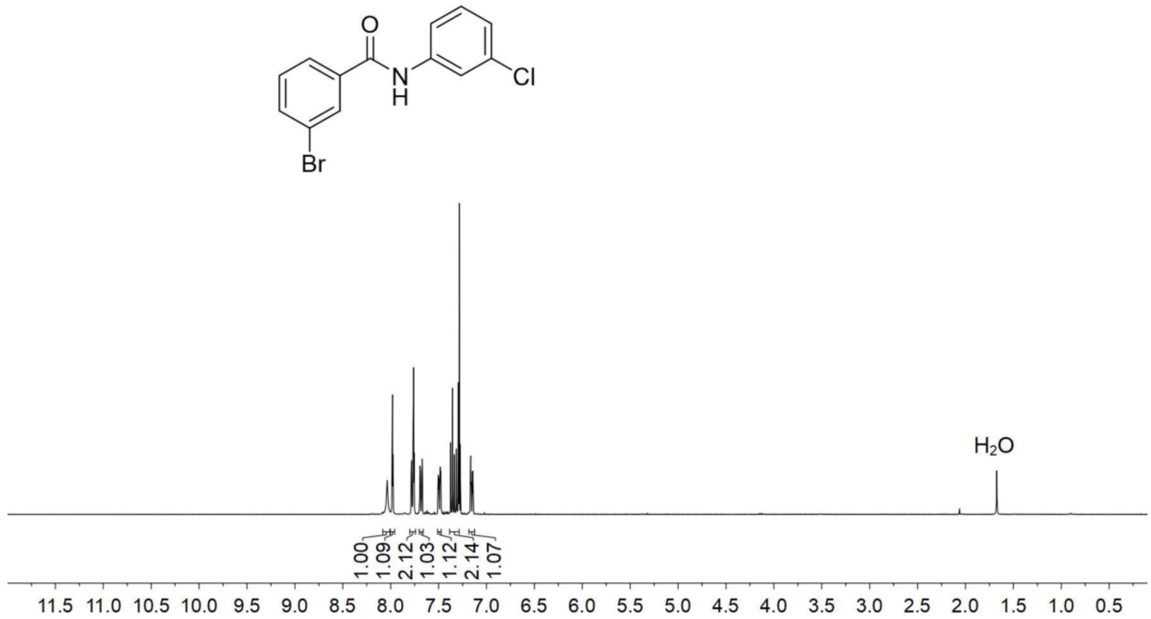
O
N
H
Cl
Br
H2O
1.00
1.09
2.12
1.03
1.12
2.14
1.07
11.5 11.0 10.5 10.0 9.5 9.0 8.5 8.0 7.5 7.0 6.5 6.0 5.5 5.0 4.5 4.0 3.5 3.0 2.5 2.0 1.5 1.0 0.5

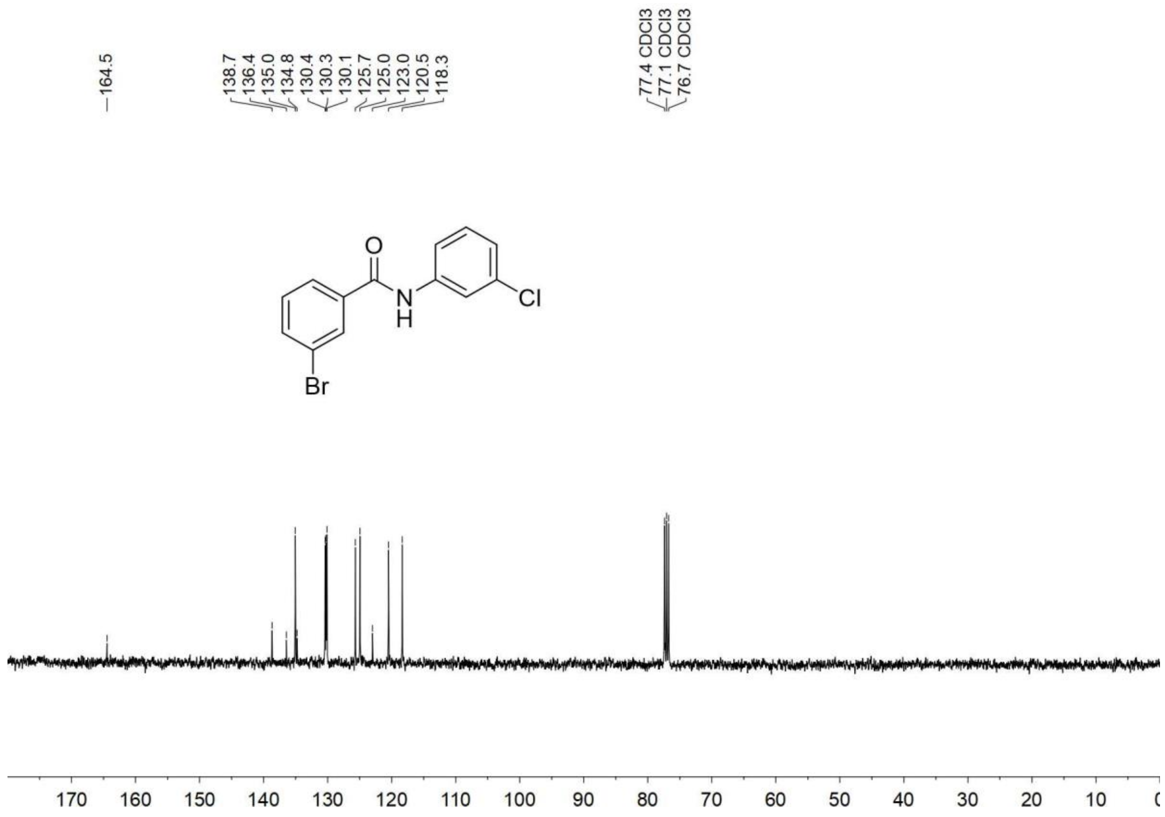

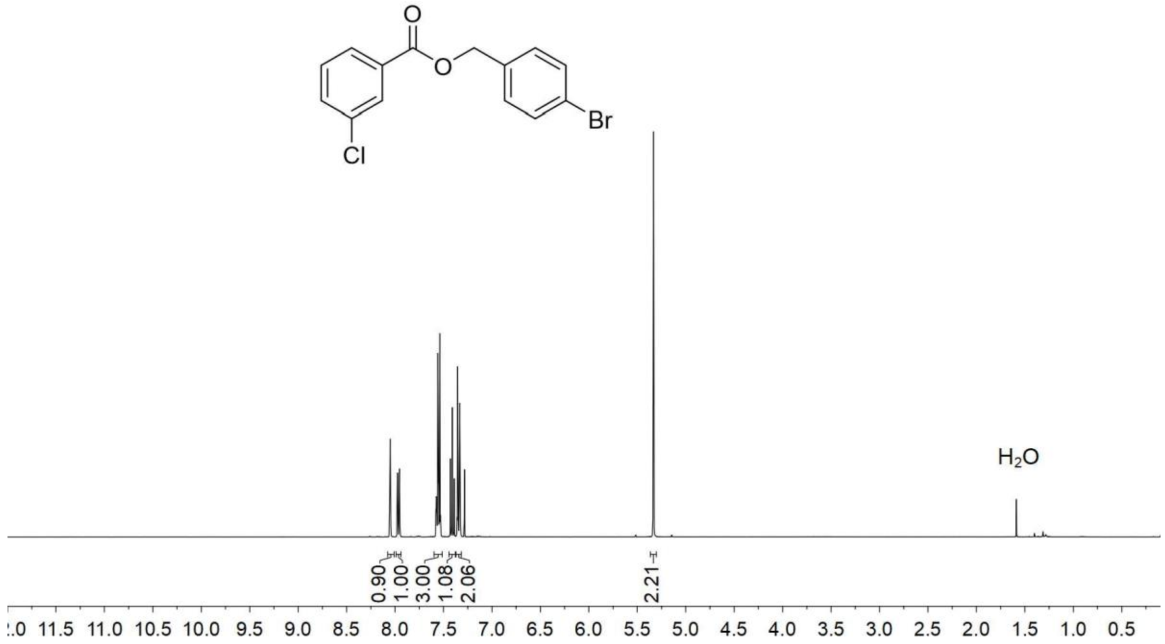

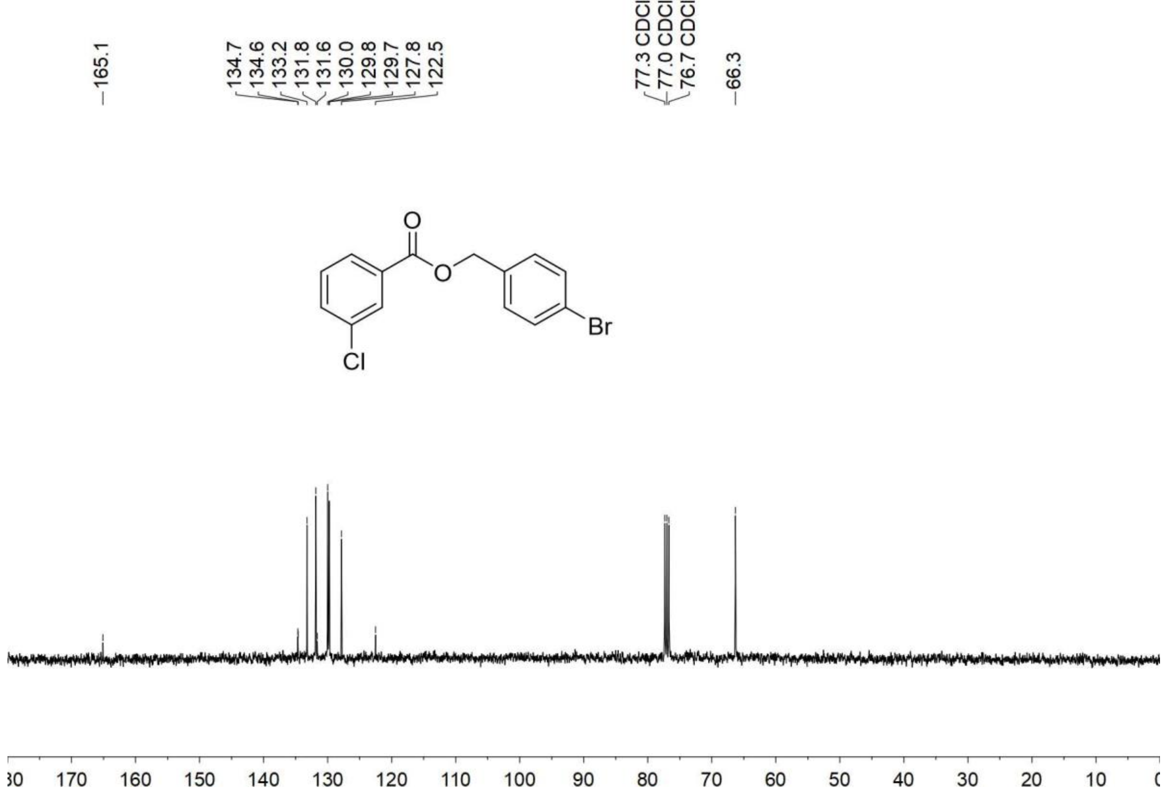

165.1
134.7
134.6
133.2
131.8
131.6
130.0
129.8
129.7
127.8
122.5
77.3 CDCl3
77.0 CDCl3
76.7 CDCl3
66.3
O
O
Cl
Br
170 160 150 140 130 120 110 100 90 80 70 60 50 40 30 20 10

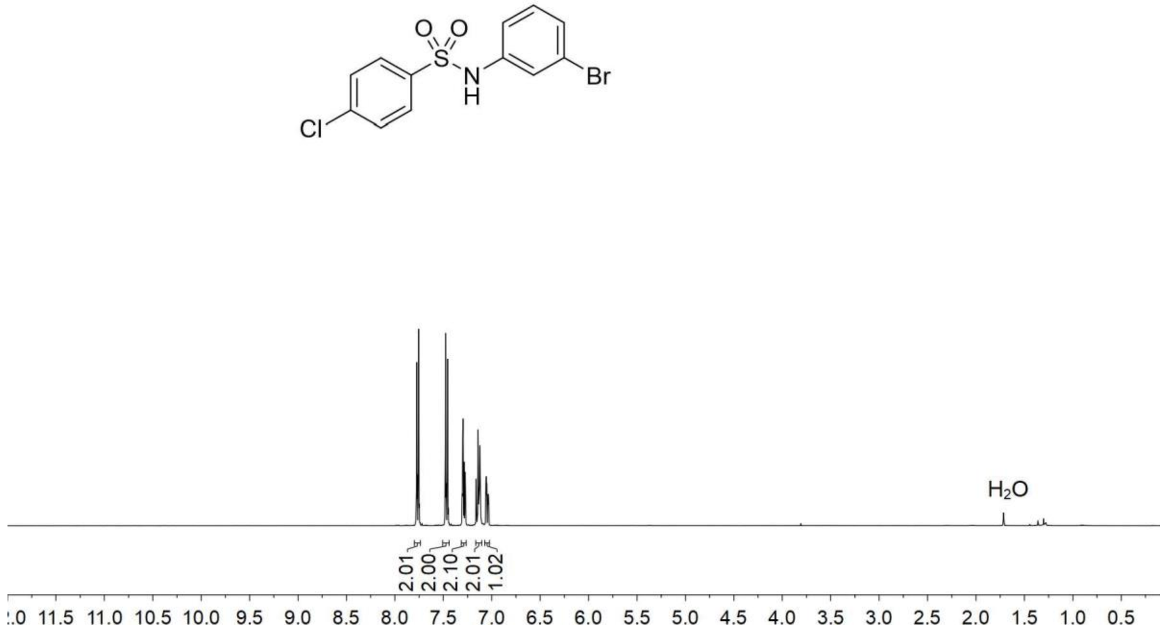

O O
S
N
H
Cl
Br
H2O
2.01
2.00
2.10
2.01
1.02
11.5 11.0 10.5 10.0 9.5 9.0 8.5 8.0 7.5 7.0 6.5 6.0 5.5 5.0 4.5 4.0 3.5 3.0 2.5 2.0 1.5 1.0 0.5

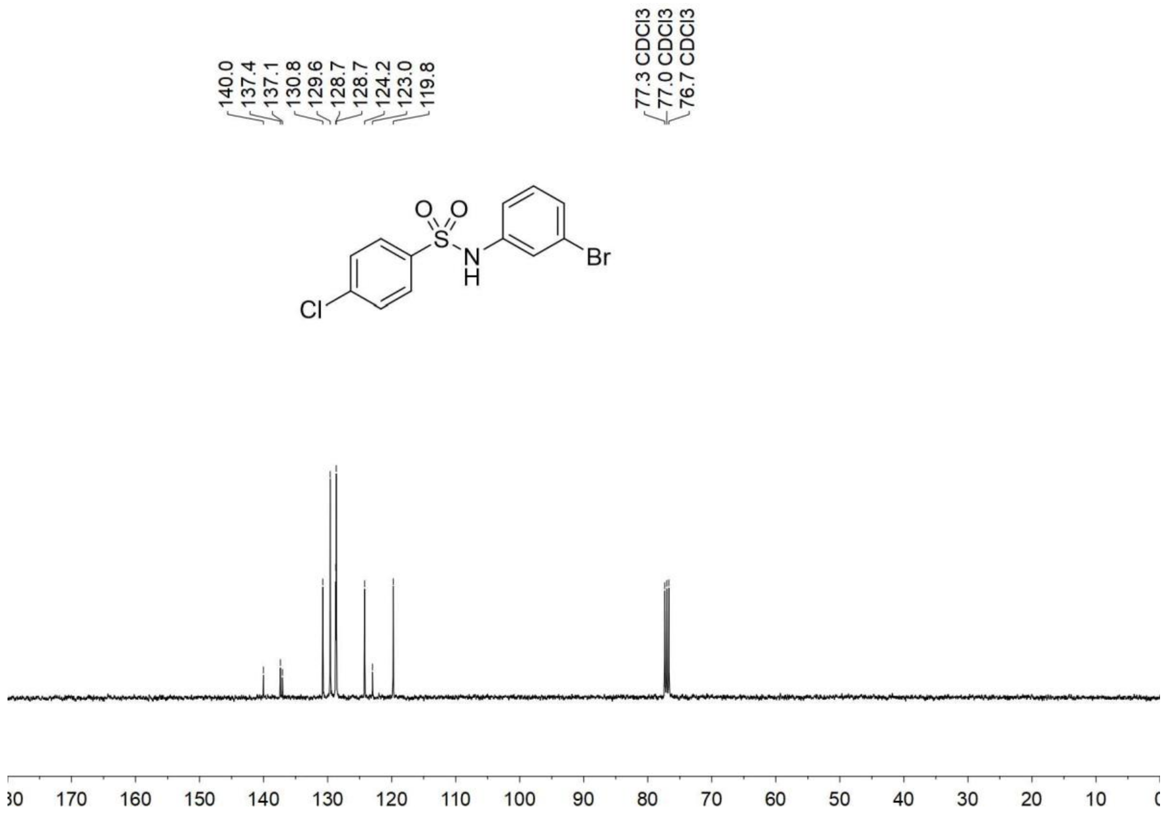
140.0
137.4
137.1
130.8
129.6
128.7
128.7
124.2
123.0
119.8
77.3 CDCl3
77.0 CDCl3
76.7 CDCl3
Cl
Br
170
160
150
140
130
120
110
100
90
80
70
60
50
40
30
20
10

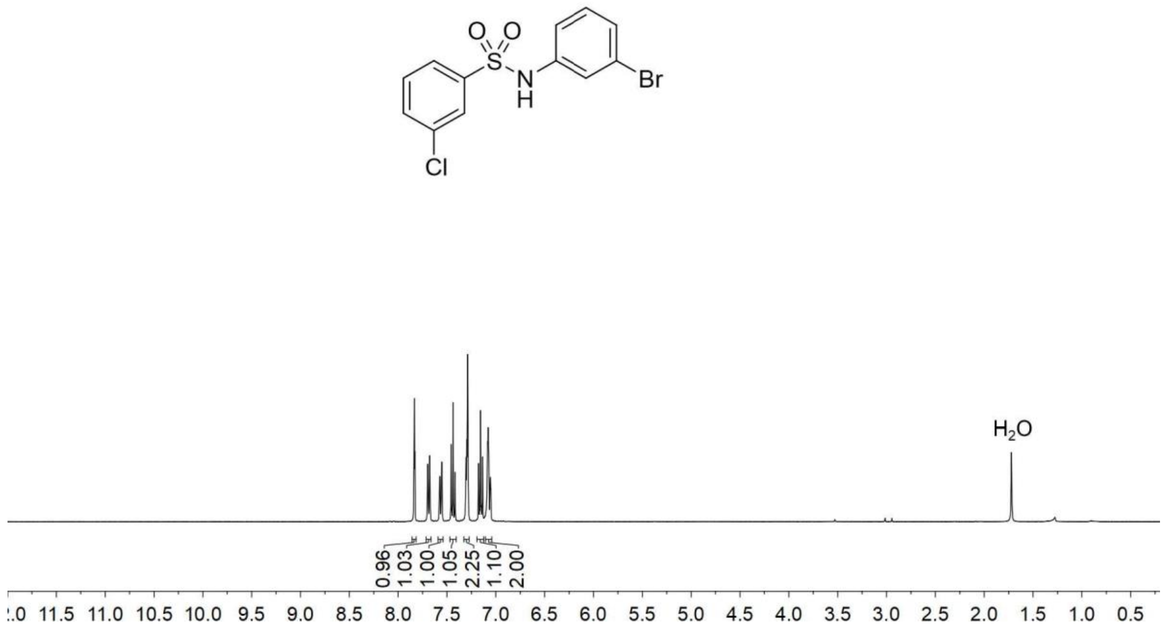
Cl
Br
H2O
0.96
1.03
1.00
1.05
2.25
1.10
2.00
11.5
11.0
10.5
10.0
9.5
9.0
8.5
8.0
7.5
7.0
6.5
6.0
5.5
5.0
4.5
4.0
3.5
3.0
2.5
2.0
1.5
1.0
0.5

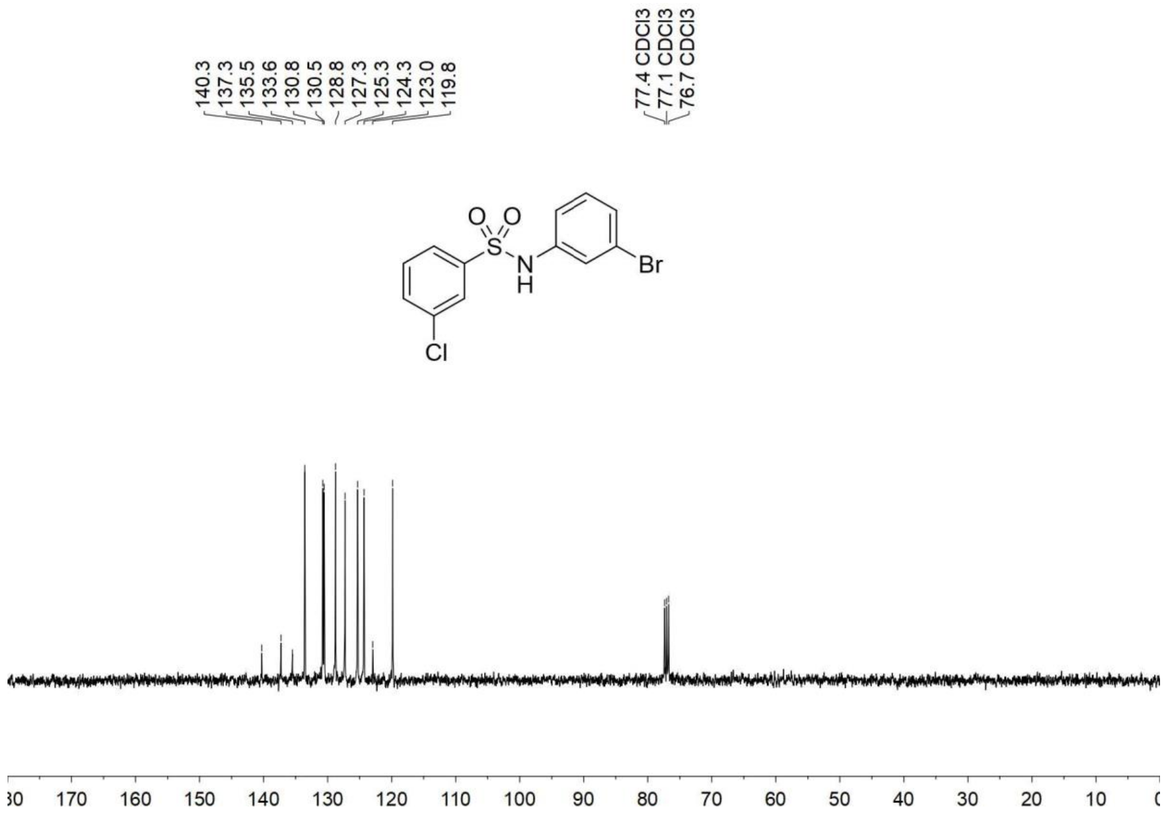
140.3
137.3
135.5
133.6
130.8
130.5
128.8
127.3
125.3
124.3
123.0
119.8
77.4 CDCl3
77.1 CDCl3
76.7 CDCl3
O
O
S
N
H
Br
Cl
170 160 150 140 130 120 110 100 90 80 70 60 50 40 30 20 10

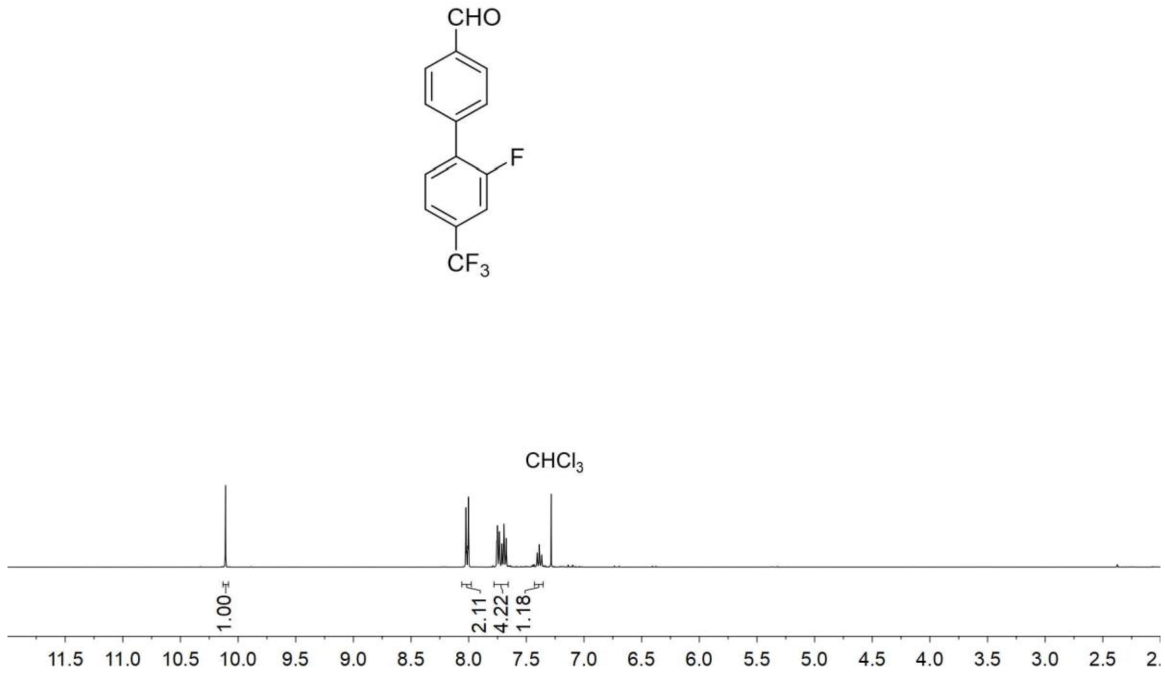
CHO
F
CF_3
$CHCl_3$
1.00
2.11
4.22
1.18
11.5 11.0 10.5 10.0 9.5 9.0 8.5 8.0 7.5 7.0 6.5 6.0 5.5 5.0 4.5 4.0 3.5 3.0 2.5

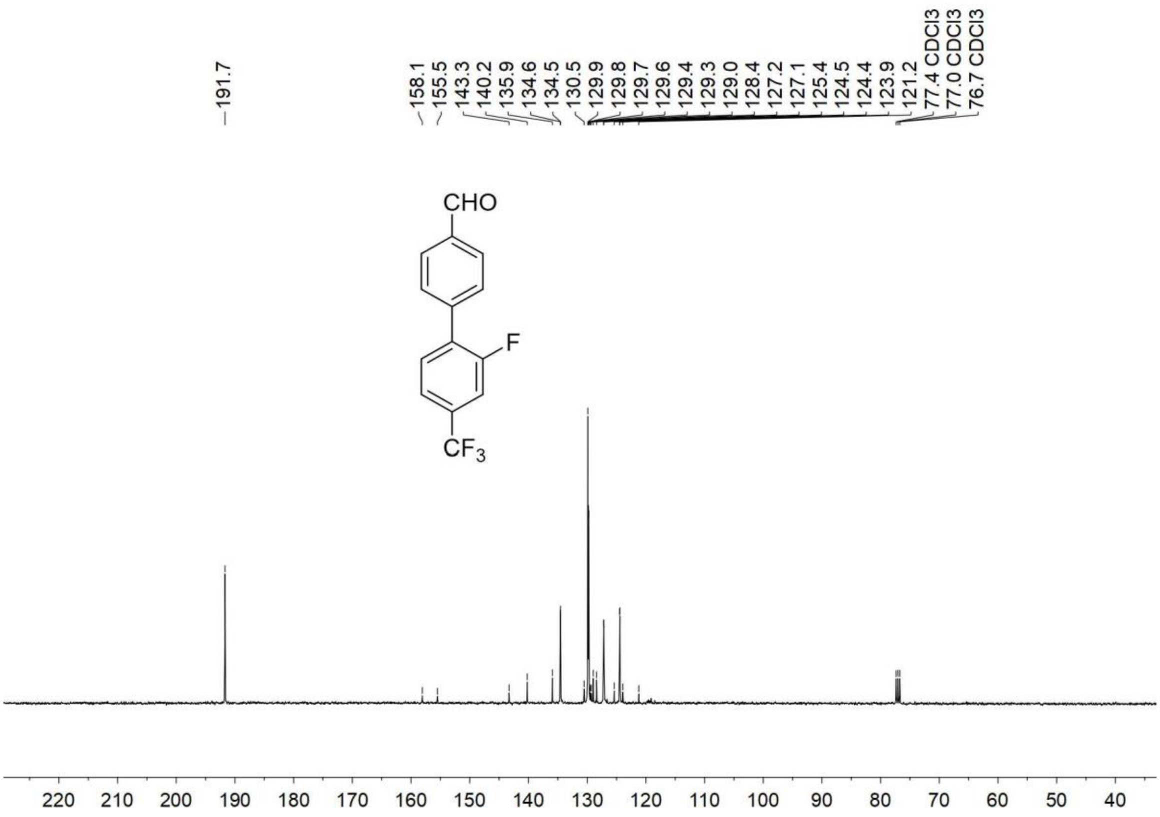

191.7
158.1
155.5
143.3
140.2
135.9
134.6
134.5
130.5
129.9
129.8
129.7
129.6
129.4
129.3
129.0
128.4
127.2
127.1
125.4
124.5
124.4
123.9
121.2
77.4 CDCl3
77.0 CDCl3
76.7 CDCl3
CHO
F
CF_3
220 210 200 190 180 170 160 150 140 130 120 110 100 90 80 70 60 50 40

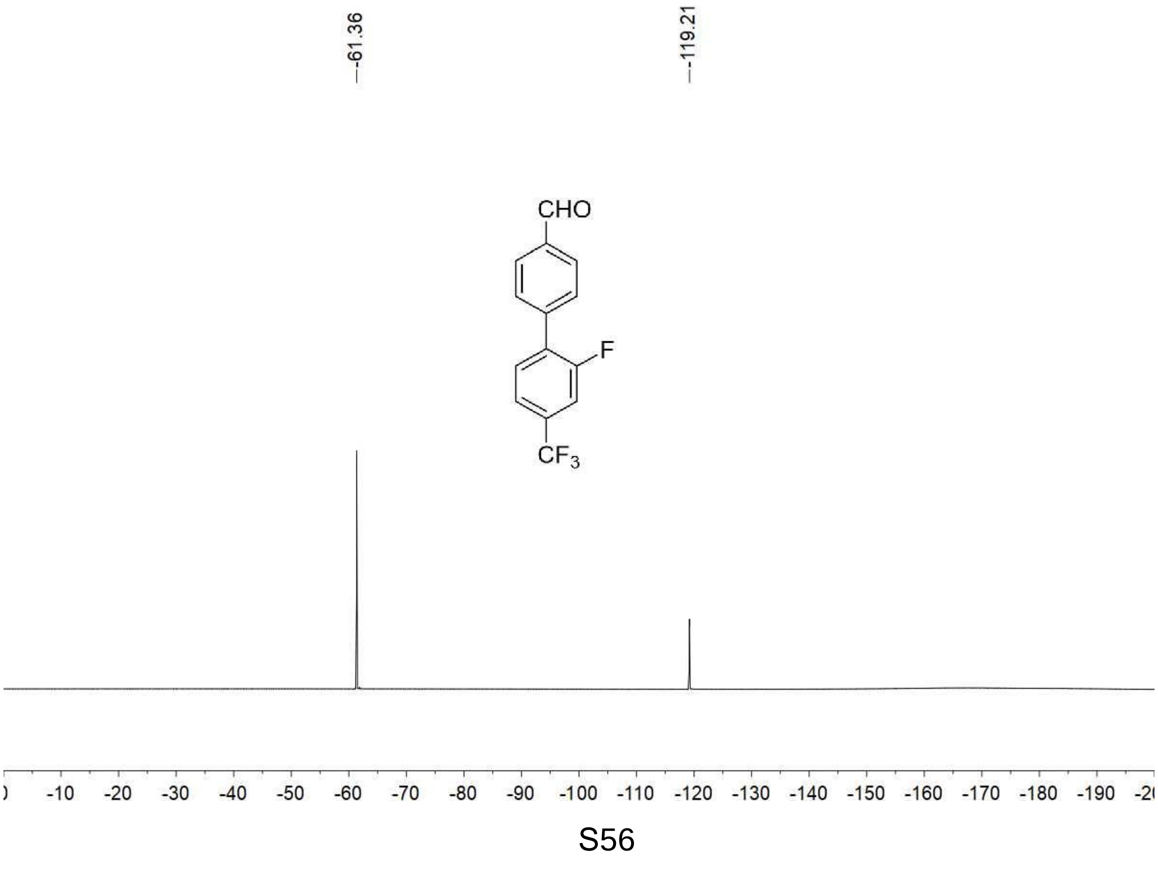

-61.36
-119.21
CHO
F
CF_3
-10 -20 -30 -40 -50 -60 -70 -80 -90 -100 -110 -120 -130 -140 -150 -160 -170 -180 -190

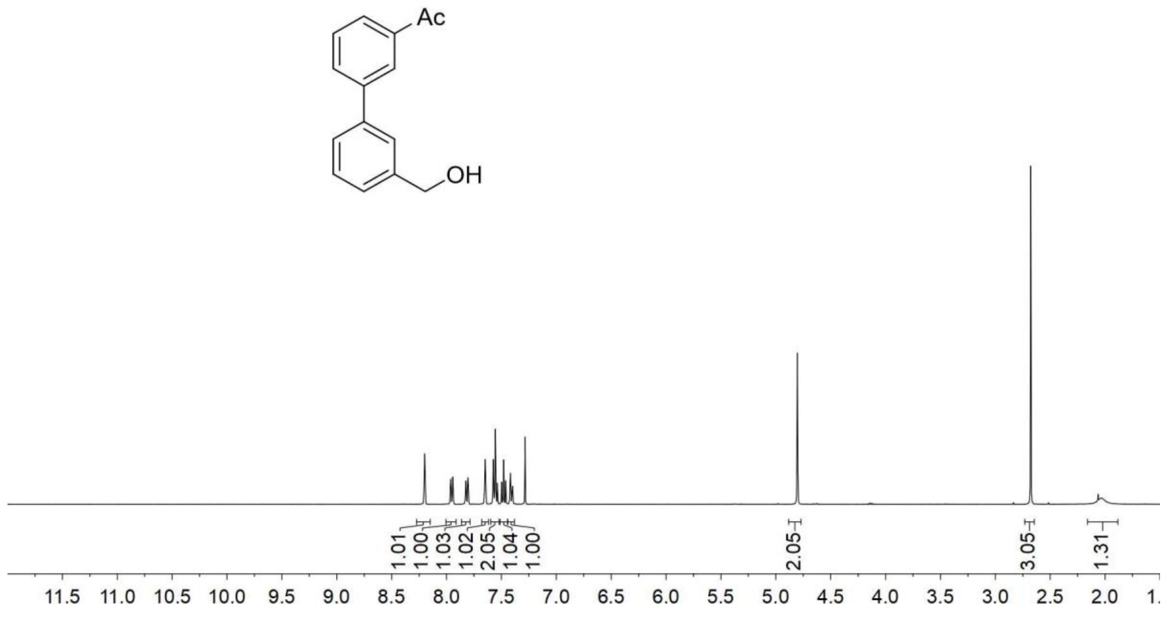

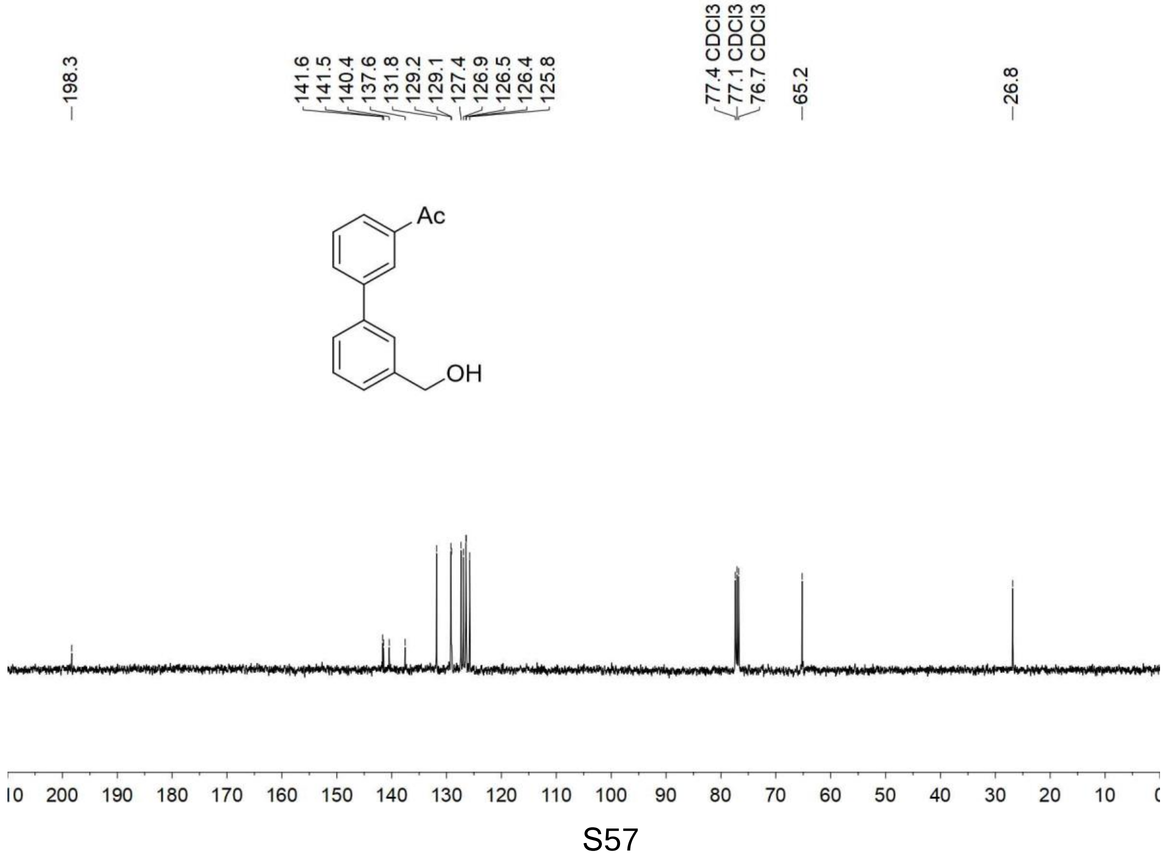

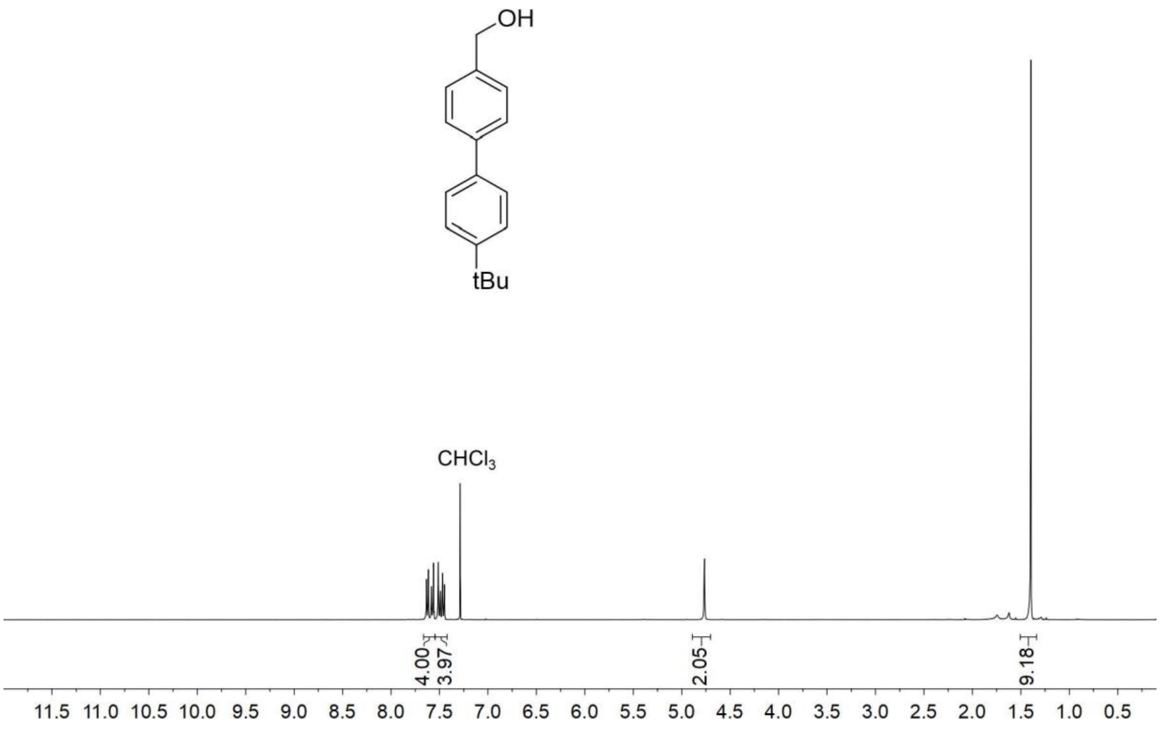

OH
tBu
CHCl3
4.00
3.97
2.05
9.18
11.5 11.0 10.5 10.0 9.5 9.0 8.5 8.0 7.5 7.0 6.5 6.0 5.5 5.0 4.5 4.0 3.5 3.0 2.5 2.0 1.5 1.0 0.5

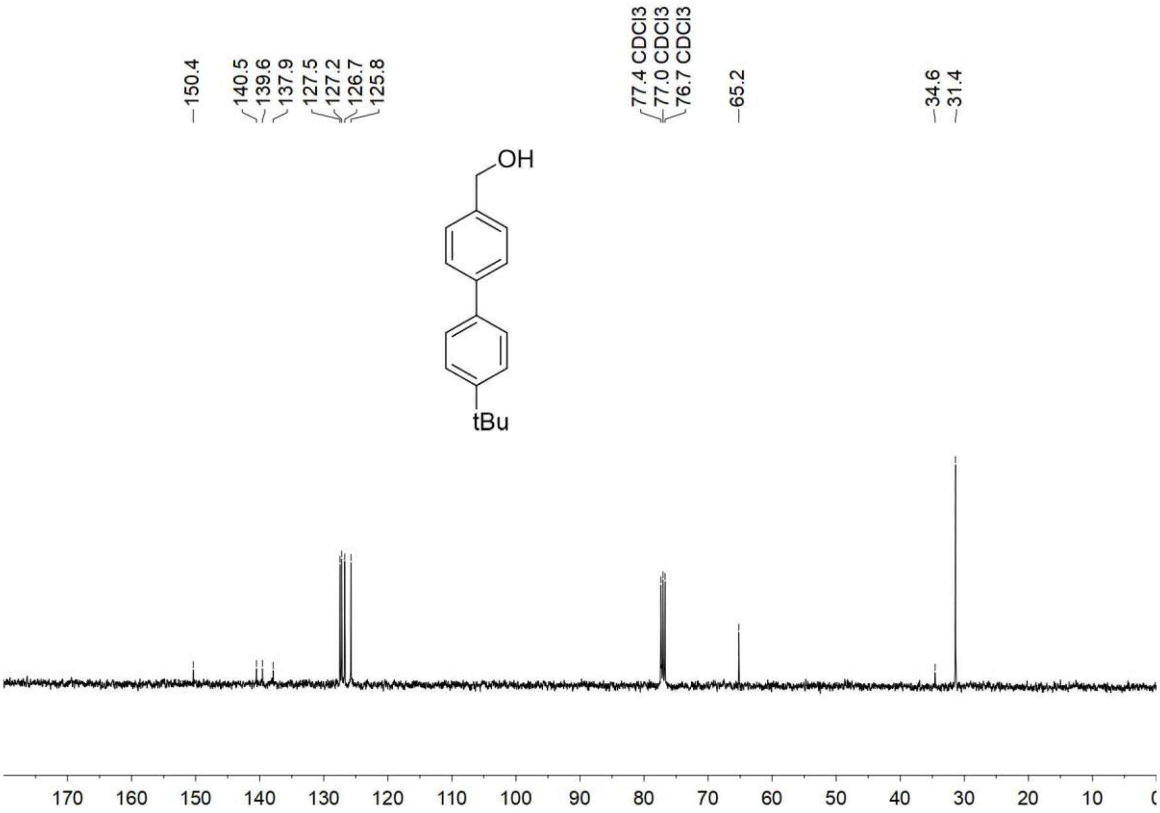

150.4
140.5
139.6
137.9
127.5
127.2
126.7
125.8
77.4 CDCl3
77.0 CDCl3
76.7 CDCl3
65.2
34.6
31.4
OH
tBu
170 160 150 140 130 120 110 100 90 80 70 60 50 40 30 20 10

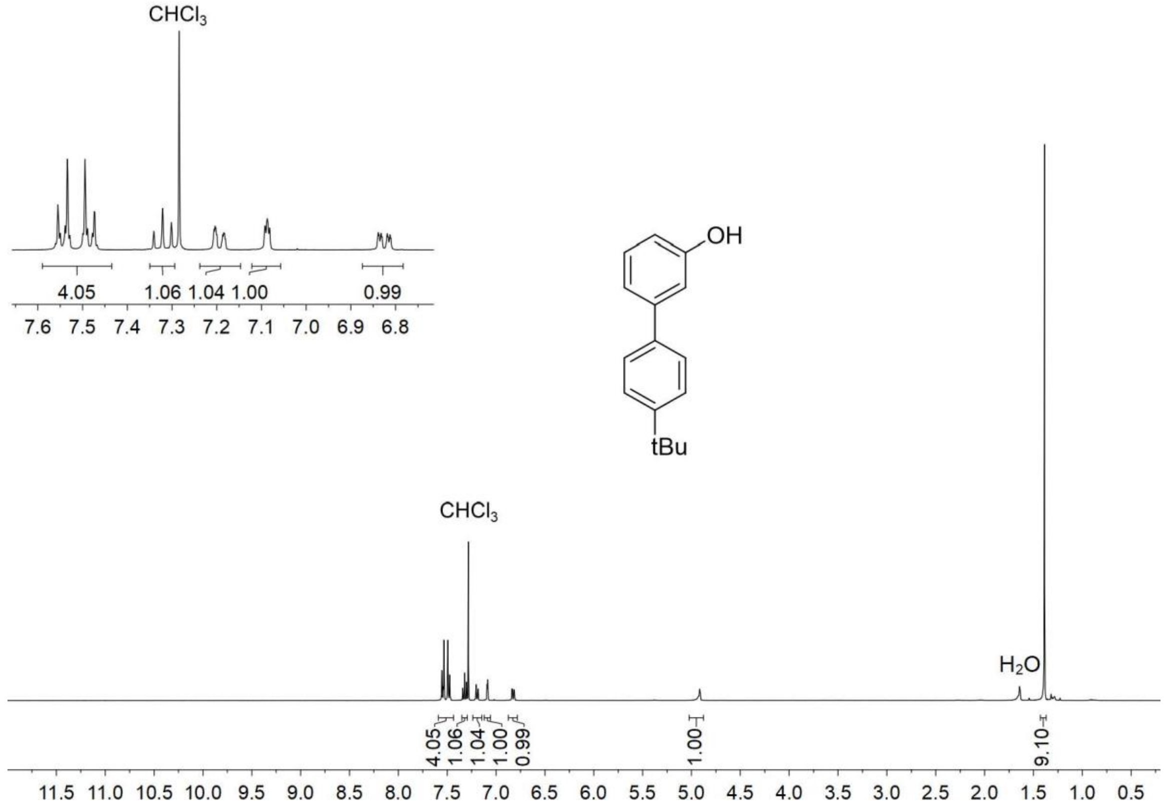
CHCl3
4.05
1.06 1.04 1.00
0.99
7.6 7.5 7.4 7.3 7.2 7.1 7.0 6.9 6.8
OH
tBu
CHCl3
H2O
4.05
1.06
1.04
1.00
0.99
1.00
9.10
11.5 11.0 10.5 10.0 9.5 9.0 8.5 8.0 7.5 7.0 6.5 6.0 5.5 5.0 4.5 4.0 3.5 3.0 2.5 2.0 1.5 1.0 0.5

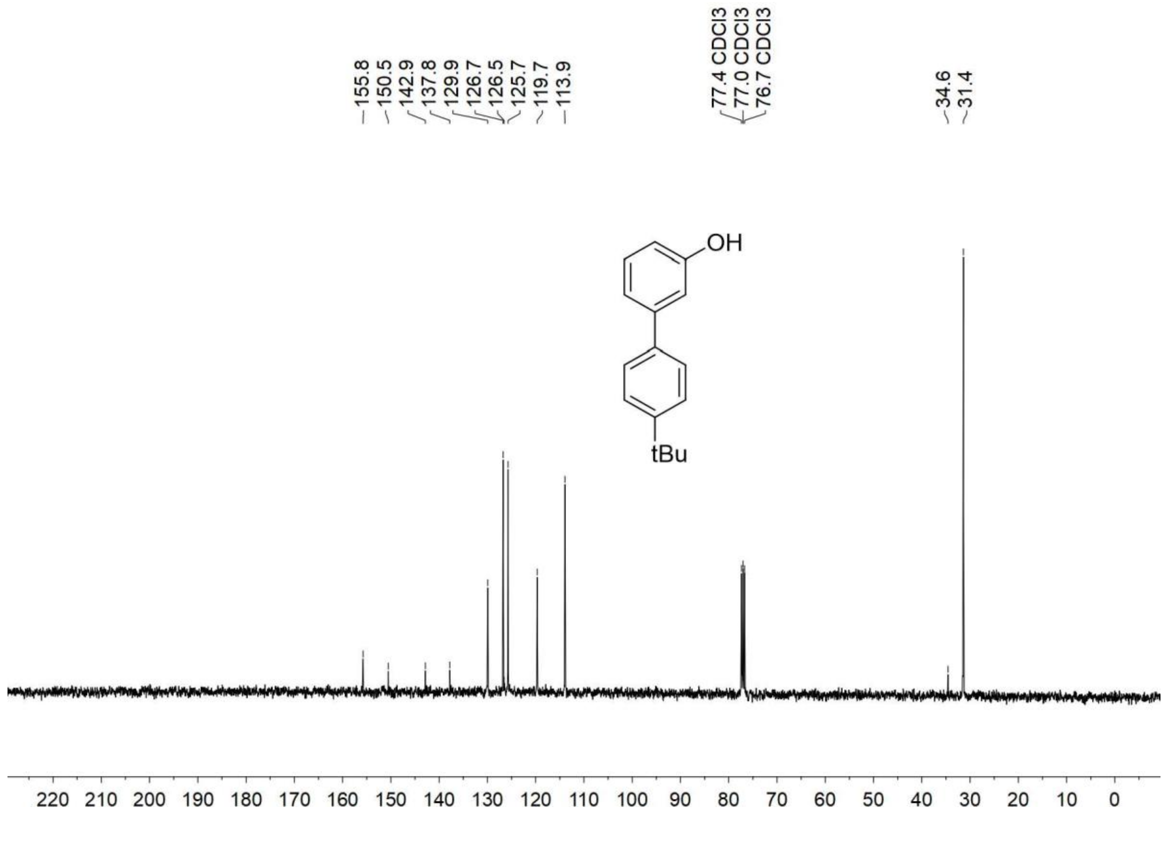
155.8
150.5
142.9
137.8
129.9
126.7
126.5
125.7
119.7
113.9
77.4 CDCl3
77.0 CDCl3
76.7 CDCl3
34.6
31.4
OH
tBu
220 210 200 190 180 170 160 150 140 130 120 110 100 90 80 70 60 50 40 30 20 10 0

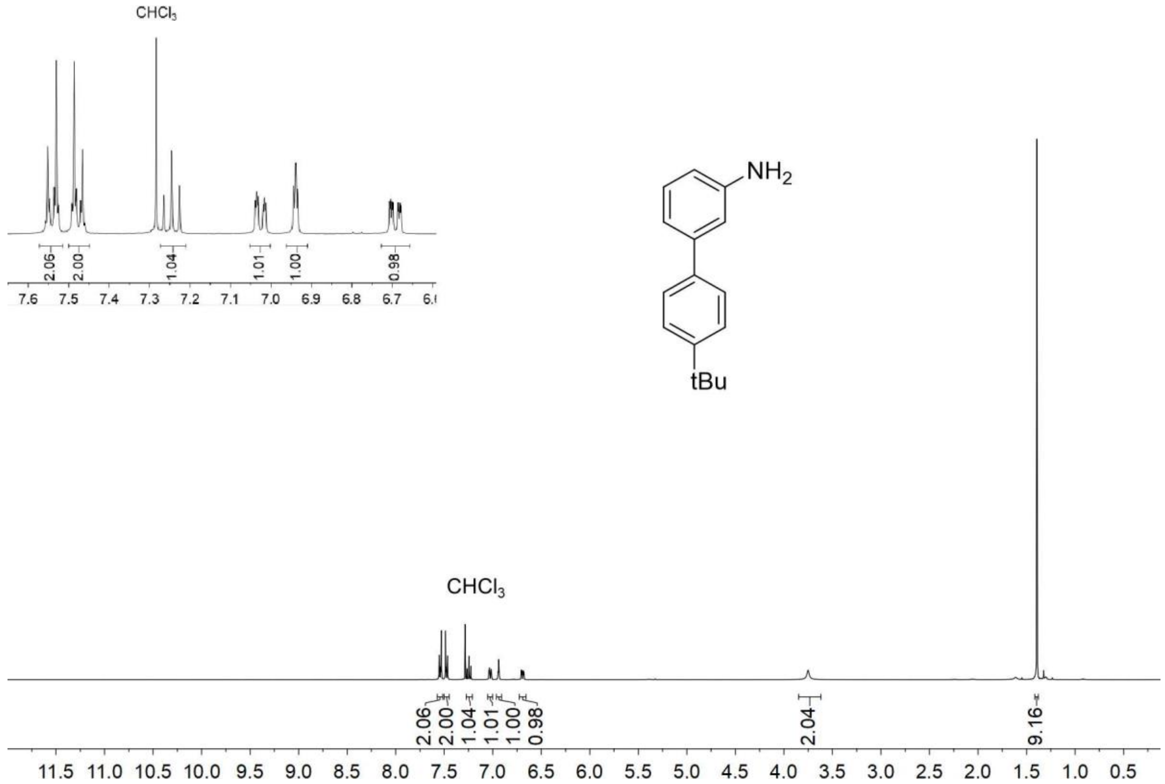
CHCl3
2.06
2.00
1.04
1.01
1.00
0.98
NH2
tBu
2.04
9.16
11.5 11.0 10.5 10.0 9.5 9.0 8.5 8.0 7.5 7.0 6.5 6.0 5.5 5.0 4.5 4.0 3.5 3.0 2.5 2.0 1.5 1.0 0.5

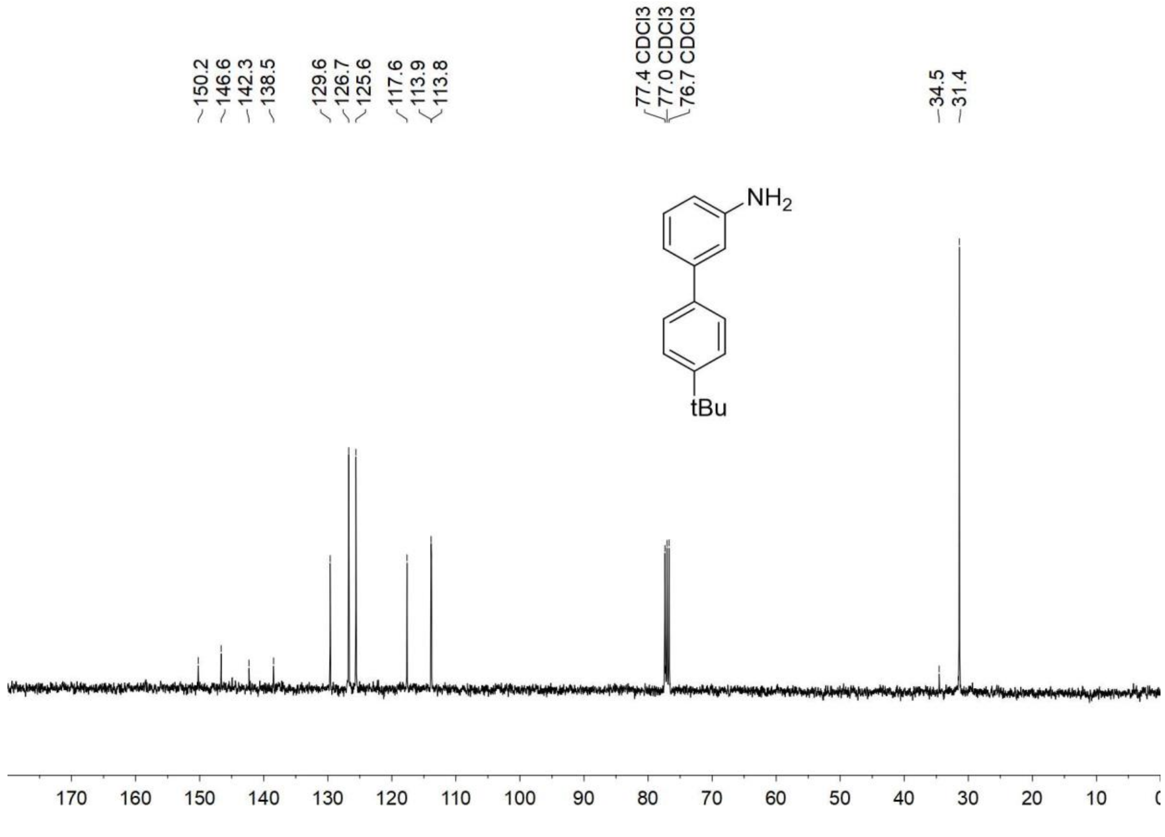
150.2
146.6
142.3
138.5
129.6
126.7
125.6
117.6
113.9
113.8
77.4 CDCl3
77.0 CDCl3
76.7 CDCl3
34.5
31.4
NH2
tBu
170 160 150 140 130 120 110 100 90 80 70 60 50 40 30 20 10

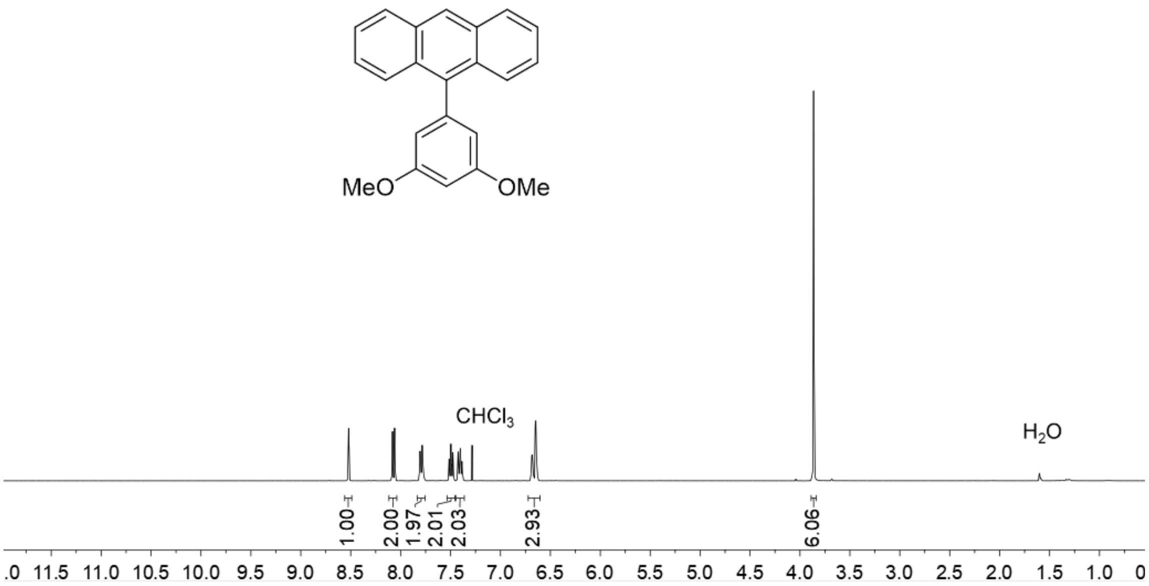
MeO
OMe
CHCl3
H2O
1.00
2.00
1.97
2.01
2.03
2.93
6.06
.0 11.5 11.0 10.5 10.0 9.5 9.0 8.5 8.0 7.5 7.0 6.5 6.0 5.5 5.0 4.5 4.0 3.5 3.0 2.5 2.0 1.5 1.0 0

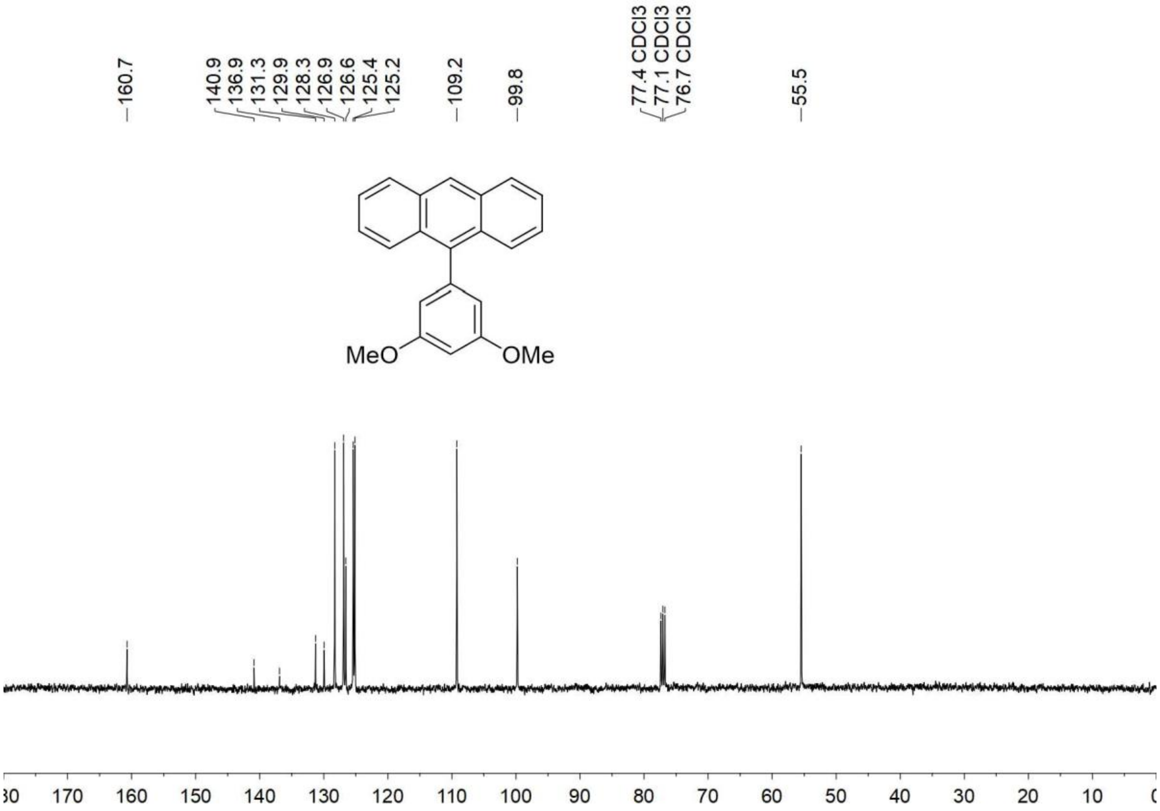
160.7
140.9
136.9
131.3
129.9
128.3
126.9
126.6
125.4
125.2
109.2
99.8
77.4 CDCl3
77.1 CDCl3
76.7 CDCl3
55.5
MeO
OMe
30 170 160 150 140 130 120 110 100 90 80 70 60 50 40 30 20 10 0

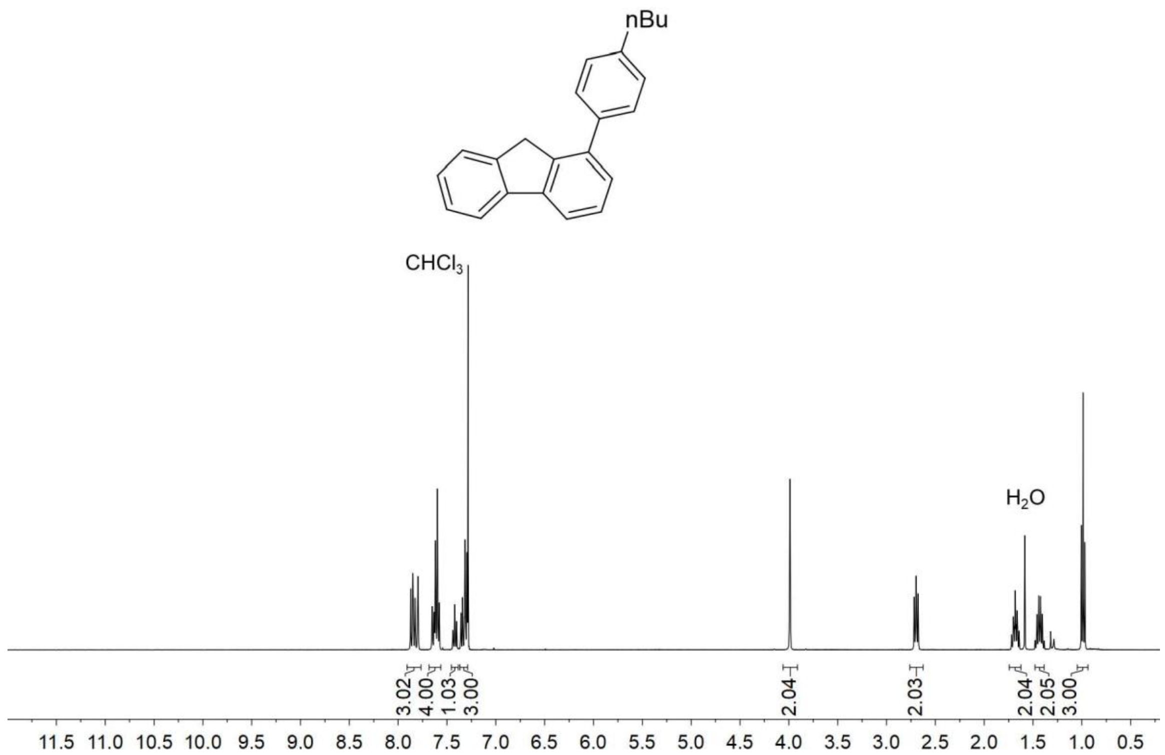
nBu
CHCl3
H2O
3.02
4.00
1.03
3.00
2.04
2.03
2.04
2.05
3.00
11.5 11.0 10.5 10.0 9.5 9.0 8.5 8.0 7.5 7.0 6.5 6.0 5.5 5.0 4.5 4.0 3.5 3.0 2.5 2.0 1.5 1.0 0.5

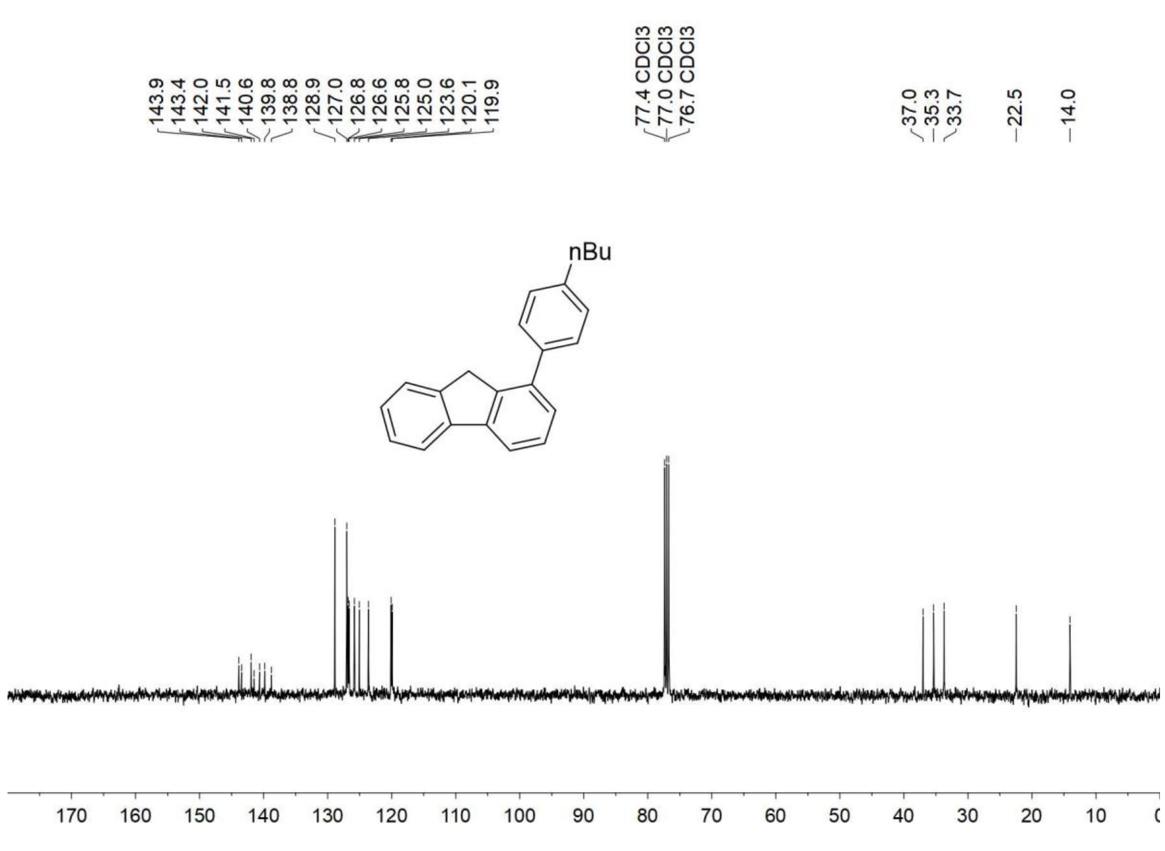
143.9
143.4
142.0
141.5
140.6
139.8
138.8
128.9
127.0
126.8
126.6
125.8
125.0
123.6
120.1
119.9
77.4 CDCl3
77.0 CDCl3
76.7 CDCl3
37.0
35.3
33.7
22.5
14.0
nBu
170 160 150 140 130 120 110 100 90 80 70 60 50 40 30 20 10

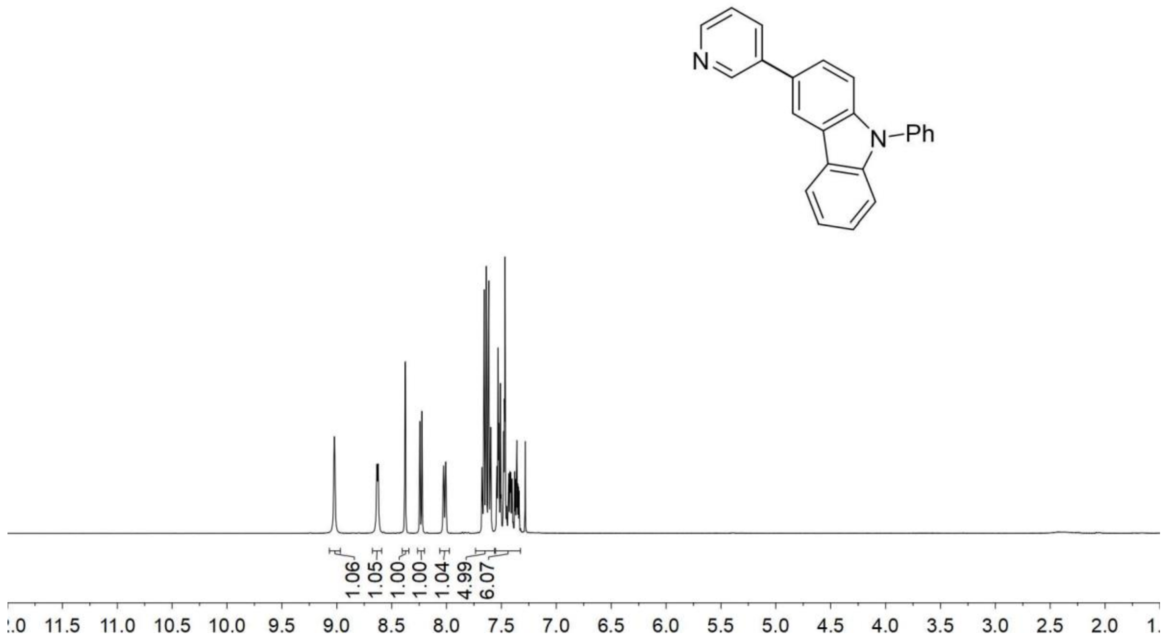
N
N–Ph
1.06
1.05
1.00
1.00
1.04
4.99
6.07
12.0 11.5 11.0 10.5 10.0 9.5 9.0 8.5 8.0 7.5 7.0 6.5 6.0 5.5 5.0 4.5 4.0 3.5 3.0 2.5 2.0 1.

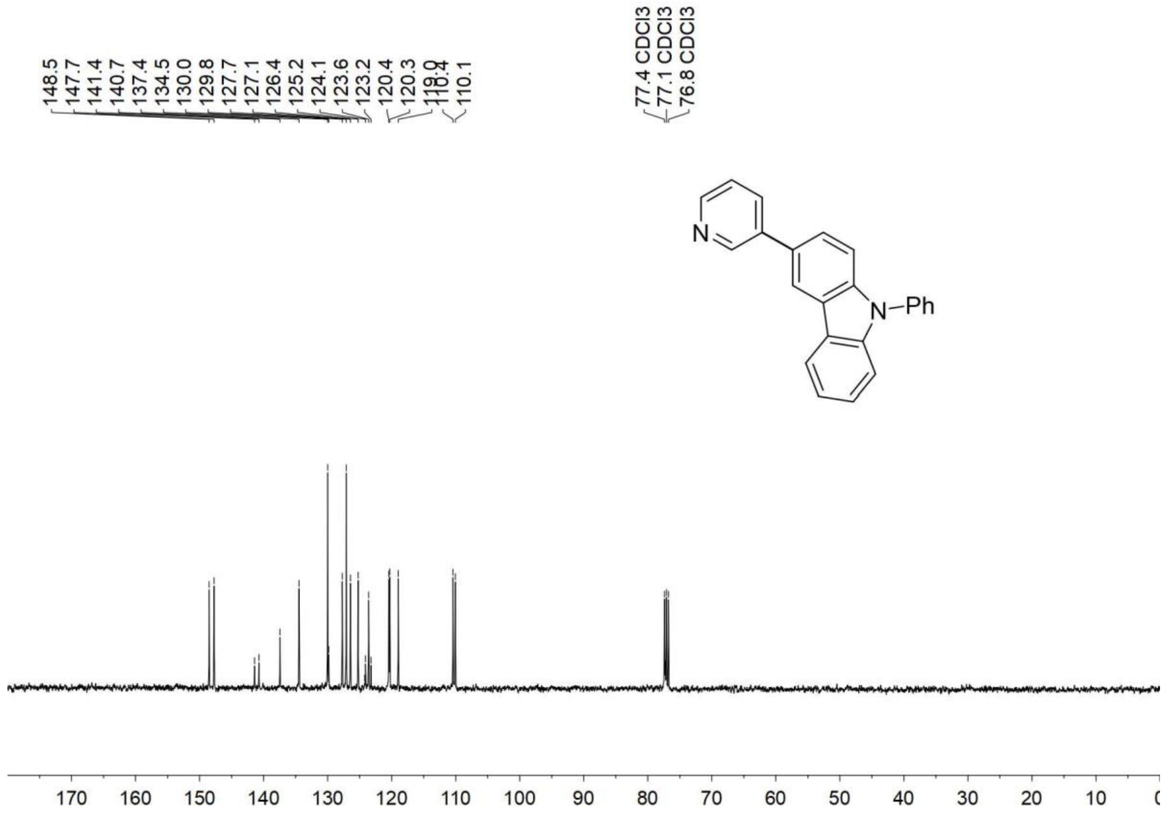
148.5
147.7
141.4
140.7
137.4
134.5
130.0
129.8
127.7
127.1
126.4
125.2
124.1
123.6
123.2
120.4
120.3
110.1
77.4 CDCl3
77.1 CDCl3
76.8 CDCl3
N
N–Ph
170 160 150 140 130 120 110 100 90 80 70 60 50 40 30 20 10 0

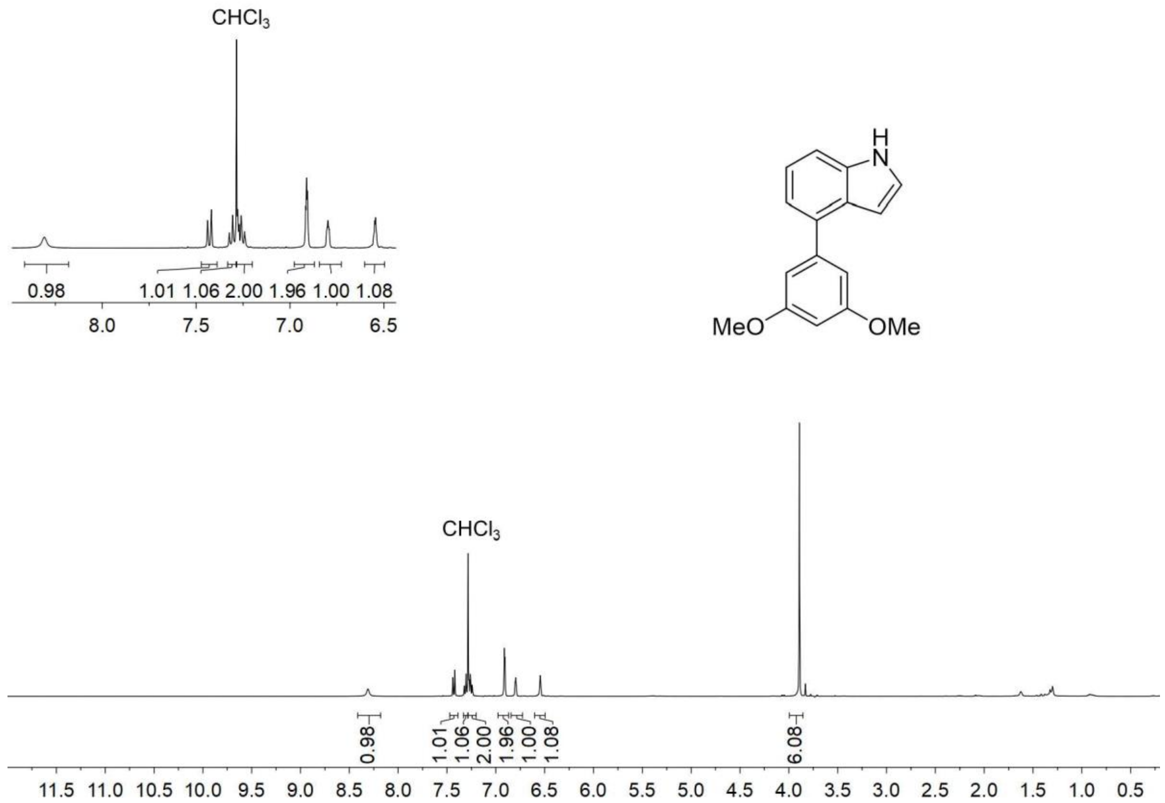
CHCl$_3$
0.98
1.01 1.06 2.00 1.96 1.00 1.08
8.0
7.5
7.0
6.5
H
N
MeO
OMe
CHCl$_3$
0.98
1.01
1.06
2.00
1.96
1.00
1.08
6.08
11.5 11.0 10.5 10.0 9.5 9.0 8.5 8.0 7.5 7.0 6.5 6.0 5.5 5.0 4.5 4.0 3.5 3.0 2.5 2.0 1.5 1.0 0.5

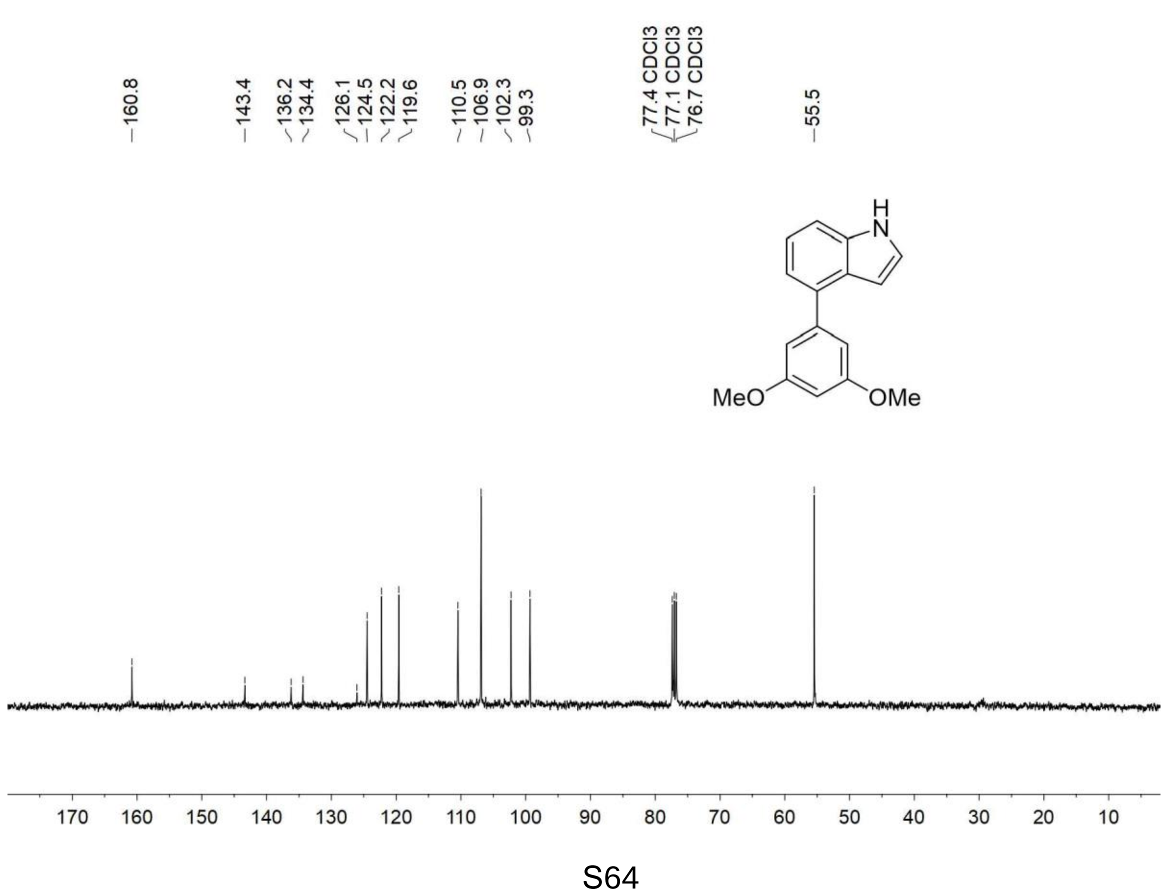
160.8
143.4
136.2
134.4
126.1
124.5
122.2
119.6
110.5
106.9
102.3
99.3
77.4 CDCl3
77.1 CDCl3
76.7 CDCl3
55.5
H
N
MeO
OMe
170 160 150 140 130 120 110 100 90 80 70 60 50 40 30 20 10

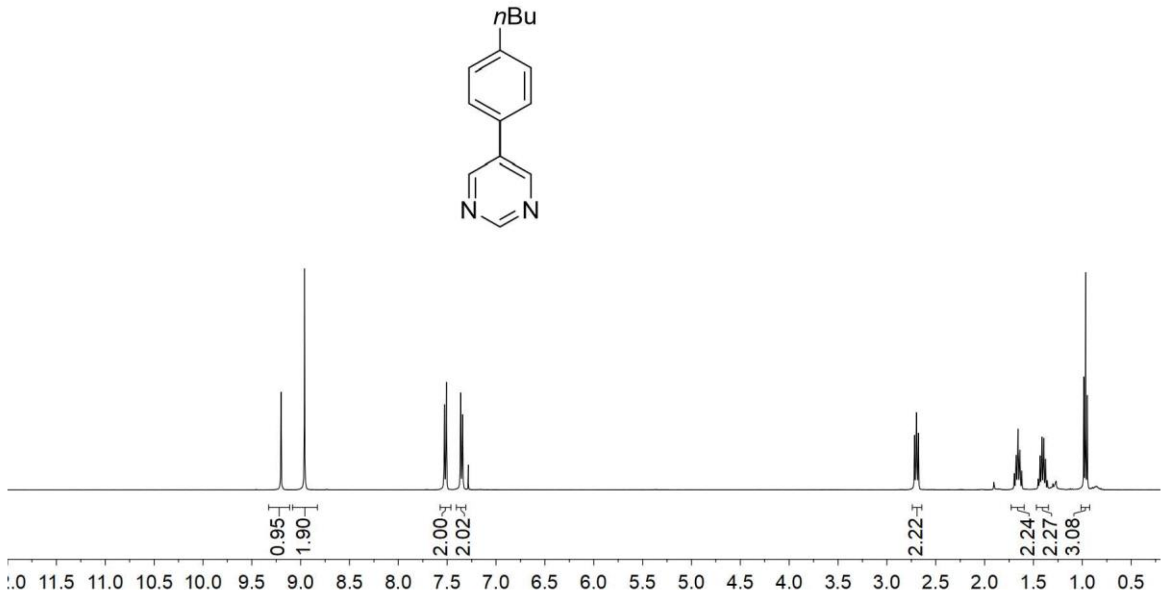
nBu
N
N
0.95
1.90
2.00
2.02
2.22
2.24
2.27
3.08
11.5 11.0 10.5 10.0 9.5 9.0 8.5 8.0 7.5 7.0 6.5 6.0 5.5 5.0 4.5 4.0 3.5 3.0 2.5 2.0 1.5 1.0 0.5

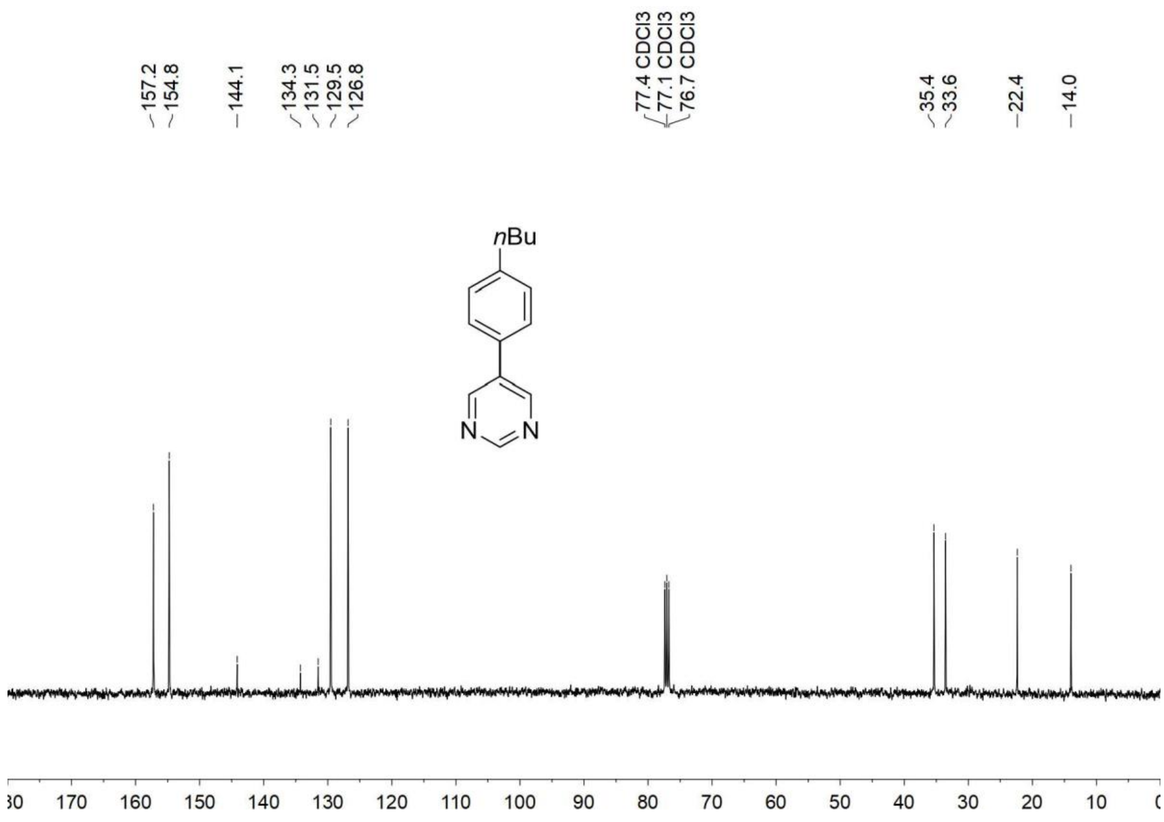
157.2
154.8
144.1
134.3
131.5
129.5
126.8
77.4 CDCl3
77.1 CDCl3
76.7 CDCl3
35.4
33.6
22.4
14.0
nBu
N
N
170 160 150 140 130 120 110 100 90 80 70 60 50 40 30 20 10

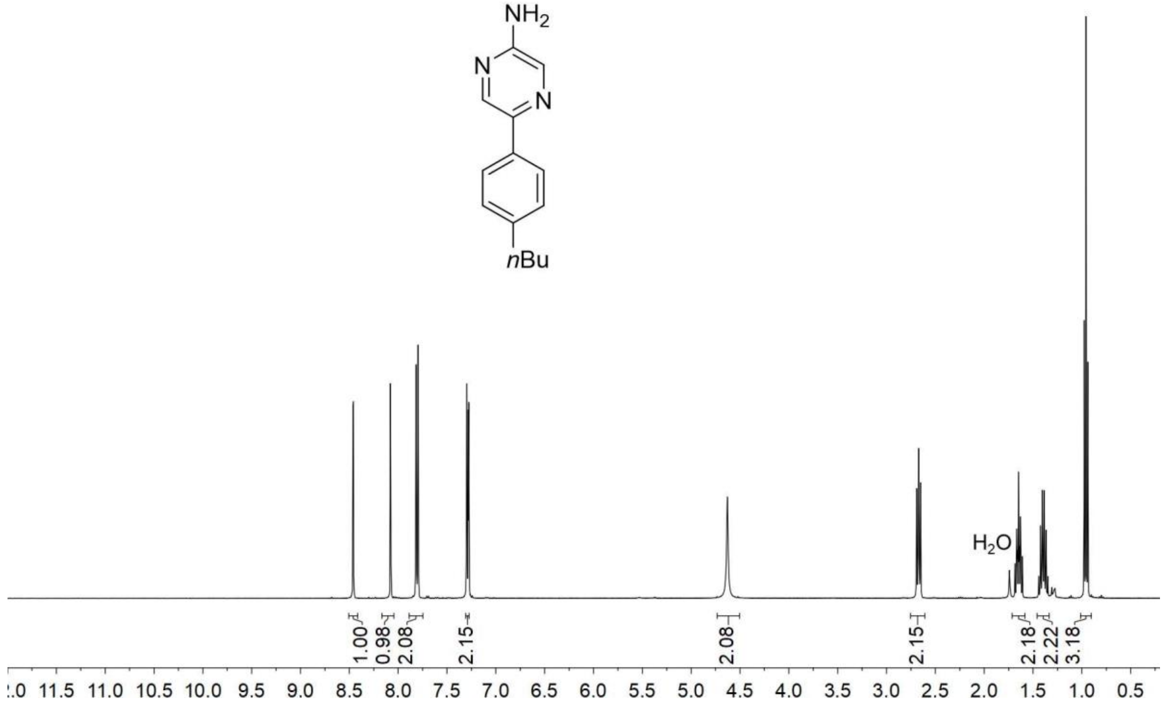
NH2
N
N
nBu
H2O
1.00
0.98
2.08
2.15
2.08
2.15
2.18
2.22
3.18
12.0 11.5 11.0 10.5 10.0 9.5 9.0 8.5 8.0 7.5 7.0 6.5 6.0 5.5 5.0 4.5 4.0 3.5 3.0 2.5 2.0 1.5 1.0 0.5

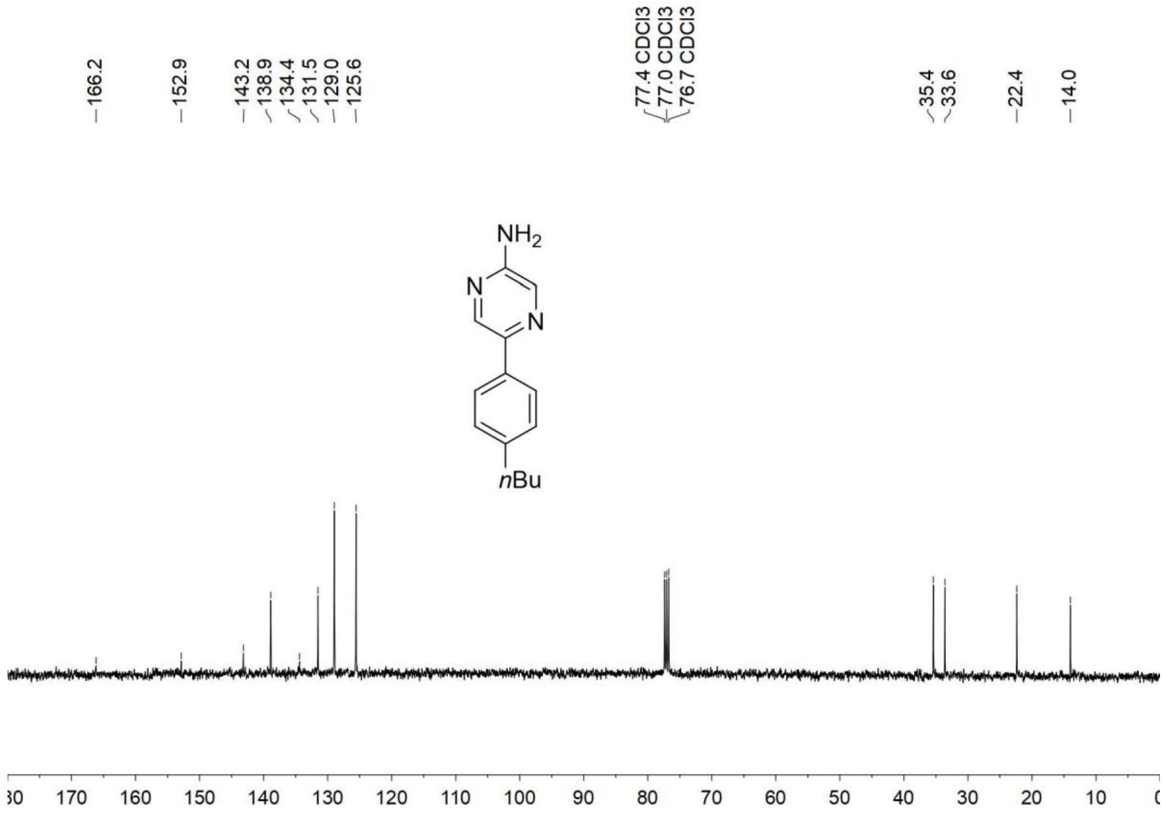
166.2
152.9
143.2
138.9
134.4
131.5
129.0
125.6
77.4 CDCl3
77.0 CDCl3
76.7 CDCl3
35.4
33.6
22.4
14.0
NH2
N
N
nBu
170 160 150 140 130 120 110 100 90 80 70 60 50 40 30 20 10

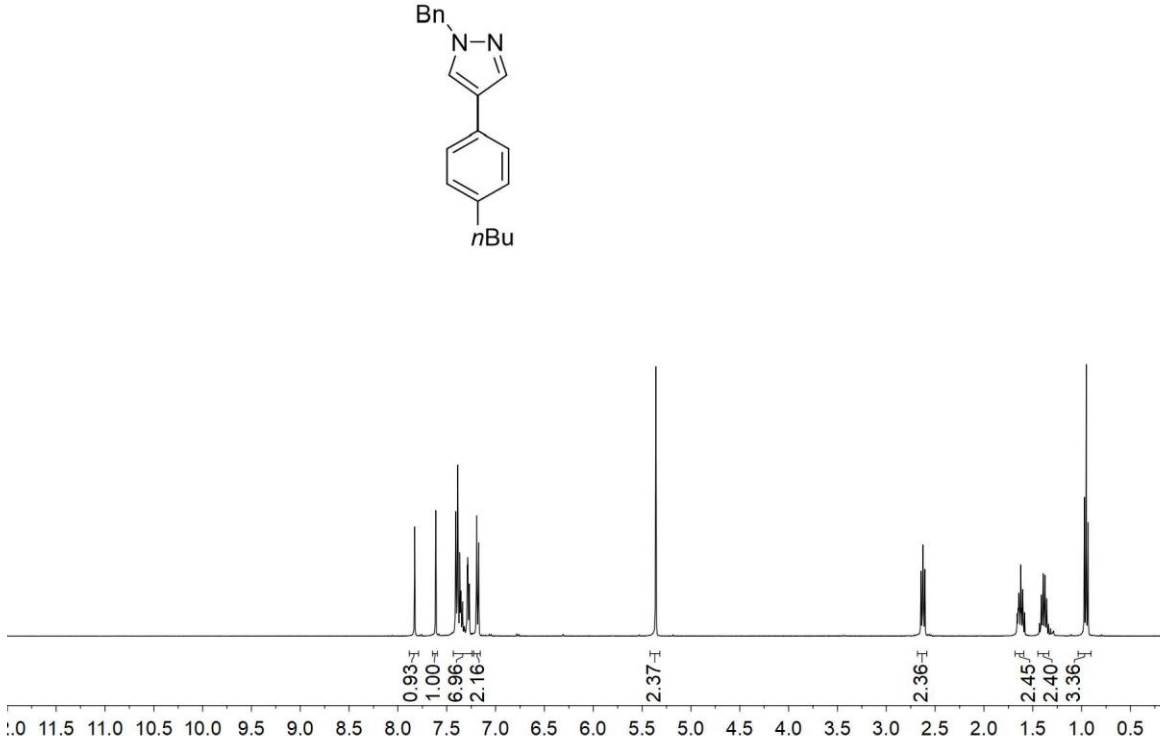
Bn
N–N
nBu
0.93
1.00
6.96
2.16
2.37
2.36
2.45
2.40
3.36

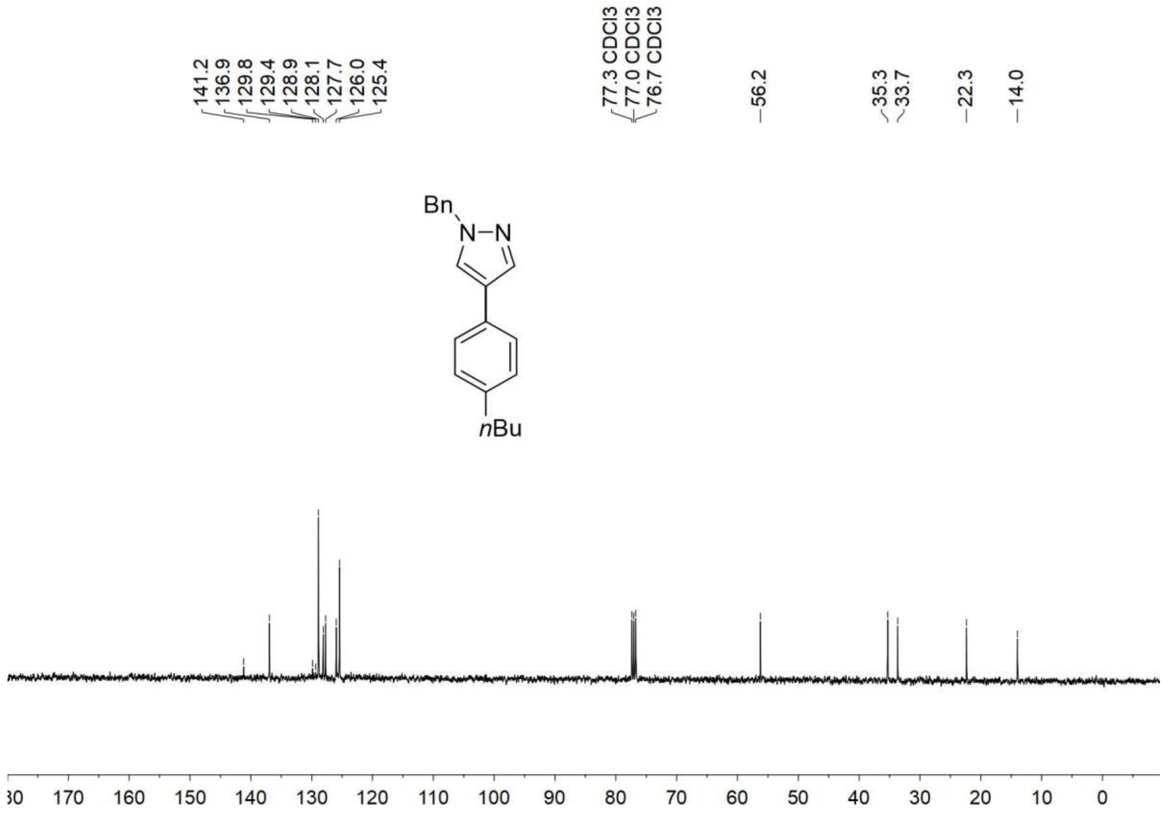
141.2
136.9
129.8
129.4
128.9
128.1
127.7
126.0
125.4
77.3 CDCl3
77.0 CDCl3
76.7 CDCl3
56.2
35.3
33.7
22.3
14.0
Bn
N–N
nBu

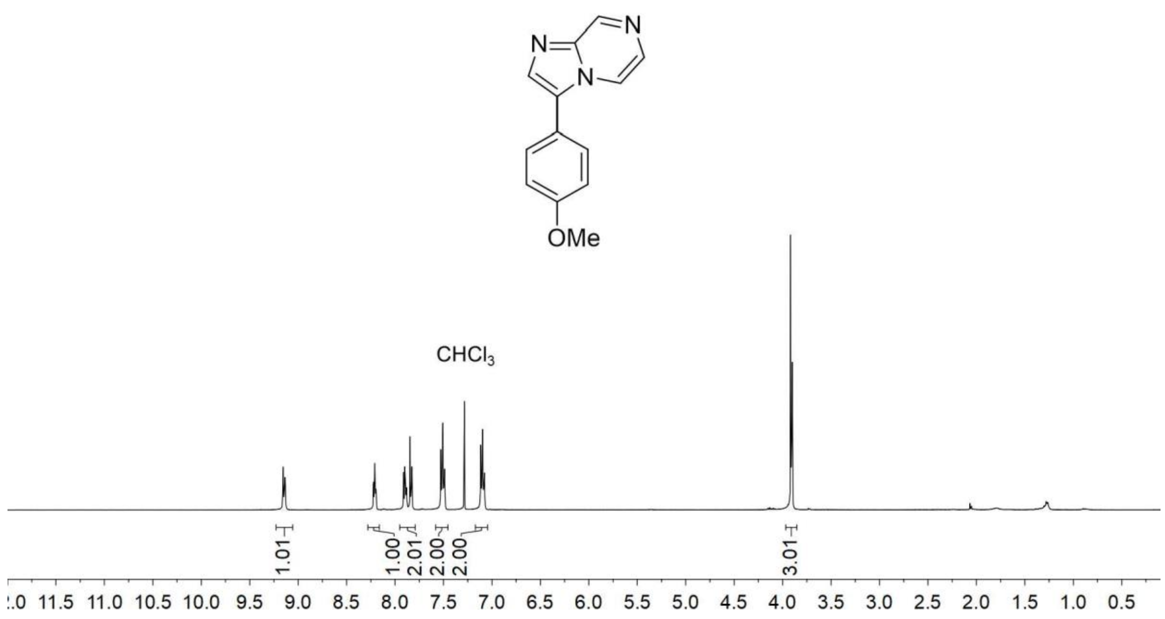

N
N
N
OMe
CHCl3
1.01
1.00
2.01
2.00
2.00
3.01
12.0 11.5 11.0 10.5 10.0 9.5 9.0 8.5 8.0 7.5 7.0 6.5 6.0 5.5 5.0 4.5 4.0 3.5 3.0 2.5 2.0 1.5 1.0 0.5

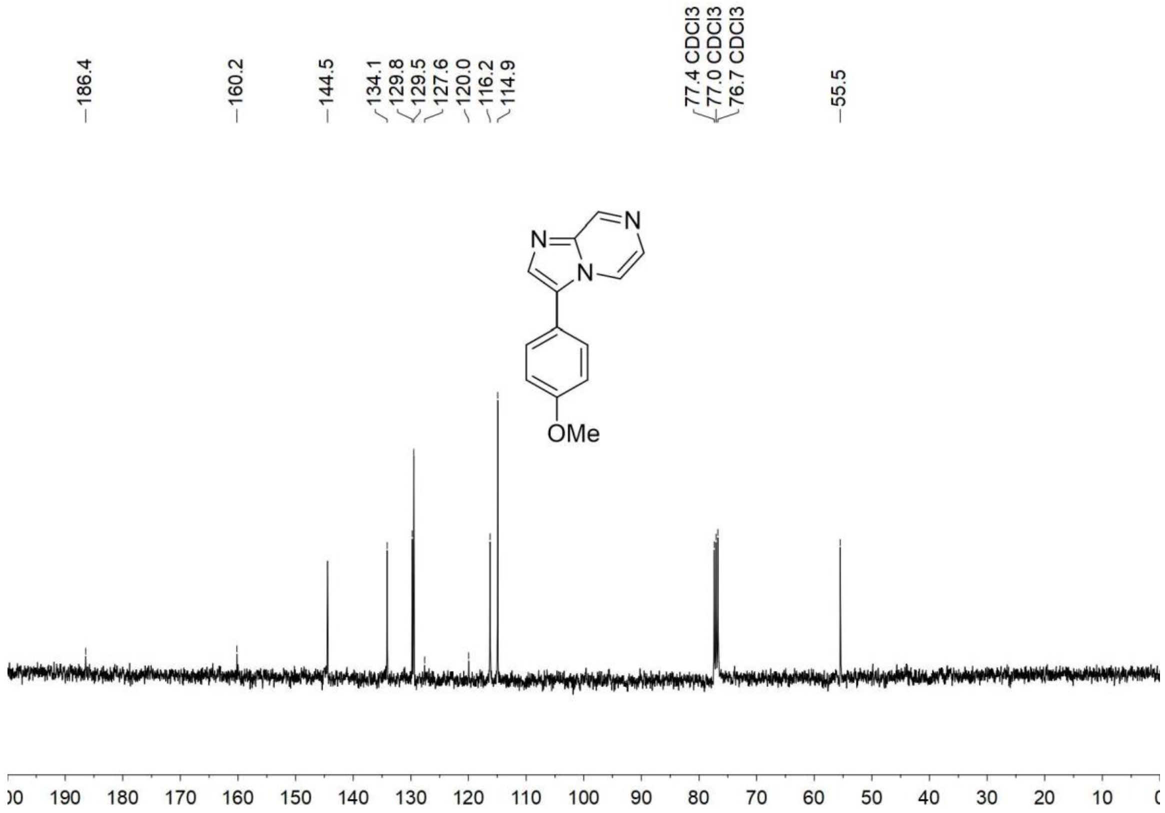

186.4
160.2
144.5
134.1
129.8
129.5
127.6
120.0
116.2
114.9
77.4 CDCl3
77.0 CDCl3
76.7 CDCl3
55.5
N
N
N
OMe
190 180 170 160 150 140 130 120 110 100 90 80 70 60 50 40 30 20 10

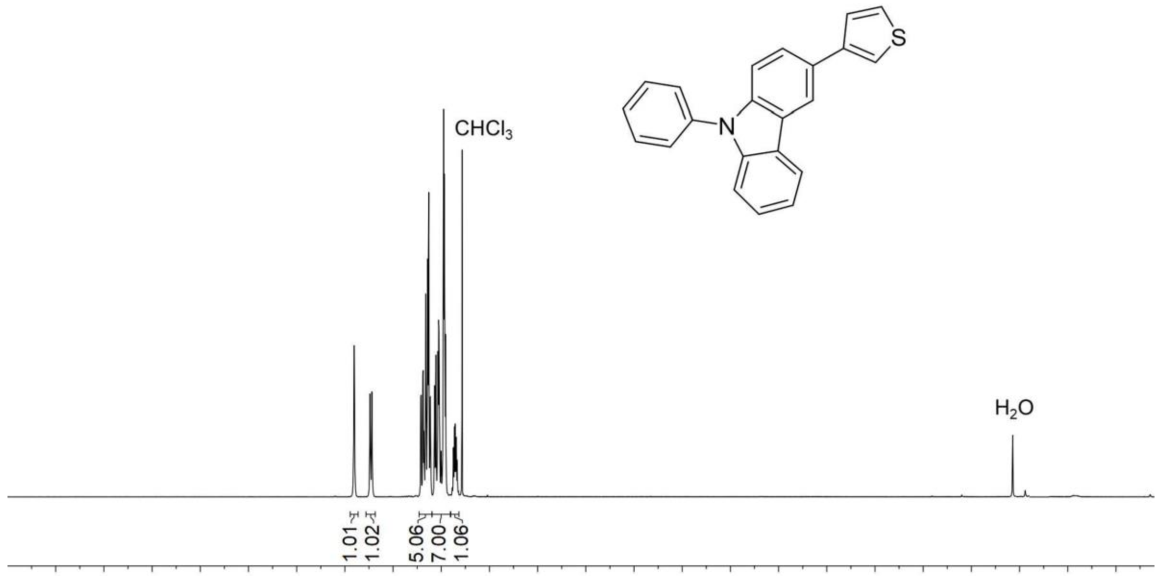
CHCl$_3$
S
N
H_2O
1.01
1.02
5.06
7.00
1.06
11.5 11.0 10.5 10.0 9.5 9.0 8.5 8.0 7.5 7.0 6.5 6.0 5.5 5.0 4.5 4.0 3.5 3.0 2.5 2.0 1.5 1.0 0.5

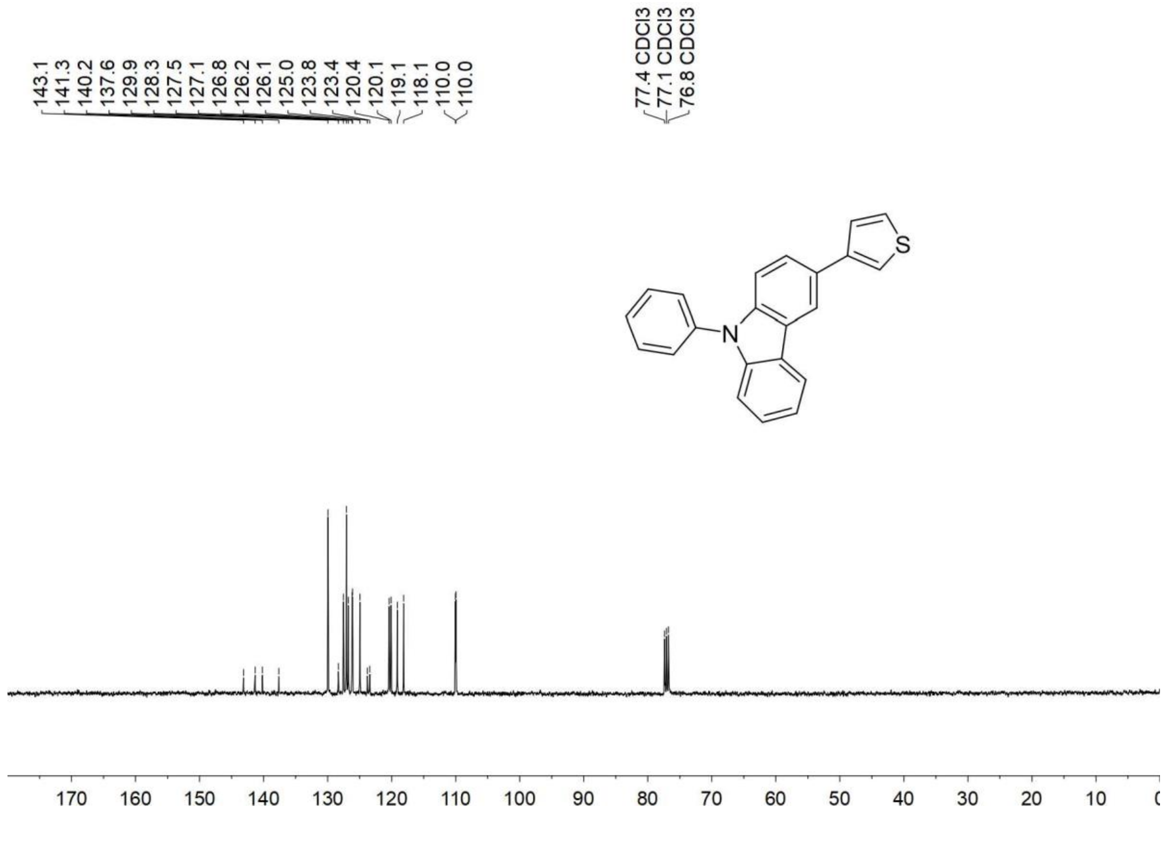
143.1
141.3
140.2
137.6
129.9
128.3
127.5
127.1
126.8
126.2
126.1
125.0
123.8
123.4
120.4
120.1
119.1
118.1
110.0
110.0
77.4 CDCl3
77.1 CDCl3
76.8 CDCl3
S
N
170 160 150 140 130 120 110 100 90 80 70 60 50 40 30 20 10

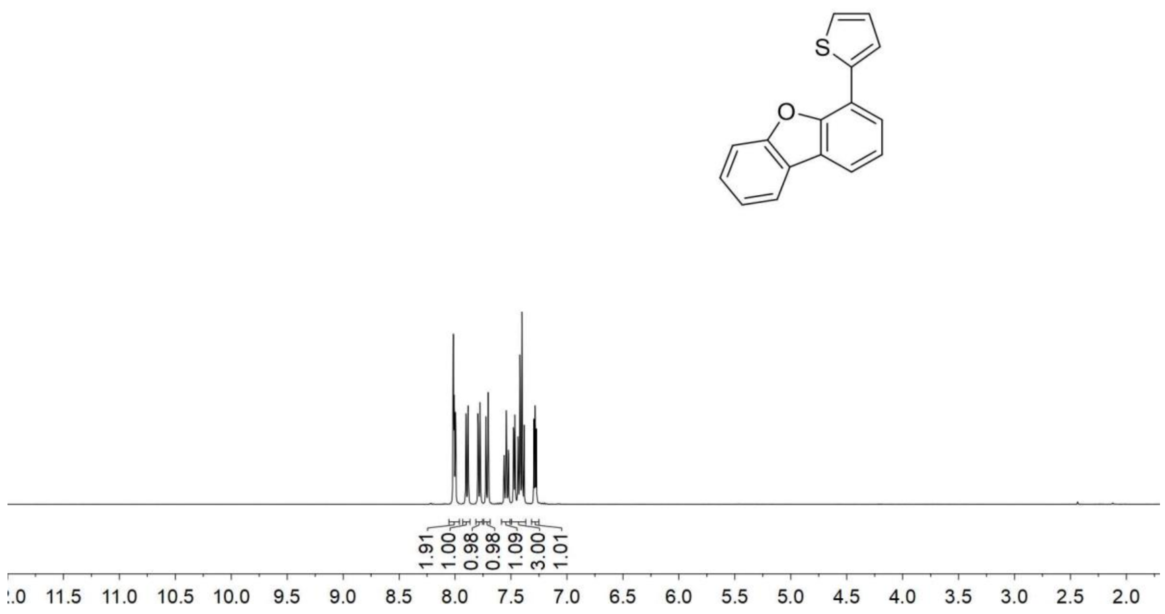
S
O
1.91
1.00
0.98
0.98
0.98
1.09
3.00
1.01
.0 11.5 11.0 10.5 10.0 9.5 9.0 8.5 8.0 7.5 7.0 6.5 6.0 5.5 5.0 4.5 4.0 3.5 3.0 2.5 2.0

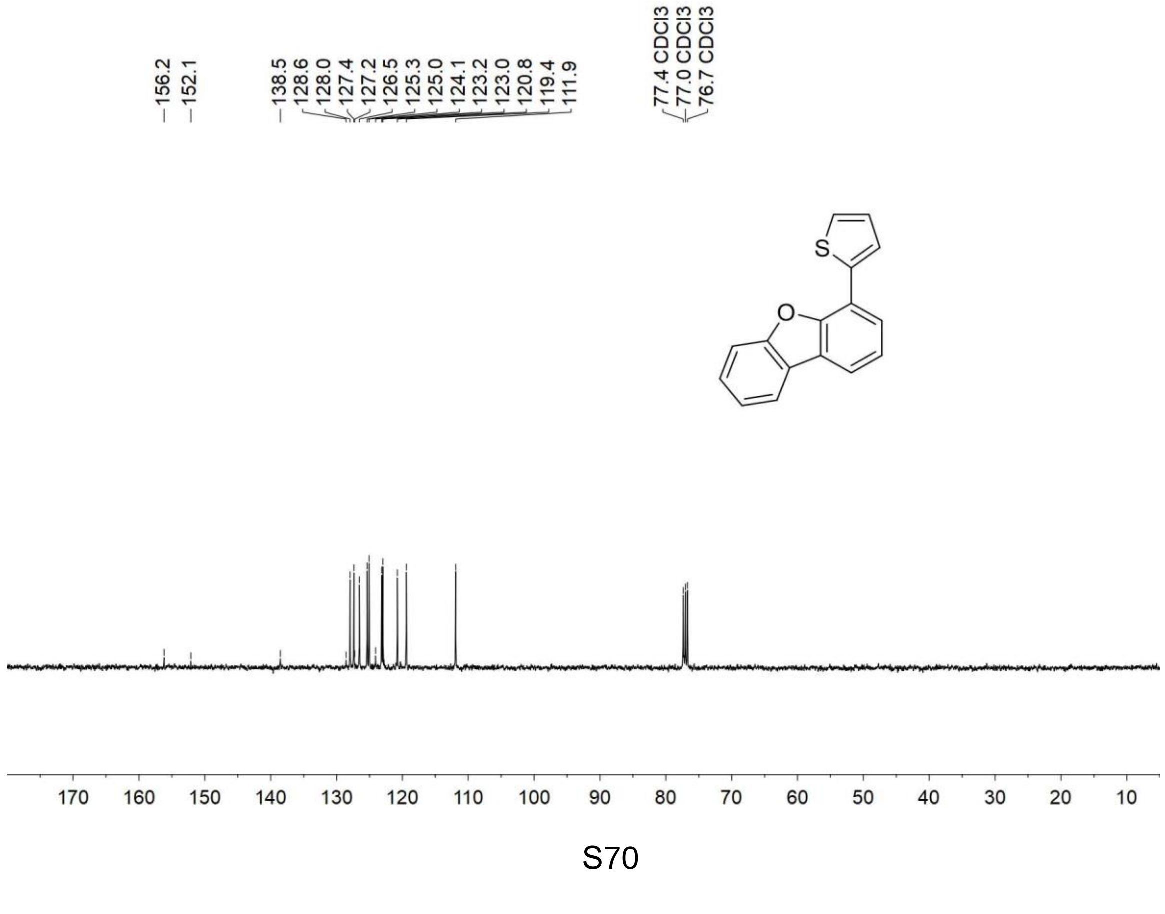
156.2
152.1
138.5
128.6
128.0
127.4
127.2
126.5
125.3
125.0
124.1
123.2
123.0
120.8
119.4
111.9
77.4 CDCl3
77.0 CDCl3
76.7 CDCl3
S
O
170 160 150 140 130 120 110 100 90 80 70 60 50 40 30 20 10

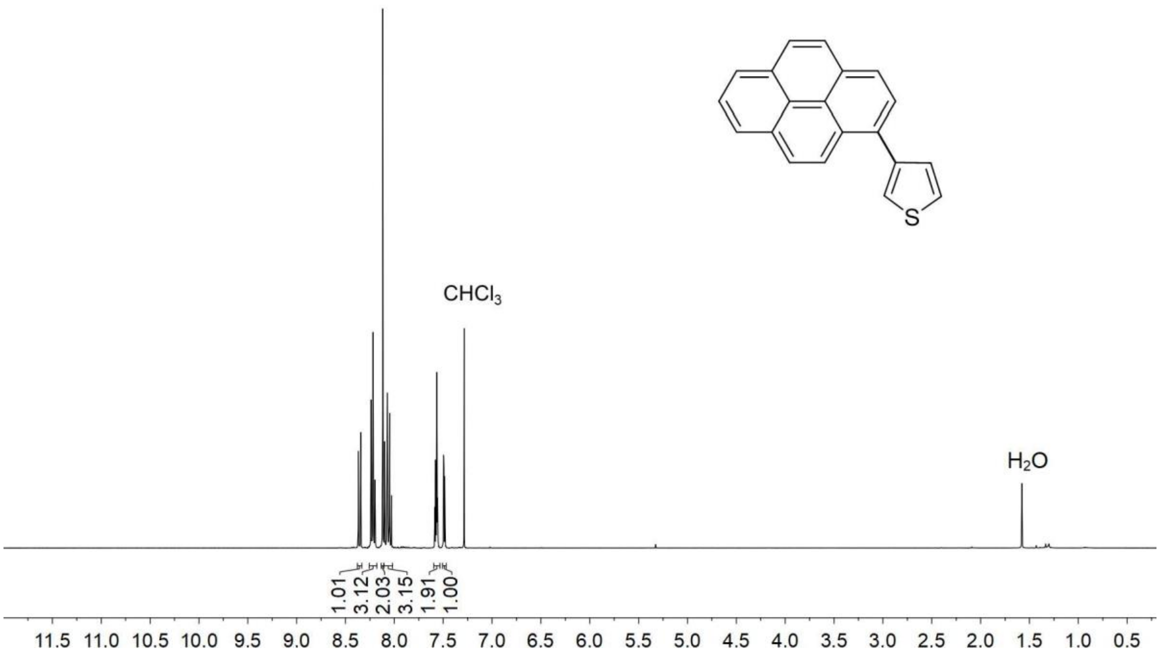
CHCl$_3$
H_2O
1.01
3.12
2.03
3.15
1.91
1.00
11.5 11.0 10.5 10.0 9.5 9.0 8.5 8.0 7.5 7.0 6.5 6.0 5.5 5.0 4.5 4.0 3.5 3.0 2.5 2.0 1.5 1.0 0.5
S

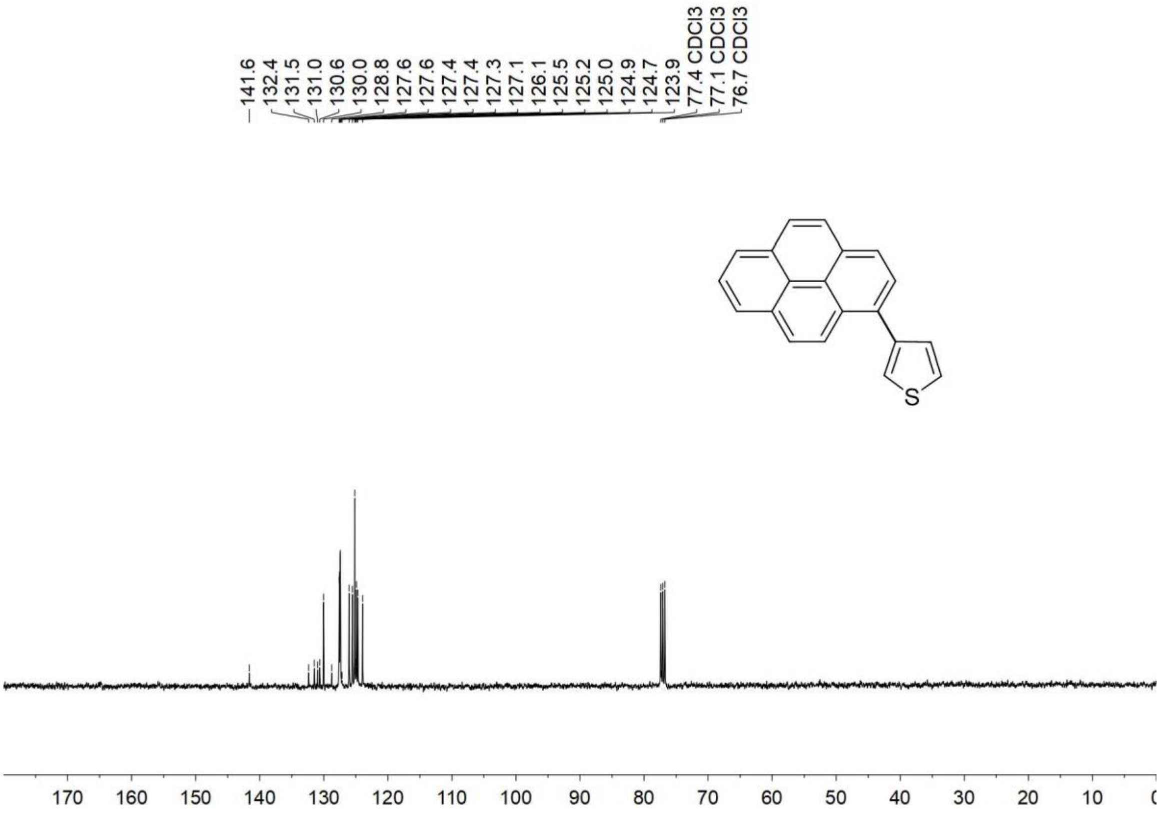
141.6
132.4
131.5
131.0
130.6
130.0
128.8
127.6
127.6
127.4
127.4
127.3
127.1
126.1
125.5
125.2
125.0
124.9
124.7
123.9
77.4 CDCl3
77.1 CDCl3
76.7 CDCl3
S
170 160 150 140 130 120 110 100 90 80 70 60 50 40 30 20 10

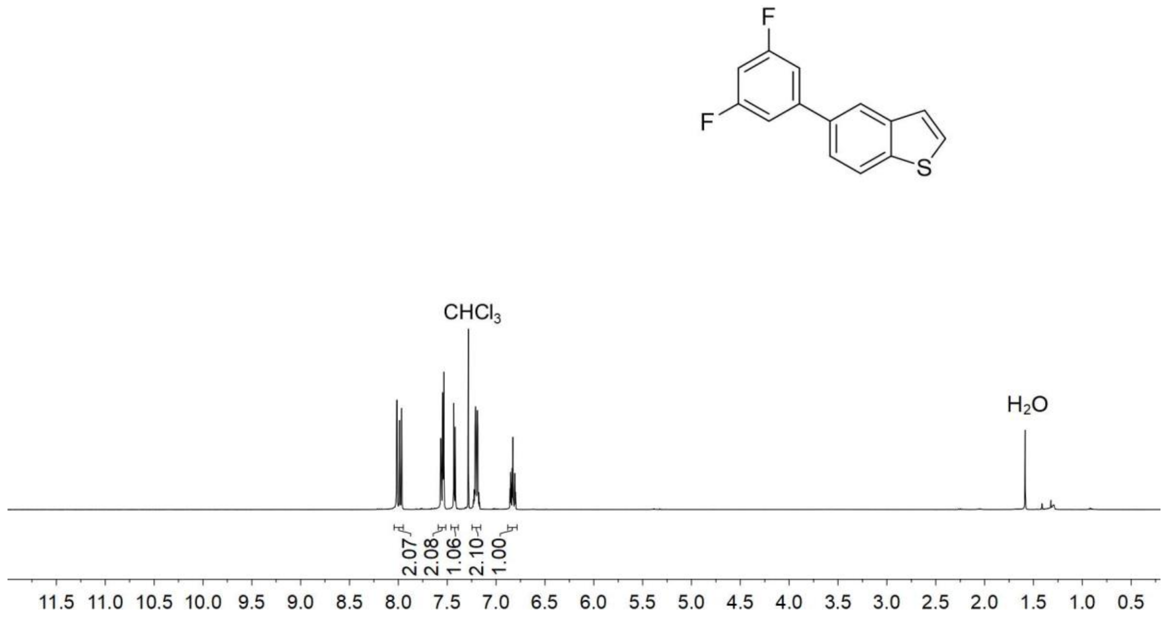
F
F
S
CHCl3
H2O
2.07
2.08
1.06
2.10
1.00
11.5 11.0 10.5 10.0 9.5 9.0 8.5 8.0 7.5 7.0 6.5 6.0 5.5 5.0 4.5 4.0 3.5 3.0 2.5 2.0 1.5 1.0 0.5

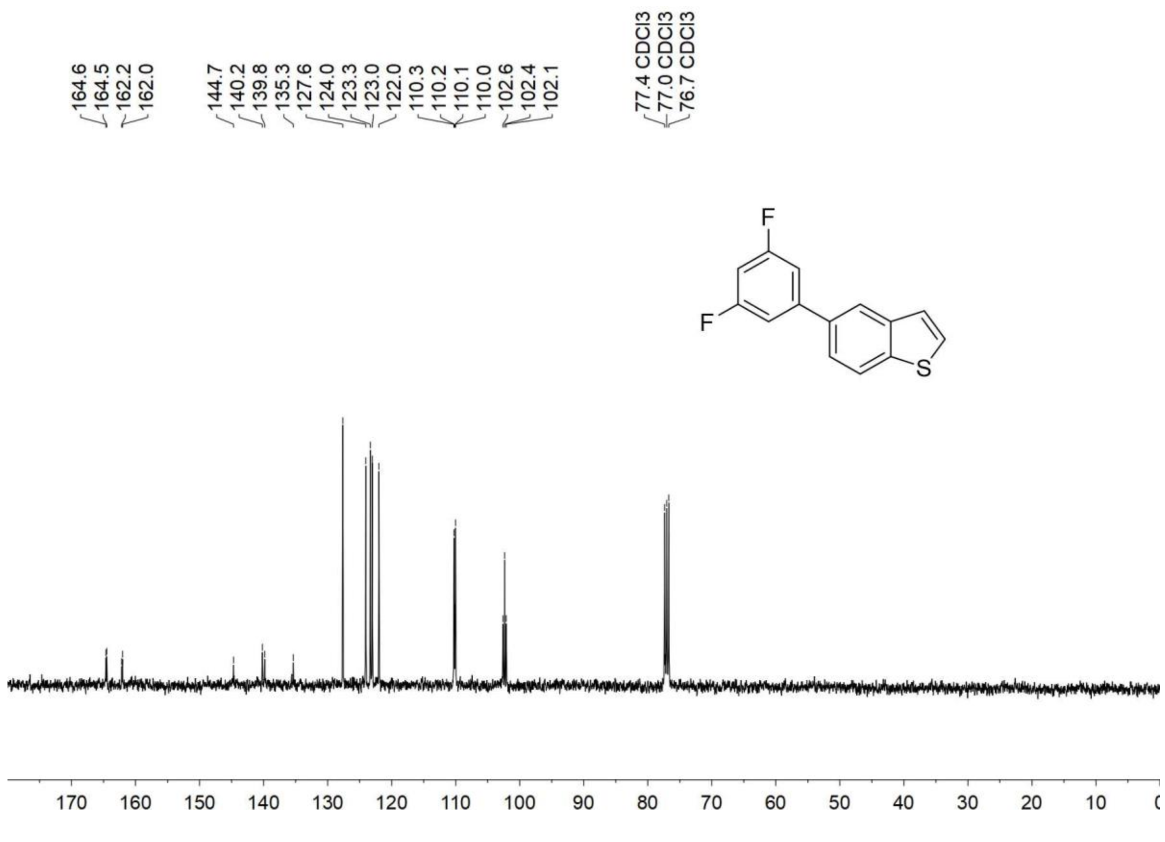
164.6
164.5
162.2
162.0
144.7
140.2
139.8
135.3
127.6
124.0
123.3
123.0
122.0
110.3
110.2
110.1
110.0
102.6
102.4
102.1
77.4 CDCl3
77.0 CDCl3
76.7 CDCl3
F
F
S
170 160 150 140 130 120 110 100 90 80 70 60 50 40 30 20 10

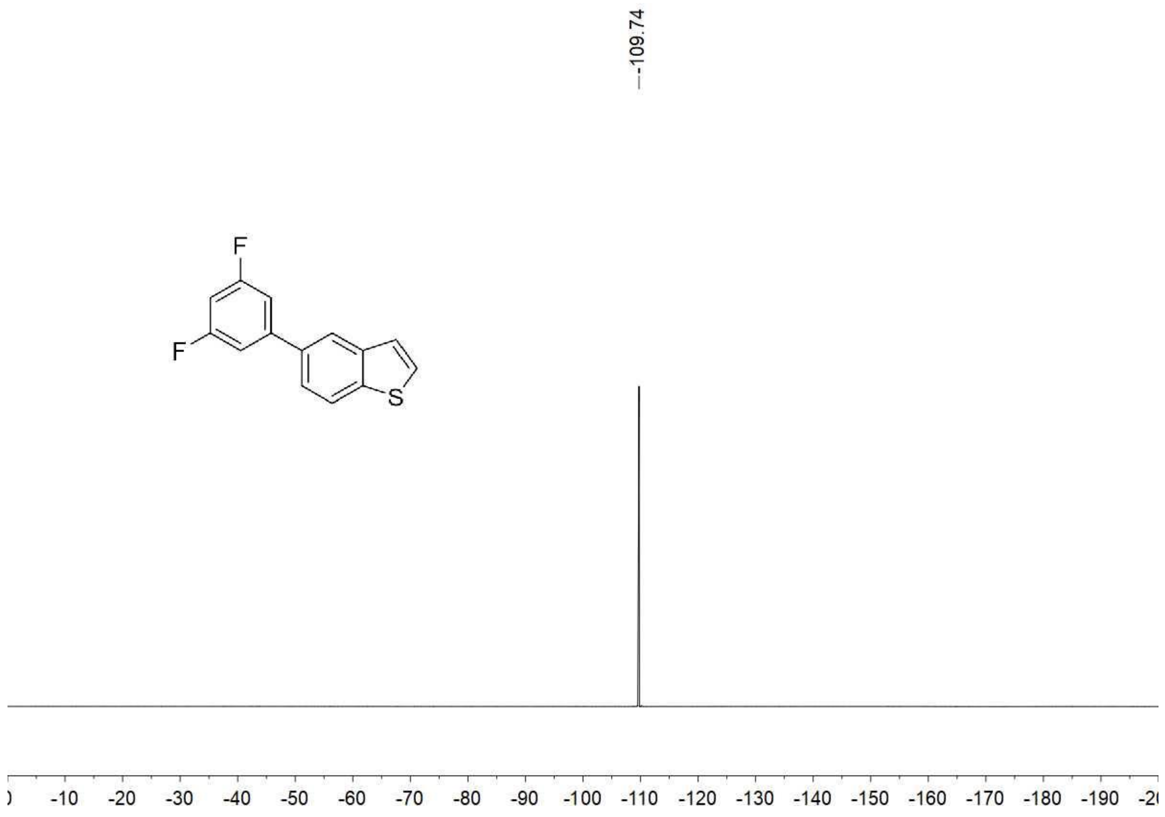

--109.74
F
F
S
-10 -20 -30 -40 -50 -60 -70 -80 -90 -100 -110 -120 -130 -140 -150 -160 -170 -180 -190

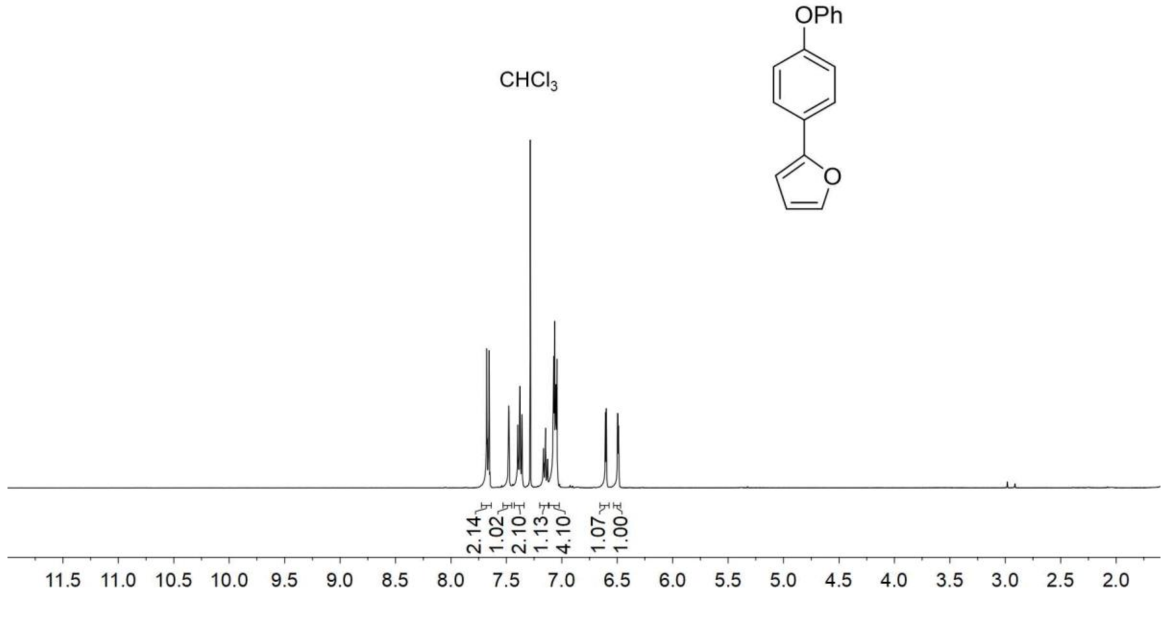

CHCl3
OPh
O
2.14
1.02
2.10
1.13
4.10
1.07
1.00
11.5 11.0 10.5 10.0 9.5 9.0 8.5 8.0 7.5 7.0 6.5 6.0 5.5 5.0 4.5 4.0 3.5 3.0 2.5 2.0

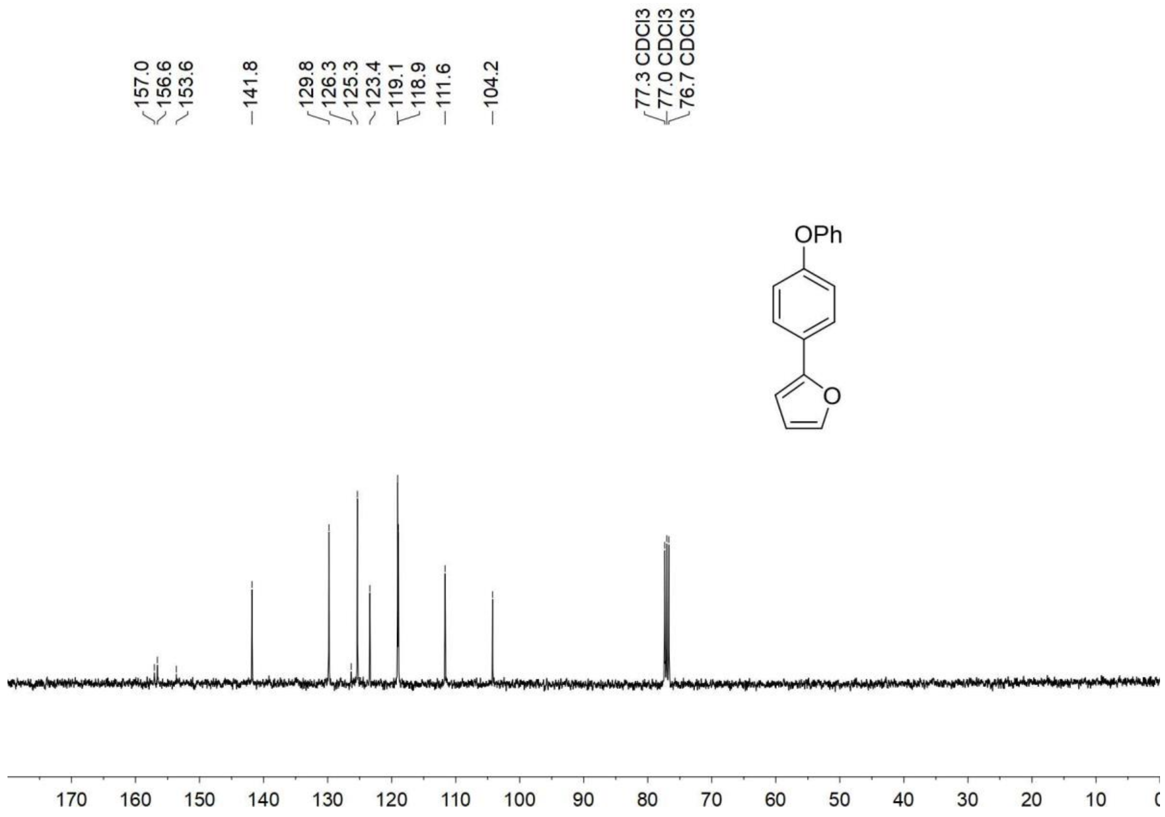
157.0
156.6
153.6
141.8
129.8
126.3
125.3
123.4
119.1
118.9
111.6
104.2
77.3 CDCl3
77.0 CDCl3
76.7 CDCl3
OPh
O
170 160 150 140 130 120 110 100 90 80 70 60 50 40 30 20 10

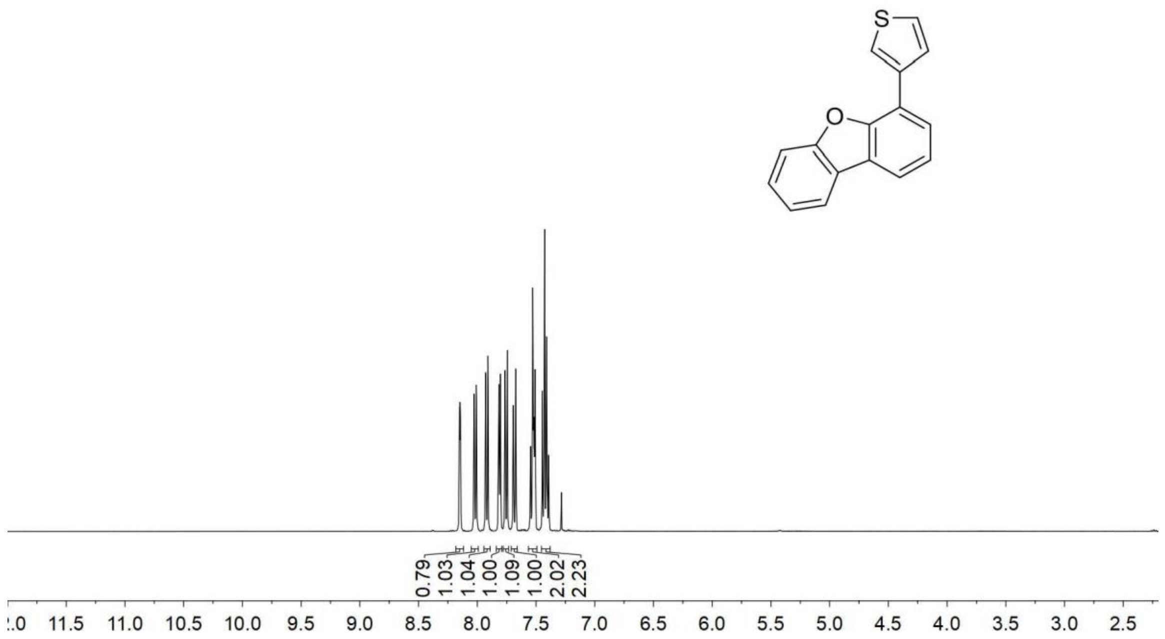
S
O
0.79
1.03
1.04
1.00
1.09
1.00
2.02
2.23
11.5 11.0 10.5 10.0 9.5 9.0 8.5 8.0 7.5 7.0 6.5 6.0 5.5 5.0 4.5 4.0 3.5 3.0 2.5

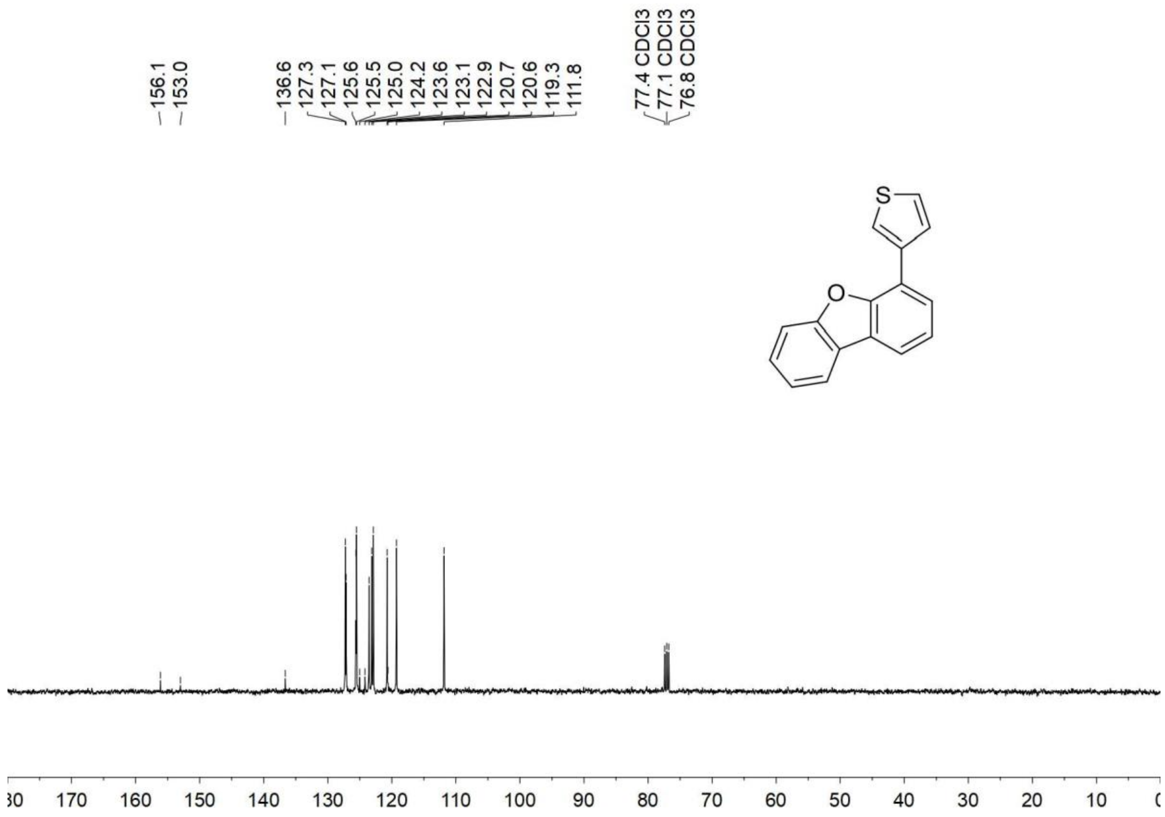
156.1
153.0
136.6
127.3
127.1
125.6
125.5
125.0
124.2
123.6
123.1
122.9
120.7
120.6
119.3
111.8
77.4 CDCl3
77.1 CDCl3
76.8 CDCl3
S
O
170
160
150
140
130
120
110
100
90
80
70
60
50
40
30
20
10

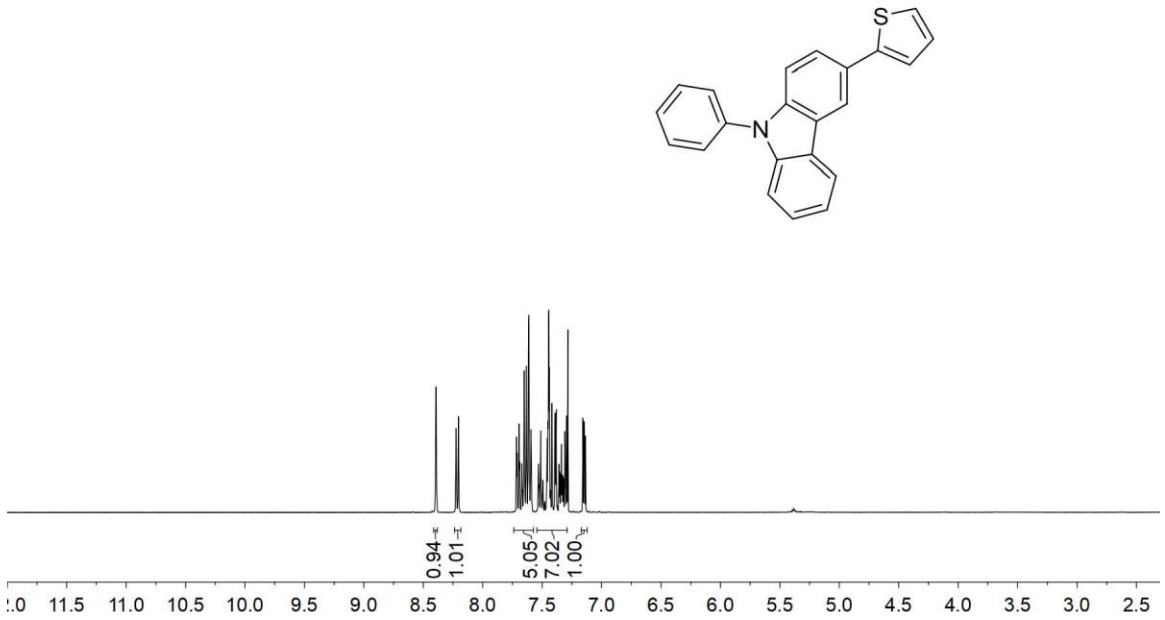
S
N
0.94
1.01
5.05
7.02
1.00
11.5
11.0
10.5
10.0
9.5
9.0
8.5
8.0
7.5
7.0
6.5
6.0
5.5
5.0
4.5
4.0
3.5
3.0
2.5

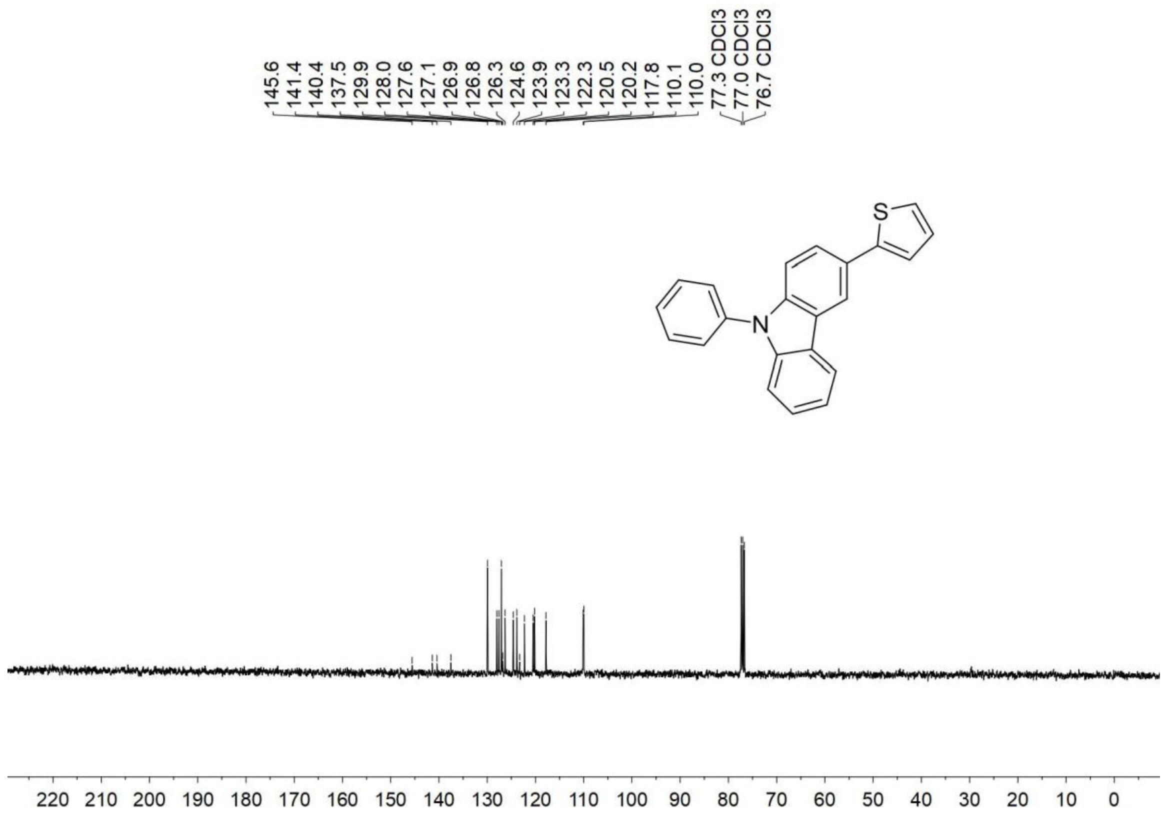
145.6
141.4
140.4
137.5
129.9
128.0
127.6
127.1
126.9
126.8
126.3
124.6
123.9
123.3
122.3
120.5
120.2
117.8
110.1
110.0
77.3 CDCl3
77.0 CDCl3
76.7 CDCl3
S
N
220 210 200 190 180 170 160 150 140 130 120 110 100 90 80 70 60 50 40 30 20 10 0

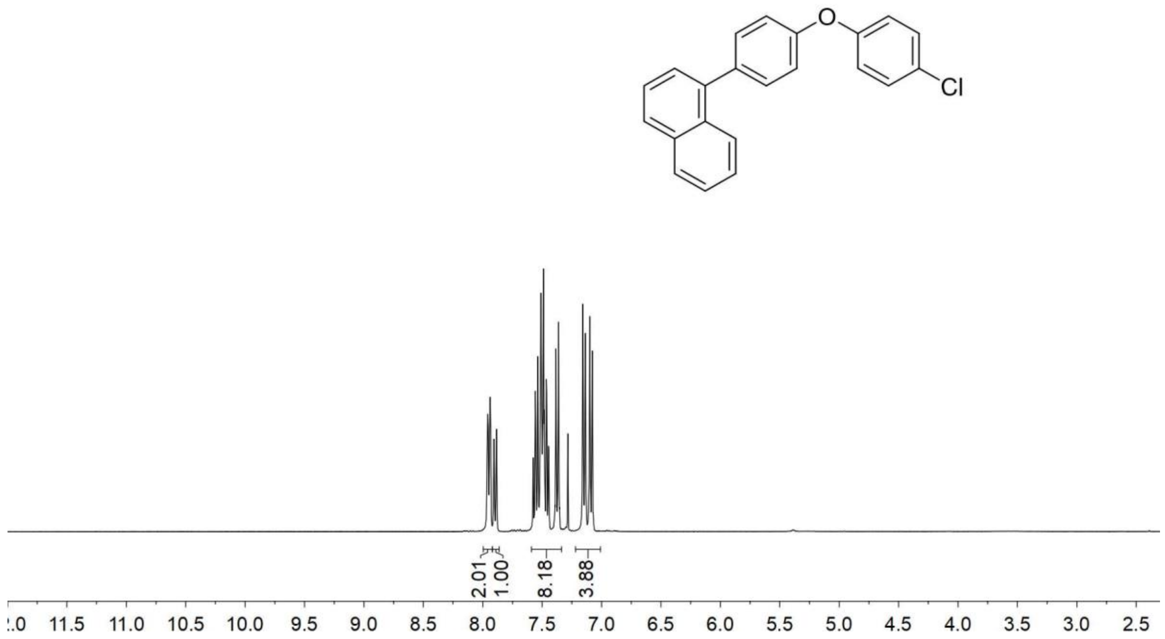
O
Cl
2.01
1.00
8.18
3.88
11.5 11.0 10.5 10.0 9.5 9.0 8.5 8.0 7.5 7.0 6.5 6.0 5.5 5.0 4.5 4.0 3.5 3.0 2.5

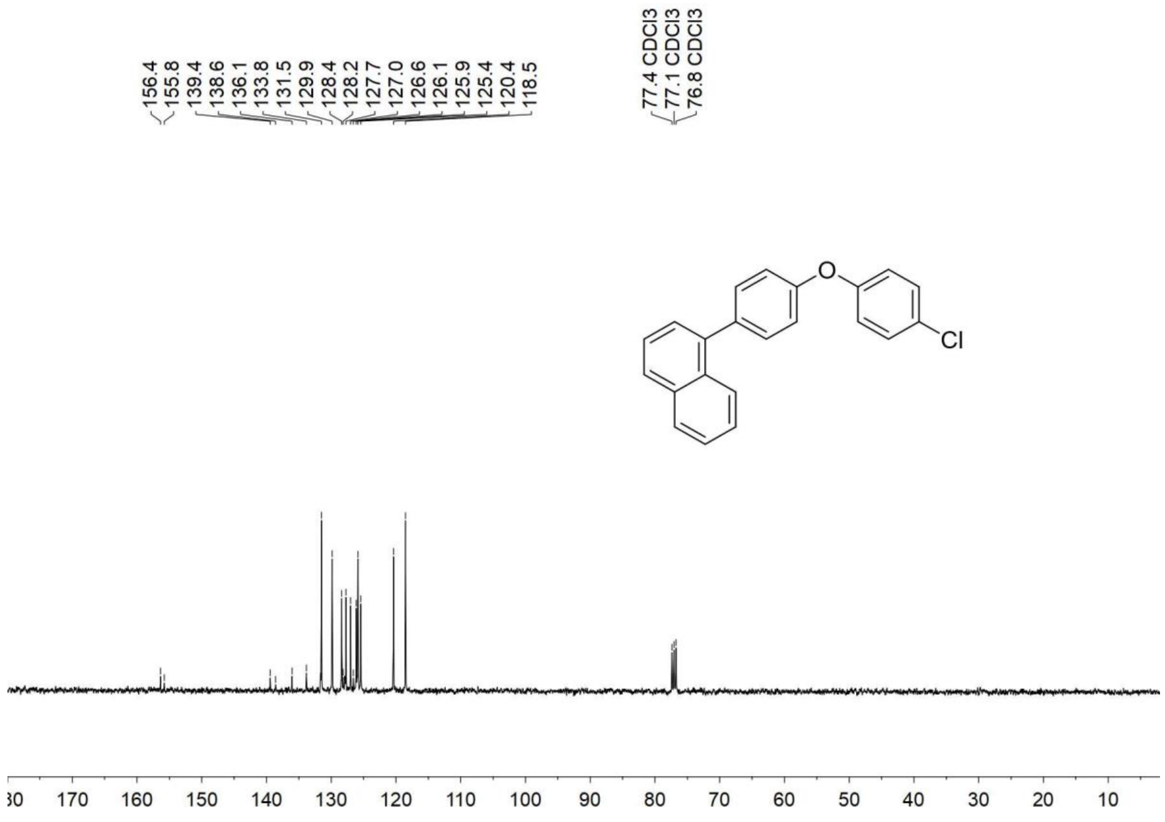
156.4
155.8
139.4
138.6
136.1
133.8
131.5
129.9
128.4
128.2
127.7
127.0
126.6
126.1
125.9
125.4
120.4
118.5
77.4 CDCl3
77.1 CDCl3
76.8 CDCl3
O
Cl

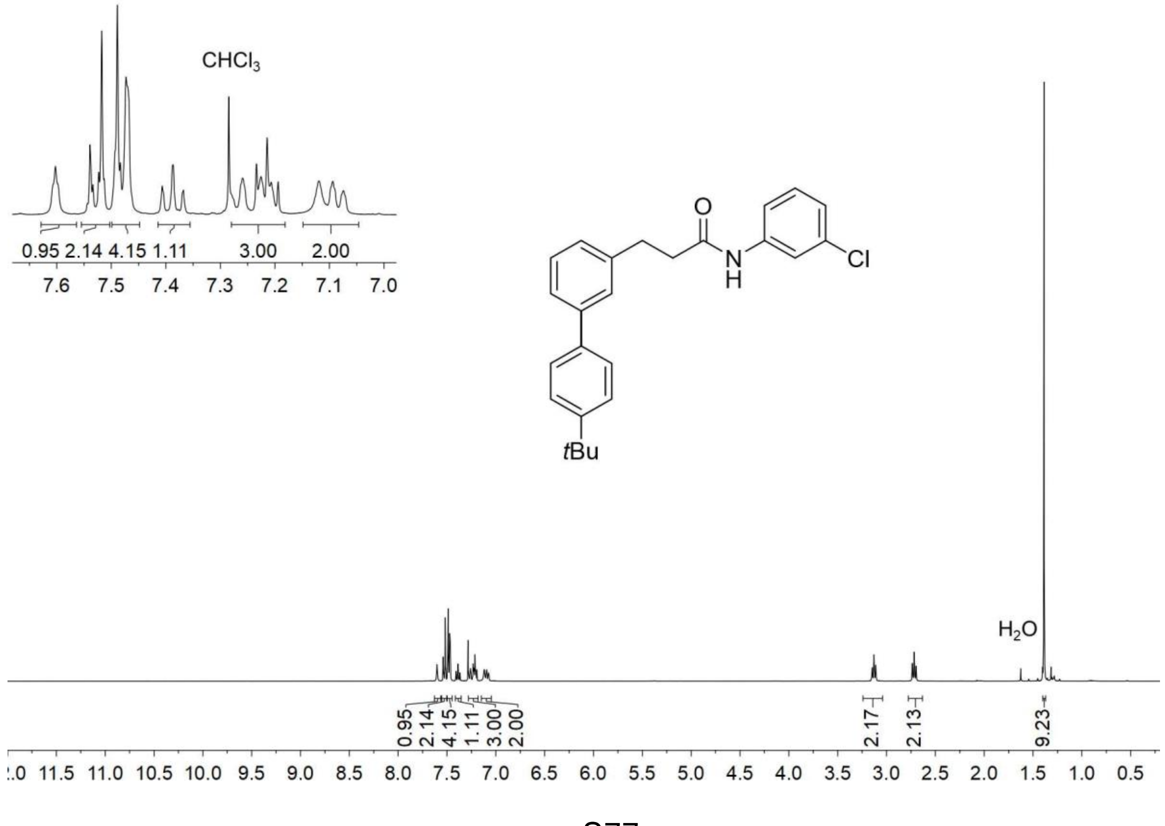
CHCl3
0.95 2.14 4.15 1.11 3.00 2.00
O
N
H
Cl
tBu
H2O
0.95
2.14
4.15
1.11
3.00
2.00
2.17
2.13
9.23

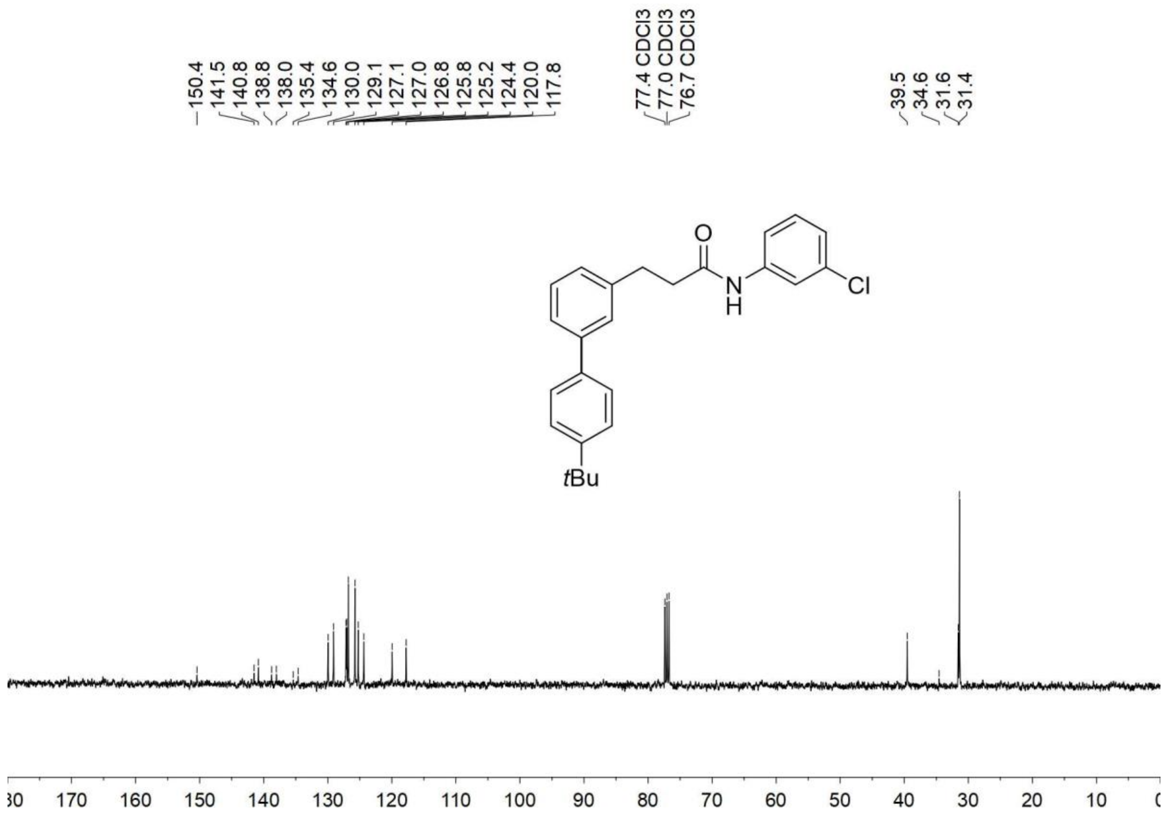

150.4
141.5
140.8
138.8
138.0
135.4
134.6
130.0
129.1
127.1
127.0
126.8
125.8
125.2
124.4
120.0
117.8
77.4 CDCl3
77.0 CDCl3
76.7 CDCl3
39.5
34.6
31.6
31.4
O
N
H
Cl
tBu
170
160
150
140
130
120
110
100
90
80
70
60
50
40
30
20
10

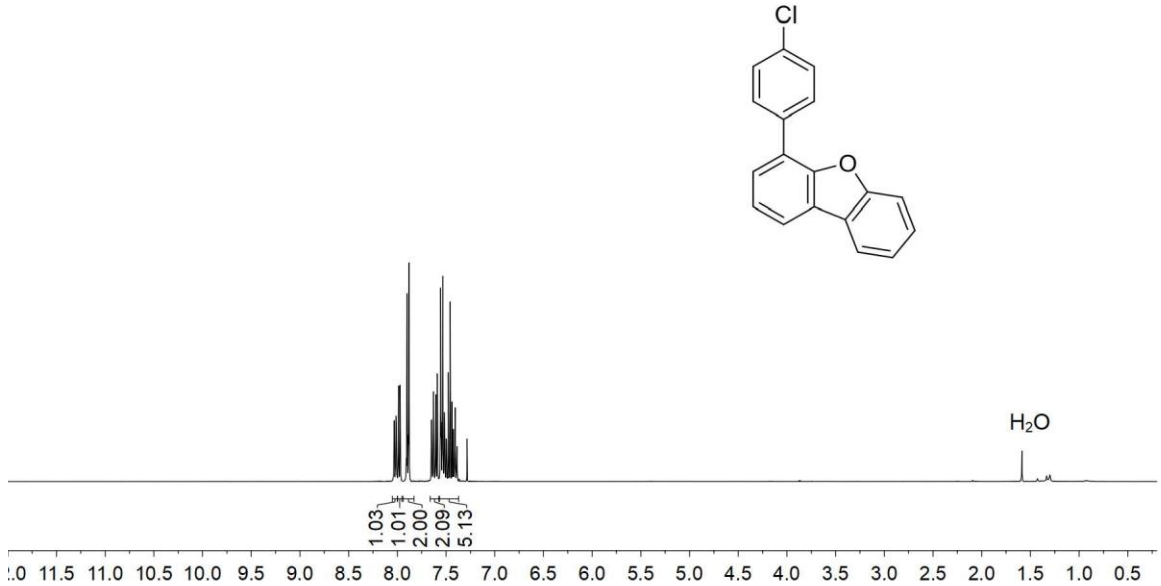

Cl
O
H2O
1.03
1.01
2.00
2.09
5.13
11.5
11.0
10.5
10.0
9.5
9.0
8.5
8.0
7.5
7.0
6.5
6.0
5.5
5.0
4.5
4.0
3.5
3.0
2.5
2.0
1.5
1.0
0.5

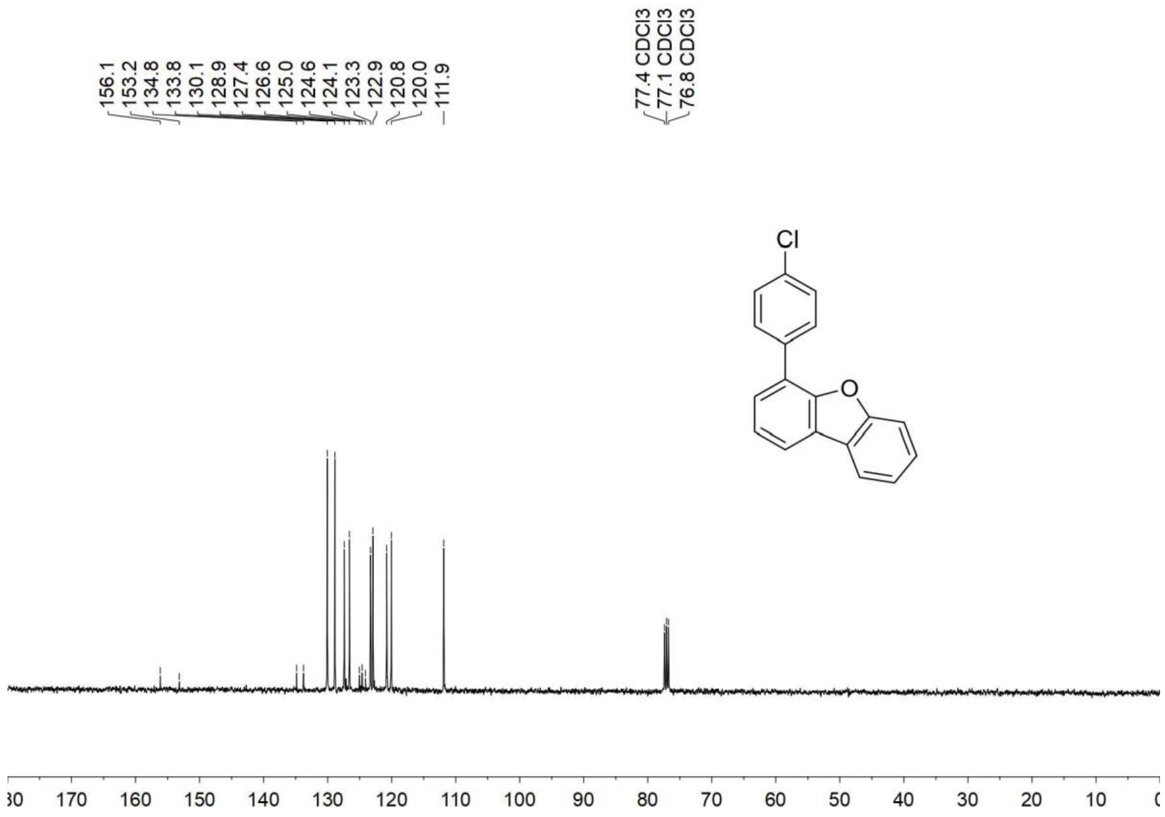
156.1
153.2
134.8
133.8
130.1
128.9
127.4
126.6
125.0
124.6
124.1
123.3
122.9
120.8
120.0
111.9
77.4 CDCl3
77.1 CDCl3
76.8 CDCl3
Cl
O
30
170
160
150
140
130
120
110
100
90
80
70
60
50
40
30
20
10

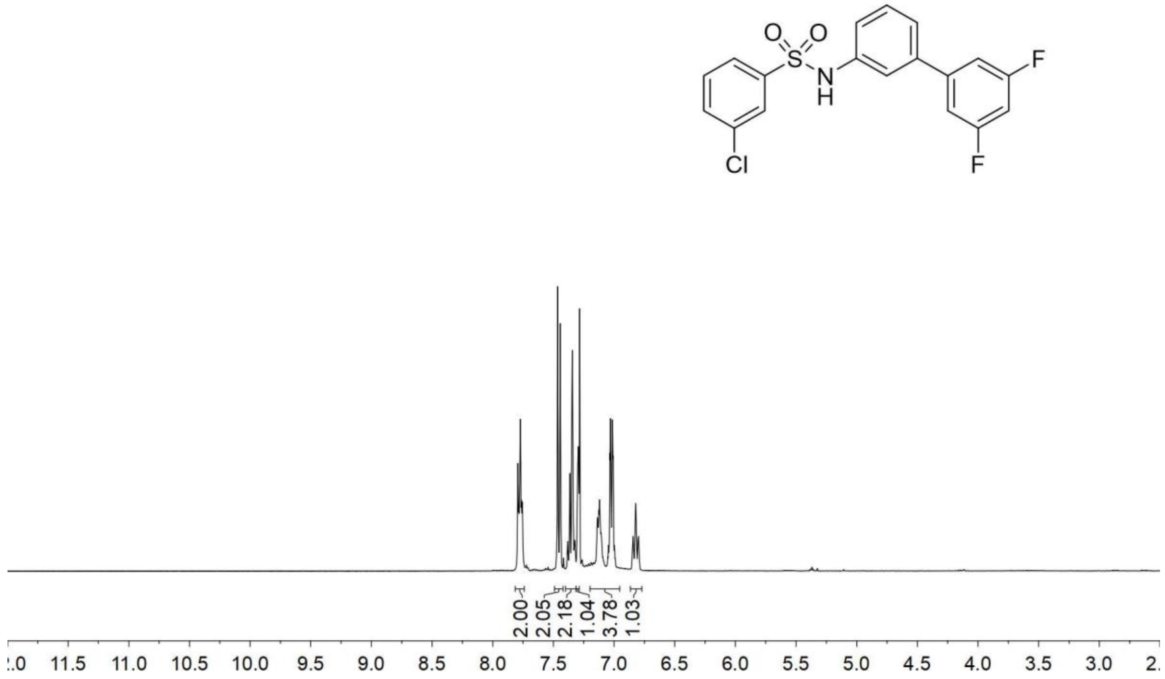
O
S
O
N
H
F
Cl
F
2.00
2.05
2.18
1.04
3.78
1.03
11.5
11.0
10.5
10.0
9.5
9.0
8.5
8.0
7.5
7.0
6.5
6.0
5.5
5.0
4.5
4.0
3.5
3.0

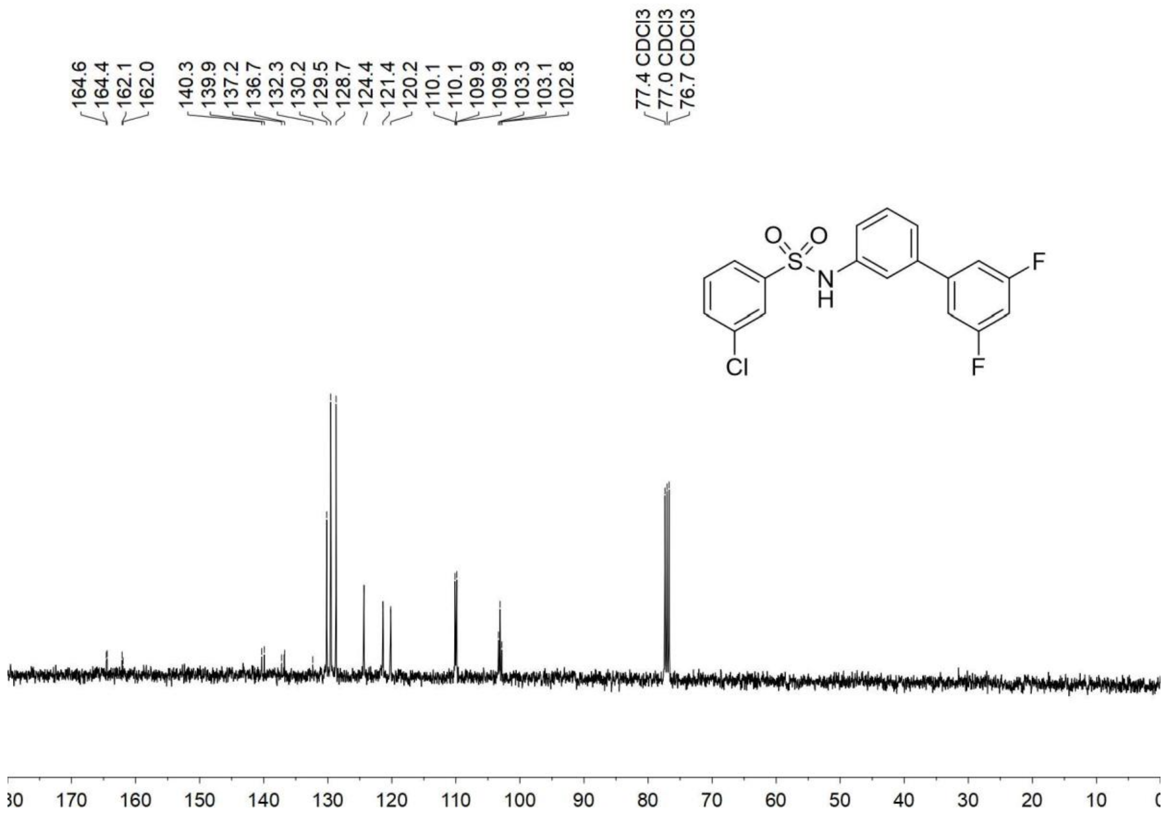
164.6
164.4
162.1
162.0
140.3
139.9
137.2
136.7
132.3
130.2
129.5
128.7
124.4
121.4
120.2
110.1
110.1
109.9
109.9
103.3
103.1
102.8
77.4 CDCl3
77.0 CDCl3
76.7 CDCl3
O
O
S
N
H
F
Cl
F
170
160
150
140
130
120
110
100
90
80
70
60
50
40
30
20
10

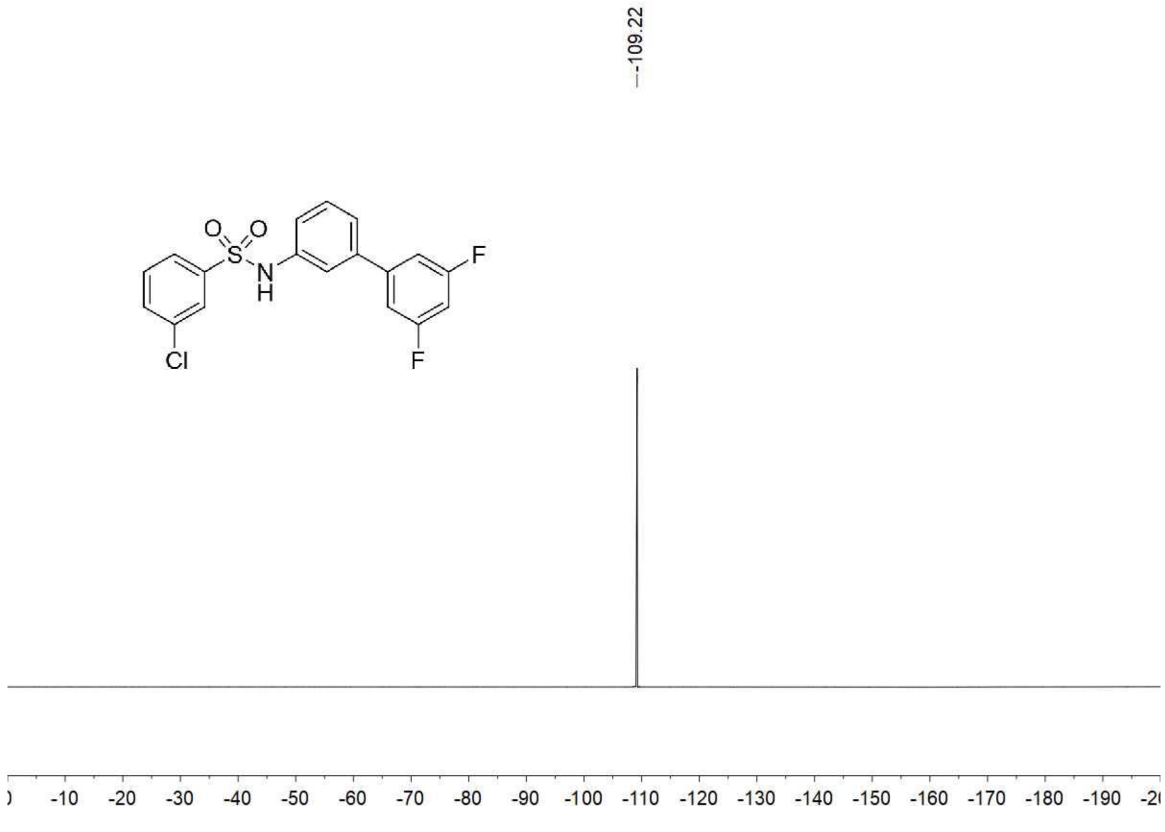
-109.22
O
O
S
N
H
F
Cl
F
-10
-20
-30
-40
-50
-60
-70
-80
-90
-100
-110
-120
-130
-140
-150
-160
-170
-180
-190

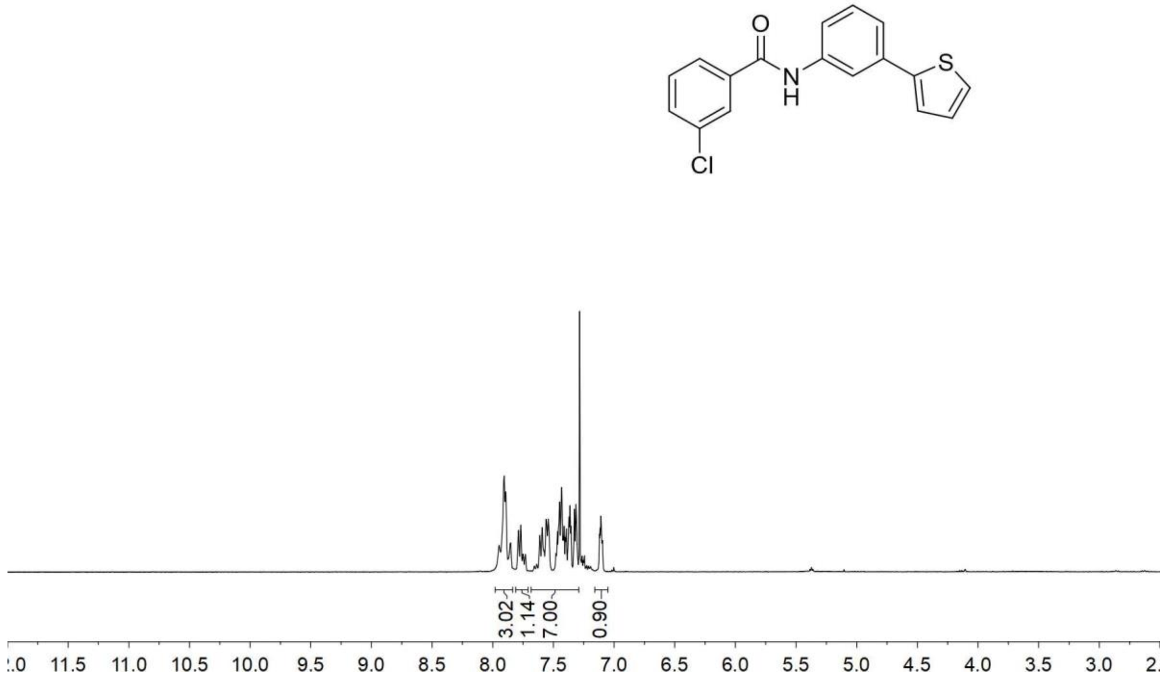
O
N
H
S
Cl
3.02
1.14
7.00
0.90
12.0 11.5 11.0 10.5 10.0 9.5 9.0 8.5 8.0 7.5 7.0 6.5 6.0 5.5 5.0 4.5 4.0 3.5 3.0 2.

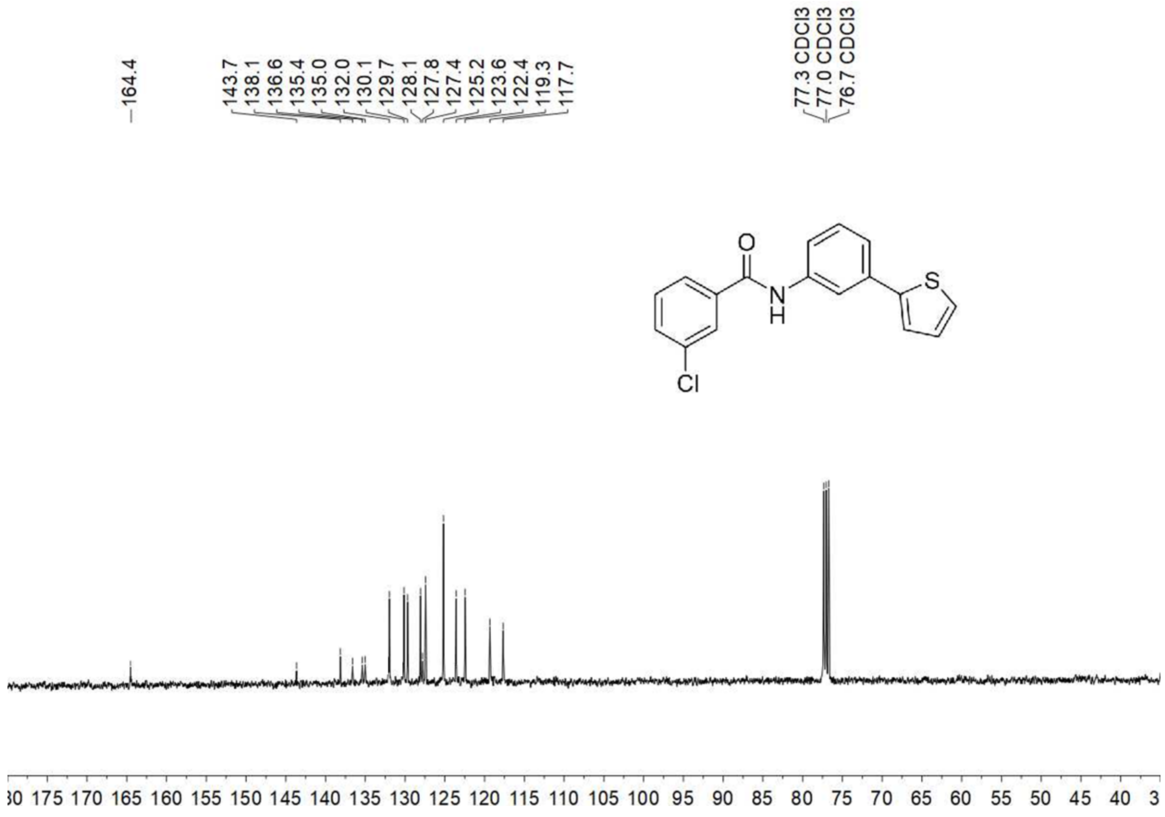
164.4
143.7
138.1
136.6
135.4
135.0
132.0
130.1
129.7
128.1
127.8
127.4
125.2
123.6
122.4
119.3
117.7
77.3 CDCl3
77.0 CDCl3
76.7 CDCl3
O
N
H
S
Cl
180 175 170 165 160 155 150 145 140 135 130 125 120 115 110 105 100 95 90 85 80 75 70 65 60 55 50 45 40 3

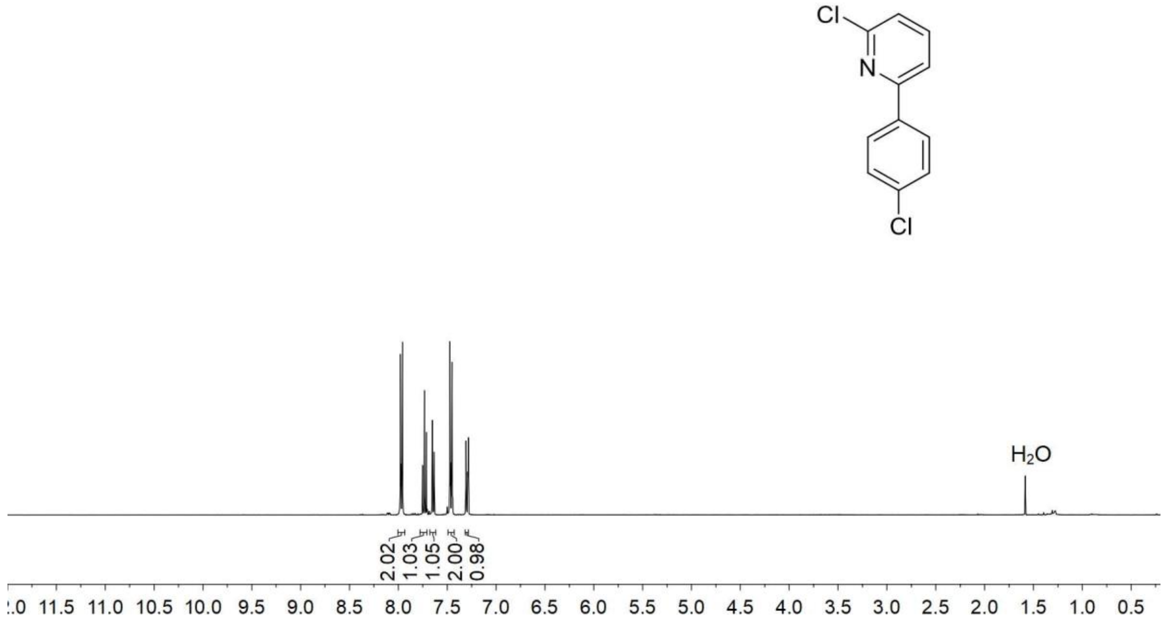
Cl
N
Cl
H2O
2.02
1.03
1.05
2.00
0.98
11.5 11.0 10.5 10.0 9.5 9.0 8.5 8.0 7.5 7.0 6.5 6.0 5.5 5.0 4.5 4.0 3.5 3.0 2.5 2.0 1.5 1.0 0.5

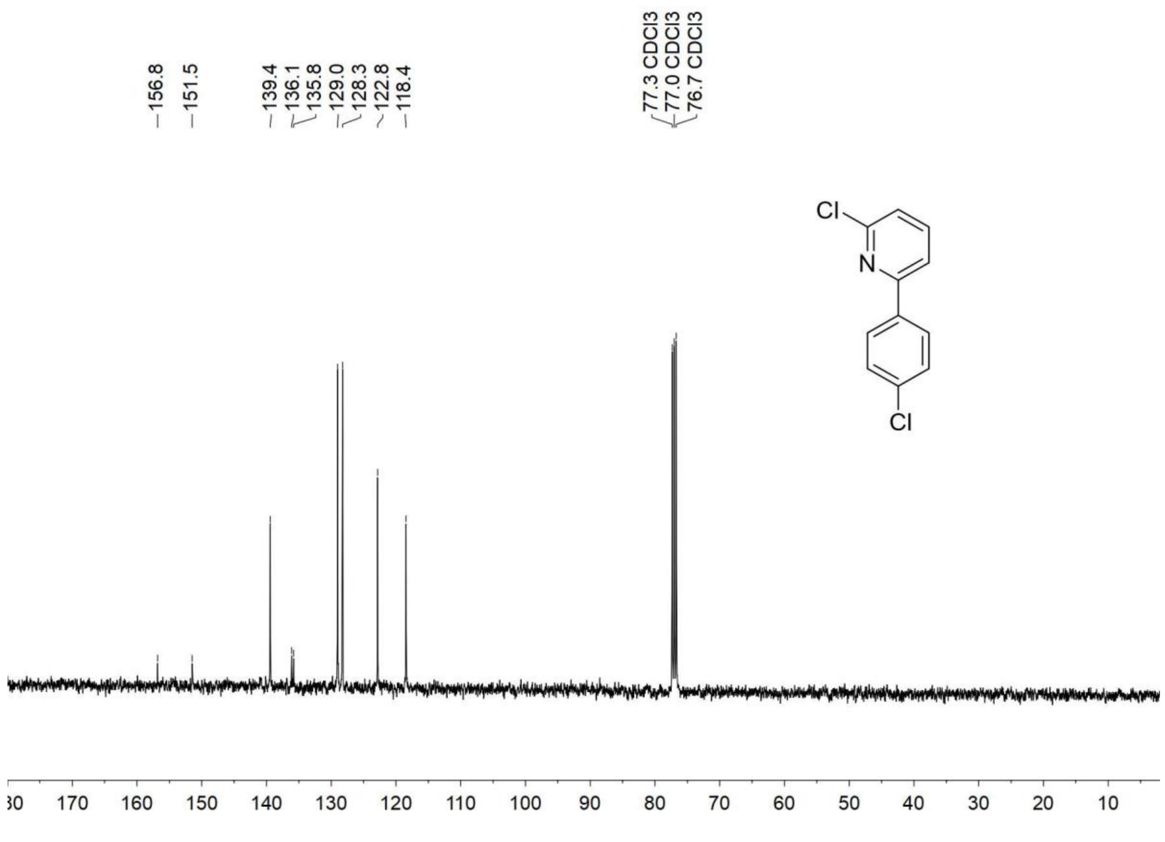
156.8
151.5
139.4
136.1
135.8
129.0
128.3
122.8
118.4
77.3 CDCl3
77.0 CDCl3
76.7 CDCl3
Cl
N
Cl
170 160 150 140 130 120 110 100 90 80 70 60 50 40 30 20 10

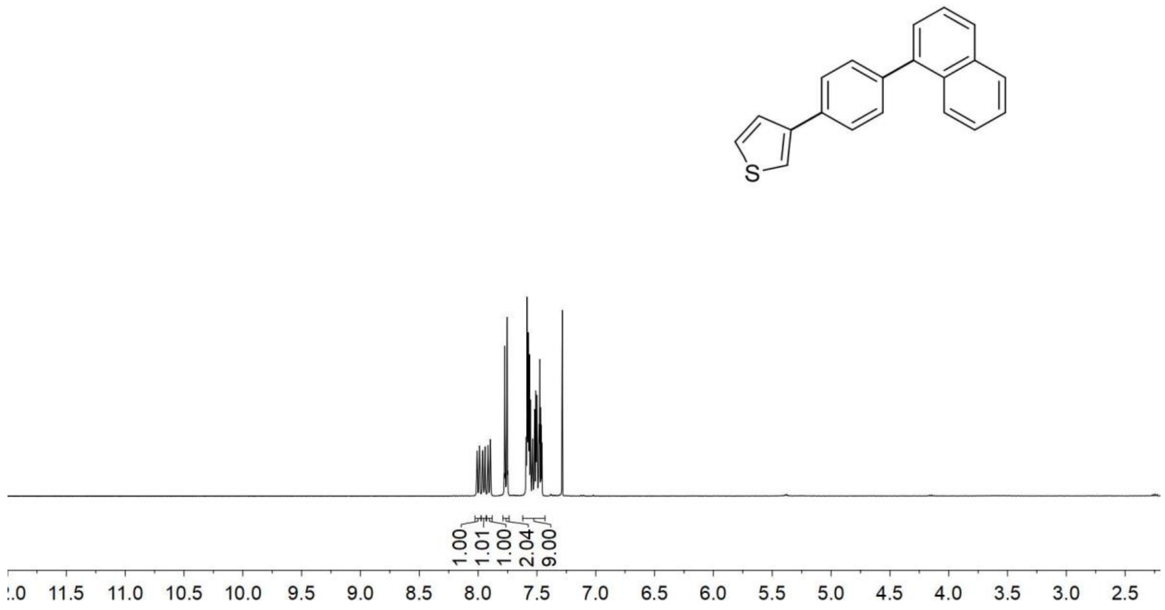
S
1.00
1.01
1.00
2.04
9.00
12.0 11.5 11.0 10.5 10.0 9.5 9.0 8.5 8.0 7.5 7.0 6.5 6.0 5.5 5.0 4.5 4.0 3.5 3.0 2.5

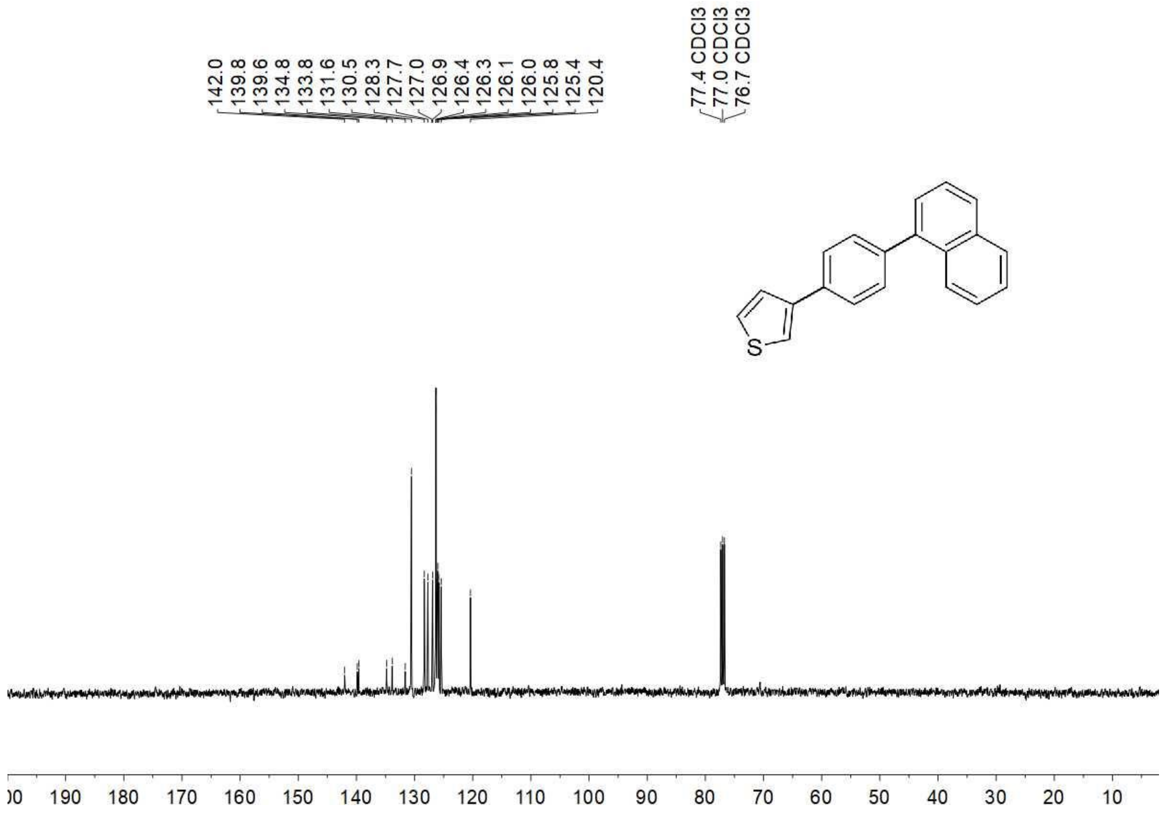
142.0
139.8
139.6
134.8
133.8
131.6
130.5
128.3
127.7
127.0
126.9
126.4
126.3
126.1
126.0
125.8
125.4
120.4
77.4 CDCl3
77.0 CDCl3
76.7 CDCl3
S
200 190 180 170 160 150 140 130 120 110 100 90 80 70 60 50 40 30 20 10

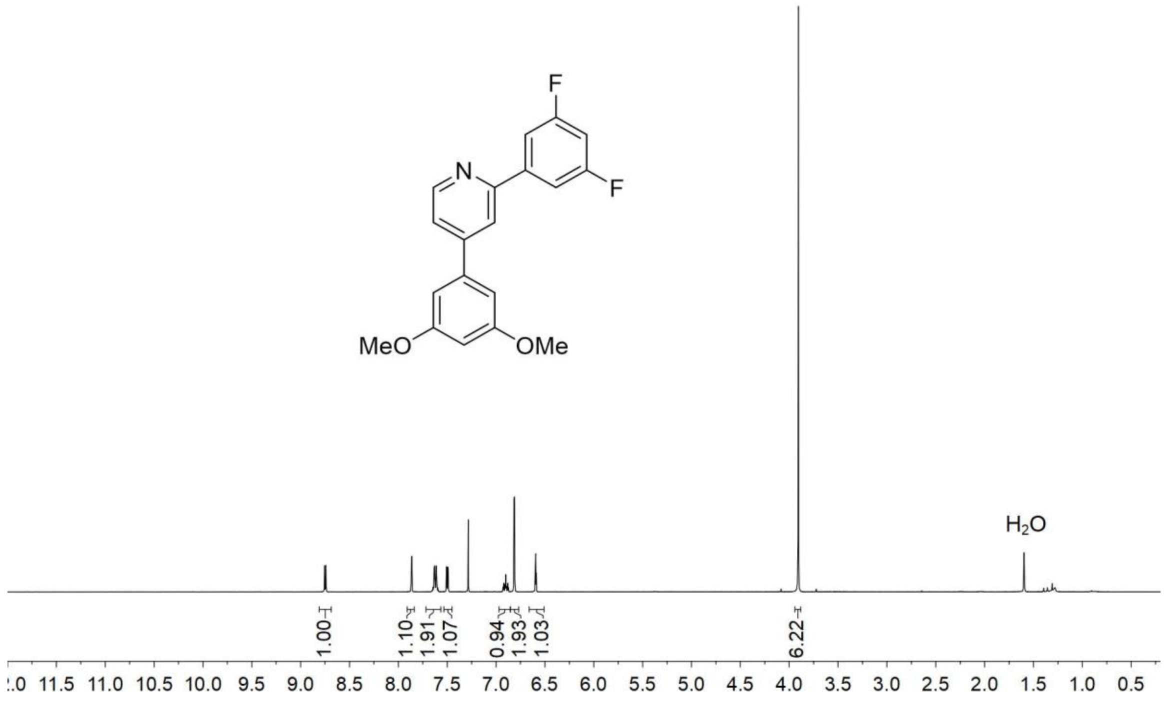
F
N
F
MeO
OMe
H2O
1.00
1.10
1.91
1.07
0.94
1.93
1.03
6.22
11.5 11.0 10.5 10.0 9.5 9.0 8.5 8.0 7.5 7.0 6.5 6.0 5.5 5.0 4.5 4.0 3.5 3.0 2.5 2.0 1.5 1.0 0.5

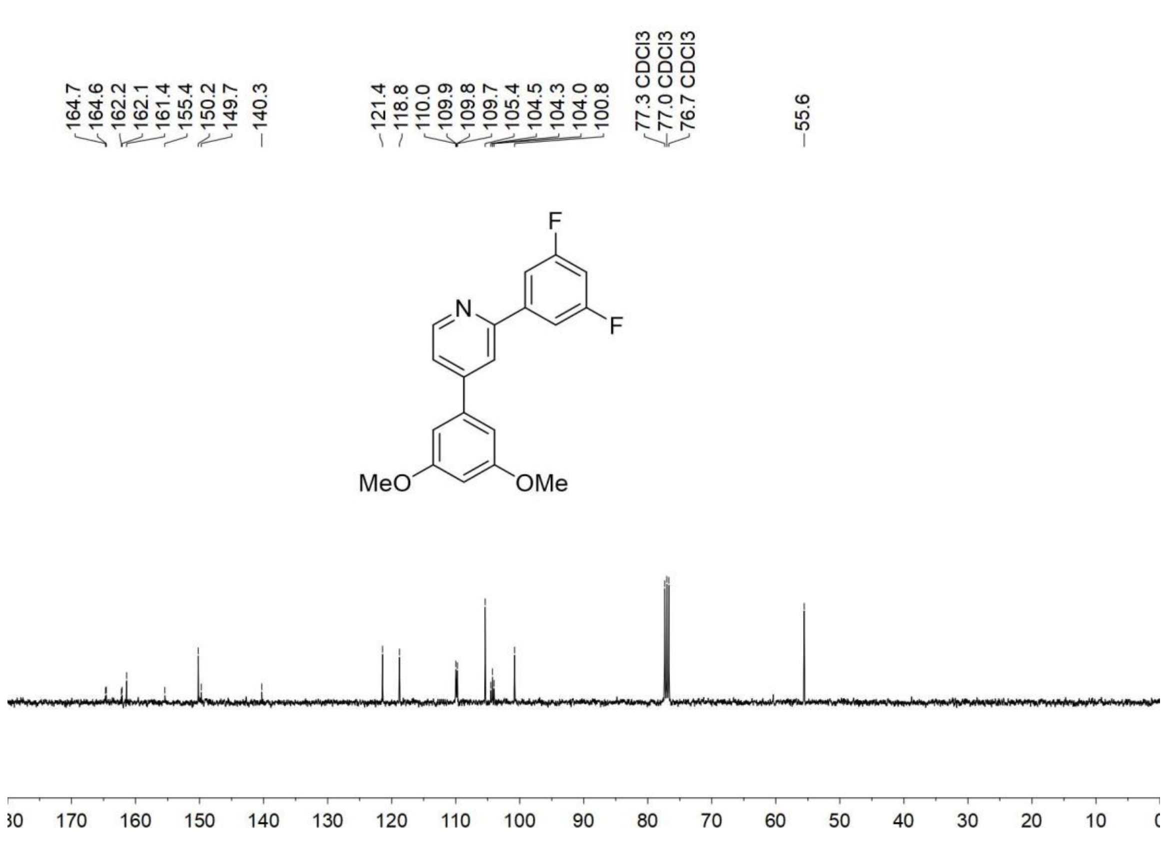
164.7
164.6
162.2
162.1
161.4
155.4
150.2
149.7
140.3
121.4
118.8
110.0
109.9
109.8
109.7
105.4
104.5
104.3
104.0
100.8
77.3 CDCl3
77.0 CDCl3
76.7 CDCl3
55.6
F
N
F
MeO
OMe
170 160 150 140 130 120 110 100 90 80 70 60 50 40 30 20 10

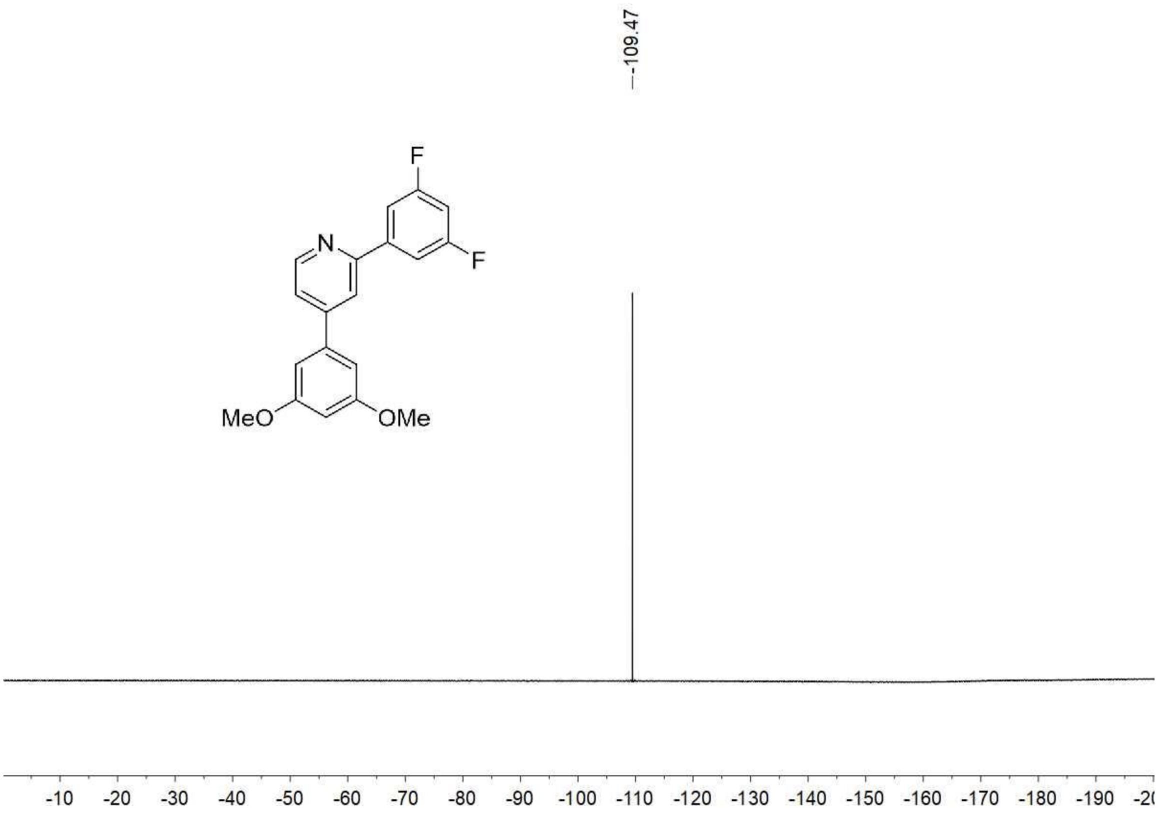

--109.47
F
N
F
MeO
OMe
-10
-20
-30
-40
-50
-60
-70
-80
-90
-100
-110
-120
-130
-140
-150
-160
-170
-180
-190

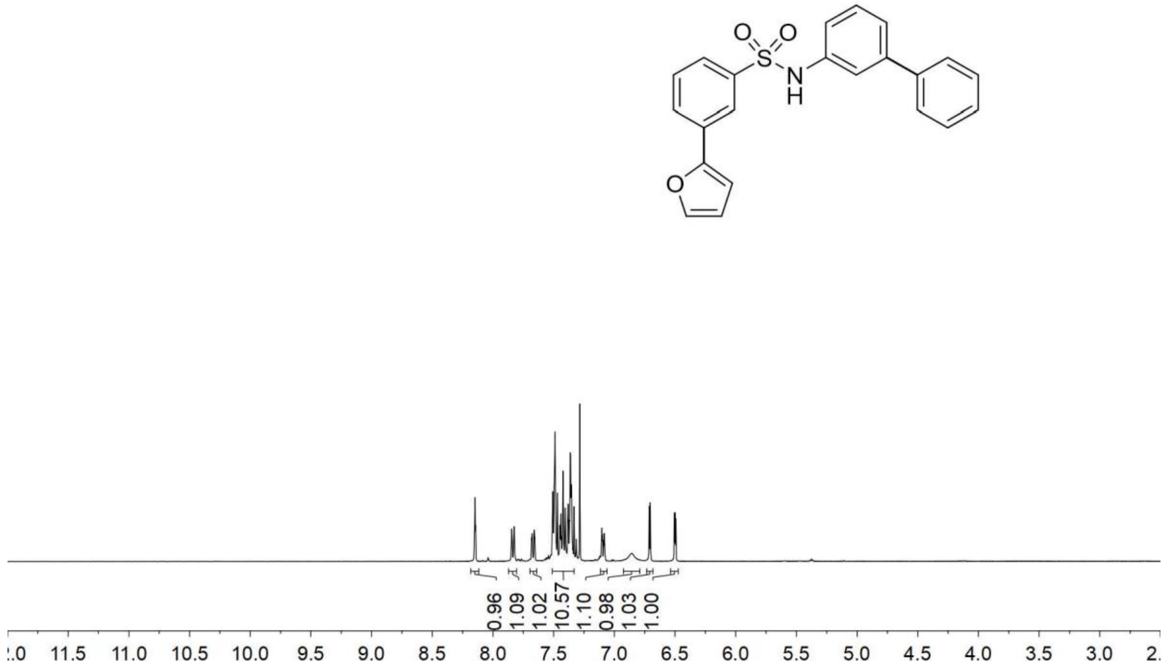

O
S
O
N
H
O
0.96
1.09
1.02
10.57
1.10
0.98
1.03
1.00
11.5
11.0
10.5
10.0
9.5
9.0
8.5
8.0
7.5
7.0
6.5
6.0
5.5
5.0
4.5
4.0
3.5
3.0

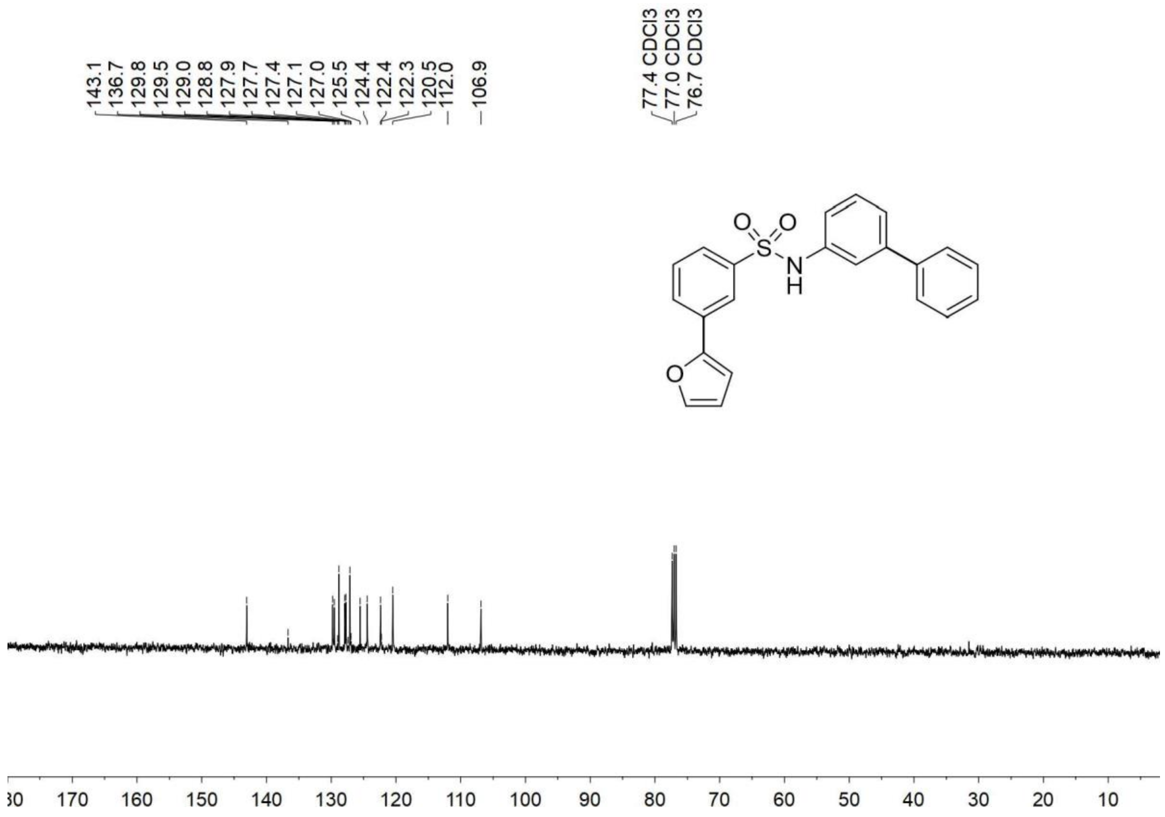
143.1
136.7
129.8
129.5
129.0
128.8
127.9
127.7
127.4
127.1
127.0
125.5
124.4
122.4
122.3
120.5
112.0
106.9
77.4 CDCl3
77.0 CDCl3
76.7 CDCl3
O
O
S
N
H
O
170 160 150 140 130 120 110 100 90 80 70 60 50 40 30 20 10

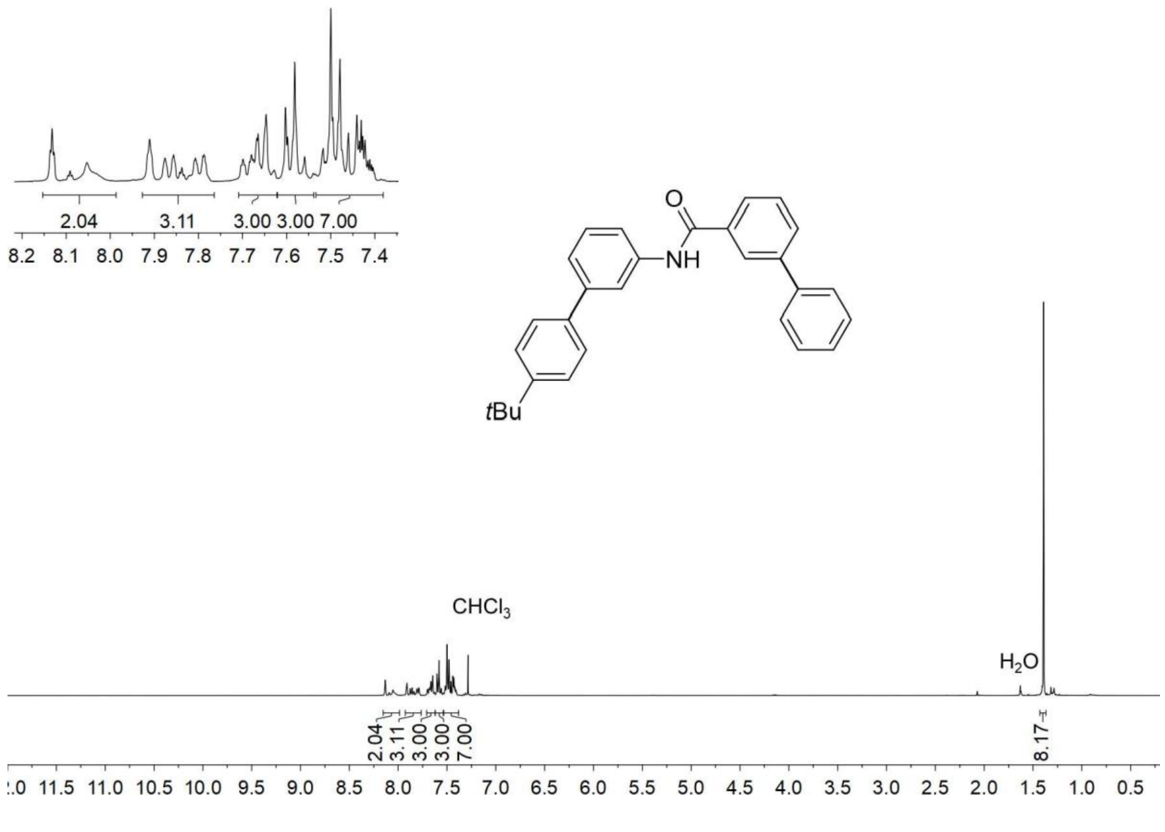
2.04 3.11 3.00 3.00 7.00
8.2 8.1 8.0 7.9 7.8 7.7 7.6 7.5 7.4
O
NH
tBu
CHCl3
H2O
2.04
3.11
3.00
3.00
7.00
8.17
11.5 11.0 10.5 10.0 9.5 9.0 8.5 8.0 7.5 7.0 6.5 6.0 5.5 5.0 4.5 4.0 3.5 3.0 2.5 2.0 1.5 1.0 0.5

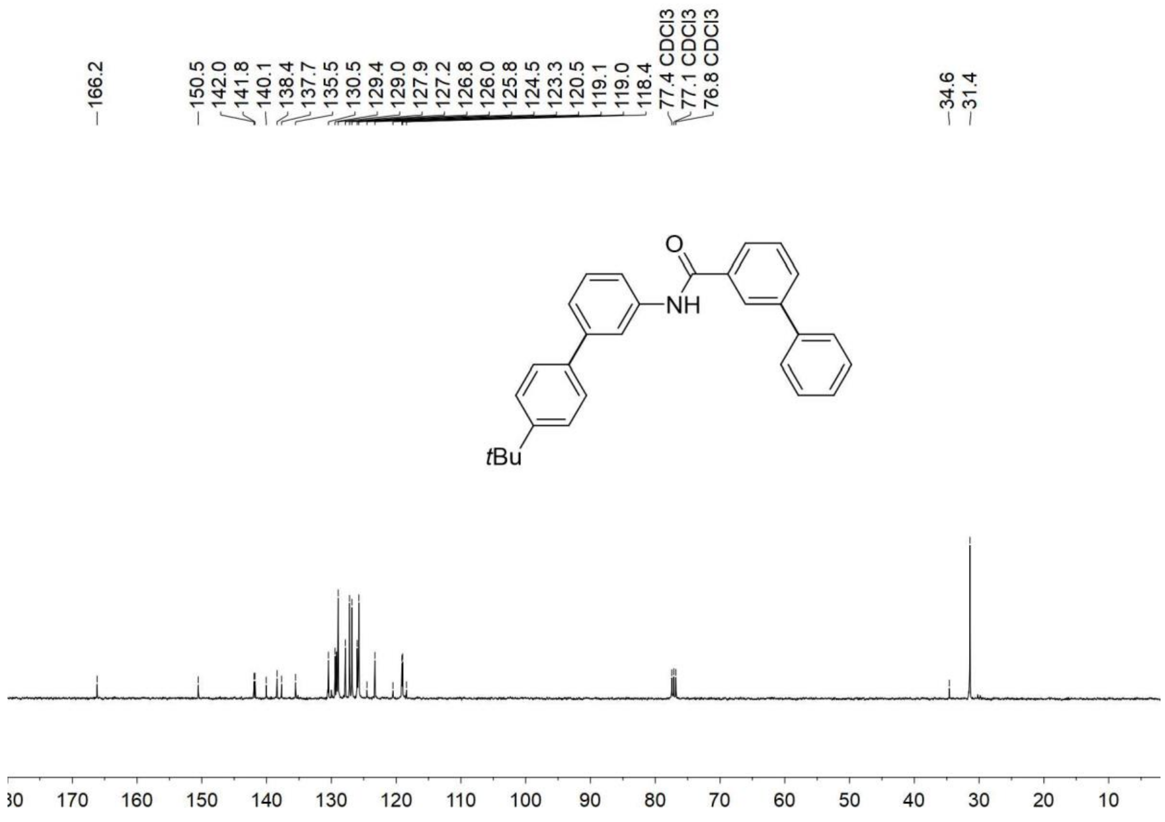

166.2
150.5
142.0
141.8
140.1
138.4
137.7
135.5
130.5
129.4
129.0
127.9
127.2
126.8
126.0
125.8
124.5
123.3
120.5
119.1
119.0
118.4
77.4 CDCl3
77.1 CDCl3
76.8 CDCl3
34.6
31.4
O
NH
tBu
170 160 150 140 130 120 110 100 90 80 70 60 50 40 30 20 10

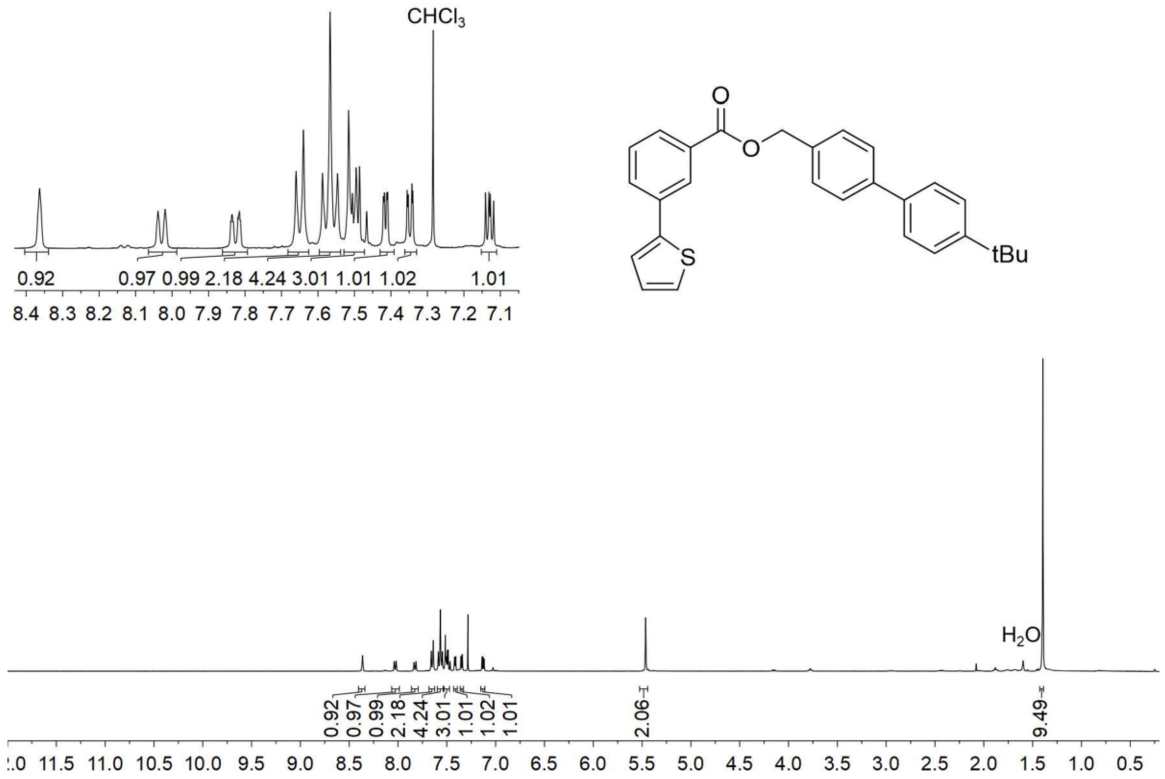

CHCl3
0.92 0.97 0.99 2.18 4.24 3.01 1.01 1.02 1.01
8.4 8.3 8.2 8.1 8.0 7.9 7.8 7.7 7.6 7.5 7.4 7.3 7.2 7.1
O
O
S
tBu
H2O
0.92
0.97
0.99
2.18
4.24
3.01
1.01
1.02
1.01
2.06
9.49
11.5 11.0 10.5 10.0 9.5 9.0 8.5 8.0 7.5 7.0 6.5 6.0 5.5 5.0 4.5 4.0 3.5 3.0 2.5 2.0 1.5 1.0 0.5

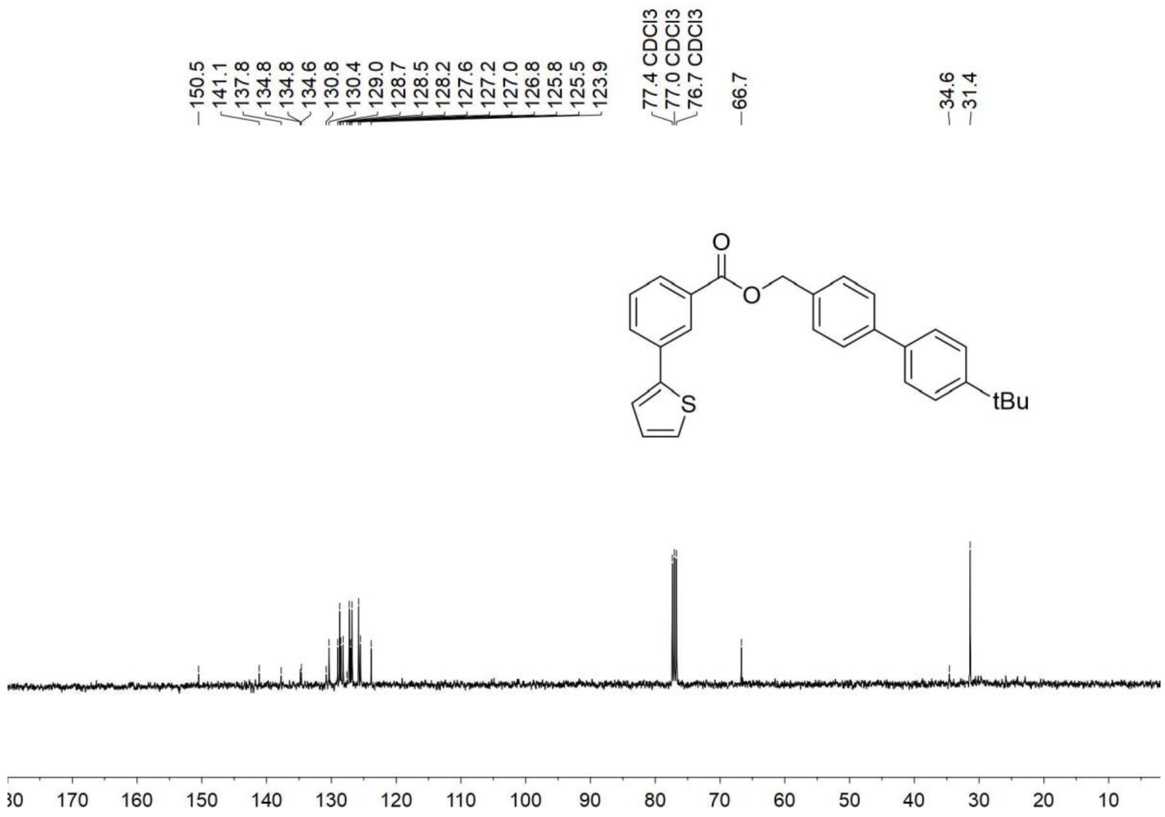
150.5
141.1
137.8
134.8
134.8
134.6
130.8
130.4
129.0
128.7
128.5
128.2
127.6
127.2
127.0
126.8
125.8
125.5
123.9
77.4 CDCl3
77.0 CDCl3
76.7 CDCl3
66.7
34.6
31.4
O
O
S
tBu
170 160 150 140 130 120 110 100 90 80 70 60 50 40 30 20 10

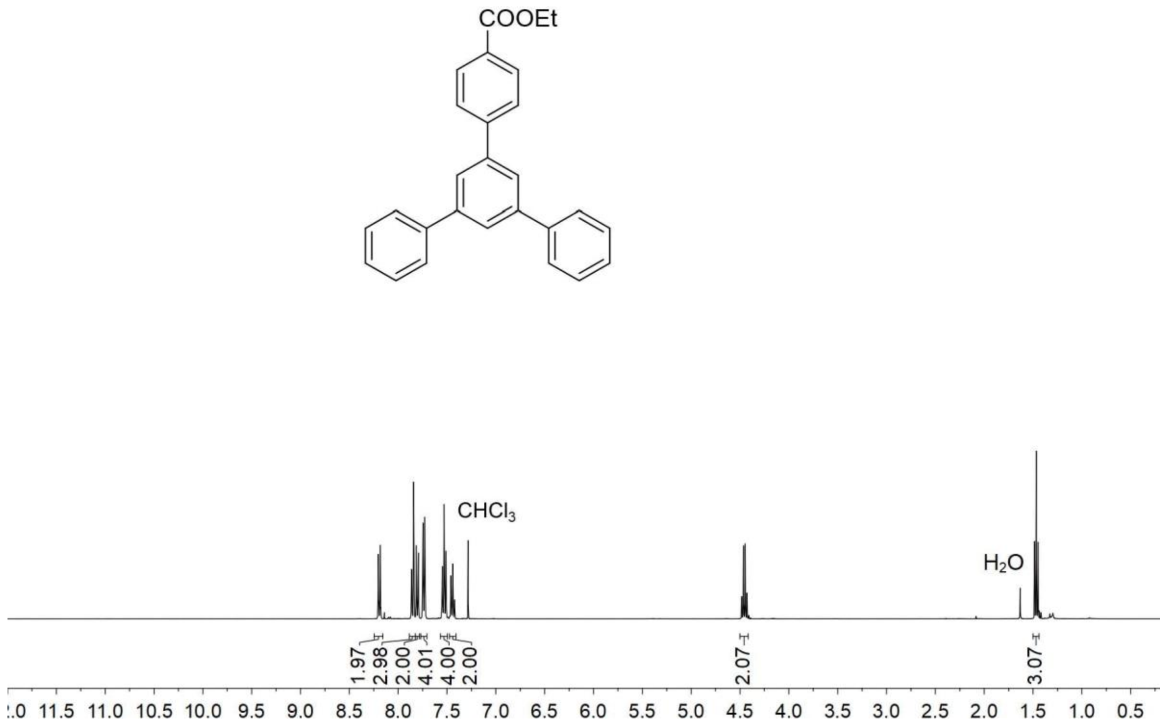
COOEt
CHCl3
H2O
1.97
2.98
2.00
4.01
4.00
2.00
2.07
3.07
11.5 11.0 10.5 10.0 9.5 9.0 8.5 8.0 7.5 7.0 6.5 6.0 5.5 5.0 4.5 4.0 3.5 3.0 2.5 2.0 1.5 1.0 0.5

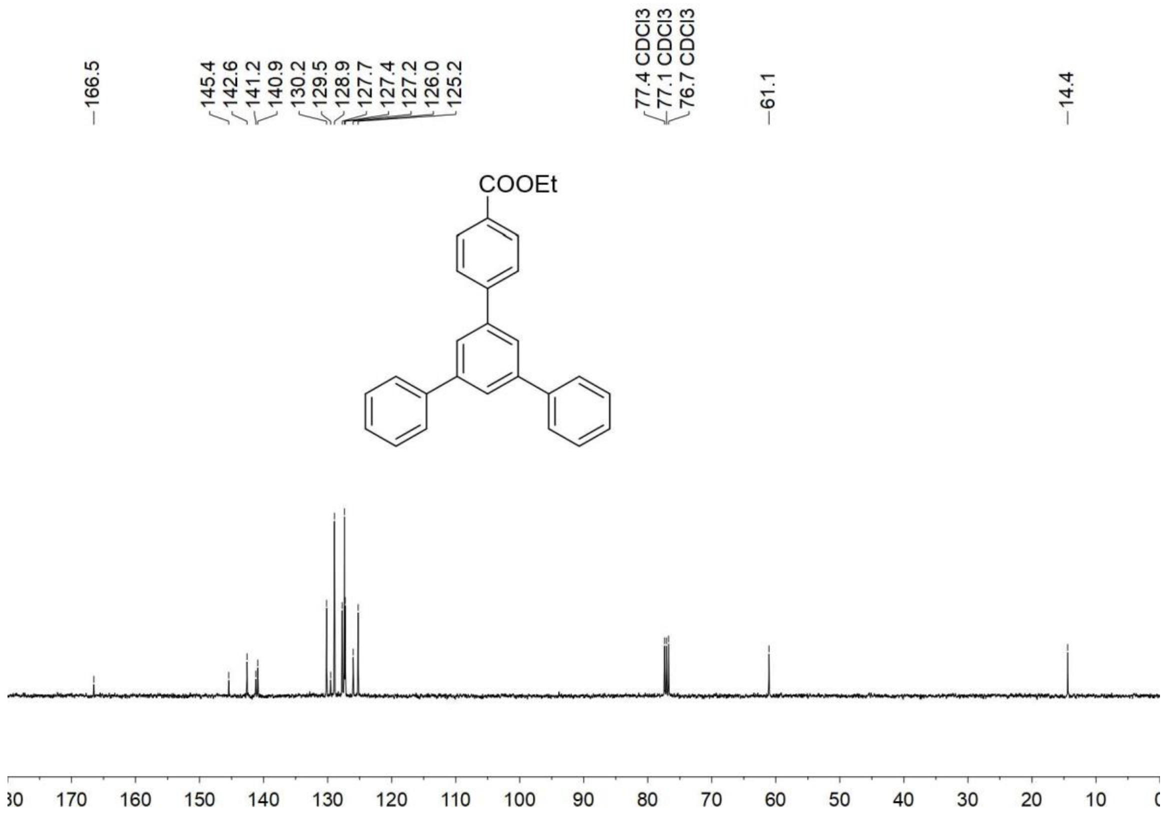
166.5
145.4
142.6
141.2
140.9
130.2
129.5
128.9
127.7
127.4
127.2
126.0
125.2
77.4 CDCl3
77.1 CDCl3
76.7 CDCl3
61.1
14.4
COOEt
170
160
150
140
130
120
110
100
90
80
70
60
50
40
30
20
10
